# Diastereoselective Synthesis of Silyl-Substituted Pyrrolidines

**DOI:** 10.1021/acs.joc.5c01023

**Published:** 2025-06-21

**Authors:** Davide Carboni, Giulio Casagranda, Simone Di Remigio, Alice Mirone, Arianna Quintavalla, Marco Lombardo

**Affiliations:** † Department of Chemistry “G. Ciamician”, 9296 Alma Mater Studiorum - University of Bologna, Bologna 40129, Italy; ‡ Center for Chemical Catalysis - C3, Alma Mater Studiorum - University of Bologna, Bologna 40129, Italy

## Abstract

In this paper, we report the synthesis and structural investigation of enantioenriched methylene isosteres of Hayashi–Jørgensen catalysts, and their application in organocatalysis. *N*-protected pyrrolidines **7b**–**d** were prepared in high yields and excellent diastereoselectivity using a new one-pot, four-step synthetic protocol involving: (a) the formation of a silyllithium reagent (**1**), (b) its addition to a diaryl olefin (**2**) to generate a silyl-substituted diphenylethyllithium intermediate (**3**), (c) the highly diastereoselective addition of this intermediate to a chiral sulfinimine (**4**), and (d) intramolecular cyclization to the desired products. After *N*-deprotection, the new catalysts **8** were further evaluated in benchmark Michael additions of aliphatic aldehydes to β-nitrostyrene, under various conditions, demonstrating reactivity and stereoselectivity comparable to the Hayashi catalyst. Notably, the trimethylsilyl derivative (*S*)-**8d** showed superior enantioselectivity, transferring its stereochemical information with remarkable efficiency (up to 99% *ee*). Structural studies through 2D-NMR and DFT calculations revealed different conformational preferences for the corresponding enamines, providing insight into the observed catalytic performance.

## Graphical abstract

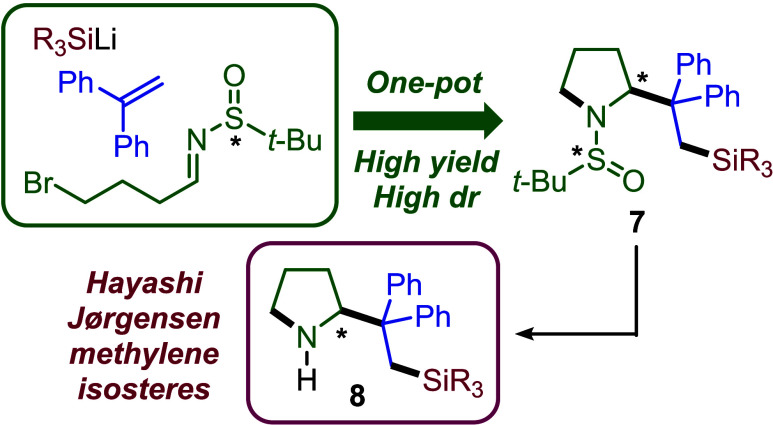

## Introduction

After Gilman’s seminal 1960 paper^1^ on the preparation of silyllithium compounds **1a,b** through the insertion of metallic lithium into the Si–Cl bond (Scheme 1A), these organometallic reagents have been extensively used for introducing silyl groups into organic molecules.^2–6^

**Scheme 1:**
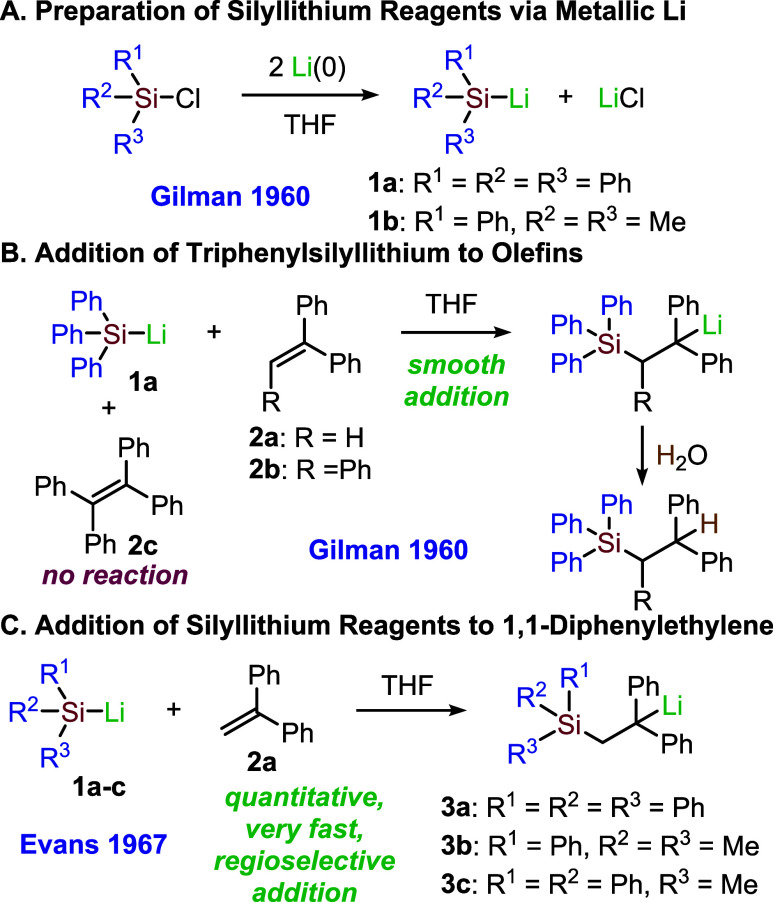
Preparation and Addition of Silyllithium Reagents to 1,1-Diphenylethylene

In the same year, Gilman also reported the addition of triphenylsilyllithium **1a** to olefins,^7^ observing excellent reactivity with 1,1-diphenylethylene (**2a**) and triphenyl-ethylene (**2b**), while noting that no addition occurred with the more hindered and highly conjugated tetraphenylethylene (**2c**, Scheme 1B). A few years later, Evans and co-workers extended this reactivity to the addition of phenyldimethyl-(**1b**) and methyldiphenylsilyl lithium (**1c**), besides triphenylsilyllithium (**1c**), to 1,1-diphenylethylene (**2a**) in tetrahydrofuran (THF) as the solvent, confirming that in all cases, the silyl group adds to the primary carbon of the olefinic reagent, leaving a stabilized diphenylethyllithium intermediate (**3a**–**c**, Scheme 1C).^8^ Interestingly, they also observed the immediate appearance of a deep red color when THF solutions of 1,1-diphenylethylene and the organosilyl-lithium reagents were mixed. Using stop-flow techniques and monitoring the change in optical density at 506 nm, they were able to determine the kinetic parameters for these very fast addition reactions.^7^

Surprisingly, after these seminal papers, the intermediate organolithium compounds **3a**–**c** have not been further explored. To the best of our knowledge, no reports on the use of these reagents have appeared in the chemical literature up to date.

Oxygen-substituted diphenylmethyllithium reagents (**5a**–**b**, Scheme 2A) similar to **3** have been recently employed by Reddy and Prasad^9^ in the highly diastereoselective addition to bromo-substituted chiral sulfinimines **4**.^10^ This synthetic strategy, originally pioneered by Ruano and co-workers,^11^ and later adopted also by our group,^12^ enabled the synthesis of various functionalized nitrogen containing heterocyclic compounds (Scheme 2A). Based on these results, we have recently applied a similar strategy for the synthesis of chiral α-disubstituted β-homoprolines **6**,^13^ through the addition of organoindium or organozinc allylic reagents to the 4-bromobutanal-derived chiral sulfinimine **4a** (Scheme 2B).

**Scheme 2:**
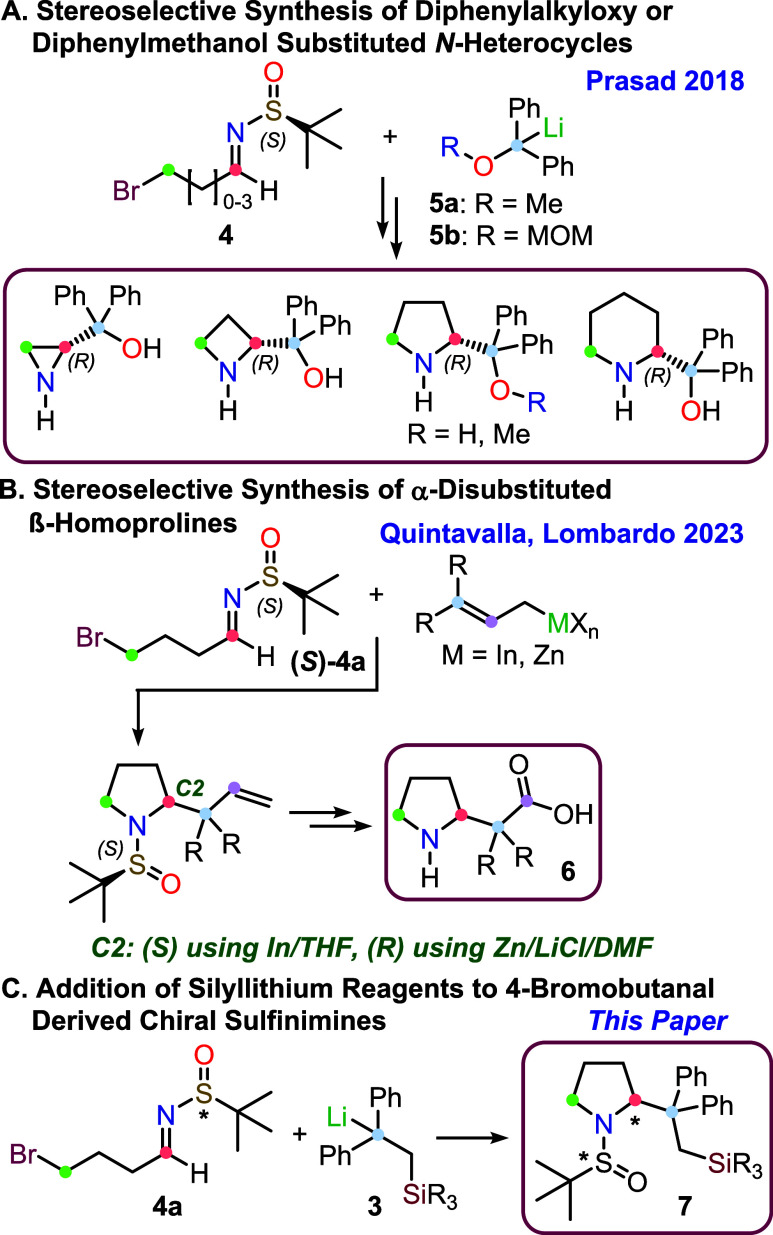
One-Pot Strategies for the Synthesis of Stereodefined Pyrrolidines

In this paper, we report the highly diastereoselective addition of silyl-substituted organolithium compounds **3** to 4-bromo-butanal derived chiral sulfinimine **4a**, for the synthesis of silicon-substituted pyrrolidines **7** (Scheme 2C). Notably, the corresponding unprotected pyrrolidines **8** are methylene isosteres of the well-known Hayashi–Jørgensen catalysts **9** (Figure 1).^14^ Thus, to gain further insight into the role of the oxygen atom in determining the catalytic activity and the selectivity of these extremely efficient organocatalysts, we finally investigated the reactivity and efficiency of the newly prepared methylene isosteres **8** in benchmark organocatalytic transformations, and completed the study by a series of combined 2D-NMR/computational DFT analyses. The replacement of the silicon–oxygen bond with a more robust silicon–carbon bond in catalysts **8** represents a significant structural modification that directly addresses some of the intrinsic limitations of the classical Hayashi–Jørgensen catalysts. These new derivatives offer enhanced stability under reaction conditions that typically lead to degradation of the parent organocatalysts, particularly in transformations requiring prolonged exposure to acids, water or oxidative reagents, while preserving the stereochemical environment essential for high levels of stereoselectivity.

**Figure 1:**
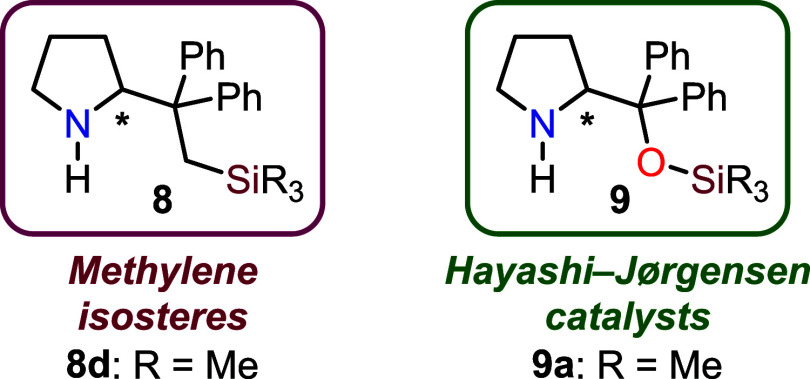
Structures of Hayashi–Jørgensen methylene isosteres **8**.

## Results and Discussion

The insertion of metallic lithium into the Si–Cl bond, enabling the rapid preparation of silyllithium reagents, requires at least one aromatic substituent on the silicon atom. Due to this requirement and its structural simplicity, the phenyldimethylsilyllithium derivative **1b** has been the most widely used silyllithium reagent in organic synthesis, from its discovery up to the present day. Furthermore, the preparation and reactivity of **1b** were studied in great detail by Fleming and co-workers in 1998.^15^ We started our investigation by preparing **1b** according to Fleming,^15^ adding it to 1,1-diphenylethylene **2a** following the procedure reported by Evans,^8^ and trapping the intermediate organolithium derivative **3b** with the chiral sulfinimine (*R*)-**4a** (Table 1). The imine **4a** was synthesized according to a literature procedure^9^ and was purified by flash chromatography on silica, before being stored at −20 °C. After a few days, we observed the formation of a white solid and signs of decomposition, confirmed by ^1^H NMR analysis of the sample. In many cases, after a week, the imine also developed an orange coloration. Concerned about the potential changes in its enantiomeric purity, we analyzed the imine before use. Chiral HPLC analysis revealed a noticeable decline in the enantiomeric excess, going from >99% *ee* in a freshly prepared sample to 96.6% *ee* after a few days of refrigerated storage.

**Table 1:**
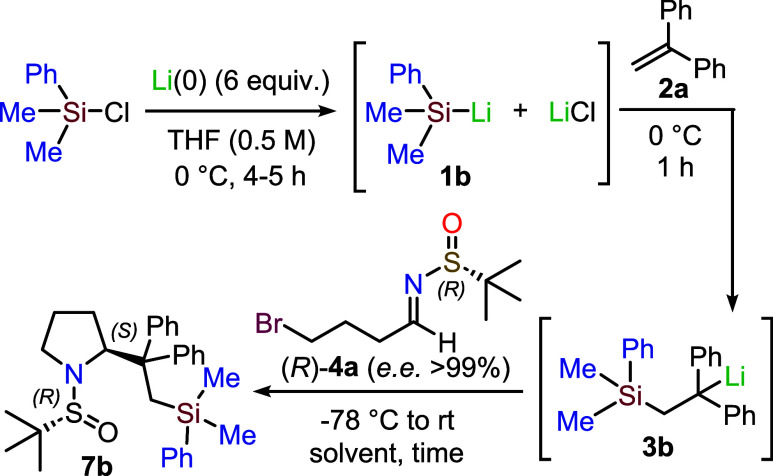
One-Pot Synthesis of Pyrrolidine **7b** ^a^

entry	solvent	time (h)	**7b** Y (%)^b^	*dr* ^c^
1	THF	4	62	99:1
2	THF/TMEDA	4	45	99:1
3	THF:Et_2_O (1:2)	12	66	>99:1
4	THF:toluene (1:2)	12	75	99:1
5	Trapp^d^	12	50	97:3

a **1b** (1.5 equiv., 1 mmol), solvent (2 mL), **2a** (1 equiv), (*R*)-**4a** (1.1 equiv); **4a** *ee* was determined by chiral HPLC analysis (OD-H column: flow rate 0.5 mL/min, *n*-hexane:isopropanol 9:1; see Supporting Infomation).

b Isolated yield after purification by flash chromatography.

c Determined by ^1^H NMR of the crude reaction mixture.

d THF:diethyl ether:*n*-pentane = 4:1:1, *T* = −110 °C; cooling bath: Et_2_O/liquid N_2_.

When the enantiopure imine **4a** (*ee* > 99%) was subjected to the reaction conditions outlined in Entry 1 of Table 1, using THF as the solvent, we were pleased to isolate the desired pyrrolidine **7b** in 62% overall yield. This is a notable result, considering that the product was obtained via a four-step, one-pot procedure without any intermediate isolation. Furthermore, the observed diastereomeric ratio (99:1) closely mirrored the enantiomeric purity of the starting imine (>99%), indicating a highly stereoselective addition to **4a**.

When TMEDA (*N*,*N*,*N'*,*N'*-tetramethylethylenediamine, 1 equivalent) was introduced in the third step, just before the addition of the imine **4a** and following the protocol of Reddy and Prasad,^9^ the isolated yield of **7b** decreased significantly to 45% (Table 1, Entry 2), while the diastereomeric ratio remained unchanged. When Et_2_O was used as a cosolvent (Table 1, Entry 3), a longer reaction time (12 h) was required to achieve a comparable yield (66%) to the one obtained using THF alone, but the diastereomeric ratio was nearly complete (>99:1). Notably, using toluene as the cosolvent (Table 1, Entry 4) led to the highest yield (75%), while maintaining a high diastereomeric ratio (99:1). Performing the reaction in the Trapp mixture at −110 °C (Table 1, Entry 5) resulted in a significantly lower yield after 12 h (50%), without any improvement in the diastereomeric ratio (97:3). The optimized reaction conditions from Table 1 (Entry 3, giving priority to stereoselectivity) were also applied using diphenylmethylsilyl chloride as the starting material, yielding the corresponding pyrrolidine **7c** in 55% purified yield, and with a considerable 97:3 diastereomeric ratio. Unfortunately, column chromatography did not allow for a complete separation of diastereoisomers **7c**, although a partial enrichment was successfully achieved after several attempts.

The (*S*) stereochemistry of the newly formed stereocenter (C2) can be inferred by considering that silyllithium reagents preferentially react adopting an open antiperiplanar transition state in the presence of stoichiometric amounts of coordinating LiCl (Figure 2),^16^ regardless of the nature of the solvent used (Table 1, Entries 1–5). We recently observed a very similar effect in the addition of organozinc reagents to the same sulfinimine **4a**.^13^

**Figure 2:**
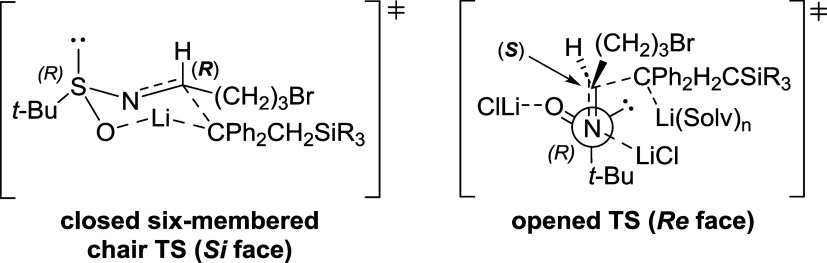
Closed and open transition states involved in the addition of silyllithium reagents to imine (*R*)-**4a**.

In 1976, Still proposed a rapid and very convenient preparation of trimethylsilyllithium (**1d**),^17^ by reacting a slight excess of the commercially available hexamethyldisilane with one equivalent of methyllithium, in THF/HMPA (hexamethylphosphoric acid triamide), at 0 °C for 10 min. This reaction was very fast, with the formation of the silyl anion evidenced by the immediate appearance of a deep red color in the solution. Following this procedure, we extended our one-pot synthetic strategy to the first addition of a trialkylsilyllithium reagent to **2a**. After the one-pot addition of the intermediate organolithium **3d** to imine **4a**, we successfully isolated the corresponding pyrrolidine **7d** in 74% overall yield (Scheme 3). Since only the signals of the major diastereoisomer were detected in the ^1^H NMR spectrum of the crude reaction mixture, the stereoselectivity for this transformation (*dr* 97.5:2.5) was determined by chiral HPLC, after sulfinyl removal and nitrogen protection with benzoyl chloride (see Supporting Information).

**Scheme 3:**
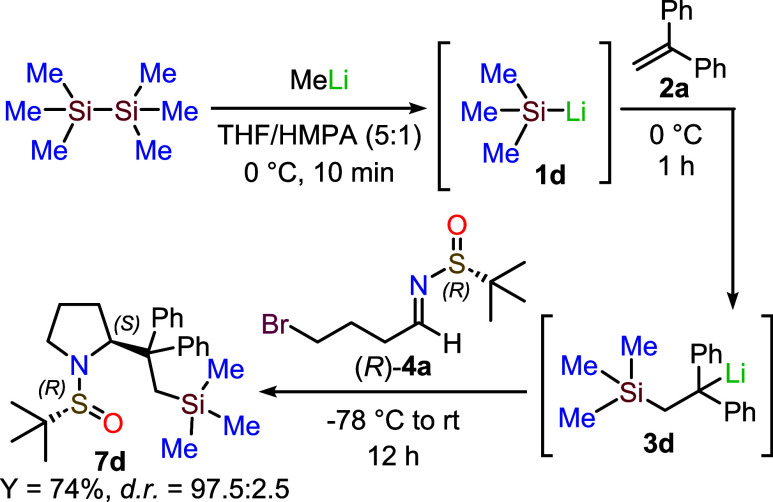
One-Pot synthesis of pyrrolidine **7d**

The use of a strongly dipolar aprotic cosolvent such as HMPA is essential to promote the addition of MeLi to one of the silicon atoms of the disilane. However, its presence also negatively affects the stereoselectivity of the addition of **3d** to **4a**. Despite starting with an enantiopure imine (*ee* > 99%), the final diastereomeric ratio was 97.5:2.5. Different from the previous protocol, this reaction does not produce LiCl in the reaction mixture. Thus, to assess the role of LiCl in determining the stereoselectivity of this transformation, we added one equivalent of the salt just before the addition of the imine **4a**. Unfortunately, this modification not only resulted in a lower yield (40%), but also significantly reduced the diastereomeric ratio (90:10). Finally, replacing THF with Et_2_O once again had a negative impact, decreasing both the final yield (60%) and to a lesser extent, also the diastereomeric ratio (94:6). Unfortunately, despite numerous attempts, it was not possible to separate diastereoisomer **7d** by column chromatography.

To further demonstrate the versatility and robustness of our proposed synthetic protocol, we extended its application by reacting intermediate **3b** with chiral imines (*R*)-**4b** and (*R*)-**4c** (Figure 3). These imines possess side chains that are one carbon shorter and one carbon longer, respectively, compared to (*R*)-**4a**. The reaction with the shorter side chain (*R*)-**4b** yielded azetidine **10** in moderate yield (25%), due to the competitive HBr elimination in the presence of the organolithium reagent, but with excellent diastereoselectivity (*dr* 99:1). On the other hand, using the longer side chain imine (*R*)-**4c**, piperidine **11** was obtained in yield comparable to those of the pyrrolidine derivatives (69%), albeit with a slightly lower diastereoselectivity (*dr* 94:6, Figure 3).

**Figure 3:**
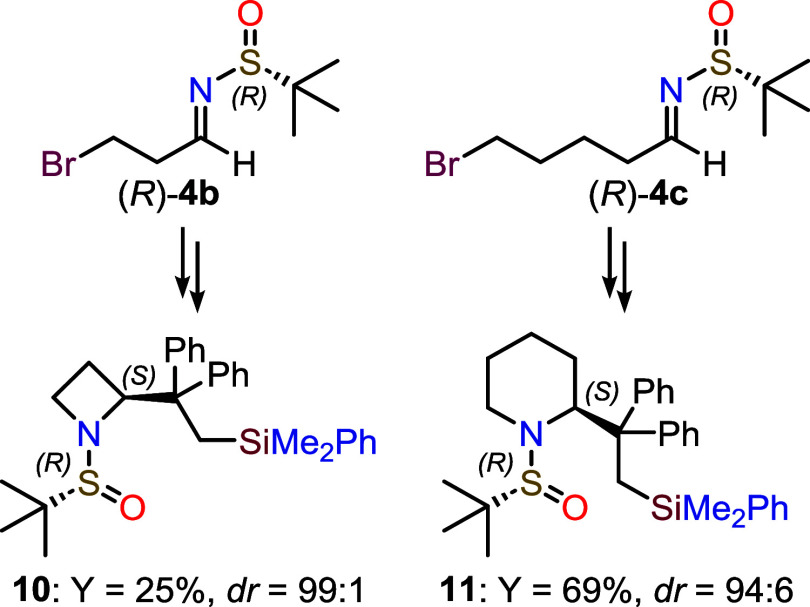
Structures of azetidine **10** and piperidine **11**.

After obtaining pyrrolidines **7b**–**d** in satisfactory yields and excellent diastereomeric ratios, the unprotected analogues **8b**–**d** were readily prepared in high purified yields, using standard conditions (acetyl chloride/methanol, Scheme 4). Their enantiomeric purity was determined by chiral HPLC after derivatization with benzoyl chloride (see Supporting Information).

**Scheme 4:**
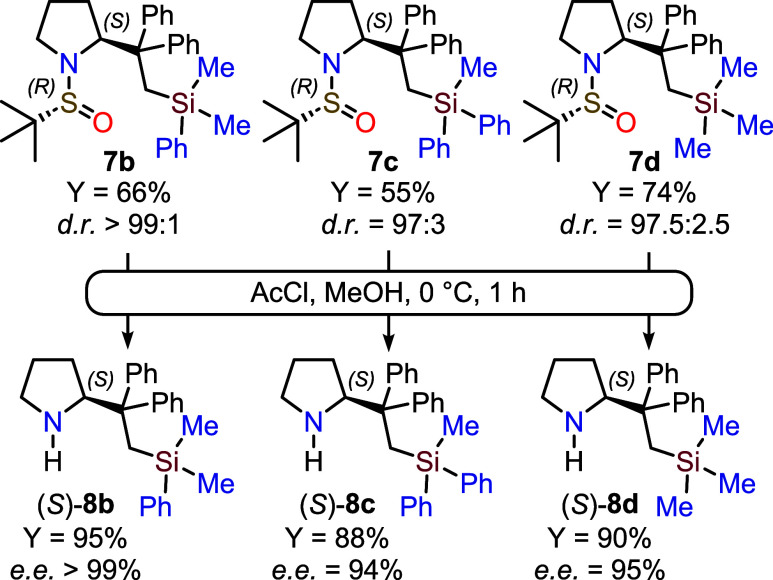
Synthesis of Enantioenriched Pyrrolidines **8b**–**d**

With the enantioenriched methylene isosteres of Hayashi–Jørgensen catalysts in hand (**8b**–**d**), we decided to evaluate them in the well-known benchmark Michael addition of aliphatic aldehydes **12a** and **12b** to β-nitrostyrene **13**. We first compared the reactivity of our catalysts with α,α-diphenyl-2-pyrrolidine methanol trimethylsilyl ether (**9a**, R = Me, Figure 1), using propanal (**12a**) under the conditions developed by Seebach and Hayashi in 2011, both with and without *p*-nitrophenol (PNP) as the cocatalyst (Table 2).^18^

**Table 2:**
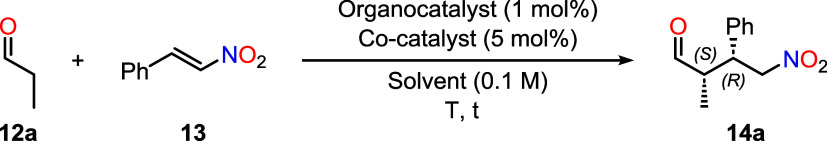
Benchmark Organocatalyzed Michael Addition of Propanal **12a** to β-Nitrostyrene **13** ^a^

entry	organocatalyst (*ee*)	co-catalyst	solvent	time (h)	*T* (°C)	conversion (%)^b^	**14a***d. r.* (*syn*:*anti*)^c^	**14a***ee* (%)^d^
1	(*S*)-**9a** (>99%)		*n*-hexane	3	rt	>99	72:28	>99
2	(*S*)-**8b** (>99%)		*n*-hexane	3	rt	>99	91:9	97
3	(*S*)-**8b** (>99%)		*n*-hexane	6	0	60	87:13	97
4	(*S*)-**8c** (94%)		*n*-hexane	2	rt	>99	85:15	91 (97)^e^
5	(*S*)-**8d** (95%)		*n*-hexane	3	rt	>99	81:19	94 (99)^e^
6	(*S*)-**9a** (>99%)	PNP	*n*-hexane	0.3	rt	>99	87:13	>99
7	(*S*)-**8b** (>99%)	PNP	*n*-hexane	0.75	rt	>99	79:21	97
8	(*S*)-**8d** (95%)	PNP	*n*-hexane	0.6	rt	>99	73:27	94 (99)^e^
9	(*S*)-**9a** (>99%)		toluene	3	rt	>99	93:7	>99
10	(*S*)-**8b** (>99%)		toluene	3	rt	25	93:7	97
11	(*S*)-**8b** (>99%)		toluene	6	rt	59	95:5	97
12	(*S*)-**8d** (95%)		toluene	3	rt	55	91:9	95 (>99)^e^

a **13** (0.1 mmol), solvent (1 mL), **12a** (1.5 equiv).

b Determined by ^1^H NMR analysis of the crude reaction mixture, by integrating the signals of **13** and **14a**.

c Determined by ^1^H NMR analysis of the crude reaction mixture, by integrating the signals of the formyl group of the diastereomeric products.

d Determined by chiral HPLC analysis (IC column: flow rate 1 mL/min, *n*-hexane:isopropanol 9:1; see Supporting Information).

e Values in parentheses are the final enantiomeric excess normalized considering the enantioenrichment of the used organocatalyst.

When the Hayashi catalyst (*S*)-**9a** was used in *n*-hexane at 1 mol %, the desired product **14a** formed quantitatively after 3 h at room temperature, with a 72:28 *syn*:*anti* ratio and, as expected, complete enantioselectivity for the major diastereoisomer (*ee* >99%, Entry 1, Table 2). Under the same conditions, (*S*)-**8b** proved at least as reactive as (*S*)-**9a** and significantly more diastereoselective (*syn*:*anti* = 91:9), although slightly less enantioselective (*ee* 97%, Entry 2, Table 2). When (*S*)-**8b** was reacted at 0 °C, the conversion resulted 60% after 6 h, with a slight decrease in diastereoselectivity (*syn*:*anti* = 87:13), and no improvement in enantioselectivity (*ee* 97%, Entry 3, Table 2). It is well established that lower diastereoselectivity values in these transformations are associated with slower reaction rates, as prolonged reaction times allow the final product **14a** to react with the organocatalyst, forming the corresponding enamine and eroding the stereochemistry at C2.^18^

Under the same reaction conditions, (*S*)-**8c** was highly reactive, but less diastereoselective than (*S*)-**8b**, while affording the same results in terms of enantioselectivity (normalized *ee* 97%, Entry 4, Table 2). Similarly, (*S*)-**8d** reacted smoothly, but pleasingly displayed the highest enantioselectivity (normalized *ee* 99%, Entry 5, Table 2). The diastereoselectivity obtained with this catalyst was the lowest among the series (*syn*:*anti* = 81:19, Entry 5, Table 2), yet still better than that observed with (*S*)-**9a**. Under the optimized Seebach-Hayashi conditions, using *p*-nitrophenol as a cocatalyst (PNP, 5 mol %) and *n*-hexane as the solvent, (*S*)-**9a** was confirmed to be extremely reactive, affording product **14a** quantitatively in just 20 min, with complete enantioselectivity (*ee* > 99%) and an 87:13 *syn*:*anti* ratio (Entry 6, Table 2). Under these conditions, both (*S*)-**8b** and (*S*)-**8d** were slightly less reactive. While (*S*)-**8b** afforded a lower enantioselectivity (*ee* 97%, Entry 7, Table 2), comparable to that obtained without the cocatalyst, (*S*)-**8d** once again transferred the stereochemical information from the catalyst to the product almost completely (normalized *ee* 99%, Entry 8, Table 2). In both cases, the diastereomeric ratio was lower than that observed using (*S*)-**9a**. However, as previously mentioned, this may be attributed to the longer reaction times required for (*S*)-**8b** and (*S*)-**8d** to reach full conversion. When the catalysts were tested in toluene as the solvent, without any cocatalyst, the Hayashi catalyst (*S*)-**9a** proved significantly more reactive than both (*S*)-**8b** and (*S*)-**8d**, requiring just 3 h to achieve a complete conversion and affording product **14a** with complete enantioselectivity (*ee* > 99%) and a 93:7 *syn*:*anti* ratio (Entry 9, Table 2). After 3 h, (*S*)-**8b** gave only 25% conversion (Entry 10, Table 2), while (*S*)-**8d** was slightly more reactive, reaching 55% conversion (Entry 12, Table 2). Extending the reaction time to 6 h increased the conversion using (*S*)-**8b** to 59% (Entry 11, Table 2), though still far from the quantitative result obtained with the Hayashi catalyst. On the other hand, (*S*)-**8b** displayed a very good diastereoselectivity, while its enantioselectivity remained unchanged at 97% (Entries 10–11, Table 2). Once again, (*S*)-**8d** furnished excellent results in terms of enantioselectivity (normalized *ee* > 99%), and a diasteroisomeric ratio comparable to that observed with the Hayashi catalyst (*S*)-**9a** (*syn*:*anti* = 91:9, Entry 12, Table 2).

Finally, since the parent (*S*)-**9a** and all our derivatives **8** favor the formation of the same enantiomer, this confirms the proposed assignment of the (*S*) configuration to the newly formed C2 stereocenter in the addition of silyllithium reagents to imine (*R*)-**4a**, further supporting the proposed open transition state model (Figure 2).

We further evaluated organocatalysts (*S*)-**8b**–**d** against the Hayashi catalyst in the Michael addition of *n*-pentanal (**12b**) to β-nitrostyrene (**13**), under the conditions reported by Ma in 2008, using benzoic acid as a cocatalyst and water as the solvent (Table 3).^19^ Under these conditions, all catalysts (*S*)-**8b**–**d** (Entries 2–4, Table 3) were less reactive than the Hayashi catalyst (*S*)-**9a**, which afforded complete conversion to product **14b** after 6 h at room temperature, with complete enantioselectivity (*ee* > 99%) and a 98:2 diastereomeric ratio (Entry 1, Table 3). Among the (*S*)-**8** series, (*S*)-**8b** was the most reactive, closely followed by (*S*)-**8d**. Unlike the reactions conducted in organic solvents (Table 2), all (*S*)-**8** catalysts displayed excellent enantioselectivity under aqueous conditions, with (*S*)-**8b** and (*S*)-**8d** being the most efficient (Entries 2 and 4, Table 3). The diastereomeric ratios were also high, though consistently slightly lower than those obtained with (*S*)-**9a**.

**Table 3:**
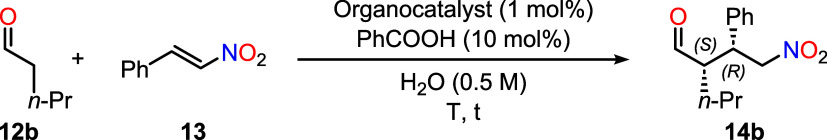
Benchmark Organocatalyzed Michael Addition of *n*-Pentanal **12b** to β-Nitrostyrene **13**.^a^

entry	organocatalyst (*ee*)	time (h)	*T* (°C)	conversion (%)^b^	**14b***d. r.* (*syn*:*anti*)^c^	**14b***ee* (%)^d^
1	(*S*)-**9a** (>99%)	6	rt	>99	98:2	>99
2	(*S*)-**8b** (>99%)	6	rt	78	93:7	>99
3	(*S*)-**8c** (94%)	6	rt	50	92:9	93 (99)^e^
4	(*S*)-**8d** (95%)	6	rt	71	91:9	95 (>99)^e^
5	(*S*)-**9a** (>99%)	6	0	83	98:2	>99
6	(*S*)-**8b** (>99%)	6	0	60	92:8	>99

a **13** (0.3 mmol), H_2_O (0.6 mL), **12b** (2 equiv).

b Determined by ^1^H NMR analysis of the crude reaction mixture, by integrating the signals of **13** and **14b**.

c Determined by ^1^H NMR analysis of the crude reaction mixture, by integrating the signals of the formyl group of the diastereomeric products.

d Determined by chiral HPLC analysis (IC column: flow rate 0.8 mL/min, *n*-hexane:isopropanol 9:1; see Supporting Information).

e Values in parentheses are the final enantiomeric excess normalized considering the enantioenrichment of the used organocatalyst.

Additionally, (*S*)-**8b** was reacted at 0 °C for 6 h and proved less reactive than the Hayashi catalyst also under these conditions (Entries 5–6, Table 3). However, its stereochemical outcomes were nearly identical to those obtained at room temperature.

## Mechanistic Studies

In 2008, Seebach conducted a detailed X-ray structural study on the reactive intermediates formed in organocatalytic transformations involving the catalyst (*S*)-**9a**.^20^ Among the various substrates examined, 2-phenylethanal (phenylacetaldehyde, **15**) was reacted with α,α-diphenyl-2-pyrrolidine methanol trimethylsilyl ether (*S*)-**9a**, and the corresponding enamine (*S*)-**16a** (Figure 4) was isolated and fully characterized. A year later, Seebach and Uchimaru expanded the scope of their study to include additional reactive intermediates, and their findings were further supported by DFT calculations, which confirmed the experimentally determined structures.^21^ Crystal structure analysis and DFT calculations revealed that enamine (*S*)-**16a** predominantly adopts the *sc*-*exo* conformation (*synclinal exocyclic*). In this conformation, one of the phenyl groups of the diphenylmethanol substituent, and especially one of the methyl groups of Me_3_Si, cause a significant steric shielding over one face of the enamine double bond, leaving the opposite face exposed to electrophilic attacks (Figure 4). To gain further insight into the structural parameters influencing the stereochemical outcomes of our proposed methylene isosteres **8**, we prepared the corresponding enamines (*S*)-**16b**–**d** (Figure 4) and studied their conformations in solution by 2D-NMR. Our experimental observations were further reinforced by a series of DFT calculations (see Supporting Information).

**Figure 4:**
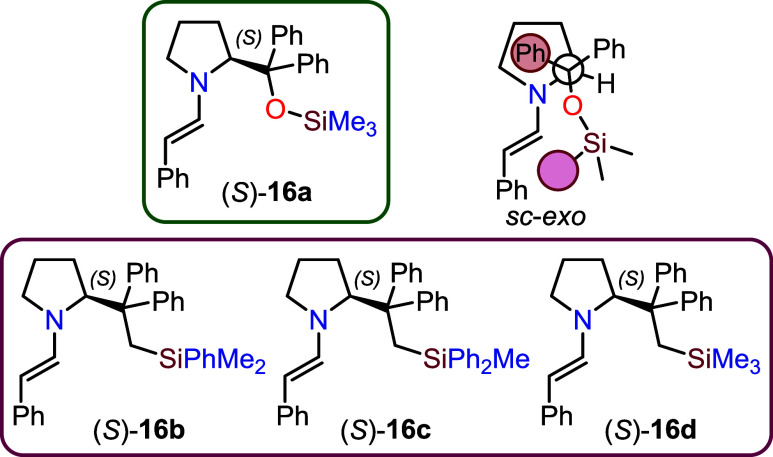
Structures of enamines **16a**–**d** derived from the reaction of 2-phenylethanal (**15**) with Hayashi catalyst (*S*)-**9a** or methylene isosteres (*S*)-**8b**–**d**.

Enamines (*S*)-**16b**–**d** were prepared by reacting equimolar amounts of (*S*)-**8b**–**d** with **15** directly in a 5 mm NMR tube, using CDCl_3_ as the solvent, and were fully characterized by NMR spectroscopy (see Supporting Information). 2D-NMR NOESY spectra clearly evidenced that both (*S*)-**16b** and (*S*)-**16c** predominantly adopt an *ap* conformation (*antiperiplanar*). The most relevant ^1^H NMR chemical shifts for (*S*)-**16b**, determined through COSY and HSQC analyses, are reported in Figure 5A, along with the relevant NOE interactions identified by NOESY. Notably, a moderate NOE signal was observed between one of the hydrogens at C3 and one of the CH_2_Si protons (green arrow), supporting the *ap* conformation in solution (Figure 5C). Furthermore, DFT conformational analysis at the B3LYP/6–31g­(d), using a simplified 2-propanal enamine model, confirmed that the *ap* conformation is by far the most stable (Figure 5B). Similar results were obtained also for enamine (*S*)-**16c** (see Supporting Information).

**Figure 5:**
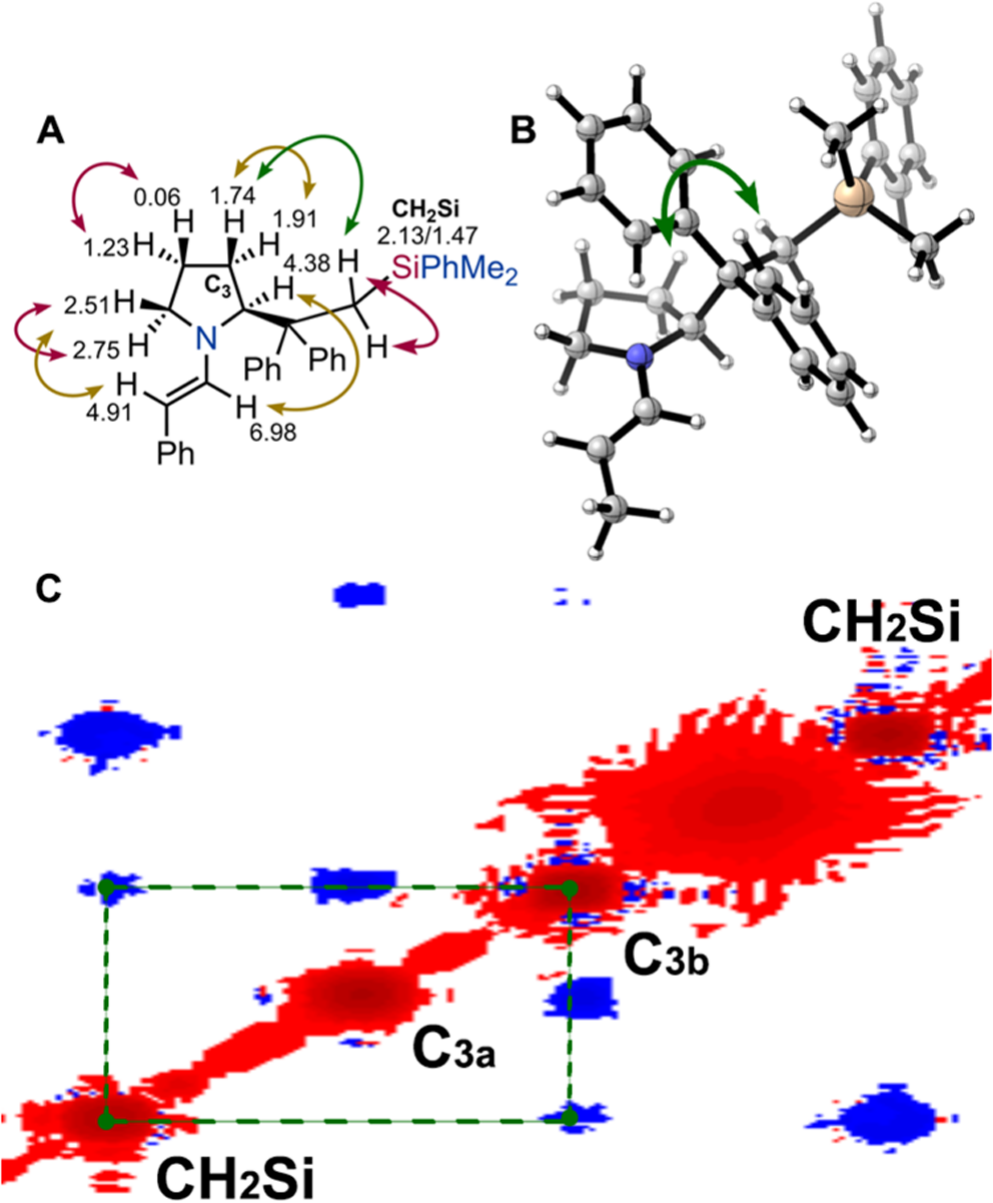
(A) ^1^H NMR chemical shifts of the relevant protons in (*S*)-**16b**, and most important NOESY responses (violet strong, brown and green medium). (B) Structure of the most stable 2-propanal derived enamine optimized at the B3LYP/6–31g­(d) in the gas phase. (C) Expansion of the NOESY spectrum of (*S*)-**16b**, evidencing the response between CH_2_Si and one of the protons at C3.

Conversely, the same analysis on (*S*)-**16d** (Figure 6), revealed that this enamine primarily adopts the *sc-exo* conformation in solution. This conclusion was supported by NOESY interactions between one of the CH_2_Si protons and the proton at the C2 stereocenter (green arrow, Figure 6A), as well as by the absence of NOE responses with aliphatic protons on the pyrrolidine ring, in contrast to the two previous cases. Once again, DFT conformational analysis confirmed this result, showing that the *sc-exo* conformation is the most stable for the corresponding 2-propanal-derived enamine (Figure 6B).

**Figure 6:**
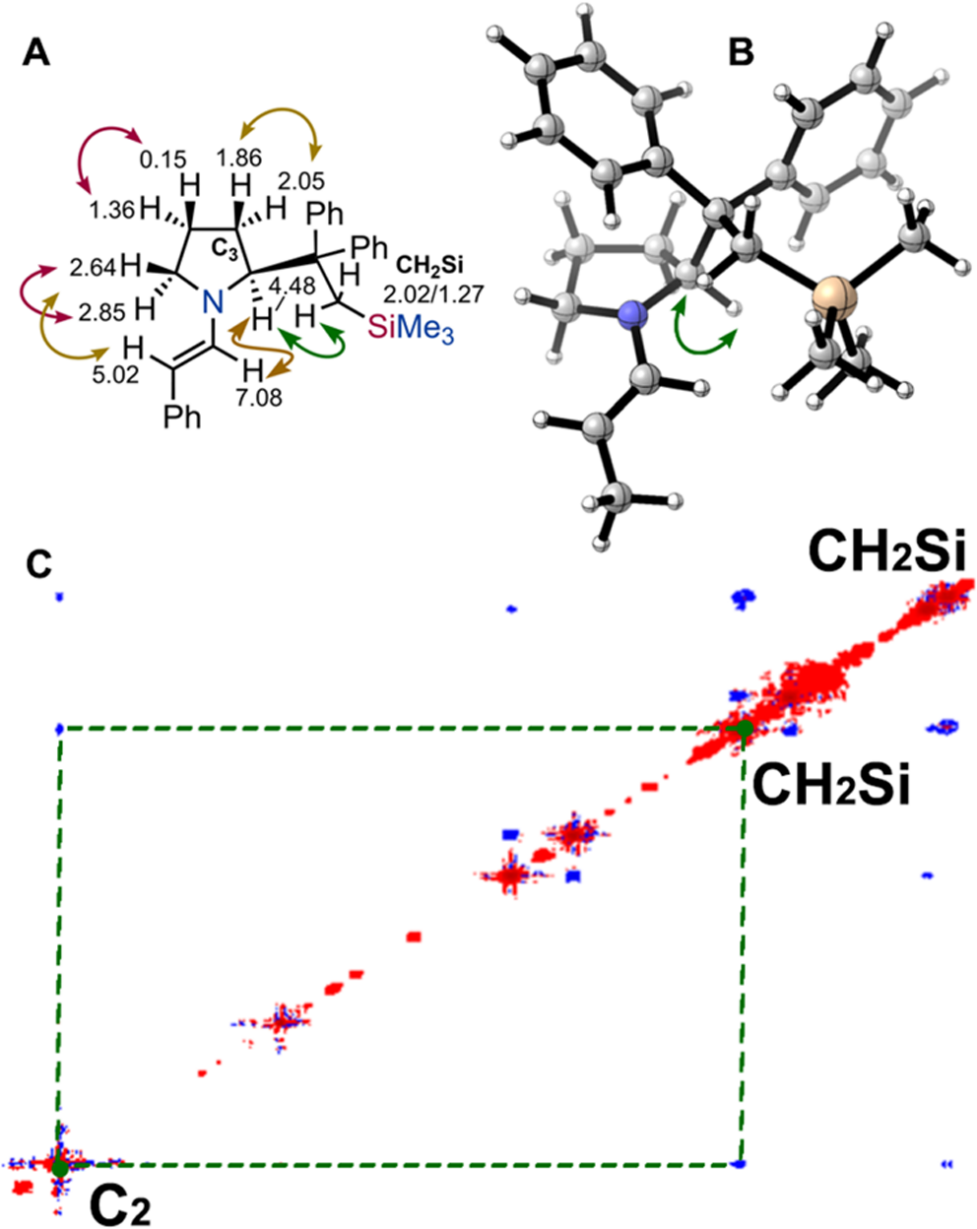
(A) ^1^H NMR chemical shifts of the relevant protons in (*S*)-**16d**, and most important NOESY responses (violet strong, brown and green medium). (B) Structure of the most stable 2-propanal derived enamine optimized at the B3LYP/6–31g­(d) in the gas phase. (C) Expansion of the NOESY spectrum of (*S*)-**16d**, evidencing the response between CH_2_Si and the proton at C2 stereocenter.

The superior stereochemical performance of catalyst (*S*)-**8d** compared to its congeners (*S*)-**8b** and (*S*)-**8c** appears to be strictly related to the most populated conformer of the corresponding enamine **16** in solution. The enamine (*S*)-**16d** predominantly adopts a *sc-exo* conformation, similarly to (*S*)-**16a**, deriving from Hayashi catalyst. In this conformation, it effectively shields one face of the enamine using a combination of the phenyl rings from the diphenylmethyl substituent, one of the methyl groups from the Me_3_Si group, and one of the hydrogen atoms of the CH_2_ spacer. This relevant shielding results in exceptionally high stereoselectivities in reaction with electrophiles. In contrast, enamines (*S*)-**16b** and (*S*)-**16c** primarily adopt an *ap* conformation, in which only the phenyl rings contribute to shield one face of the enamine, resulting in slightly lower stereoselectivities.

## Conclusions

In conclusion, we have developed a novel and efficient synthetic route to enantioenriched methylene isosteres of Hayashi–Jørgensen catalysts, achieving high yields and excellent diastereoselectivity. The new pyrrolidine-based organocatalysts demonstrated a comparable stereoselectivity in benchmark Michael additions, with the trimethylsilyl derivative (*S*)-**8d** exhibiting almost complete enantioselectivity (up to 99% *ee*). Structural investigations using 2D-NMR and DFT calculations provided key insights into the conformational preferences of the corresponding enamines, offering a rationale for the observed catalytic performances. These findings highlight the potential of silyl-modified pyrrolidines as effective chiral organocatalysts and pave the way for further exploration of their applications in asymmetric synthesis. In particular, these derivatives are expected to be stable under hydrolytic conditions, whereas Hayashi–Jørgensen catalysts are known to undergo slow desilylation, followed by rapid oxazolidine formation in DMSO.^22^ Furthermore, the Hayashi–Jørgensen catalyst has been reported to decompose during the α-bromination of aldehydes with NBS, via a Grob-type fragmentation pathway.^23^ Experiments to evaluate the stability of our newly proposed organocatalysts are currently in progress and will be reported in due course.

## Experimental Section

### General Information

All the commercial chemicals were used without additional purification unless otherwise stated. The ^1^H and ^13^C­{^1^H} spectra were recorded on a Varian INOVA 400, a Varian INOVA 600 or a Bruker Ascend-600 instrument with a 5 mm probe. All chemical shifts have been quoted relative to residue solvent signal; chemical shifts (δ) are reported in ppm and coupling constants (*J*) are reported in hertz (Hz). Structural assignments were made with additional information from gCOSY and gHSQC, experiments. Low-resolution MS (LRMS) ESI analyses were performed on an Agilent Technologies MSD1100 single quadrupole mass spectrometer. High-resolution MS (HRMS) ESI analyses were performed on a Xevo G2-XS QTof (Waters) mass spectrometer. HPLC analyses were performed on an Agilent Technologies HP1260 instrument. Melting point (m.p.) measurements were performed on Bibby Stuart Scientific SMP3 apparatus. Optical rotation measurements ([α]_D_^20^) were performed on a polarimeter Schmidt+Haensch UniPol L1000. Flash chromatography purifications were carried out using VWR silica gel (40–63 μm particle size). Thin-layer chromatography was performed on Merck 60 F254 plates. The diastereoisomeric ratios of products **10** and **11** were determined by HPLC-MS analysis by comparing the area of the peaks of the two diastereoisomers.

### General Procedure A: Synthesis of Silicon-Substituted Heterocycles **7b**–**c**, **10**, **11**

A two-neck round-bottom flask was dried under *vacuum* and then refilled with Argon. To this, metallic lithium (9 mmol, 6 equiv) and 2 mL of dry THF were added. Few drops of trimethylsilyl chloride (TMSCl) were then added to wash the metallic lithium and the mixture was left stirring at room temperature until the color of Li turned from black to gray. The solvent was removed with a glass syringe and the lithium was washed with dry THF (2 × 2 mL). Once a clean lithium was obtained, THF (2 mL, 0.5 M with respect to the chlorosilane) was added, and the heterogeneous mixture was cooled to 0 °C. 1.5 mmol (1.5 equiv) of the corresponding chlorosilane was then added and the reaction mixture was left stirring while reaching room temperature over a period of 4 h. Within the first 5 min of stirring, the characteristic deep red color of silyl-lithium **1** appears, indicating the beginning of the reaction. After the reported time, a three-neck round-bottom flask equipped with a dropping funnel was dried under *vacuum* and refilled with argon. To it, a solution of diphenylethylene **2a** (1 mmol, 1 equiv) in 4 mL (0.25 M) of diethyl ether was added. The silyl lithium **1** was then transferred to the dropping funnel and added dropwise to the alkene at 0 °C. After 1 h, the addition of the silyl-lithium to the alkene was checked by ^1^H NMR analysis and the conversion was calculated by comparing the signals of the starting alkene with those of the formed organosilane **3**. At this point the solution was cooled to −78 °C and 0.9 mmol (0.9 equiv) of imine **4a**–**c** in 2 mL (0.45 M with respect to the imine) of THF:Et_2_O (1:2) were added dropwise. The reaction mixture was stirred at the same temperature for 2 h and then allowed to reach room temperature while stirring overnight. The mixture was quenched with saturated aqueous ammonium chloride (5 mL) and extracted with diethyl ether (3 × 5 mL). The combined organic layers were dried over sodium sulfate and the solvent was removed under reduced pressure. The crude product was purified with flash chromatography on silica gel (90:10 CyH:EtOAc).

### (*S*)-1-((*R*)-*tert*-Butylsulfinyl)-2-(2-(dimethyl­(phenyl)­silyl)-1,1-diphenylethyl)­pyrrolidine (**7b**)

Product **7b** was obtained as a white solid in 66% isolated yield (258 mg, 0.53 mmol) after purification with flash column chromatography (CyH:EtOAc = 90:10), starting from (*R*)-**4a** (203 mg, 0.8 mmol) and following general procedure **A**. *dr* > 99:1; mp 62–64 °C; [α]_D_^20^: −73.4 (c = 1.4, CHCl_3_); ^1^H NMR (600 MHz, CDCl_3_) δ 7.37–7.31 (m, 6H), 7.30–7.26 (m, 3H), 7.25–7.17 (m, 6H), 4.75 (dd, *J* = 9.0, 2.8, 1H), 3.40 (ddd, *J* = 10.2, 8.5, 7.0, 1H), 2.13 (d, *J* = 14.7, 1H) 2.02–1.97 (m, 1H), 1.95–1.91 (m, 1H), 1.77 (bs, 1H), 1.54 (d, *J* = 14.7, 1H), 1.43–1.37 (m, 1H), 1.04 (s, 9H), 0.40–0.33 (m, 1H), 0.10 (s, 3H), −0.40 (s, 3H); ^13^C­{^1^H} NMR (CDCl_3_, 150 MHz) δ 146.0, 140.8, 133.5, 130.2, 128.7, 127.7, 127.6, 127.4, 126.6, 126.4, 75.2, 58.8, 54.9, 43.3, 30.6, 28.6, 26.0, 24.6, −1.4, −1.7; LRMS (ESI) *m*/*z*: 512.1 [M + Na]^+^, 528.2 [M+K]^+^, 1001.1 [2M+Na]^+^; HRMS (ESI) *m*/z: [M + Na]^+^ calcd. for C_30_H_39_NNaOSSi 512.2414; found. 512.2417.

### (*S*)-1-((*R*)-*tert*-Butylsulfinyl)-2-(2-(methyldiphenylsilyl)-1,1-diphenylethyl)­pyrrolidine (**7c**)

Product **7c** was obtained as colorless wax in 55% isolated yield (241 mg, 0.44 mmol) after purification with flash column chromatography (CyH:EtOAc = 90:10), starting from (*R*)-**4a** (200 mg, 0.79 mmol) and following general procedure **A**.·*dr =* 97:3; [α]_D_^20^: −40.8 (c = 1.1, CHCl_3_); ^1^H NMR (600 MHz, CDCl_3_. peaks of the major isomer) δ 7.43–7.39 (m, 2H), 7.31–7.25 (m, 7H), 7.23–7.20 (m, 1H), 7.15–7.10 (m, 7H), 7.09–7.04 (m, 3H), 4.76 (dd, *J* = 9.0, 2.4 Hz, 1H), 3.39 (ddd, *J* = 10.2, 8.5, 7.2 Hz, 1H), 2.43 (d, *J* = 14.8 Hz, 1H), 2.07 (d, *J* = 14.9 Hz, 1H), 2.02–1.94 (m, 2H), 1.79–1.69 (m, 2H), 1.43–1.36 (m, 1H), 1.01 (s, 9H), 0.38–0.31 (m, 1H), 0.30 (s, 3H); ^13^C­{^1^H} NMR (150 MHz, CDCl_3_, peaks of the major isomer) δ 145.5, 138.5, 137.9, 134.5, 134.1, 130.2, 128.9, 128.6, 127.7, 127.4, 127.3, 127.2, 126.5, 126.4, 75.3, 58.7, 54.8, 43.3, 29.0, 28.8, 25.9, 24.5,–3.4. LRMS (ESI) *m*/*z*: 574.2 [M + Na]^+^, 1126.2 [2M+Na]^+^; HRMS (ESI) *m*/z: [M + Na]^+^ calcd. for C_35_H_41_NNaOSSi 574.8532; found. 574.8534.

### (*S*)-1-((*R*)-*tert*-Butylsulfinyl)-2-(2-(dimethyl­(phenyl)­silyl)-1,1-diphenylethyl)­azetidine (**10**)

Product **10** was obtained as a colorless oil in 25% isolated yield (43 mg, 0.09 mmol) after purification with flash column chromatography (CyH:EtOAc = 90:10), starting from (*R*)-**4a** (87 mg, 0.36 mmol) and following general procedure **A**. *d.r:* 99:1; [α]_D_^20^:–67.6 (c = 0.7 CHCl_3_); ^1^H NMR (600 MHz, CDCl_3_) δ 7.37–7.35 (m, 3H), 7.30–7.28 (m, 3H), 7.24–7.20 (m, 7H), 7.09–7.07 (m, 2H), 4.98 (dd, *J* = 9.2, 6.1 Hz, 1H), 4.02 (ddd, *J* = 10.4, 8.3, 6.4 Hz, 1H), 2.45–2.39 (m, 1H), 2.35–2.30 (m, 1H), 2.06 (d, *J* = 14.7 Hz, 1H), 1.94–1.88 (m, 1H), 1.60 (d, *J* = 14.7 Hz, 1H), 1.10 (s, 9H), 0.17 (s, 3H), −0.24 (s, 3H); ^13^C­{^1^H} NMR (150 MHz, CDCl_3_) δ 146.2, 144.9, 140.4, 133.6, 130.2, 129.1, 128.8, 127.9, 127.7, 127.0, 126.6, 126.3, 68.5, 57.9, 53.3, 39.3, 27.4, 23.9, 22.5, −0.9, −1.8; LRMS (ESI) *m*/*z*: 498.2 [M + Na]^+^. HRMS (ESI) *m*/z: [M + Na]^+^ calcd. for C_29_H_37_NNaOSSi 498.2257; found. 498.2260.

### (*S*)-1-((*R*)-*tert*-Butylsulfinyl)-2-(2-(dimethyl­(phenyl)­silyl)-1,1-diphenylethyl)­piperidine (**11**)

Product **11** was obtained as a colorless oil in 69% isolated yield (223 mg, 0.44 mmol) after purification with flash column chromatography (CyH:EtOAc = 90:10), starting from (*R*)-**4a** (172 mg, 0.64 mmol) and following general procedure **A**. *d.r:* 94:6 (determined by ^1^H NMR of the crude reaction mixture); [α]_D_^20^: −46.1 (c = 0.9, CHCl_3_); ^1^H NMR (600 MHz, CDCl_3_, peaks of the major isomer) δ 7.43–7.38 (m, 4H), 7.35–7.30 (m, 2H), 7.29–7.16 (m, 9H), 4.44 (t, *J* = 8.2 Hz, 1H), 2.97–2.82 (m, 1H), 2.02–1.94 (m, 1H), 1.74 (s, 2H), 1.70–1.61 (m, 2H), 1.53–1.45 (m, 1H), 1.44–1.36 (m, 1H), 1.36–1.26 (m, 1H), 1.04 (s, 9H), 0.89–0.76 (m, 1H), −0.20 (2, 3H), −0.37 (s, 3H);^13^C­{^1^H} NMR (150 MHz, CDCl_3_, peaks of the major isomer) δ 143.7, 143.2, 140.7, 133.3, 130.8, 130.4, 128.7, 127.7, 127.5, 127.3, 126.6, 126.5, 69.9, 59.4, 55.6, 41.6, 31.0, 25.3, 23.8, 23.1, 19.0, −1.9, −2.1; LRMS (ESI) *m*/*z*: 526.2 [M + Na]^+^, 542.2 [M+K]^+^; HRMS (ESI) *m*/z: [M + Na]^+^ calcd. for C_31_H_41_NNaOSSi 526.2570; found. 526.2568.

### General Procedure B: Synthesis of Silicon-Substituted Pyrrolidine **7d**

A HMPA (0.5 mL) solution of hexamethyldisilane (1.25 mmol, 1.8 equiv)­under argon atmosphere was cooled until frozen. One mmol (1.4 equiv) of MeLi was added followed by the addition of 2 mL (0.35 M) of THF. The mixture was then allowed to warm to 0 °C and stirred at the same temperature, observing the typical deep red color of the silyl-lithium **1d**. After 10 min, a solution of 0.7 mmol (1 equiv) of **2a** in 1 mL (0.7 M) of THF was added dropwise and the mixture was stirred at 0 °C for 1 h. The addition of the silyl-lithium to the alkene was checked by ^1^H NMR analysis and the conversion was calculated by comparing the signals of the starting alkene with those of the formed organosilane **3d**. At this point the solution was cooled to −78 °C and 0.8 equiv of imine **4a** in 2 mL of THF were added dropwise. The reaction mixture was stirred at the same temperature for 2 h and then allowed to reach room temperature while stirring overnight. The mixture was quenched with saturated aqueous ammonium chloride (5 mL) and extracted with diethyl ether (3 × 5 mL). The combined organic layers were dried over sodium sulfate and the solvent was removed under reduced pressure. The crude product was purified with flash chromatography on silica gel (90:10 CyH:EtOAc).

### (*S*)-1-((*R*)-*tert*-Butylsulfinyl)-2-(1,1-diphenyl-2-(trimethylsilyl)­ethyl)­pyrrolidine (**7d**)

Product **7d** was obtained as white wax in 74% isolated yield (528 mg, 1.23 mmol) after purification with flash column chromatography (CyH:EtOAc = 90:10), starting from (*R*)-**4a** (422 mg, 1.66 mmol) and following general procedure **B**. *er =* 97.5:2.5; [α]_D_^20^: −87.6 (c = 1.4, CHCl_3_); ^1^H NMR (600 MHz, CDCl_3_) δ (ppm): ^1^H NMR (600 MHz, CDCl_3_) δ 7.36–7.32 (m, 4H), 7.28 (t, *J* = 8.0, 2H), 7.23–7.16 (m, 4H), 4.73 (dd, *J* = 9.1, 3.0, 1H), 3.40 (ddd, *J* = 10.1, 8.5, 6.8, 1H), 2.04–1.97 (m, 1H), 1.93–1.89 (m, 2H), 1.79–1.70 (m, 1H), 1.43–1.46 (m, 1H), 1.25 (d, *J* = 14.5 Hz, 1H), 1.10 (s, 9H), 0.43–0.36 (m, 1H), 0.39 (s, 9H). ^13^C­{^1^H} (150 MHz, CDCl_3_) δ 146.1, 130.2, 127.5, 127.4, 126.5, 126.3, 75.6, 58.7, 55.0, 43.4, 31.0, 28.6, 26.1, 24.6, 0.2; LRMS (ESI) *m*/*z*: 428.1 [M + H]^+^, 450.2 [M + Na]^+^, 877.2 [2M+Na]^+^; HRMS (ESI) *m*/z: [M + Na]^+^ calcd. for C_25_H_37_NNaOSSi 450.2257; found. 450.2255.

### General Procedure C: Removal of the Sulfinyl Group

Acetyl chloride (3 mmol, 3 equiv) was added at 0 °C to a solution of **7** (1 mmol, 1 equiv) in MeOH (2 mL, 0.5 M). The reaction was stirred at room temperature for 1h (monitored by TLC), then quenched with saturated sodium bicarbonate (5 mL) and extracted with ethyl acetate (3 × 5 mL). The combined organic layers were dried over sodium sulfate and the solvent was removed under reduced pressure. The crude product was purified with flash chromatography (95:5 DCM/MeOH).

### (*S*)-2-(2-(Dimethyl­(phenyl)­silyl)-1,1-diphenylethyl)­pyrrolidine (**8b**)

Product **8b** was obtained as a white solid in 95% isolated yield (194 mg, 0.5 mmol) after purification with flash column chromatography (DCM/MeOH = 95:5), starting from **7b** (258 mg, 0.53 mmol) and following general procedure **C**. m.p.: 112–114 °C; [α]_D_^20^: + 5.2 (c: 1.3, CHCl_3_). The enantiomeric excess was determined to be >99% by HPLC analysis on a Daicel Chiralpak IC column: 90:10 hexane/IPA, flow rate: 0.8 L/min, λ: 254 nm, τ_major_: 6.17 min, τ_minor_: 7.53 min; ^1^H NMR (600 MHz, CDCl_3_) δ 7.46–7.44 (m, 2H), 7.36–7.32 (m, 5H), 7.28–7.25 (m, 6H), 7.23–7.18 (m, 2H), 3.78 (t, *J* = 7.7 Hz, 1H), 2.64–2.60 (m, 1H), 2.45–2.41 (m, 1H), 1.90 (d, *J* = 14.4 Hz, 1H), 1.86 (d, *J* = 14.4 Hz, 1H), 1.77–1.71 (m, 1H), 1.44–1.37 (m, 1H), 1.30–1.24 (m, 1H), 1.13–1.06 (m, 1H), −0.08 (s, 3H), −0.18 (s, 3H); ^13^C­{^1^H} NMR (150 MHz, CDCl_3_) δ 147.9, 146.7, 140.6, 133.6, 129.8, 129.5, 128.8, 127.8, 127.7, 127.5, 126.2, 126.1, 63.4, 53.4, 46.7, 30.5, 28.0, 25.2, −1.8, −2.0; LRMS (ESI) *m*/*z*: 386.3 [M + H]^+^. HRMS (ESI) *m*/z: [M + H]^+^ calcd. for C_26_H_32_NSi 386.2299; found. 386.2301.

### (*S*)-2-(2-(Methyldiphenylsilyl)-1,1-diphenylethyl)­pyrrolidine (**8c**)

Product **8c** was obtained as a white solid in 88% isolated yield (172 mg, 0.38 mmol) after purification with flash column chromatography (DCM/MeOH = 95:5), starting from **7c** (241 mg, 0.44 mmol) and following general procedure **C**. m.p.: 112–114 °C; [α]_D_^20^: + 4.9 (c = 0.5, CHCl_3_). The enantiomeric excess was determined to be 94% by HPLC analysis on a Daicel Chiralpak IC column: 90:10 hexane/IPA, flow rate = 0.8 mL/min, λ = 254 nm, τ_major_ = 6.42 min, τ_minor_ = 7.69 min; ^1^H NMR (600 MHz, CDCl_3_) δ 7.47–7.42 (m, 4H), 7.34–7.27 (m, 7H), 7.28–7.23 (m, 5H), 7.22–7.18 (m, 3H), 7.16–7.13 (m, 1H), 3.60 (t, *J* = 7.4 Hz, 1H), 2.50–2.46 (m, 1H), 2.34–2.30 (m, 2H), 2.21 (d, *J* = 14.3 Hz, 1H), 1.64–1.58 (m, 1H), 1.28–1.19 (m, 2H), 1.01–0.94 (m, 1H), −0.17 (s, 3H); ^13^C­{^1^H} NMR (150 MHz, CDCl_3_) δ 147.8, 146.9, 138.6, 138.5, 134.7, 134.5, 129.7, 129.6, 129.1, 129.0, 127.8, 127.8, 127.7, 127.6, 126.2, 126.1, 63.0, 53.3, 46.5, 27.9, 25.1, −4.0; LRMS (ESI) *m*/*z*: [M + H]^+^. HRMS (ESI) *m*/z: [M + H]^+^ calcd. for C_31_H_34_NSi^+^ 448.2455; found. 448.2458.

### (*S*)-2-(1,1-Diphenyl-2-(trimethylsilyl)­ethyl)­pyrrolidine (**8d**)

Product **8d** was obtained as a yellow wax in 90% isolated yield (293 mg, 0.91 mmol) after purification with flash column chromatography (DCM/MeOH = 95:5), starting from **7d** (428 mg, 1.0 mmol) and following general procedure **C**. [α]_D_^20^: + 8.7 (c = 1.1, CHCl_3_). The enantiomeric excess was determined to be 95% by HPLC analysis on a Daicel Chiralpak IC column: 90:10 hexane/IPA, flow rate = 0.8 mL/min, λ = 254 nm, τ_major_ = 5.75 min, τ_minor_ = 7.26 min; ^1^H NMR (600 MHz, CDCl_3_) δ 7.35–7.32 (m, 2H), 7.29–7.24 (m, 6H), 7.22–7.16 (m, 2H), 3.98 (t, *J* = 7.8 Hz, 1H), 2.77–2.73 (m, 1H), 2.51–2.48 (m, 1H), 1.90–1.84 (m, 1H), 1.62 (d, *J* = 3.3 Hz, 2H), 1.59–1.54 (m, 1H), 1.37–1.31 (m, 1H), 1.21–1.15 (m, 1H), −0.33 (s, 9H); ^13^C­{^1^H} NMR (150 MHz, CDCl_3_) δ 129.8, 129.4, 127.8, 127.4, 126.1, 126.0, 64.0, 46.9, 31.0, 30.9, 28.0, 25.3, 0.10. LRMS (ESI) *m*/*z*: 324.2 [M + H]^+^. HRMS (ESI) *m*/z: [M + H]^+^ calcd. for C_21_H_30_NSi 324.2142; found. 324.2145.

### General Procedure E: Organocatalysis under Hayashi’s Conditions

To a nitrostyrene **13** (0.1 mmol, 1 equiv) and aminocatalyst **8** (0.005 mmol, 5 mol %) solution in *n*-hexane (0.1 mL, 1 M), 0.15 mmol (1.5 equiv) of propanal **12a** were added. After stirring for 3 h, the reaction was quenched with saturated ammonium chloride (4 mL) and extracted with EtOAc (3 × 3 mL). The combined organic layers were dried over sodium sulfate and the solvent was removed under reduced pressure. The crude product, without any other purification, was dissolved in a solution of *n*-hexane and isopropanol (1:1) and injected into chiral stationary phase HPLC.

## Supplementary Material



## Data Availability

The data underlying this study are available in the published article and its Supporting Information.

## Notes

Detailed experimental procedures, spectroscopic and analytical data, copies of ^1^H and ^13^C NMR spectra for all new compounds, mechanistic study details, and DFT coordinates for all optimized structures (PDF)
