# Supplementary material for: Diastereoselective Synthesis of Silyl-Substituted Pyrrolidines
Source: J Org Chem. 2025 Jun 21;90(26):9160–9. doi: 10.1021/acs.joc.5c01023 (PMC12235646; doi:10.1021/acs.joc.5c01023)
Supplement: Supplementary file 1 [file jo5c01023_si_001.pdf]

Supporting Information

Diastereoselective Synthesis of Silyl Substituted Pyrrolidines

Davide Carboni,[a,b] Giulio Casagranda,[a,b] Simone Di Remigio,[a,b] Alice Mirone,[a] Arianna Quintavalla,[a,b,*] and Marco Lombardo[a,b,*]

[a]Department of Chemistry "G. Ciamician", Alma Mater Studiorum - University of Bologna, via P. Gobetti, 85 – 40129, Bologna, Italy

[b]Center for Chemical Catalysis - C3, Alma Mater Studiorum - University of Bologna, via P. Gobetti, 85 – 40129, Bologna, Italy

E-mail: arianna.quintavalla@unibo.it; marco.lombardo@unibo.it

Table of contents

1 General information **S1**

1.1 General methods S1

1.2 Preparation of known starting materials S2

2 Conditions optimization for the synthesis of 7d **S3**

3 Experimental procedures **S4**

3.1 General procedure **A**: Synthesis of silicon substituted pyrrolidines **7b-7c** (Table 1) S4

3.2 General procedure **B**: Synthesis of silicon substituted pyrrolidine **7d** (Scheme 3) S4

3.3 General procedure **C**: Removal of the sulfinyl group (Scheme 4) S5

3.4 General procedure **D**: Benzoylation reaction S5

3.5 General procedure **E**: Organocatalysis under Hayashi's conditions (Table 2) S5

3.6 General procedure **F**: Organocatalysis under Ma's conditions (Table 3) S5

4 Characterization data of products 7b-d, 10 and 11 .... **S6**

5 Characterization data of products 8b-d **S8**

6 Determination of the stereochemistry of products 8b-d **S10**

7 Mechanistic studies **S11**

7.1 General procedure for enamine preparation S11

7.2 Characterization of enamine **16a** S11

7.3 Characterization of enamine **16b** S16

7.4 Characterization of enamine **16c** S22

7.5 Characterization of enamine **16d** S26

7.6 **DFT Calculations** S30

8 Copies of ^{1}H and $^{13}C\{^{1}H\}$ NMR spectra **S148**

9 Copies of HPLC-MS spectra of products 10 and 11 .... **S157**

10 Chiral HPLC traces **S158**

10.1 Chiral HPLC traces of benzoylated **8b-8d** S158

10.2 Chiral HPLC traces of product **14a** S162

10.3 Chiral HPLC traces of product **14b** S164

10.4 Chiral HPLC traces of imine **4a** S166

11 Bibliography **S167**

1 General information

1.1 General methods

The ^{1}**H** and ^{13}**C{**^{1}**H} NMR** spectra were recorded on a Varian INOVA 400, or a Varian INOVA 600 or a Bruker Ascend-600 instrument with a 5 mm probe. The spectra were recorded at 400 MHz or 600 MHz for ^{1}H and at 100 MHz or 150 MHz for ^{13}C{^{1}H}, respectively. All chemical shifts have been quoted relative to residue solvent signal; chemical shifts (δ) are reported in ppm and coupling constants (J) are reported in hertz (Hz). The following abbreviations are used to indicate the multiplicity: s (singlet), d (doublet), t (triplet), q (quartet), p (pentet), hept (heptet), m (multiplet), br (broad), app (apparent). Structural assignments were made with additional information from gCOSY and gHSQC, experiments. Low-resolution MS (**LRMS**) ESI analyses were performed on an Agilent Technologies MSD1100 single- quadrupole mass spectrometer. Mass spectrometric detection was performed in the full-scan mode from *m/z* 50 to 2500, with a scan time of 0.1 s in the positive ion mode, ESI spray voltage of 4500 V, nitrogen gas pressure of 35 psi, drying gas flow rate of 11.5 mL min^{-1} and fragmentor voltage of 30 V. High-resolution MS (**HRMS**) ESI analyses were performed on a Xevo G2-XS QTof (Waters) mass spectrometer. Mass spectrometric detection was performed in the full-scan mode from *m/z* 50 to 1200, with a scan time of 0.15 s in the positive ion mode, cone voltage: 40 V, collision energy: 6.00 eV. ESI: capillary: 3kV, cone: 40 V, source temperature: 120 °C, desolvation temperature: 600 °C, cone gas flow: 50 L/h, desolvation gas flow: 1000 L/h. **HPLC** analyses were performed on an Agilent Technologies HP1260 instrument. A Phenomenex Gemini C18 3 µm (100 x 3 mm) column was employed for the chromatographic separation: mobile phase H_2O/CH_3CN, gradient from 30% to 80% of CH_3CN in 8 min, 80% of CH_3CN until 22 min, then up to 90% of CH_3CN in 2 min, flow rate 0.4 mL/min. Melting point (**m.p.**) measurements were performed on Bibby Stuart Scientific SMP3 apparatus. Optical rotation measurements ($[\alpha]_D^{20}$) were performed on a polarimeter Schmidt+Haensch UniPol L1000. **Flash chromatography** purifications were carried out using VWR silica gel (40 – 63 µm particle size). **Thin-layer chromatography** was performed on Merck 60 F254 plates. The diastereoisomeric ratios of products **10** and **11** were determined by HPLC-MS analysis by comparing the area of the peaks of the two diastereoisomers.

Materials. All the commercial chemicals were purchased from Sigma-Aldrich, VWR, Alfa Aesar, Fluorochem or TCI Chemicals and used without additional purification unless otherwise stated. The solvents used — tetrahydrofuran (THF), diethyl ether (Et_2O), pentane, and hexamethylphosphoramide (HMPA) — were dried over 3 Å molecular sieves for 72 hours, then distilled and stored over molecular sieves (3 Å) under an inert atmosphere. Diphenylethylene **2a**, Propanal **12a** and Pentanal **12b** were distilled and then stored under inert atmosphere at 4 °C. Phenylacetaldehyde **15** was distilled and then stored under inert atmosphere at -20 °C.

1.2 Preparation of known starting materials

Sulfinimines (*R*)-**4a-c** were prepared following known literature procedures[1] and were stored under argon at 4 °C. Their spectroscopic data matched the reported ones.[1,2] Their stereochemical purity was verified by chiral HPLC analysis before use (See Section 10.4 for more details).

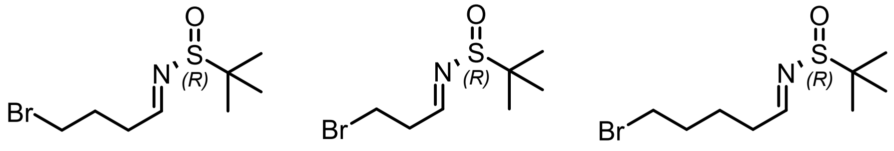

4a **4b** **4c**

Figure S1 - -Sulfinimines *(R)*-**4a-c** prepared according to known literature procedures

2 Conditions optimization for the synthesis of 7d

For the preparation of compound **7d**, we first synthesized trimethylsilyllithium (**1d**) by following the procedure previously reported by Still in 1976[3]. The resulting trimethylsilyllithium (**1d**) was then employed in the one-pot protocol for the preparation of product **7d** (Table S1, Entry 1). Under these conditions, the desired product **7d** was obtained in 74% yield with excellent diastereoselectivity (*d.r.* 97.5:2.5). To further improve the diastereomeric ratio, we explored different solvent systems. First, we tested Et_2O:HMPTA (5:1) mixture. Under these conditions, we observed a slightly lower yield along with a reduced diastereomeric ratio (Table S1, Entry 2). Next, we tried to add one equivalent of LiCl just before the addition of imine **4a**. However, this modification not only led to a significantly lower yield (40%) but also further reduced the diastereomeric ratio (90:10, Table S1, Entry 3).

Table S1 - Optimization of the reaction conditions for the preparation of product **7d**.[a]

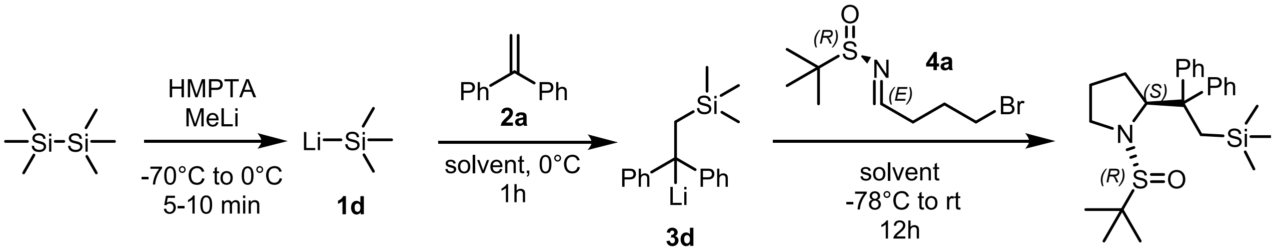

Entry	**4a** *e.e* (%)[b]	Solvent	Time (h)	**7d** Yield (%)[c]	**7d** *d.e.* (%)[d]
1	>99	THF:HMPTA 5:1	12	74	95
2	>99	Et_2O:HMPTA 5:1	12	60	88
3	>99	THF:HMPTA 5:1[e]	12	40	80

[a]**1d** (1.5 equiv., 1 mmol), solvent (2 mL), **2a** (1 equiv.), (*R*)-**4a** (1.1 equiv.). [b]Determined by chiral HPLC analysis (OD-H column: flow rate 0.5 mL/min, *n*-hexane:isopropanol 9:1). [c]Isolated yield after purification by flash-chromatography. [d]The diastereomeric excess was determined after removal of the sulfinyl group and benzoylation reaction, as enantiomeric excess of the derived product. [e]LiCl 1.0 equivalent was added before the addition of sulfinyl imine **4a**.

3 Experimental procedures

3.1 General procedure **A**: Synthesis of silicon substituted pyrrolidines **7b-7c** (Table 1)

A two neck round bottom flask was dried under *vacuum* and then backfilled with Argon. To this, metallic lithium (9 mmol, 9 eq) and 2 mL of dry THF were added. Few drops of trimethyl silyl chloride (TMSCl) were then added to wash the metallic lithium and the mixture was left stirring at room temperature until the color of Li turned from black to gray. The solvent was removed with a glass syringe and the lithium was washed with dry THF (2 x 2 mL). Once a clean lithium was obtained, THF (2 mL, 0.5 M with respect to the chlorosilane) was added, and the heterogeneous mixture was cooled to 0 °C. 1.5 mmol (1.5 eq.) of the corresponding chlorosilane was then added and the reaction mixture was left stirring while reaching room temperature over a period of 4 hours. Within the first five minutes of stirring, the characteristic deep red color of silyl-lithium **1** appears, indicating the beginning of the reaction. After the reported time, a three neck round bottom flask equipped with a dropping funnel was dried under *vacuum* and refilled with argon. To it, a solution of diphenylethylene **2a** (1 mmol, 1 eq.) in 4 mL (0.25 M) of diethyl ether was added. The silyl lithium **1** was then transferred to the dropping funnel and added dropwise to the alkene at 0 °C. After one hour, the addition of the silyl-lithium to the alkene was checked by ^{1}H-NMR analysis and the conversion was calculated by comparing the signals of the starting alkene with those of the formed organosilane **3**. At this point the solution was cooled to -78 °C and 0.9 mmol (0.9 eq.) of imine **4a** in 2 mL (0.45 M with respect to the imine) of THF:Et_2O (1:2) were added dropwise. The reaction mixture was stirred at the same temperature for 2 hours and then allowed to reach room temperature while stirring overnight. The mixture was quenched with saturated aqueous ammonium chloride (5 mL) and extracted with diethyl ether (3x5 mL). The combined organic layers were dried over sodium sulfate and the solvent was removed under reduced pressure. The crude product was purified with flash chromatography on silica gel (90:10 CyH:EtOAc).

3.2 General procedure **B**: Synthesis of silicon substituted pyrrolidine **7d** (Scheme 3)

An HMPA (0.5 mL) solution of hexametil-disilane (1.25 mmol, 1.8 eq) under argon atmosphere was cooled until frozen. 1 mmol (1.4 eq) of MeLi was added followed by the addition of 2 mL (0.35 M) of THF. The mixture was then allowed to warm to 0 °C and stirred at the same temperature, observing the typical deep red color of the silyl-lithium **1d**. After 10 minutes, a solution of 0.7 mmol (1 eq) of **2a** in 1 mL (0.7 M) of THF was added dropwise and the mixture was stirred at 0 °C for one hour. The addition of the silyl-lithium to the alkene was checked by ^{1}H-NMR analysis and the conversion was calculated by comparing the signals of the starting alkene with those of the formed organosilane **3d**. At this point the solution was cooled to -78 °C and 0.8 equivalents of imine **4a** in 2 mL of THF were added dropwise. The reaction mixture was stirred at the same temperature for 2 hours and then allowed to reach room temperature while stirring overnight. The mixture was quenched with saturated aqueous ammonium chloride (5 mL) and extracted with diethyl ether (3x5 mL). The combined organic layers were dried over sodium sulfate and the solvent was removed under reduced pressure. The crude product was purified with flash chromatography on silica gel (90:10 CyH:EtOAc).

3.3 General procedure **C**: Removal of the sulfinyl group (Scheme 4)

Acetyl chloride (3 mmol, 3 eq.) was added at 0 °C to a solution of **7** (1 mmol, 1 eq.) in MeOH (2 mL, 0.5 M). The reaction was stirred at room temperature for 1h (monitored by TLC), then quenched with saturated sodium bicarbonate (5 mL) and extracted with ethyl acetate (3x5 mL). The combined organic layers were dried over sodium sulfate and the solvent was removed under reduced pressure. The crude product was purified with flash chromatography (95:5 DCM:MeOH).

3.4 General procedure **D**: Benzoylation reaction

To a solution of 0.1 mmol (1 eq.) of **8** in 1 mL (0.1 M) of dichloromethane, triethylamine (0.12 mmol, 1.2 eq) and benzoyl chloride (0.11 mmol, 1.1 eq) were added at 0 °C. The reaction was stirred for 1.5 h at room temperature (monitored by TLC), then quenched with saturated ammonium chloride (3 mL) and extracted with dichloromethane (3x3 mL). The combined organic layers were dried over sodium sulfate and the solvent was removed under reduced pressure. The crude product, without any other purification, was dissolved in a solution of hexane and isopropanol (1:1) and injected into chiral HPLC.

3.5 General procedure **E**: Organocatalysis under Hayashi's conditions (Table 2)[4]

To a nitrostyrene **13** (0.1 mmol, 1 eq.) and aminocatalyst **8** (0.005 mmol, 5 mol%) solution in *n*-hexane (0.1 mL, 1 M), 0.15 mmol (1.5 eq.) of propanal **12a** were added. After stirring for 3 h, the reaction was quenched with saturated ammonium chloride (4 mL) and extracted with EtOAc (3x3 mL). The combined organic layers were dried over sodium sulfate and the solvent was removed under reduced pressure. The crude product, without any other purification, was dissolved in a solution of *n*-hexane and isopropanol (1:1) and injected into chiral HPLC.

3.6 General procedure **F**: Organocatalysis under Ma's conditions (Table 3)[5]

To a nitrostyrene **13** (0.3 mmol, 1 eq.), aminocatalyst **8b-d** (0.003 mmol, 10 mol%) and benzoic acid (0.03 mmol, 10 mol%) suspension in water (0.6 mL, 0.5 M), 0.6 mmol (2 eq.) of *n*-pentanal **12b** were added. After stirring for 6 h, the reaction was quenched with saturated ammonium chloride solution (4 mL) and extracted with EtOAc (3x3 mL). The combined organic layers were dried over sodium sulfate and the solvent was removed under reduced pressure. The crude product, without any other purification step, was dissolved in a solution of hexane and isopropanol (1:1) and injected into chiral HPLC.

4 Characterization data of products 7b-d, 10 and 11

(*S*)-1-((*R*)-tert-butylsulfinyl)-2-(2-(dimethyl(phenyl)silyl)-1,1-diphenylethyl)pyrrolidine (7b)

Product **7b** was obtained as a white solid in 66% isolated yield (258 mg, 0.53 mmol) after purification with flash column chromatography (CyH:EtOAc = 90:10), starting from (*R*)-**4a** (203 mg, 0.8 mmol) and following general procedure **A**.

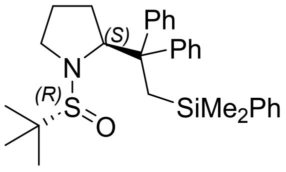

7b

Y% = 66. ***dr*** = >99:1*. **m.p.** = 62-64 °C. $[\alpha]_D^{20}$ = -73.4 (c = 1.4, $CHCl_3$). **^{1}H NMR** (600 MHz, $CDCl_3$) δ 7.37 – 7.31 (m, 6H), 7.30 – 7.26 (m, 3H), 7.25 – 7.17 (m, 6H), 4.75 (dd, J = 9.0, 2.8, 1H), 3.40 (ddd, J =10.2, 8.5, 7.0, 1H), 2.13 (d, J = 14.7, 1H) 2.02 – 1.97 (m, 1H), 1.95 – 1.91 (m, 1H), 1.77 (bs, 1H), 1.54 (d, J = 14.7, 1H), 1.43 – 1.37 (m, 1H), 1.04 (s, 9H), 0.40 – 0.33 (m, 1H), 0.10 (s, 3H), -0.40 (s, 3H). **^{13}C{^{1}H} NMR** ($CDCl_3$, 150 MHz) δ 146.0, 140.8, 133.5, 130.2, 128.7, 127.7, 127.6, 127.4, 126.6, 126.4, 75.2, 58.8, 54.9, 43.3, 30.6, 28.6, 26.0, 24.6, -1.4, -1.7. **LRMS** (ESI) m/z = 512.1 $[M+Na]^+$, 528.2 $[M+K]^+$, 1001.1 $[2M+Na]^+$. **HRMS** *m*/z: $[M + Na]^+$ Calcd. for $[C_{30}H_{39}NNaOSSi]^+$ 512.2414; Found. 512.2417.

(*S*)-1-((*R*)-tert-butylsulfinyl)-2-(2-(methyldiphenylsilyl)-1,1-diphenylethyl)pyrrolidine (7c)

Product **7c** was obtained as colorless wax in 55% isolated yield (241 mg, 0.44 mmol) after purification with flash column chromatography (CyH:EtOAc = 90:10), starting from (*R*)-**4a** (200 mg, 0.79 mmol) and following general procedure **A**.

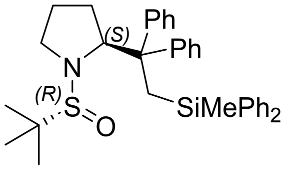

7c

Y% = 55. ***dr*** =97:3*. $[\alpha]_D^{20}$ = -40.8 (c = 1.1, $CHCl_3$). **^{1}H NMR** (600 MHz, $CDCl_3$) δ 7.43 – 7.39 (m, 2H), 7.31 – 7.25 (m, 7H), 7.23 – 7.20 (m, 1H), 7.15 – 7.10 (m, 7H), 7.09 – 7.04 (m, 3H), 4.76 (dd, J = 9.0, 2.4 Hz, 1H), 3.39 (ddd, J = 10.2, 8.5, 7.2 Hz, 1H), 2.43 (d, J = 14.8 Hz, 1H), 2.07 (d, J = 14.9 Hz, 1H), 2.02 – 1.94 (m, 2H), 1.79 – 1.69 (m, 2H), 1.43 – 1.36 (m, 1H), 1.01 (s, 9H), 0.38 – 0.31 (m, 1H), 0.30 (s, 3H). **^{13}C{^{1}H} NMR** (150 MHz, $CDCl_3$) δ 145.5, 138.5, 137.9, 134.5, 134.1, 130.2, 128.9, 128.6, 127.7, 127.4, 127.3, 127.2, 126.5, 126.4, 75.3, 58.7, 54.8, 43.3, 29.0, 28.8, 25.9, 24.5, -3.4. **LRMS** (ESI) *m/z* = 574.2 $[M+Na]^+$, 1126.2 $[2M+Na]^+$. **HRMS** *m*/z: $[M + Na]^+$ Calcd. for $[C_{35}H_{41}NNaOSSi]^+$ 574.8532; Found. 574.8534.

(*S*)-1-((*R*)-tert-butylsulfinyl)-2-(1,1-diphenyl-2-(trimethylsilyl)ethyl)pyrrolidine (7d)

Product **7d** was obtained as white wax in 74% isolated yield (528 mg, 1.23 mmol) after purification with flash column chromatography (CyH:EtOAc = 90:10), starting from (*R*)-**4a** (422 mg, 1.66 mmol) and following general procedure **B**.

* The diastereomeric ratio was determined by ^{1}H NMR analysis of the crude mixture and integrating the signals of the two diastereoisomers.

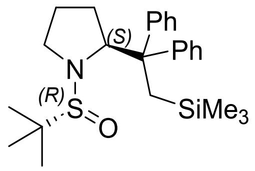

7d

Y% = 74. ***er*** =97.5:2.5†. **[α]$_D$^{20}** = -87.6 (c = 1.4, $CHCl_3$). **^{1}H NMR** (600 MHz, $CDCl_3$) δ 7.36 – 7.32 (m, 4H), 7.28 (t, J = 8.0, 2H), 7.23 – 7.16 (m, 4H), 4.73 (dd, J = 9.1, 3.0, 1H), 3.40 (ddd, J = 10.1, 8.5, 6.8, 1H), 2.04 – 1.97 (m, 1H), 1.93 – 1.89 (m, 2H), 1.79 – 1.70 (m, 1H), 1.43 – 1.46 (m, 1H), 1.25 (d, *J* = 14.5 Hz, 1H), 1.10 (s, 9H), 0.43 – 0.36 (m, 1H), 0.39 (s, 9H). **^{13}C{^{1}H}** (150 MHz, $CDCl_3$) δ 146.1, 130.2, 127.5, 127.4, 126.5, 126.3, 75.6, 58.7, 55.0, 43.4, 31.0, 28.6, 26.1, 24.6, 0.2. **LRMS** (ESI) *m/z* = 428.1 $[M+H]^+$, 450.2 $[M+Na]^+$, 877.2 $[2M+Na]^+$. **HRMS** *m*/z: [M + Na]$^+$ Calcd. for $[C_{25}H_{37}NNaOSSi]^+$ 450.2257; Found. 450.2255.

(*S*)-1-((*R*)-tert-butylsulfinyl)-2-(2-(dimethyl(phenyl)silyl)-1,1-diphenylethyl)azetidine (10)

Product **10** was obtained as a colorless oil in 25% isolated yield (43 mg, 0.09 mmol) after purification with flash column chromatography (CyH:EtOAc = 90:10), starting from (*R*)-**4a** (87 mg, 0.36 mmol) and following general procedure **A**.

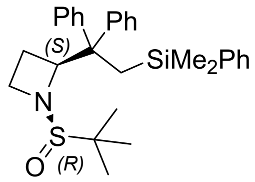

10

Y% = 25. ***d.r*** =99:1‡. **[α]$_D$^{20}** = - 67.6 (c = 0.7 $CHCl_3$). ***d.r.*** = 99:1, λ = 210 nm τ_{major} = 16.7 min, τ_{minor} = 18.6 min. **^{1}H NMR** (600 MHz, $CDCl_3$) δ 7.37 – 7.35 (m, 3H), 7.30 – 7.28 (m, 3H), 7.24 – 7.20 (m, 7H), 7.09 – 7.07 (m, 2H), 4.98 (dd, J = 9.2, 6.1 Hz, 1H), 4.02 (ddd, J = 10.4, 8.3, 6.4 Hz, 1H), 2.45 – 2.39 (m, 1H), 2.35 – 2.30 (m, 1H), 2.06 (d, J = 14.7 Hz, 1H), 1.94 – 1.88 (m, 1H), 1.60 (d, J = 14.7 Hz, 1H), 1.10 (s, 9H), 0.17 (s, 3H), -0.24 (s, 3H). **^{13}C{^{1}H} NMR** (150 MHz, $CDCl_3$) δ 146.2, 144.9, 140.4, 133.6, 130.2, 129.1, 128.8, 127.9, 127.7, 127.0, 126.6, 126.3, 68.5, 57.9, 53.3, 39.3, 27.4, 23.9, 22.5, -0.9, -1.8. **LRMS** (ESI) *m/z* = 498.2 $[M+Na]^+$. **HRMS** *m*/z: [M + Na]$^+$ Calcd. for $[C_{29}H_{37}NNaOSSi]^+$ 498.2257; Found. 498.2260.

(*S*)-1-((*R*)-tert-butylsulfinyl)-2-(2-(dimethyl(phenyl)silyl)-1,1-diphenylethyl)piperidine (11)

Product **11** was obtained as a colorless oil in 69% isolated yield (223mg, 0.44 mmol) after purification with flash column chromatography (CyH:EtOAc = 90:10), starting from (*R*)-**4a** (172 mg, 0.64 mmol) and following general procedure **A**.

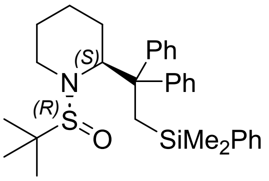

11

Y% = 69. ***d.r*** = 94:6‡. **[α]$_D$^{20}** = -46.1 (c = 0.9, $CHCl_3$). ***dr*** = 94:6, λ = 210 nm τ_{minor} = 18.3 min, τ_{major} = 20.4 min. **^{1}H NMR** (600 MHz, $CDCl_3$) δ 7.43 – 7.38 (m, 4H), 7.35 – 7.30 (m, 2H), 7.29 – 7.16 (m, 9H), 4.44 (t, *J* = 8.2 Hz, 1H), 2.97 – 2.82 (m, 1H), 2.02 – 1.94 (m, 1H), 1.74 (s, 2H), 1.70 – 1.61 (m, 2H), 1.53 – 1.45 (m, 1H), 1.44 – 1.36 (m, 1H), 1.36 – 1.26 (m, 1H), 1.04 (s, 9H), 0.89 – 0.76 (m, 1H), -0.20 (2, 3H), -0.37 (s, 3H).**^{13}C{^{1}H} NMR** (150 MHz, $CDCl_3$) δ 143.7, 143.2, 140.7, 133.3, 130.8, 130.4, 128.7, 127.7, 127.5, 127.3, 126.6, 126.5, 69.9, 59.4, 55.6, 41.6, 31.0, 25.3, 23.8, 23.1, 19.0, -1.9, -2.1. **LRMS** (ESI) *m/z* = 526.2 $[M+Na]^+$, 542.2 $[M+K]^+$. **HRMS** *m*/z: [M + Na]$^+$ Calcd. for $[C_{31}H_{41}NNaOSSi]^+$ 526.2570; Found. 526.2568.

† The enantiomeric ratio was determined after removal of the sulfinyl group and benzoylation reaction by chiral HPLC analysis. See Section 10.1 for the chromatograms.
‡ The diastereomeric ratio was determined by means of HPLC-MS analysis integrating the peaks of the two diastereoisomers. See section 9 for the chromatograms and MS-Spectra.

5 Characterization data of products 8b-d

(*S*)-2-(2-(dimethyl(phenyl)silyl)-1,1-diphenylethyl)pyrrolidine (8b)

Product **8b** was obtained as a white solid in 95% isolated yield (194 mg, 0.5 mmol) after purification with flash column chromatography (DCM:MeOH = 95:5), starting from **7b** (258 mg, 0.53 mmol) and following general procedure **C**.

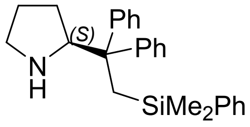

8b

Y% = 95. **m.p.** = 112-114 °C $[\alpha]_D^{20}$ = +5.2 (c = 1.3, $CHCl_3$). The enantiomeric excess was determined to be >99% by HPLC analysis on a Daicel Chiralpak IC column: 90:10 hexane/IPA, flow rate = 0.8 mL/min, λ = 254 nm, τ_{major} = 6.17 min, τ_{minor} = 7.53 min. **^{1}H NMR** (600 MHz, $CDCl_3$) δ 7.46 – 7.44 (m, 2H), 7.36 – 7.32 (m, 5H), 7.28 – 7.25 (m, 6H), 7.23 – 7.18 (m, 2H), 3.78 (t, J = 7.7 Hz, 1H), 2.64 – 2.60 (m, 1H), 2.45 – 2.41 (m, 1H), 1.90 (d, *J* = 14.4 Hz, 1H), 1.86 (d, *J* = 14.4 Hz, 1H), 1.77 – 1.71 (m, 1H), 1.44 – 1.37 (m, 1H), 1.30 – 1.24 (m, 1H), 1.13 – 1.06 (m, 1H), -0.08 (s, 3H), -0.18 (s, 3H). **^{13}C{^{1}H} NMR** (150 MHz, $CDCl_3$) δ 147.9, 146.7, 140.6, 133.6, 129.8, 129.5, 128.8, 127.8, 127.7, 127.5, 126.2, 126.1, 63.4, 53.4, 46.7, 30.5, 28.0, 25.2, -1.8, -2.0. **LRMS** (ESI) *m/z* = 386.3 $[M+H]^+$. **HRMS** *m*/z: $[M + H]^+$ Calcd. for $[C_{26}H_{32}NSi]^+$ 386.2299; Found. 386.2301.

(*S*)-2-(2-(methyldiphenylsilyl)-1,1-diphenylethyl)pyrrolidine (8c)

Product **8c** was obtained as a white solid in 88% isolated yield (172 mg, 0.38 mmol) after purification with flash column chromatography (DCM:MeOH = 95:5), starting from **7c** (241 mg, 0.44 mmol) and following general procedure **C**.

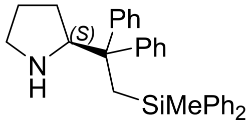

8c

Y% = 88. **m.p.** = 112–114 °C. $[\alpha]_D^{20}$ = +4.9 (c = 0.5, $CHCl_3$). The enantiomeric excess was determined to be 94% by HPLC analysis on a Daicel Chiralpak IC column: 90:10 hexane/IPA, flow rate = 0.8 mL/min, λ = 254 nm, τ_{major} = 6.42 min, τ_{minor} = 7.69 min. **^{1}H NMR** (600 MHz, $CDCl_3$) δ 7.47 – 7.42 (m, 4H), 7.34 – 7.27 (m, 7H), 7.28 – 7.23 (m, 5H), 7.22 – 7.18 (m, 3H), 7.16 – 7.13 (m, 1H), 3.60 (t, J = 7.4 Hz, 1H), 2.50 – 2.46 (m, 1H), 2.34 – 2.30 (m, 2H), 2.21 (d, J = 14.3 Hz, 1H), 1.64 – 1.58 (m, 1H), 1.28 – 1.19 (m, 2H), 1.01 – 0.94 (m, 1H), - 0.17 (s, 3H). **^{13}C{^{1}H} NMR** (150 MHz, $CDCl_3$) δ 147.8, 146.9, 138.6, 138.5, 134.7, 134.5, 129.7, 129.6, 129.1, 129.0, 127.8, 127.8, 127.7, 127.6, 126.2, 126.1, 63.0, 53.3, 46.5, 27.9, 25.1, -4.0. **LRMS** (ESI) *m/z* = $[M+H]^+$. **HRMS** *m*/z: $[M + H]^+$ Calcd. for $[C_{31}H_{34}NSi]^+$ 448.2455; Found. 448.2458.

(*S*)-2-(1,1-diphenyl-2-(trimethylsilyl)ethyl)pyrrolidine (8d)

Product **8d** was obtained as a yellow wax in 90% isolated yield (293 mg, 0.91 mmol) after purification with flash column chromatography (DCM:MeOH = 95:5), starting from **7d** (428 mg, 1.0 mmol) and following general procedure **C**.

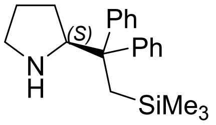

8d

Y% = 90. $[\alpha]_D^{20}$ = +8.7 (c = 1.1, $CHCl_3$). The enantiomeric excess was determined to be 95% by HPLC analysis on a Daicel Chiralpak IC column: 90:10 hexane/IPA, flow rate = 0.8 mL/min, λ = 254 nm, τ_{major} = 5.75 min, τ_{minor} = 7.26 min **^{1}H NMR** (600 MHz, $CDCl_3$) δ 7.35 – 7.32 (m, 2H), 7.29

– 7.24 (m, 6H), 7.22 – 7.16 (m, 2H), 3.98 (t, J = 7.8 Hz, 1H), 2.77 – 2.73 (m, 1H), 2.51 – 2.48 (m, 1H), 1.90 – 1.84 (m, 1H), 1.62 (d, J = 3.3 Hz, 2H), 1.59 – 1.54 (m, 1H), 1.37 – 1.31 (m, 1H), 1.21 – 1.15 (m, 1H), - 0.33 (s, 9H). **^{13}C{^{1}H} NMR** (150 MHz, $CDCl_3$) δ 129.8, 129.4, 127.8, 127.4, 126.1, 126.0, 64.0, 46.9, 31.0, 30.9, 28.0, 25.3, 0.10. **LRMS** (ESI) *m/z* = 324.2 $[M+H]^+$. **HRMS** *m*/z: $[M + H]^+$ Calcd. for $[C_{21}H_{30}NSi]^+$ 324.2142; Found. 324.2145.

6 Determination of the stereochemistry of products 8b-d

The (*S*)-stereochemistry of the newly formed stereocenter at C2 in products **8b–d** was determined by comparing the results obtained using (*S*)-**9**a and our catalysts in the benchmark organocatalyzed Michael addition of aldehydes **12** to β-nitrostyrene **13**. Since both catalyst (*S*)-**9a** and all our derivatives **8** favor the formation of the same enantiomer (Figure S2), this indicates that both catalysts have the same absolute configuration.

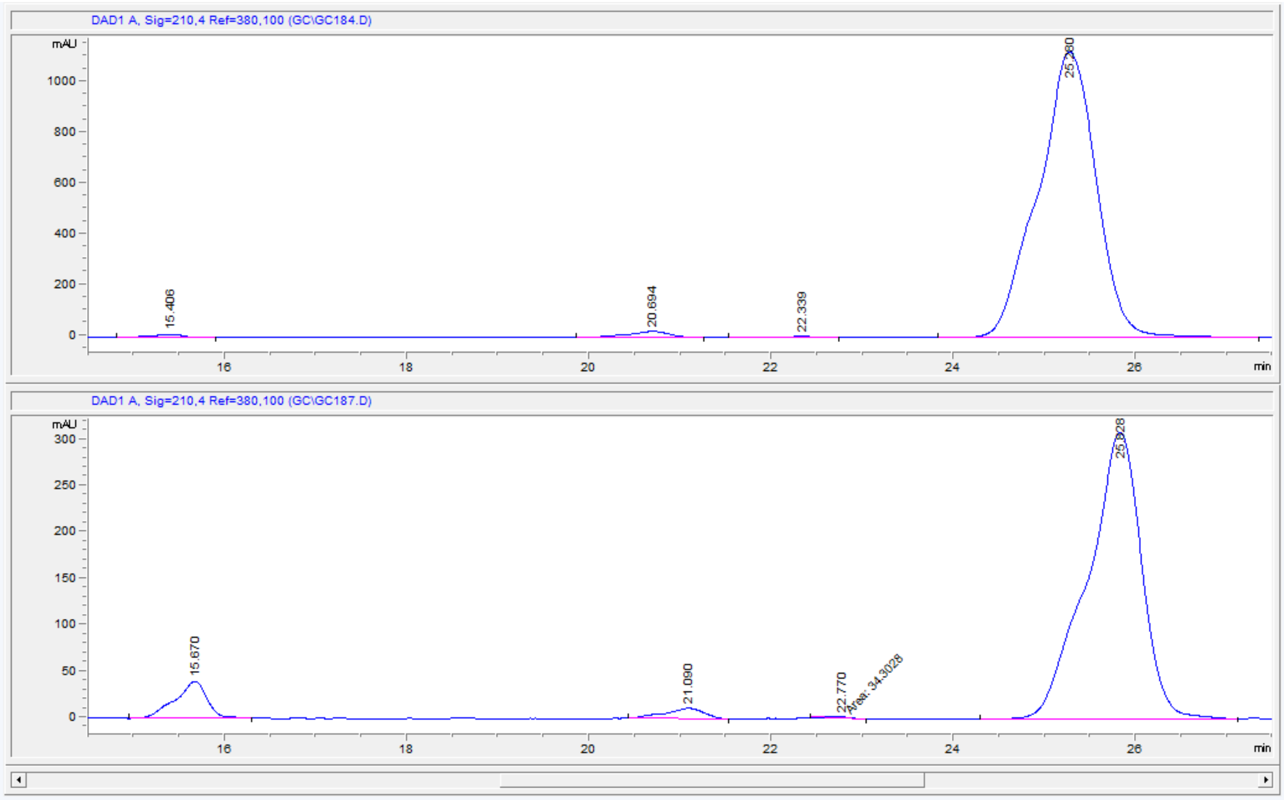

Figure S2 – Comparison of the chiral HPLC traces of the products from the benchmark organocatalyzed Michael addition of propanal **12a** to β-nitrostyrene **13**, performed using (*S*)-**9a** (top) and catalyst **8b** (bottom).

7 Mechanistic studies

7.1 General procedure for enamine preparation

In a 2 mL screw-cap vial, the selected catalyst **9a** or **8b–d** (60 µmol) was dissolved in $CDCl_3$ (0.6 mL). The resulting solution was transferred into a 5 mm NMR tube, and phenylacetaldehyde **15** (1.1 equiv., 66 µmol, 7.3 µL) was added. The final solution, containing the corresponding enamines **16a–d**, was fully characterized by means of NMR analysis with a Bruker-600 instrument.

7.2 Characterization of enamine **16a**

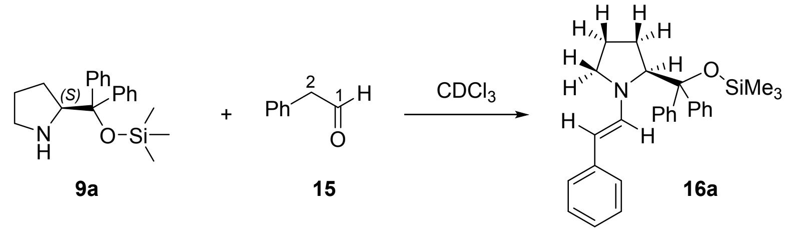

^{1}H NMR (600 MHz, $CDCl_3$) δ 7.49 – 7.44 (m, 4H), 7.39 – 7.30 (m, 6H), 7.19 (d, J = 15.0 Hz, 1H), 7.14 (t, J = 7.8 Hz, 2H), 7.01 (d, *J* = 7.2 Hz, 2H), 6.90 (tt, *J* = 7.3, 1.3 Hz, 1H), 4.96 (d, *J* = 14.0 Hz, 1H), 4.59 (dd, *J* = 9.1, 2.1 Hz, 1H), 2.89 (q, *J* = 8.5 Hz, 1H), 2.53 (td, *J* = 9.3, 3.0 Hz, 1H), 2.04 (ddt, *J* = 13.4, 10.8, 9.0 Hz, 1H), 1.93 (ddt, *J* = 13.3, 8.4, 2.4 Hz, 1H), 1.48 – 1.39 (m, 1H), 0.57 – 0.47 (m, 1H), -0.17 (s, 9H). **$^{13}C\{^1H\}$ NMR** (150 MHz, $CDCl_3$) δ 142.9, 142.4, 140.5, 137.6, 129.9, 129.6, 128.5, 127.7, 127.7, 127.6, 127.5, 123.3, 122.7, 97.2, 84.0, 70.1, 48.6, 28.0, 22.6, 2.1.

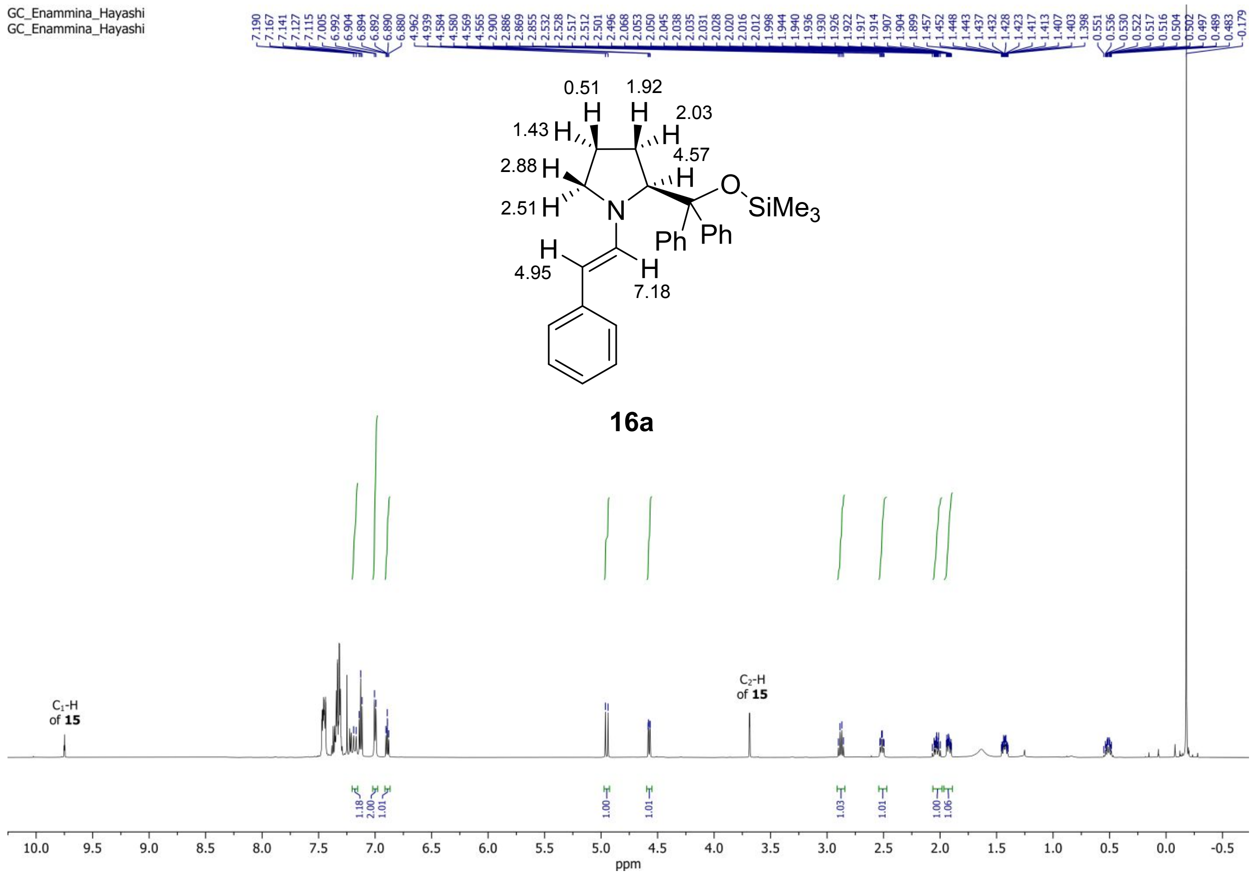

Figure S3 - ^{1}H NMR spectrum of (*S*) – **16a** recorded at 600MHz. The most relevant ^{1}H-NMR chemical shifts, determined through COSY and HSQC analyses, are reported in the corresponding molecule. The impurities unrelated to the product are marked in the spectrum.

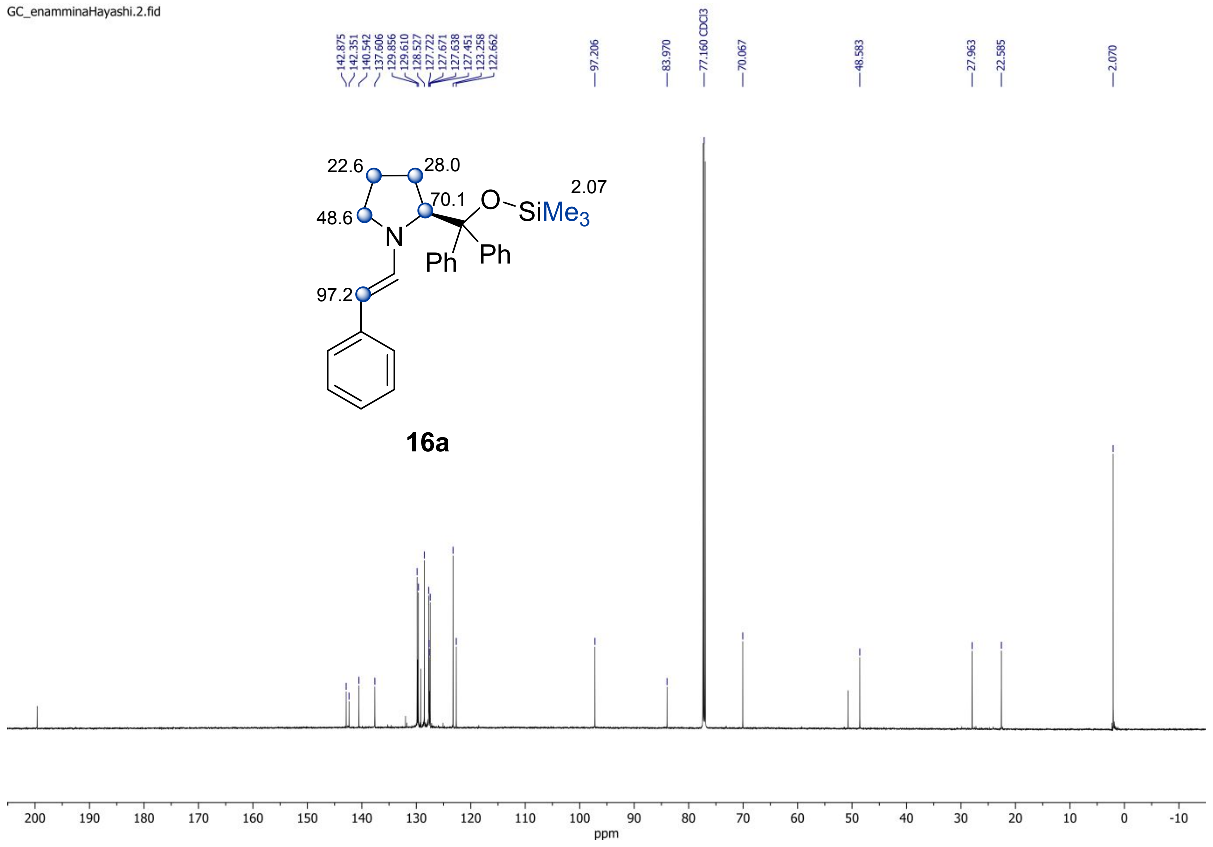

Figure S4 - $^{13}C\{^{1}H\}$ NMR spectrum of (*S*)-**16a** recorded at 150MHz. The most relevant ^{13}C-NMR chemical shifts, determined through HSQC analyses, are reported in the corresponding molecule.

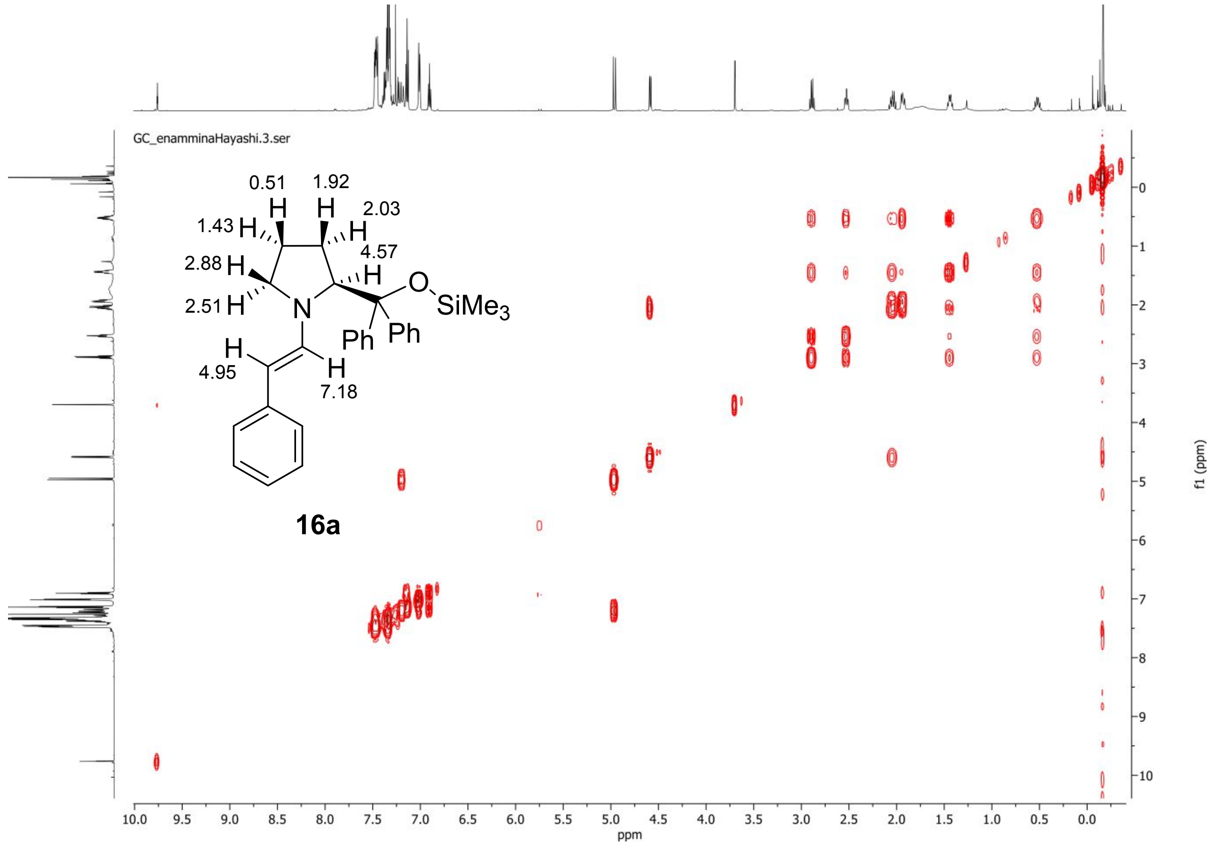

Figure S5 – ^{1}H – ^{1}H -COSY of (*S*)-**16a** recorded at 600 MHz.

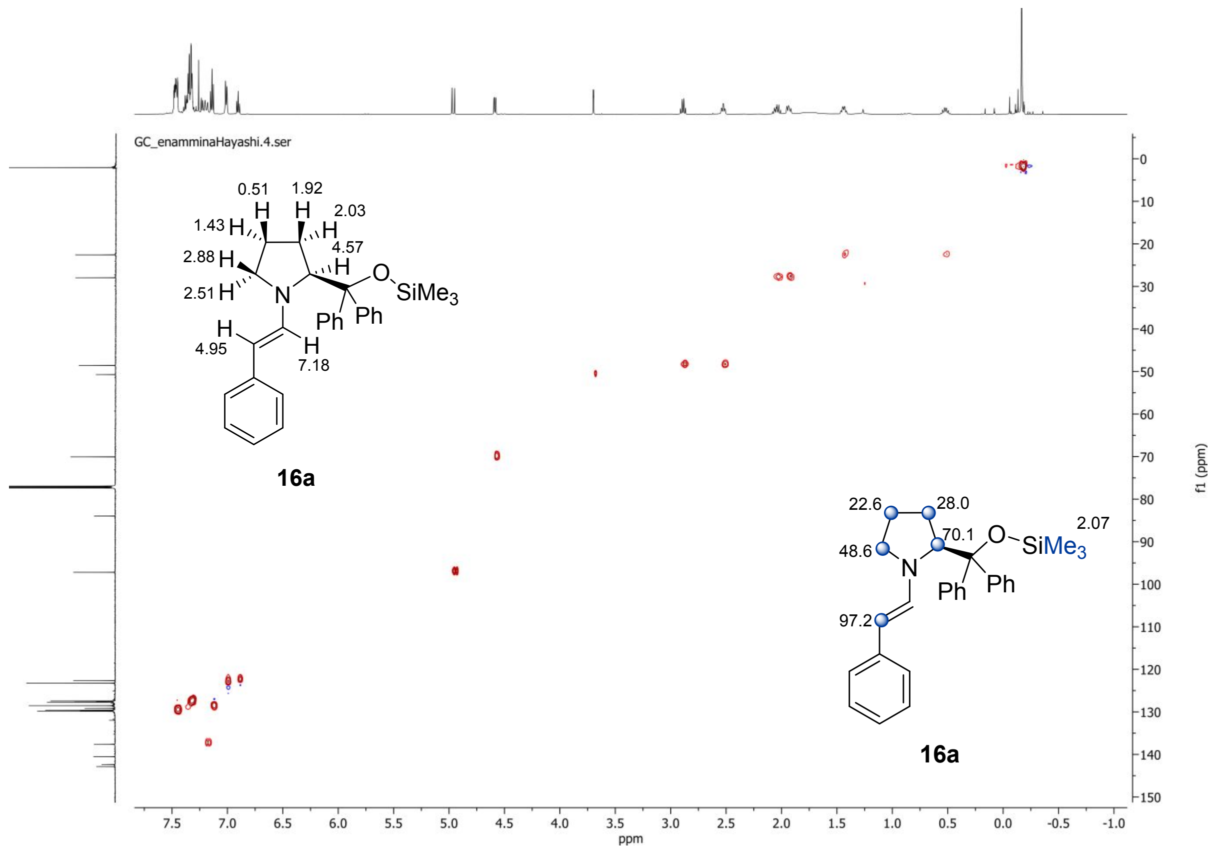

Figure S6 - ^{1}H – ^{13}C -HSQC of (*S*)-**16a** recorded at 600 MHz.

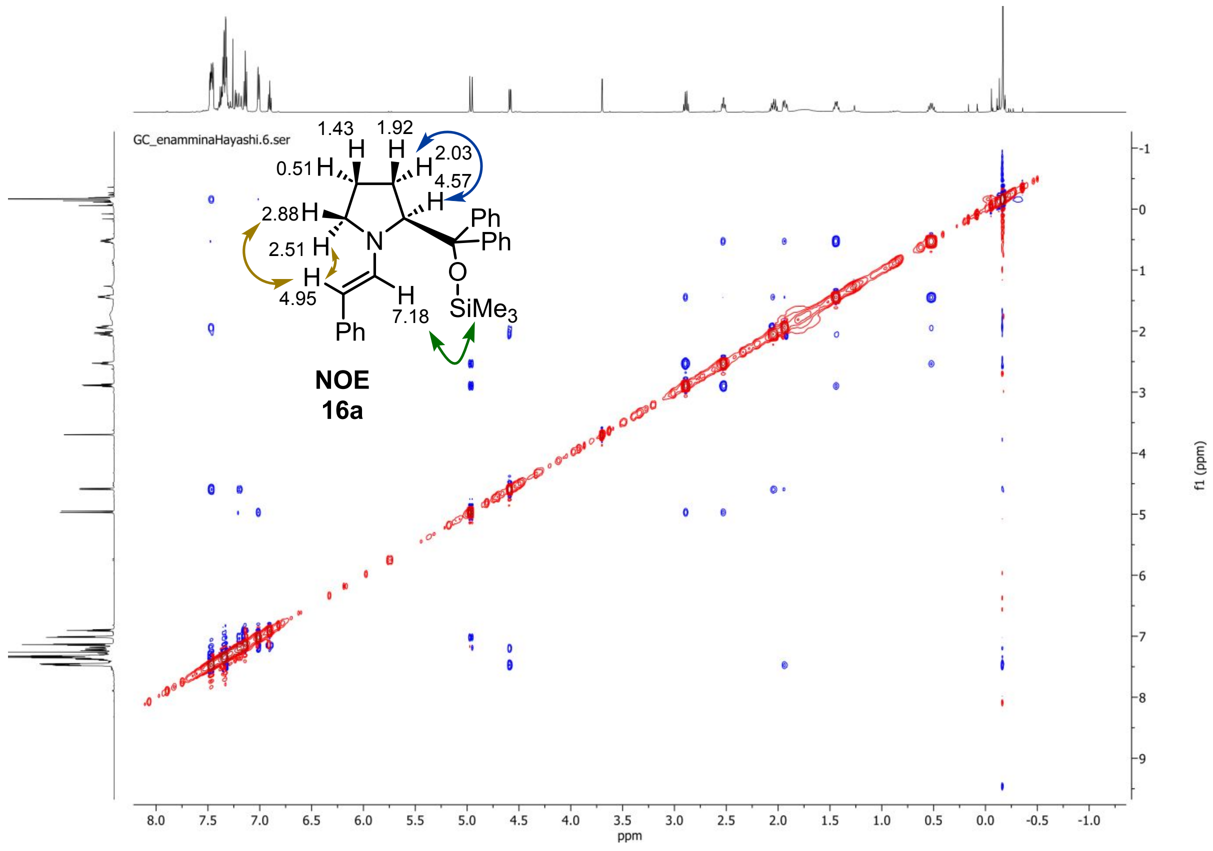

Figure S7 - 2D-NOESY of (*S*) – **16a** recorded at 600 MHz. The most important correlations are highlighted in the reported molecule.

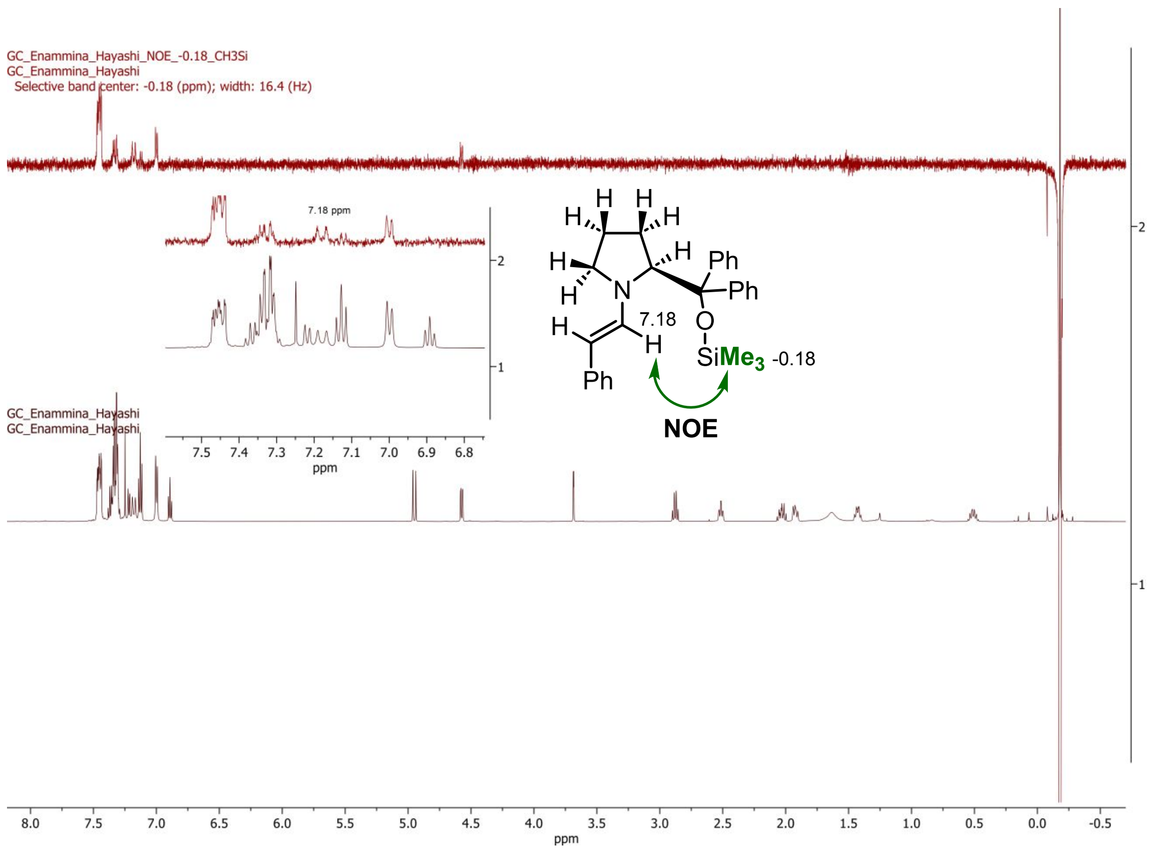

Figure S8 – NOESY – 1D of enamine (*S*)-**16a** recorded at 400 MHz. Irradiated proton: -0.18 ppm ($\mathbf{CH_3}$)$_3$-Si-. The responding proton is shown in the corresponding molecule.

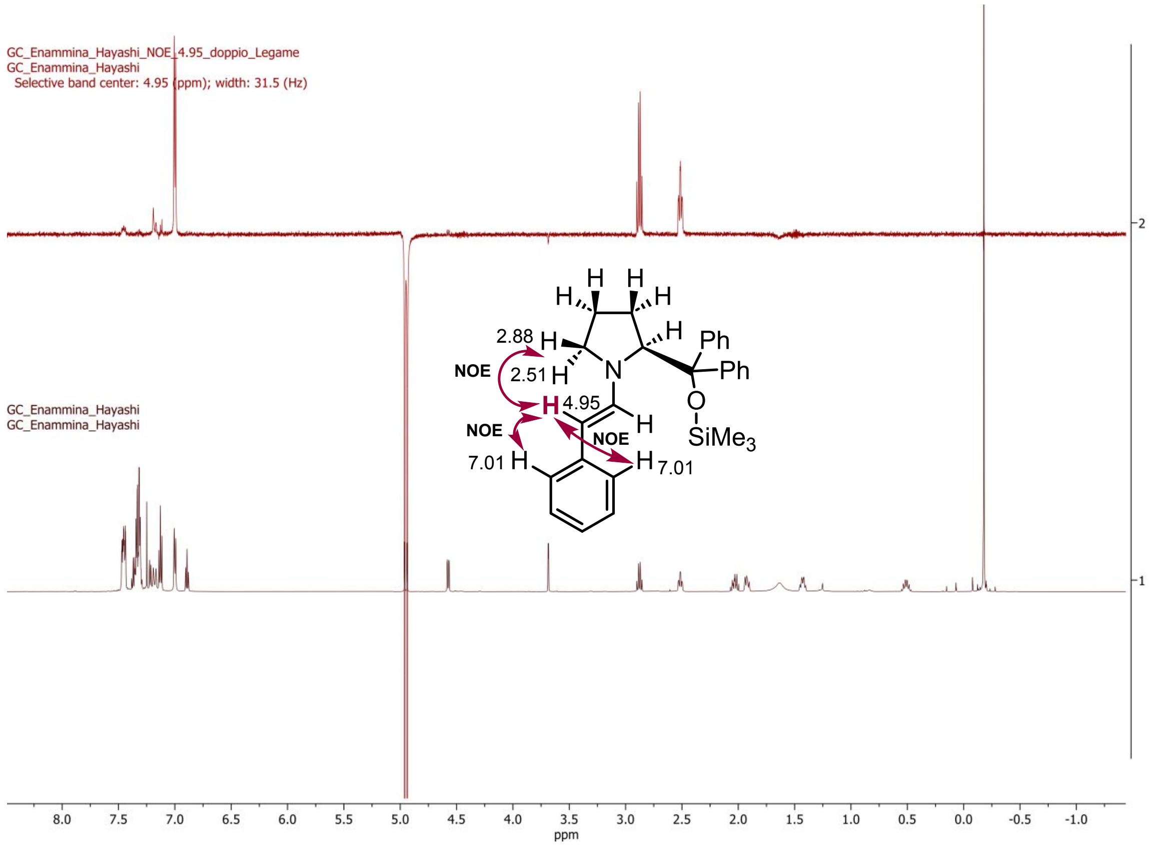

Figure S9 - NOESY – 1D of enamine (*S*)-**16a** recorded at 400 MHz. Irradiated proton: 4.95 ppm Ph-C(**H**)=C(H)-. The responding protons are shown in the corresponding molecule.

The NMR-analyses performed on enamine **16a** showed that also in solution its preferred conformation is a *sc-exo* (Figure S10) as already reported by Seebach[6] and Uchimaru[7] by employing crystal structure analysis and DFT calculations. We used this analysis as a reference for comparison with our catalysts.

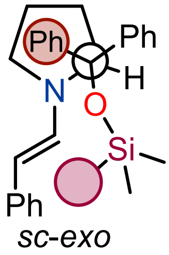

Figure S10 - Preferred conformation adopted by enamine (***S***)-**16a**

7.3 Characterization of enamine **16b**

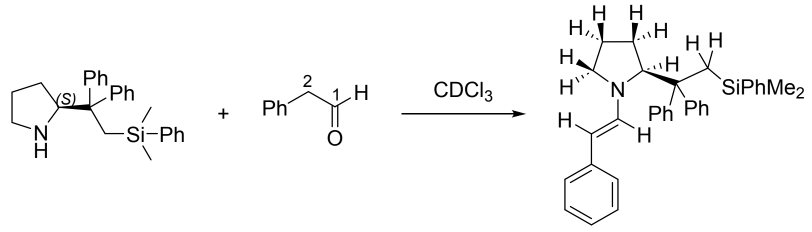

^{1}H NMR (600 MHz, $CDCl_3$) δ 7.29 – 7.25 (m, 4H), 7.24 – 7.20 (m, 3H), 7.19 – 7.11 (m, 8H), 7.01 (t, *J* = 7.7 Hz, 2H), 6.98 (d, *J* = 13.2 Hz, 1H), 6.82 (t, *J* = 7.3 Hz, 1H), 6.79 (d, *J* = 7.6 Hz, 2H), 4.91 (d, *J* = 13.8 Hz, 1H), 4.38 (dd, *J* = 8.6, 2.2 Hz, 1H), 2.75 (q, *J* = 8.6 Hz, 1H), 2.52 (td, *J* = 9.3, 3.0 Hz, 1H), 2.14 (d, *J* = 15.3 Hz, 1H), 1.92 (ddt, *J* = 13.2, 10.8, 8.8 Hz, 1H), 1.74 (ddt, *J* = 13.0, 8.1, 2.5 Hz, 1H), 1.48 (d, *J* = 15.0 Hz, 1H), 1.29 – 1.19 (m, 1H), 0.10 – 0.04 (m, 1H), 0.00 (s, 3H), -0.46 (s, 3H). **^{13}C{^{1}H} NMR** (150 MHz, $CDCl_3$) δ 146.0, 140.5, 140.2, 137.9, 133.5, 130.5, 129.8, 129.2, 128.8, 128.5, 127.8, 127.7, 127.5, 126.6, 126.5, 123.5, 122.9, 97.8, 70.6, 54.9, 49.5, 29.6, 28.6, 22.5, -0.9, -1.5.

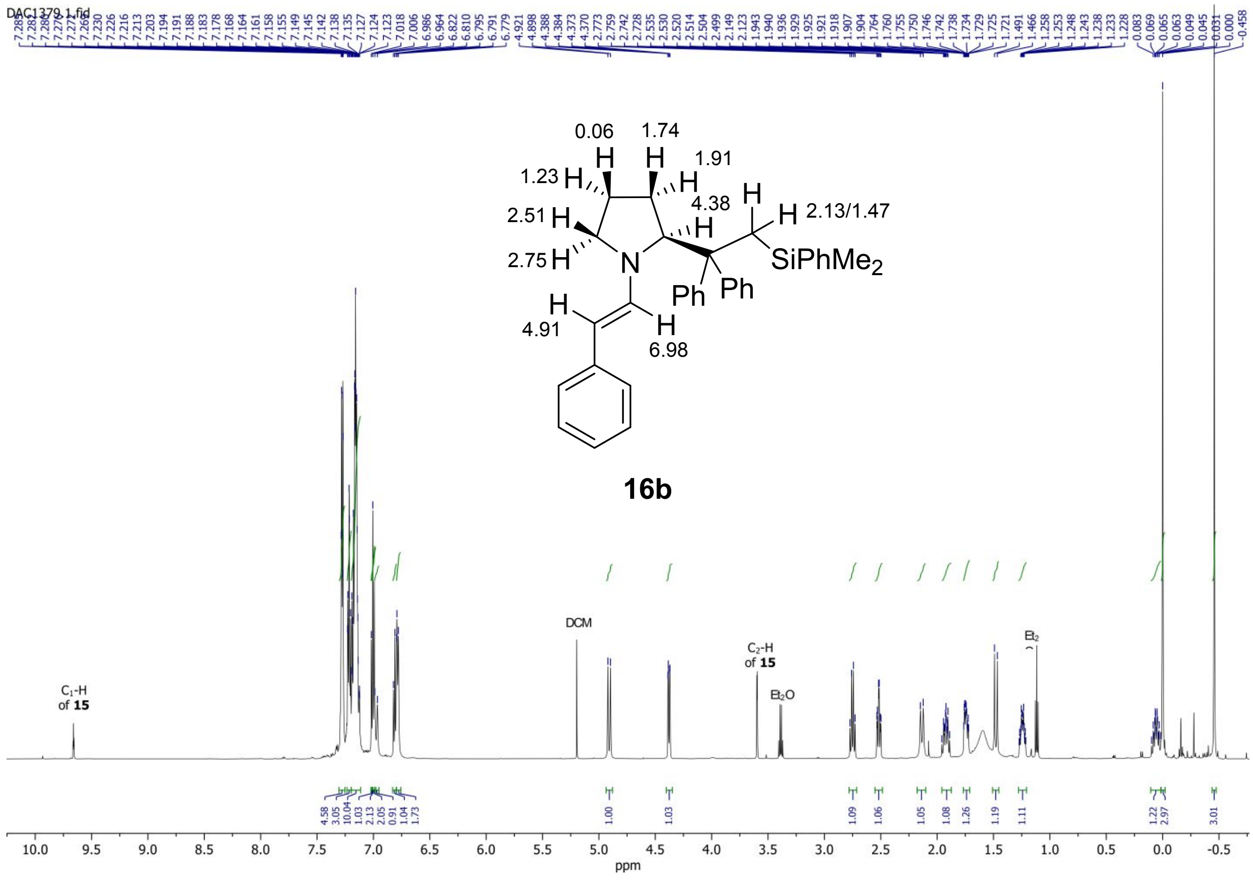

Figure S11 – ^{1}H NMR spectrum of (*S*) - **16b** recorded at 600MHz. The most relevant ^{1}H-NMR chemical shifts, determined through COSY and HSQC analyses, are reported in the corresponding molecule. The impurities unrelated to the product are marked in the spectrum. DCM = dichloromethane, Et_2O = diethyl ether.

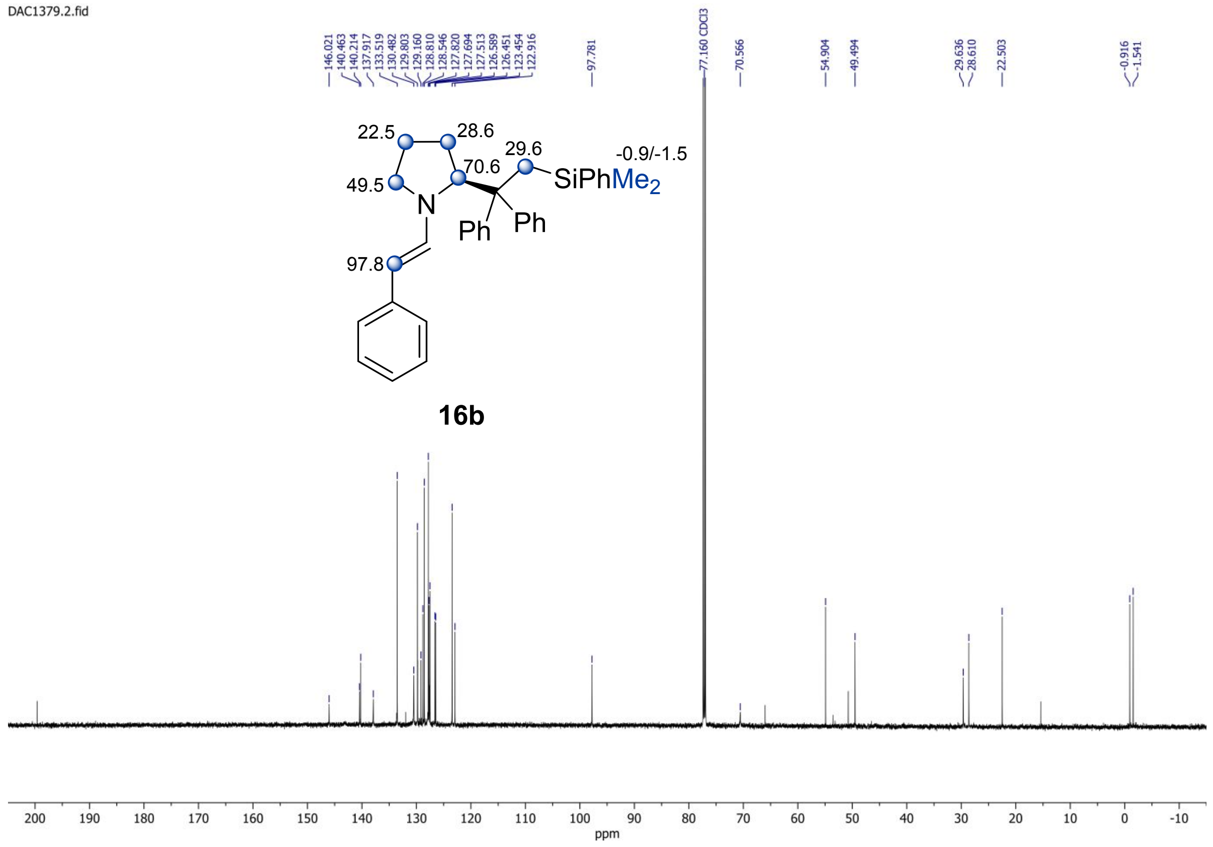

Figure S12 – $^{13}C\{^{1}H\}$ NMR spectrum of (*S*) - **16b** recorded at 150MHz. The most relevant ^{13}C-NMR chemical shifts, determined through HSQC analyses, are reported in the corresponding molecule.

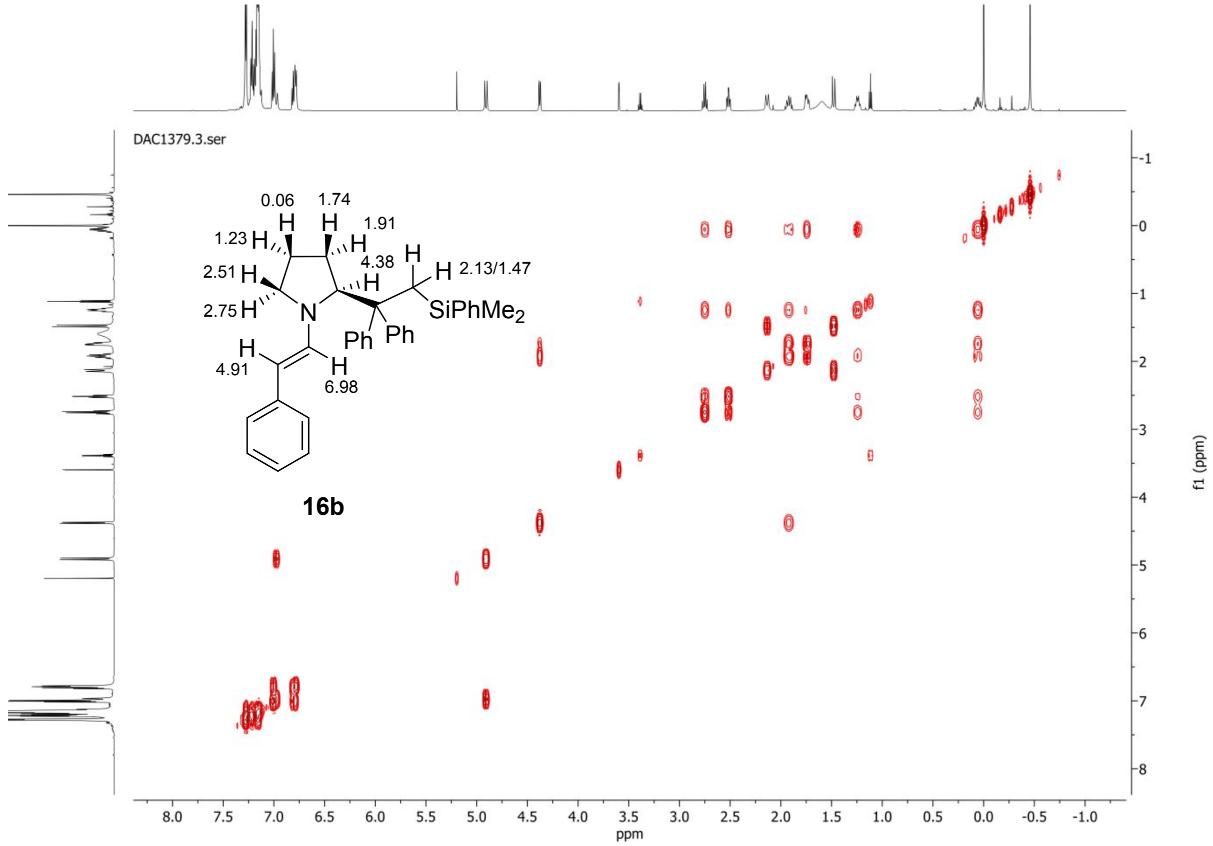

Figure S13 – ^{1}H – ^{1}H -COSY of (*S*) - **16b** recorded at 600 MHz.

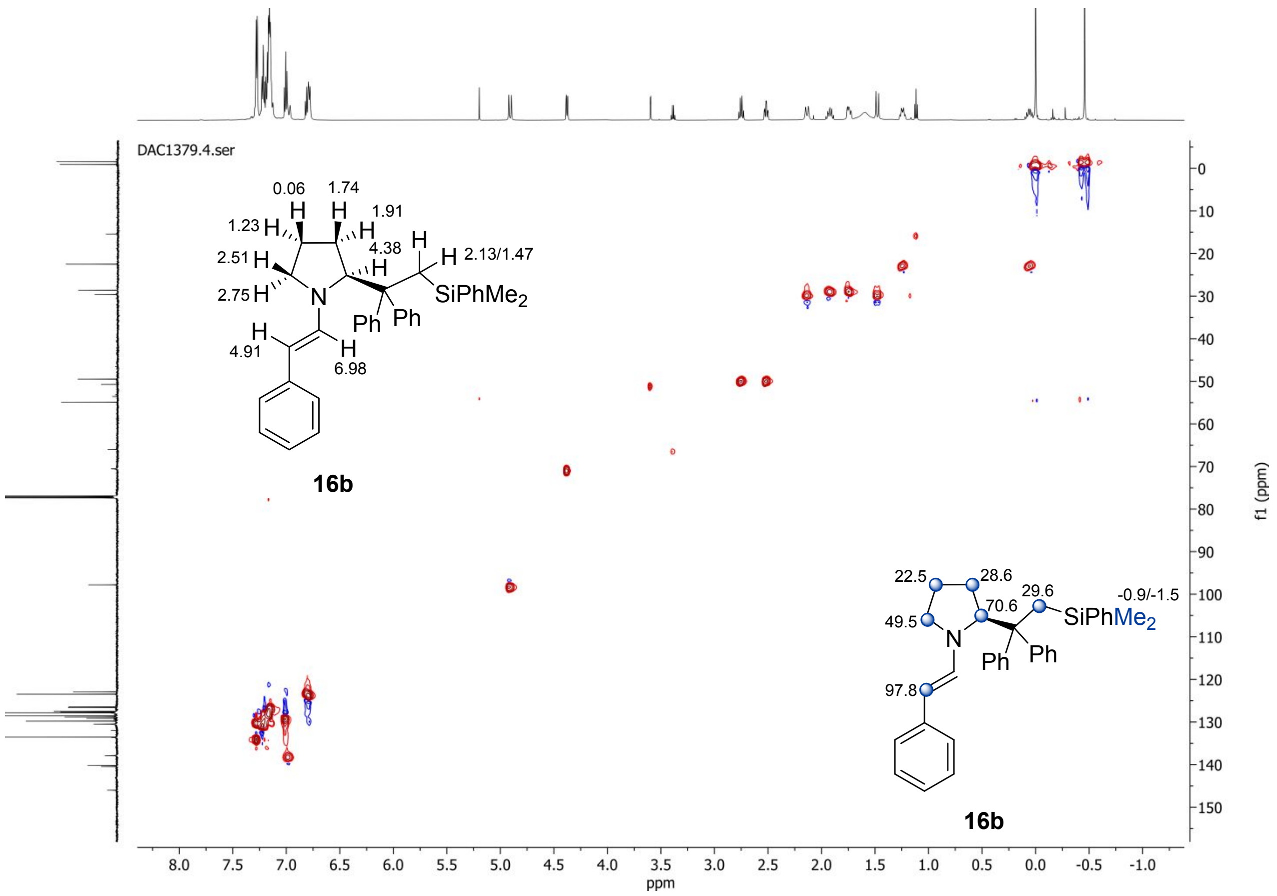

Figure S14 - ^{1}H – ^{13}C -HSQC of (*S*) - **16b** recorded at 600 MHz

NOESY

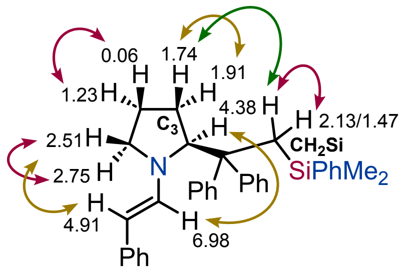

16b

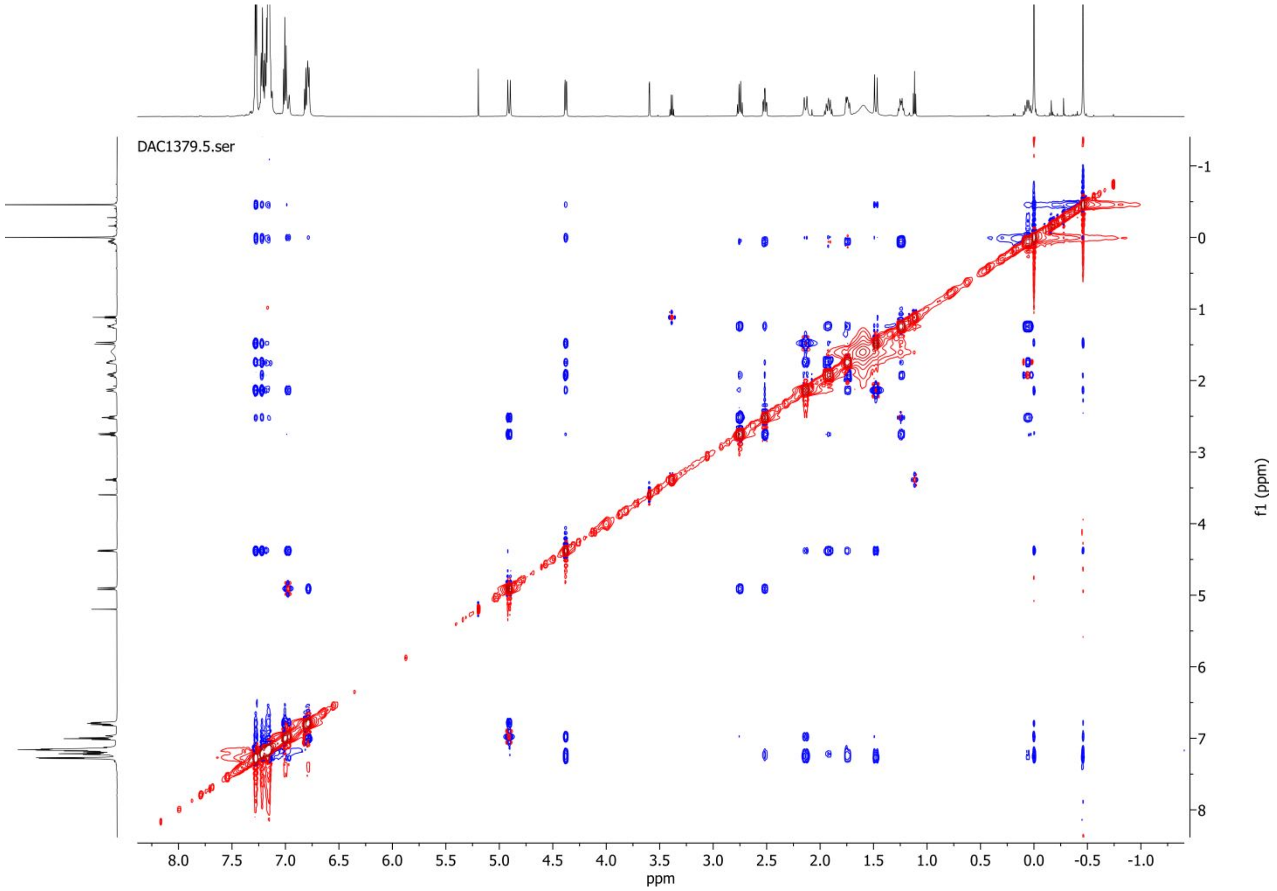

Figure S15 – 2D-NOESY of **16b** recorded at 600 MHz. The most important correlations are highlighted in the reported molecule.

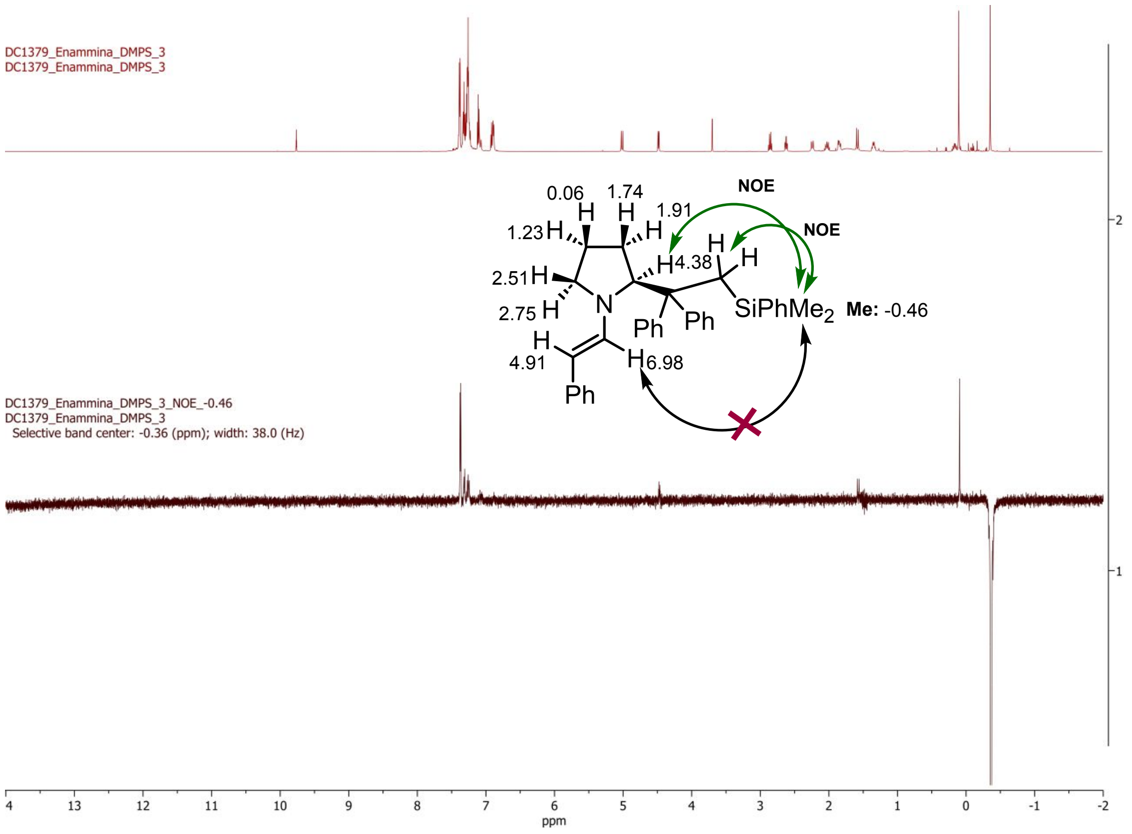

Figure S16 - NOESY – 1D of enamine (*S*)-**16b** recorded at 400 MHz. Irradiated proton: -0.46 ppm -SiPh**Me**$_2$. The responding protons are shown in the corresponding molecule. No NOE response is observed between the double bond proton at 6.98 ppm and the irradiated methyl protons.

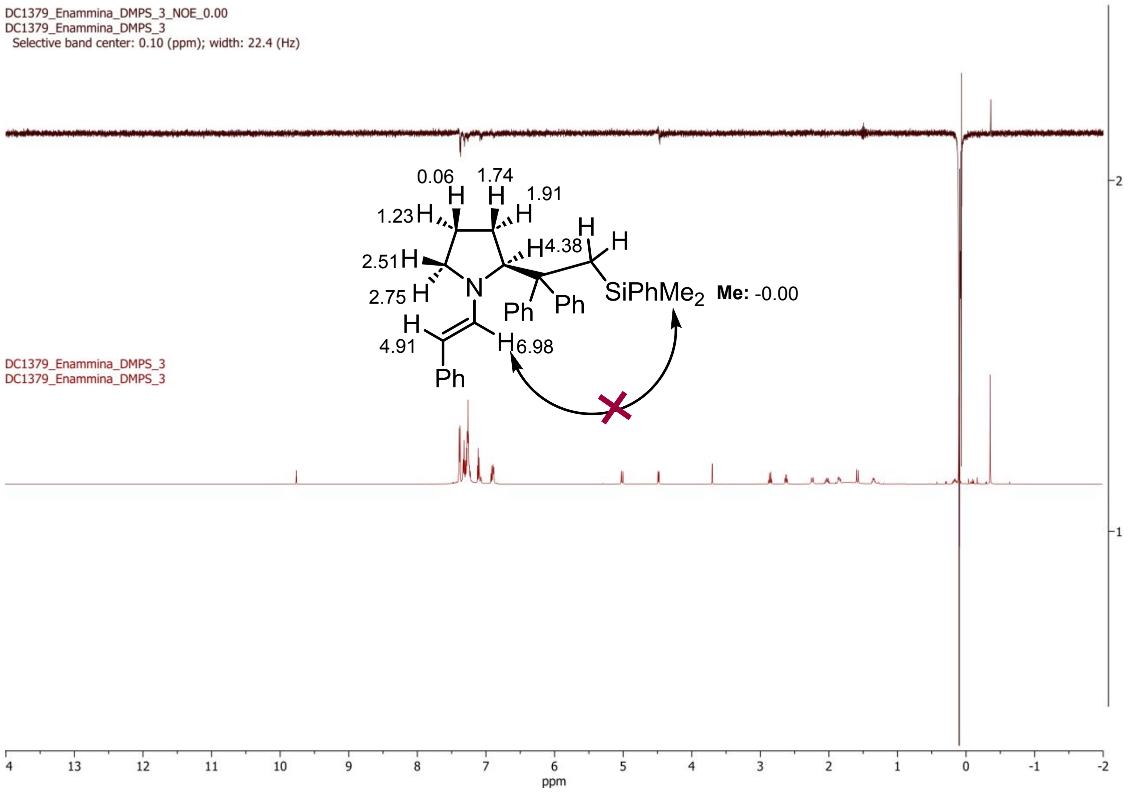

Figure S17 - NOESY – 1D of enamine (*S*)-**16b** recorded at 400 MHz. Irradiated proton: -0.00 ppm -SiPh**Me**$_2$. No NOE response is observed between the double bond proton at 6.98 ppm and the irradiated methyl protons.

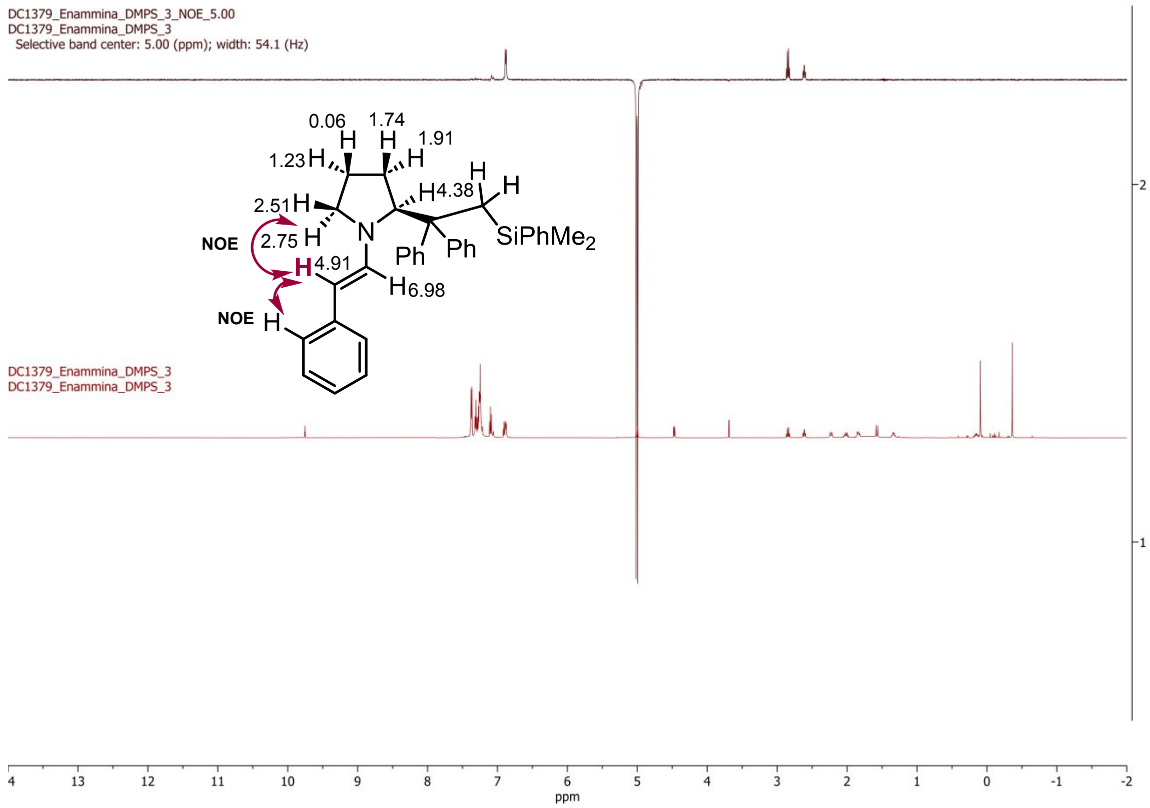

Figure S18 - NOESY – 1D of enamine (*S*)-**16b** recorded at 400 MHz. Irradiated proton: 4.91 ppm Ph-C(**H**)=C(H)-. The responding protons are shown in the corresponding molecule.

7.4 Characterization of enamine **16c**

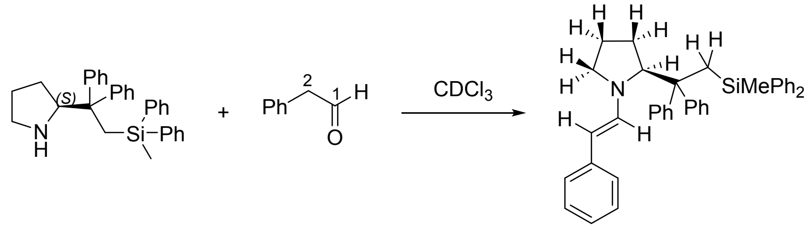

8c **15** **16c**

^{1}H NMR (600 MHz, $CDCl_3$) δ 7.24 (d, J = 6.7 Hz, 2H), 7.18 (t, J = 7.6 Hz, 1H), 7.14 – 7.08 (m, 4H), 7.05 (d, J = 6.8 Hz, 2H), 7.04 – 7.01 (m, 3H), 6.97 – 6.93 (m, 6H), 6.93 – 6.88 (m, 3H), 6.85 (t, J = 7.7 Hz, 2H), 6.69 (t, J = 7.3 Hz, 1H), 6.63 (d, J = 7.8 Hz, 2H), 4.84 (d, J = 13.7 Hz, 1H), 4.24 (dd, J = 8.7, 2.1 Hz, 1H), 2.66 (q, J = 8.8 Hz, 1H), 2.53 – 2.37 (m, 2H), 1.89 – 1.84 (m, 1H), 1.84 – 1.79 (m, 1H), 1.70 – 1.62 (m, 1H), 1.19 – 1.10 (m, 1H), 0.00 (s, 3H), -0.02 – -0.10 (m, 1H). **^{13}C{^{1}H} NMR** (150 MHz, $CDCl_3$) δ 145.7, 140.1, 138.2, 138.2, 137.9, 134.8, 134.2, 130.4, 129.8, 129.2, 128.7, 128.5, 127.9, 127.6, 127.5, 127.3, 126.6, 126.4, 123.5, 122.9, 98.0, 70.6, 54.8, 49.6, 28.8, 28.1, 22.5, -2.8.

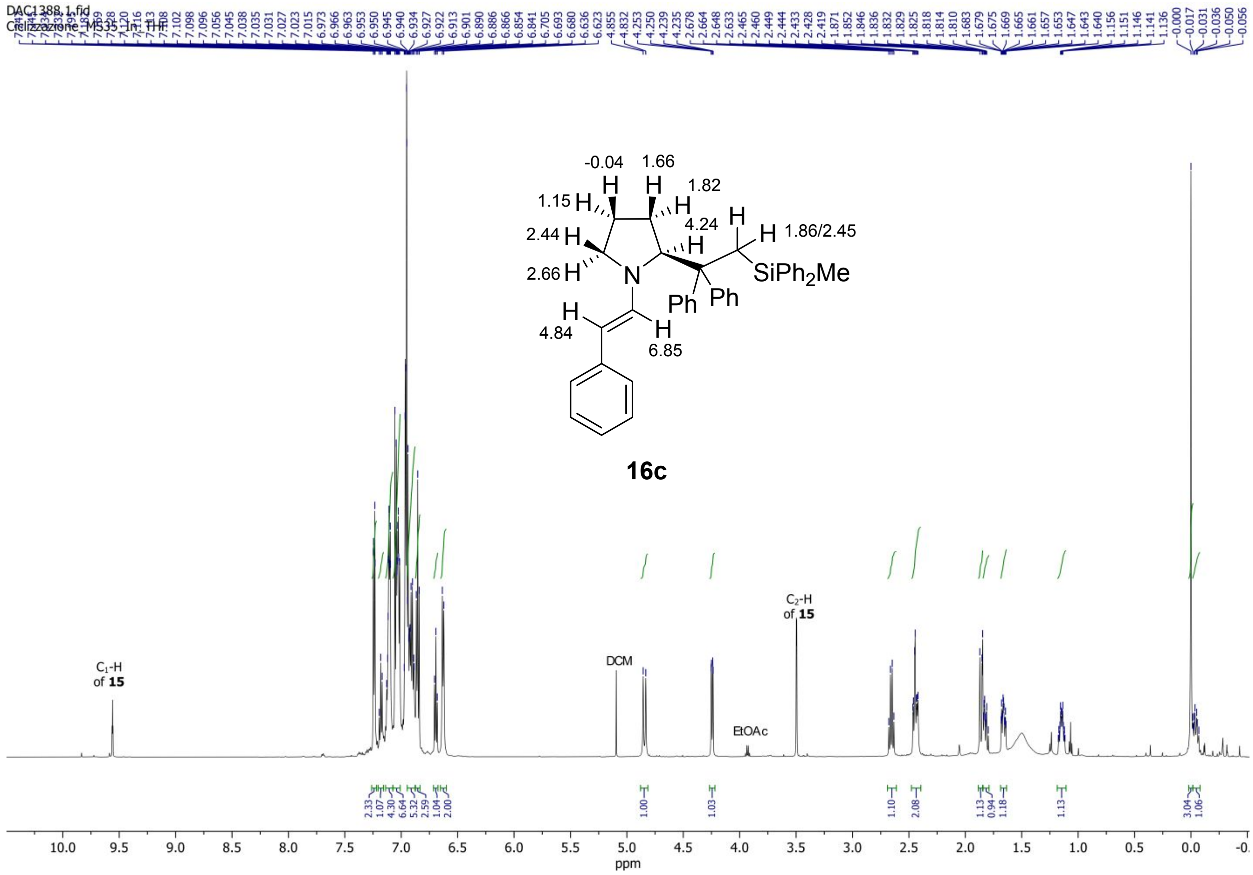

Figure S19 - ^{1}H NMR spectrum of (*S*) – **16c** recorded at 600MHz. The most relevant ^{1}H-NMR chemical shifts, determined through COSY and HSQC analyses, are reported in the corresponding molecule. The impurities unrelated to the product are marked in the spectrum. DCM = dichloromethane, EtOAc = Ethyl acetate

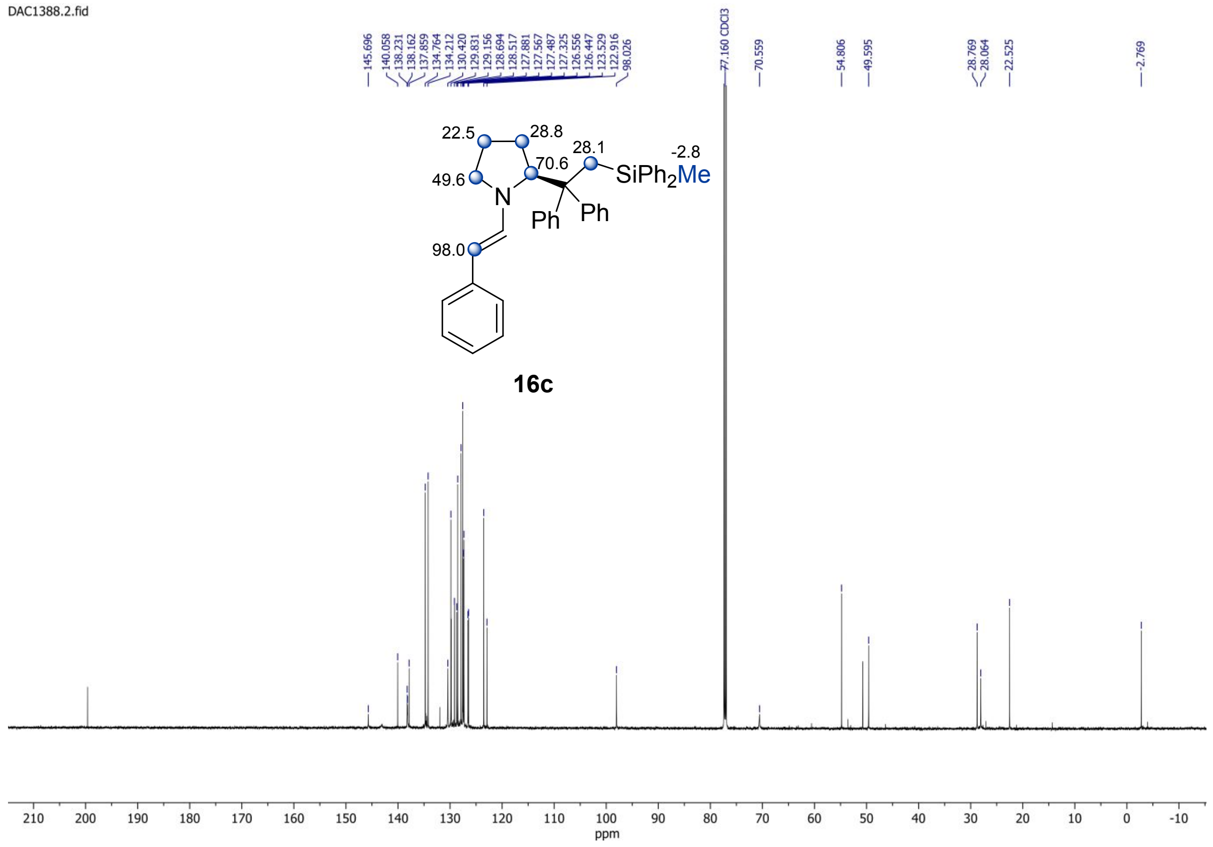

Figure S20 - $^{13}C\{^{1}H\}$ NMR spectrum of (*S*) - **16c** recorded at 150MHz. The most relevant ^{13}C-NMR chemical shifts, determined through HSQC analyses, are reported in the corresponding molecule.

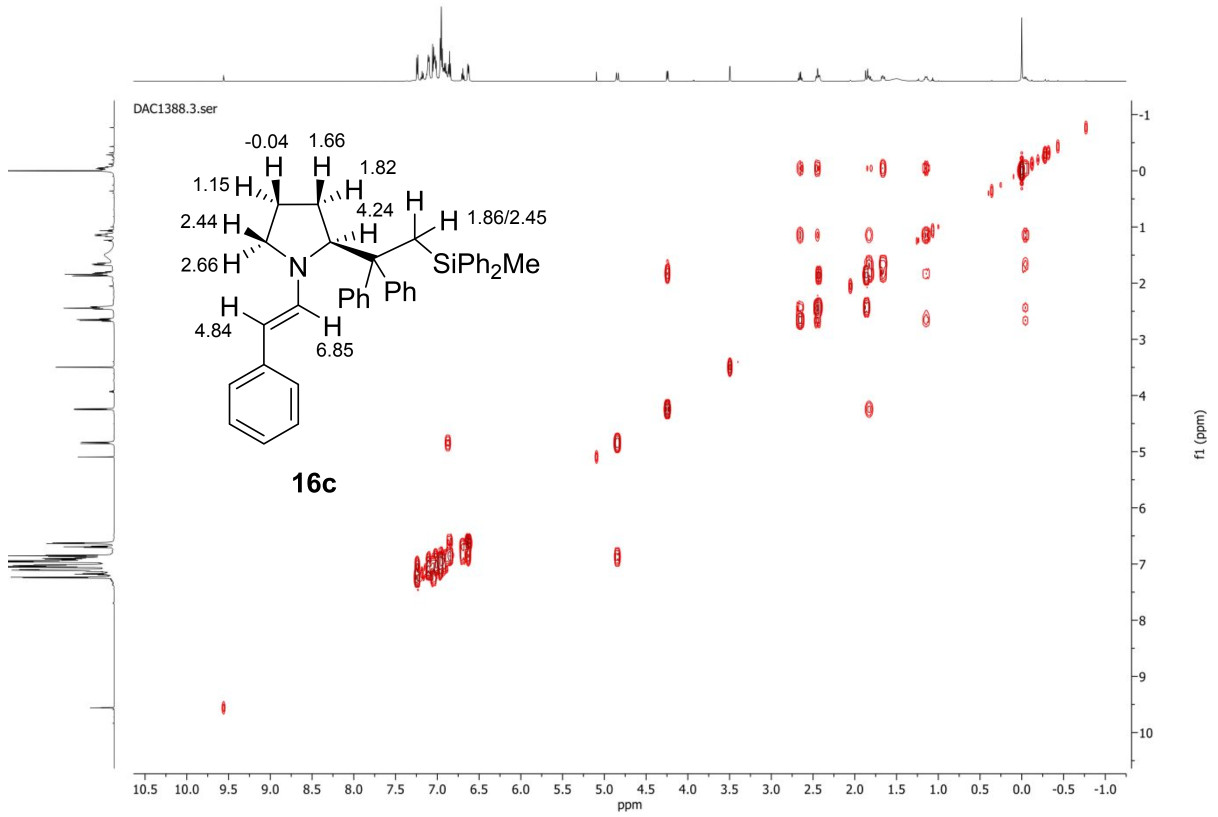

Figure S21 - ^{1}H – ^{1}H -COSY of (*S*) - **16c** recorded at 600 MHz.

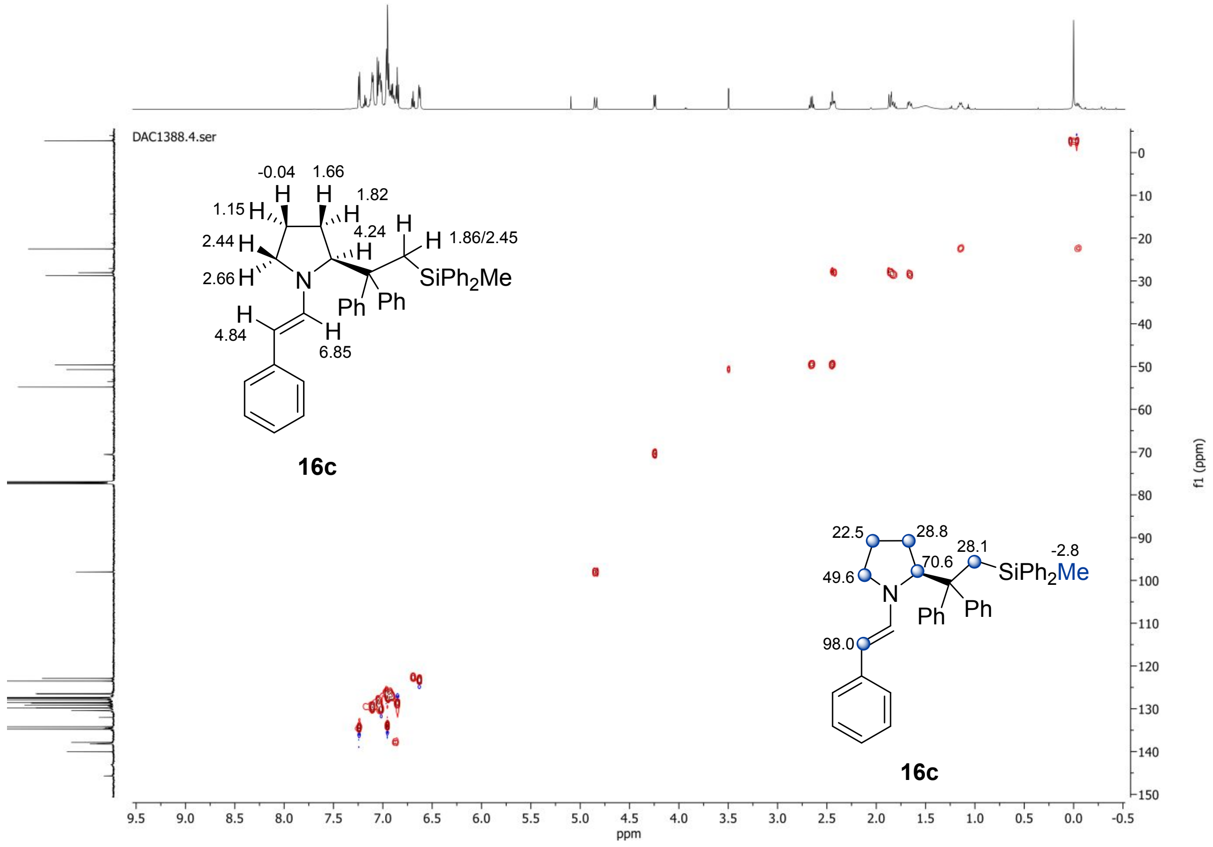

Figure S22 - ^{1}H – ^{13}C -HSQC of (*S*) - **16c** recorded at 600 MHz

NOESY

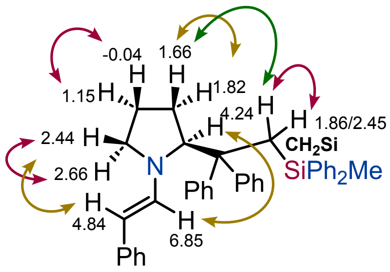

16c

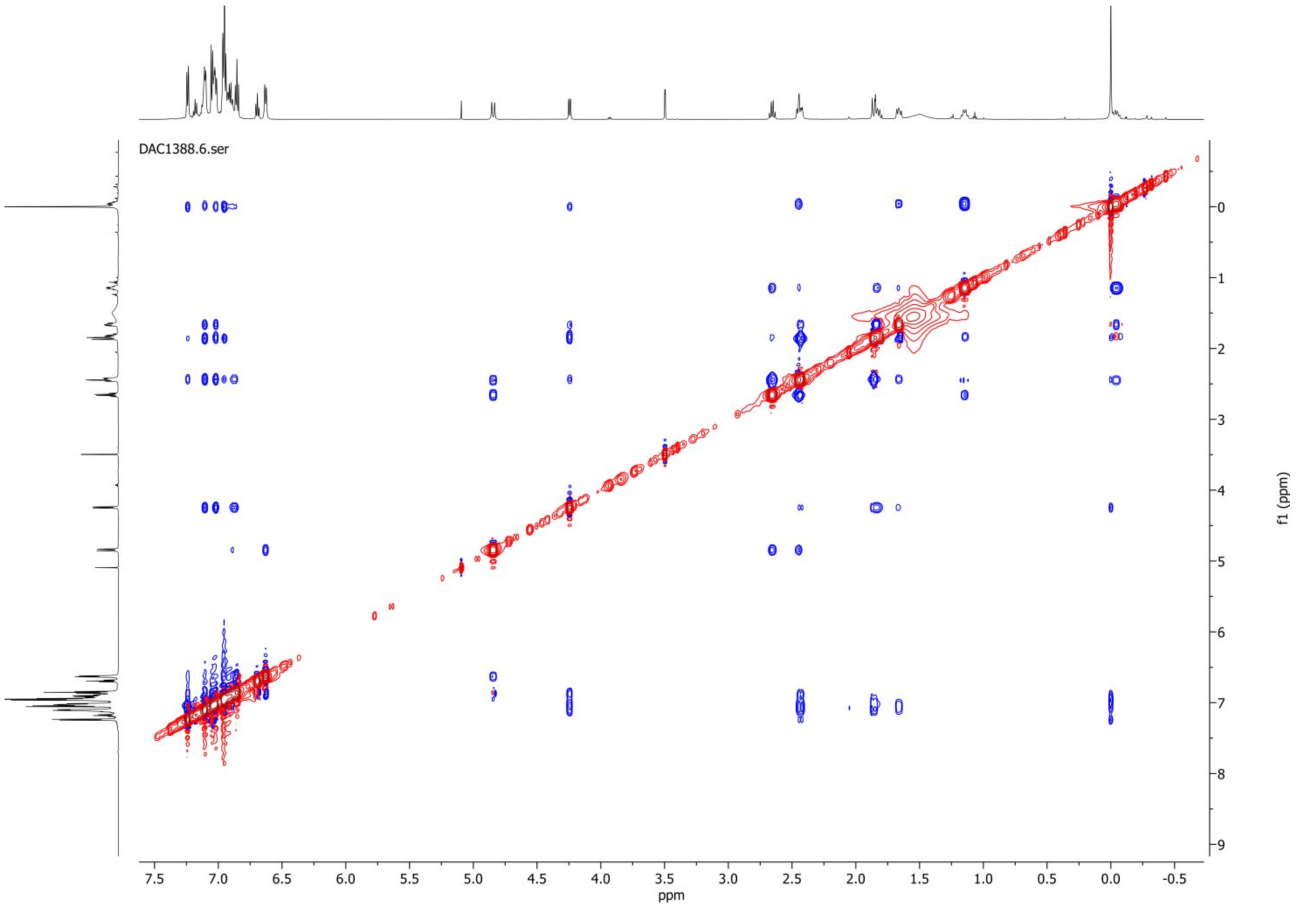

Figure S23 - 2D-NOESY of (*S*) - **16c** recorded at 600 MHz. The most important correlations are highlighted in the reported molecule.

The NMR studies on both enamines (*S*) - **16b-c** showed that both predominantly adopt an *ap* conformation (*antiperiplanar*). Indeed, as observed from the spectra reported before, a moderate NOE signal was observed between one of the hydrogens at C3 and one of the CH_2Si protons (green arrow), supporting the *ap* conformation in solution. Moreover, no NOE interaction is observed between the substituent on the silicon atom and the double bond protons, indicating that this group is pointing in another direction with respect to the enamine system.

7.5 Characterization of enamine **16d**

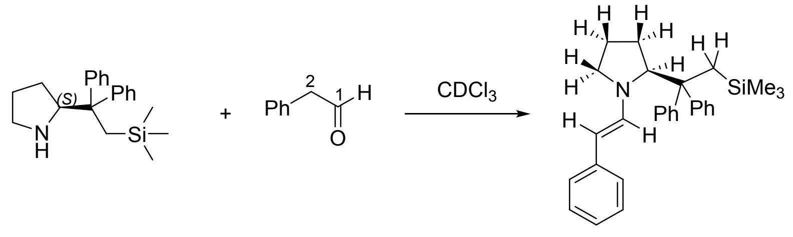

8d **15** **16d**

^{1}H NMR (600 MHz, $CDCl_3$) δ 7.37 (dd, *J* = 7.8, 1.8 Hz, 2H), 7.33 – 7.30 (m, 4H), 7.29 – 7.25 (m, 4H), 7.25 – 7.22 (m, 1H), 7.18 – 7.08 (m, 3H), 6.97 (d, *J* = 7.5 Hz, 2H), 6.92 (tt, *J* = 7.4, 1.3 Hz, 1H), 5.02 (d, *J* = 13.7 Hz, 1H), 4.50 (dd, *J* = 8.7, 2.1 Hz, 1H), 2.91 – 2.75 (m, 1H), 2.64 (td, *J* = 9.3, 3.0 Hz, 1H), 2.08 – 1.96 (m, 2H), 1.86 (ddt, *J* = 12.9, 8.0, 2.4 Hz, 1H), 1.44 – 1.33 (m, 1H), 1.29 (d, *J* = 14.9 Hz, 1H), 0.24 – 0.12 (m, 1H), -0.31 (s, 9H). **^{13}C{^{1}H} NMR** (150 MHz, $CDCl_3$) δ 146.3, 140.3, 137.9, 130.4, 129.8, 128.6, 127.6, 127.6, 126.9, 126.5, 126.3, 123.3, 122.9, 97.5, 70.3, 54.9, 49.4, 30.2, 28.6, 22.5, 0.4.

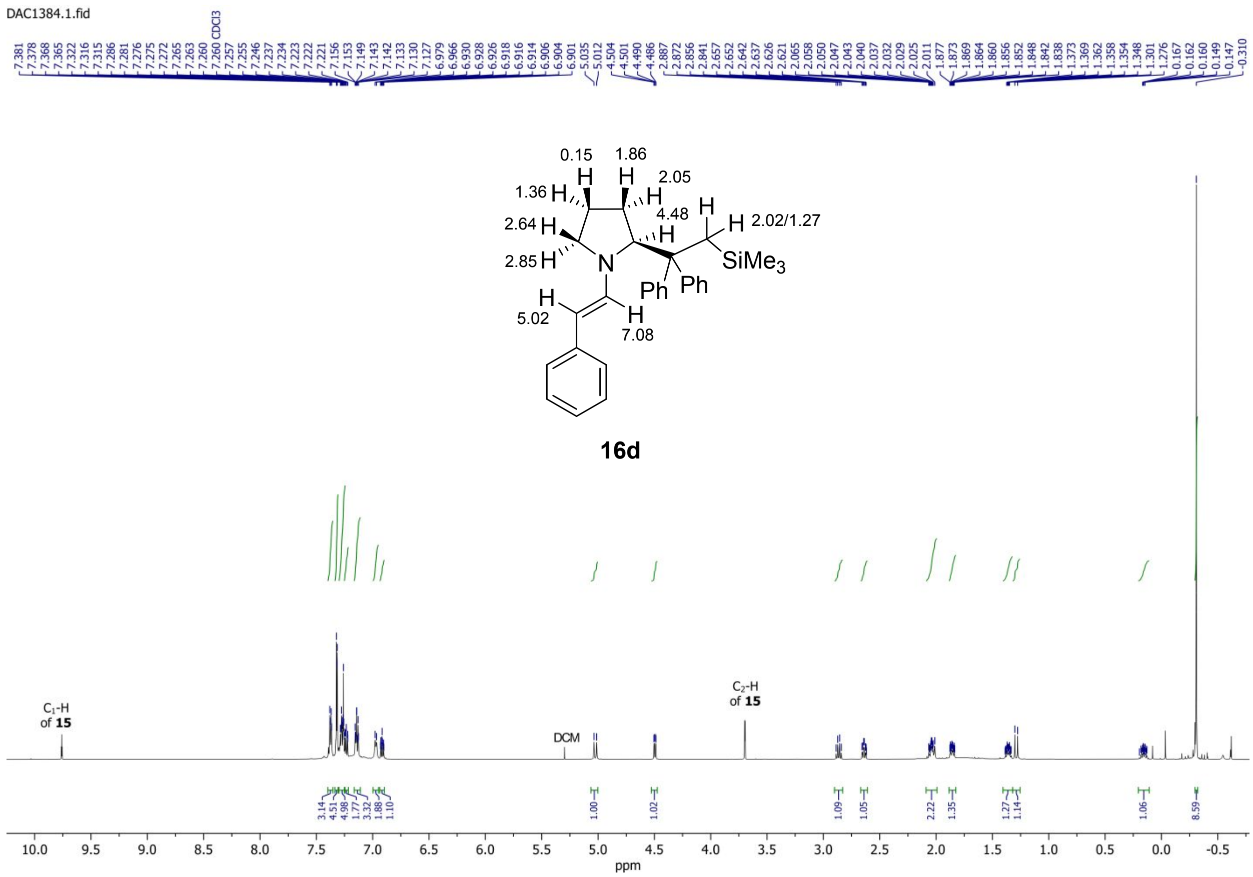

Figure S24 – ^{1}H NMR spectrum of (*S*) – **16d** recorded at 600 MHz. The most relevant ^{1}H-NMR chemical shifts, determined through COSY and HSQC analyses, are reported in the corresponding molecule. The impurities unrelated to the product are marked in the spectrum. DCM = dichloromethane.

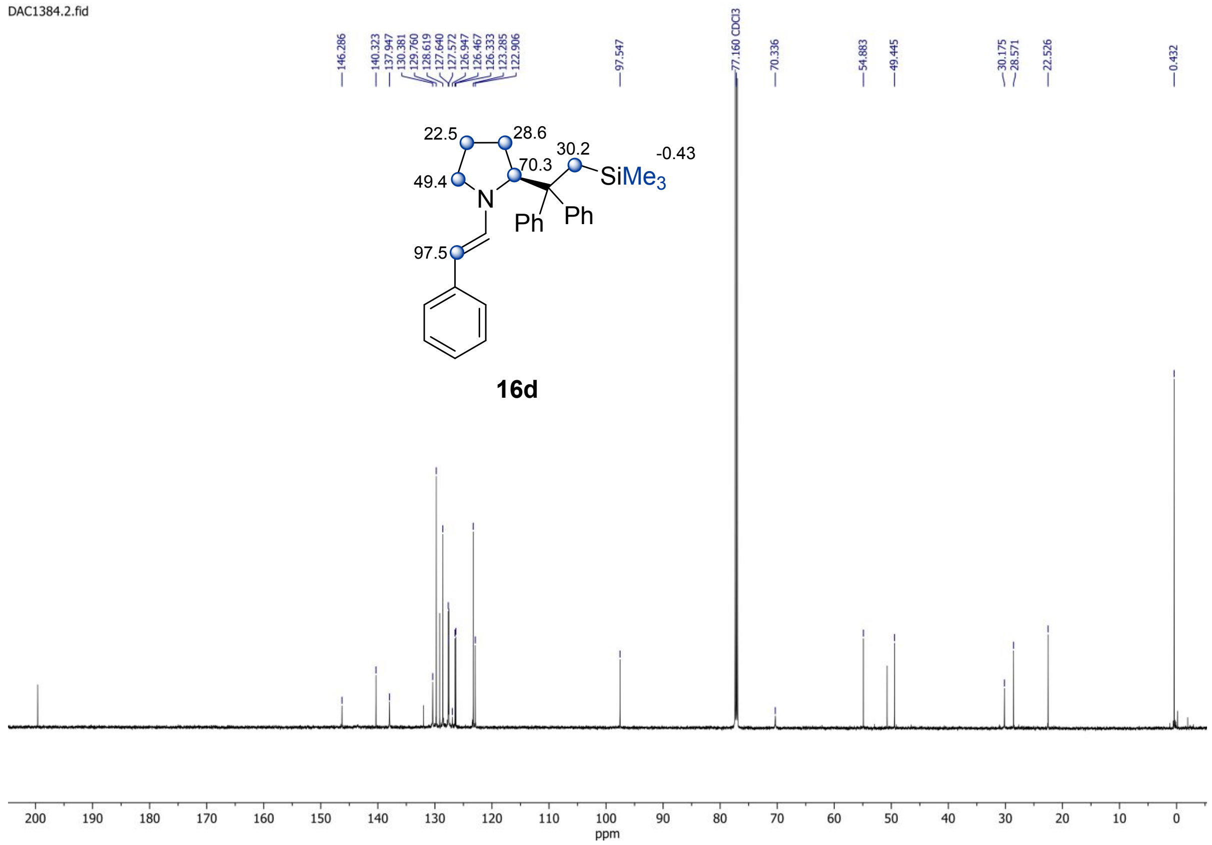

Figure S25 - $^{13}C\{^{1}H\}$ NMR spectrum of (*S*) - **16d** recorded at 150 MHz. The most relevant ^{13}C-NMR chemical shifts, determined through HSQC analyses, are reported in the corresponding molecule.

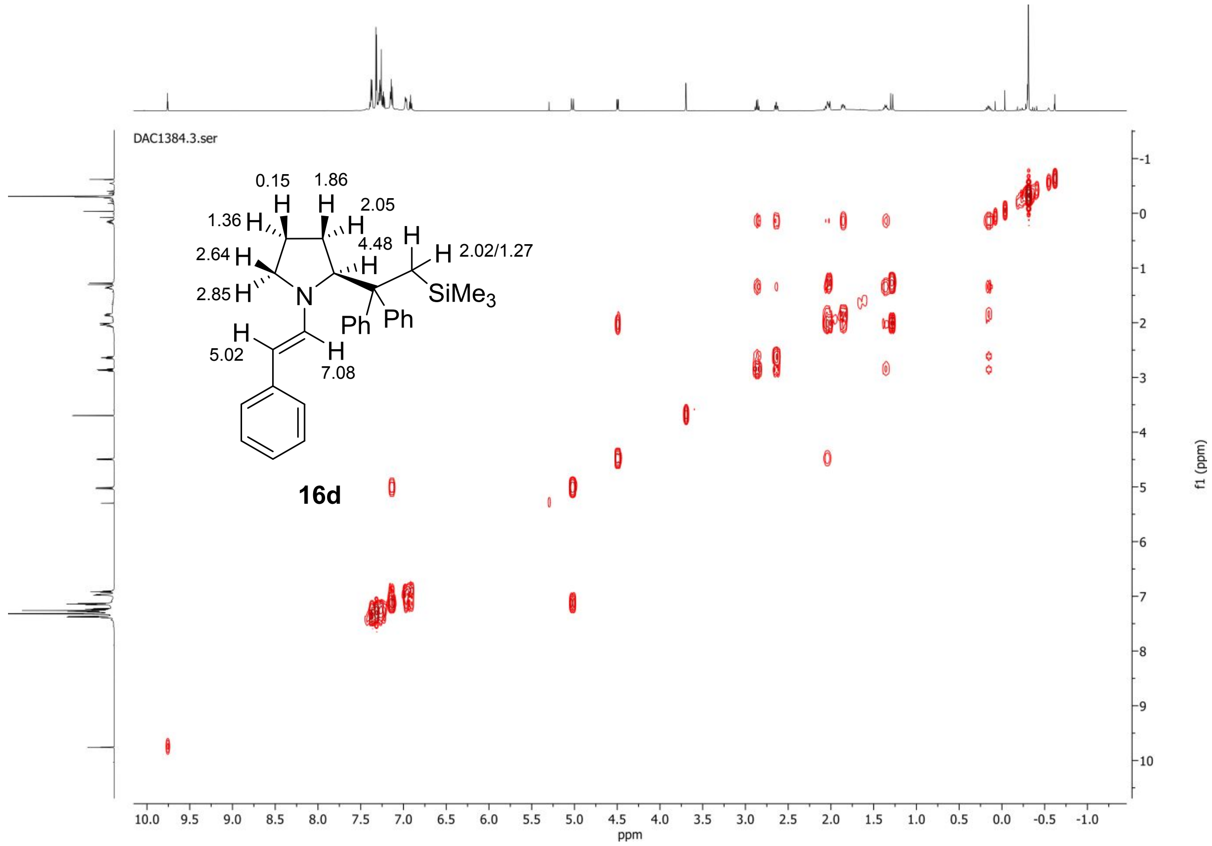

Figure S25 - ^{1}H – ^{1}H -COSY of (*S*) - **16d** recorded at 600 MHz.

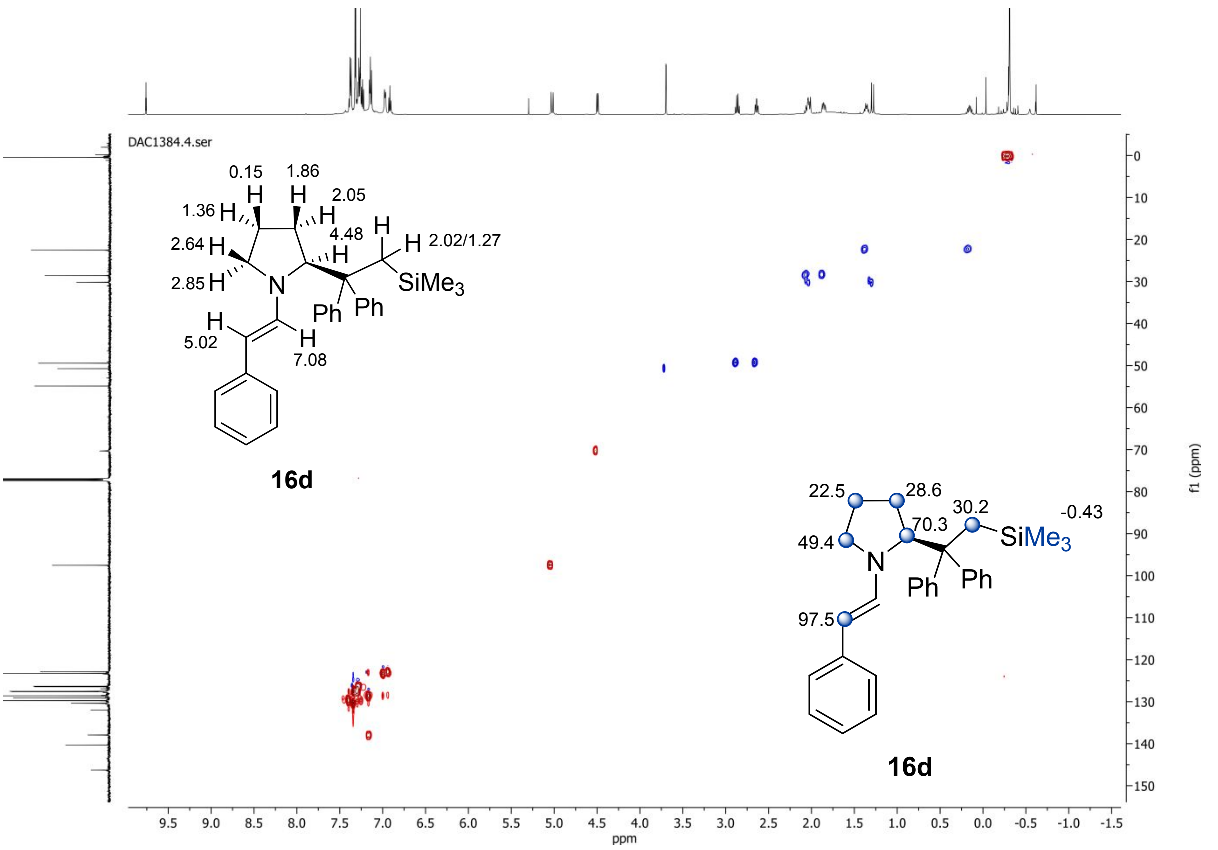

Figure S26 - ^{1}H – ^{13}C -HSQC of (*S*) - **16d** recorded at 600 MHz.

NOESY

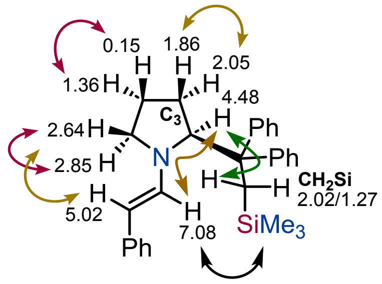

16d

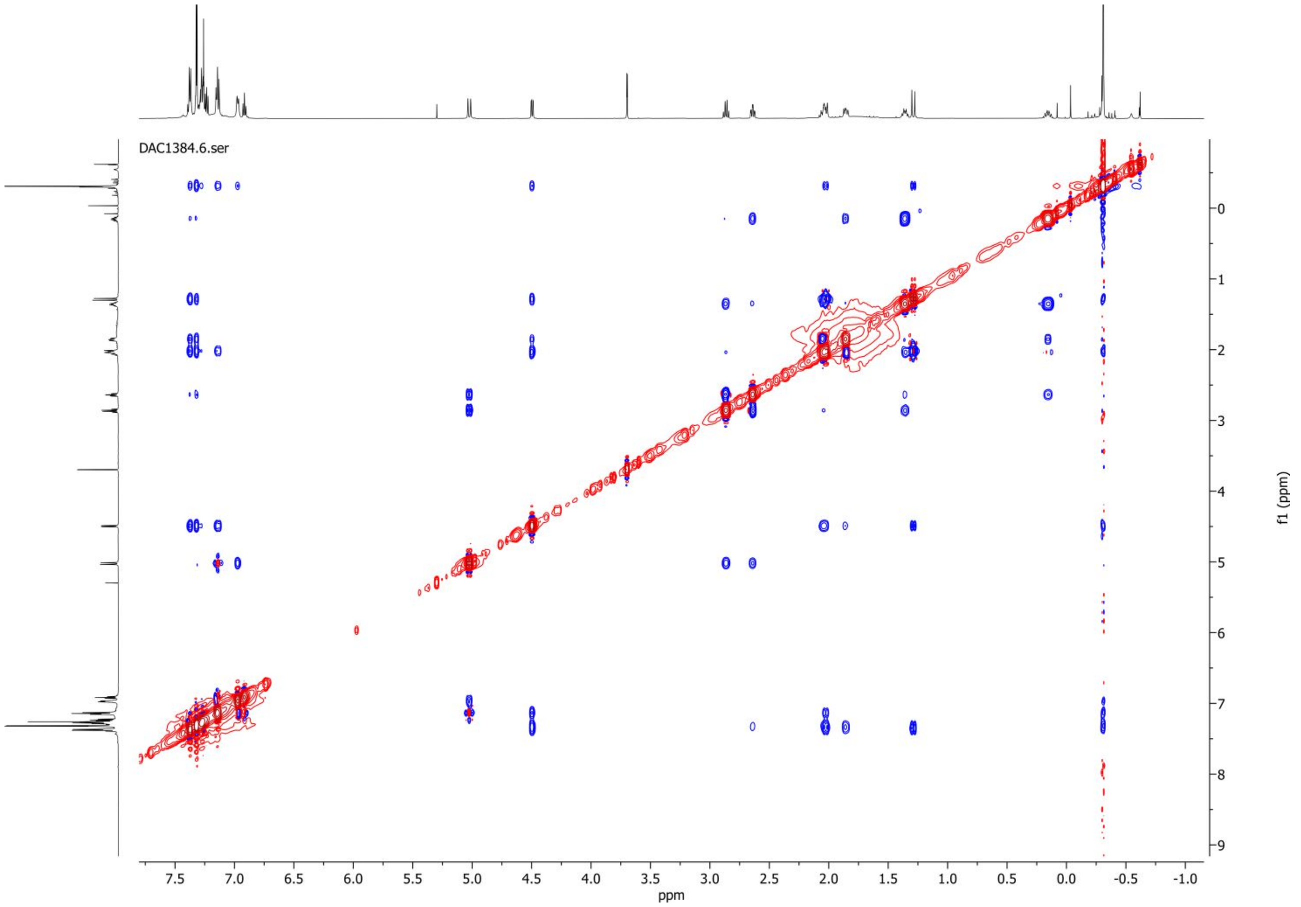

Figure S27 - 2D-NOESY of (*S*) - **16d** recorded at 600 MHz. The most important correlations are highlighted in the reported molecule.

The NMR analysis on enamine (*S*) – **16d** revealed that this enamine primarily adopts the *sc-exo* conformation in solution. Indeed, as observed from the previous spectra, a NOESY interaction between one of the CH_2Si protons and the proton at the C2 stereocenter (green arrow) is observed. Moreover, the absence of NOE responses with aliphatic protons on the pyrrolidine ring, in contrast to the two previous cases, further confirm this hypothesis. Finally, in this case a NOE interaction between the trimethylsilyl group and the double bond proton at 7.08 ppm is observed, further indicating that this group points in that direction.

7.6 DFT Calculations

Computational Details

Calculations were performed using Gaussian 16, Revision C.01.[8] MMFF conformational search was performed with the Spartan suite of programs, using the keywords SEARCHMETHOD=MC, FINDBOATS and KEEPALL. All the conformers within a 10 kcal·mol^{-1} window were then re-optimized using DFT (B3LYP/6-31g(d)). All molecule illustrations were made using CYLView.[9]

Enamine (S)-16b

```
                                   Gibbs Energies
--------------------------------------------------------------------------------
en_Conf15b.log: 1 G298 = -1469.90687900 au = -922380.49268 Kcal/mol
en_Conf11b.log: 2 G298 = -1469.90673400 au = -922380.40169 Kcal/mol
en_Conf1b.log : 3 G298 = -1469.90627600 au = -922380.11429 Kcal/mol
en_Conf18b.log: 4 G298 = -1469.90588000 au = -922379.86579 Kcal/mol
en_Conf16b.log: 5 G298 = -1469.90563500 au = -922379.71205 Kcal/mol
en_Conf2.log  : 6 G298 = -1469.90541600 au = -922379.57463 Kcal/mol
en_Conf8b.log : 7 G298 = -1469.90473500 au = -922379.14730 Kcal/mol
en_Conf4b.log : 8 G298 = -1469.90424500 au = -922378.83982 Kcal/mol
en_Conf3b.log : 9 G298 = -1469.90396200 au = -922378.66223 Kcal/mol
en_Conf13b.log: 10      G298 = -1469.90370500 au = -922378.50096 Kcal/mol
en_Conf6b.log : 11      G298 = -1469.90367800 au = -922378.48402 Kcal/mol
en_Conf9b.log : 12      G298 = -1469.90365000 au = -922378.46645 Kcal/mol
en_Conf7b.log : 13      G298 = -1469.90333300 au = -922378.26753 Kcal/mol
en_Conf5b.log : 14      G298 = -1469.90327500 au = -922378.23113 Kcal/mol
en_Conf17b.log: 15      G298 = -1469.90264500 au = -922377.83580 Kcal/mol
en_Conf19.log : 16      G298 = -1469.90194100 au = -922377.39403 Kcal/mol
en_Conf12b.log: 17      G298 = -1469.90135800 au = -922377.02820 Kcal/mol
en_Conf14b.log: 18      G298 = -1469.90085500 au = -922376.71256 Kcal/mol
--------------------------------------------------------------------------------
     G298 (Kcal/mol)  eexp(-Ei/KT)        Ni          Excess (%)      Sum (%)
     -------------- -------------- -------------- -------------- --------------
 1      0.00000        1.00000        0.27849         27.85           27.8
 2      0.09099        0.85764        0.23884         23.88           51.7
 3      0.37839        0.52801        0.14704         14.70           66.4
 4      0.62688        0.34713        0.09667          9.67           76.1
 5      0.78062        0.26779        0.07458          7.46           83.6
 6      0.91805        0.21236        0.05914          5.91           89.5
 7      1.34538        0.10324        0.02875          2.87           92.4
```

8	1.65286	0.06144	0.01711	1.71	94.1
9	1.83045	0.04553	0.01268	1.27	95.3
10	1.99172	0.03468	0.00966	0.97	96.3
11	2.00866	0.03370	0.00939	0.94	97.2
12	2.02623	0.03272	0.00911	0.91	98.1
13	2.22515	0.02339	0.00651	0.65	98.8
14	2.26154	0.02199	0.00612	0.61	99.4
15	2.65688	0.01128	0.00314	0.31	99.7
16	3.09864	0.00535	0.00149	0.15	99.9
17	3.46448	0.00289	0.00080	0.08	100.0
18	3.78012	0.00169	0.00047	0.05	100.0

en_Conf15b

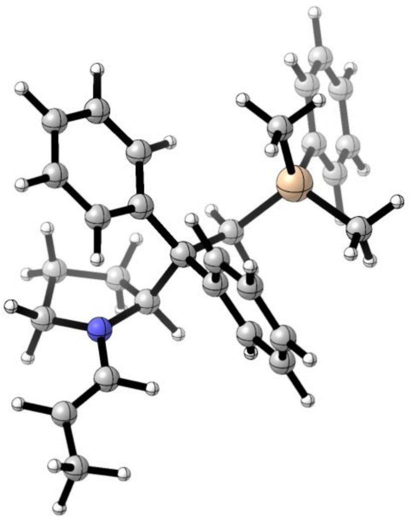

```
--------------------------------------------------------------------------------
   -- Stationary point found.
        Item               Value     Threshold  Converged?
Maximum Force            0.000000     0.000450     YES
RMS     Force            0.000000     0.000300     YES
Maximum Displacement     0.000060     0.001800     YES
RMS     Displacement     0.000011     0.001200     YES
Predicted change in Energy=-1.274357D-12
Optimization completed.

SCF Done:  E(RB3LYP) =  -1470.41028420     A.U. after    6 cycles

    Eigenvalues ---    0.00037   0.00065   0.00143   0.00152   0.00191

                        Standard orientation:
```

Center Number	Atomic Number	Atomic Type	Coordinates (Angstroms) X	Y	Z
1	6	0	0.940443	-1.836705	-2.129510
2	6	0	1.413205	-0.521061	-1.456622
3	7	0	2.833500	-0.781863	-1.201188
4	6	0	3.232234	-2.161160	-1.469389
5	6	0	1.915253	-2.921419	-1.644083
6	6	0	3.798324	0.213533	-1.230509
7	6	0	5.134273	0.039576	-1.248365
8	6	0	6.108223	1.184814	-1.269416
9	6	0	0.549477	-0.067756	-0.176683
10	6	0	0.641549	-1.191797	0.868004
11	6	0	0.926026	-3.379530	2.655181
12	6	0	-0.416282	-2.080472	1.113628
13	6	0	1.853099	-1.435501	1.546002
14	6	0	1.992677	-2.508173	2.426070
15	6	0	-0.279742	-3.158487	1.993454
16	6	0	1.060121	1.300746	0.349604
17	6	0	1.838435	3.880557	1.254639
18	6	0	1.277596	1.566745	1.709988
19	6	0	1.221308	2.379550	-0.540515
20	6	0	1.611115	3.644549	-0.102058
21	6	0	1.661251	2.834294	2.157146
22	6	0	-0.910863	0.178038	-0.681124
23	14	0	-2.287593	1.014040	0.382931
24	6	0	-3.912643	0.327129	-0.324782
25	6	0	-6.308143	-0.711706	-1.423049
26	6	0	-4.308715	0.629301	-1.642388
27	6	0	-4.758690	-0.511573	0.423252
28	6	0	-5.941075	-1.026193	-0.114442
29	6	0	-5.487418	0.119885	-2.188104
30	6	0	-2.240164	0.674877	2.244152
31	6	0	-2.276108	2.890171	0.122440
32	1	0	-0.099222	-2.088661	-1.913399
33	1	0	1.025104	-1.718816	-3.216407
34	1	0	1.318766	0.298189	-2.179463

35	1	0	3.840849	-2.560235	-0.647161
36	1	0	3.852726	-2.199398	-2.380926
37	1	0	1.582960	-3.339972	-0.690705
38	1	0	2.009823	-3.746213	-2.357446
39	1	0	3.394994	1.221416	-1.221093
40	1	0	5.557997	-0.962229	-1.234529
41	1	0	6.776934	1.173320	-0.396179
42	1	0	6.756808	1.158321	-2.157371
43	1	0	5.588603	2.150296	-1.270678
44	1	0	1.034208	-4.216013	3.340532
45	1	0	-1.368592	-1.951095	0.612599
46	1	0	2.699201	-0.779690	1.378876
47	1	0	2.942053	-2.662450	2.932806
48	1	0	-1.123562	-3.824328	2.155942
49	1	0	2.142283	4.864739	1.600573
50	1	0	1.145870	0.777499	2.440343
51	1	0	1.034185	2.243339	-1.601438
52	1	0	1.730342	4.447836	-0.824703
53	1	0	1.821978	2.996500	3.219951
54	1	0	-0.843187	0.817394	-1.573674
55	1	0	-1.341458	-0.756865	-1.052944
56	1	0	-7.228035	-1.109474	-1.844201
57	1	0	-3.689290	1.279113	-2.258571
58	1	0	-4.496564	-0.768007	1.447197
59	1	0	-6.575630	-1.670512	0.489408
60	1	0	-5.767511	0.372651	-3.207935
61	1	0	-3.117967	1.129538	2.720312
62	1	0	-2.230431	-0.389760	2.495641
63	1	0	-1.350660	1.127127	2.693956
64	1	0	-3.137687	3.346095	0.625073
65	1	0	-1.366506	3.345875	0.527199
66	1	0	-2.336176	3.157567	-0.939431

Alpha occ. eigenvalues --   -0.24478 -0.24464 -0.24189 -0.23373 -0.17100

Alpha virt. eigenvalues --   -0.00719 -0.00644 -0.00362   0.00057   0.00402

LUMO E: -0.00719 au = -0.196 eV

HOMO E: -0.17100 au = -4.653 eV

DELTA E: +0.16381 au = +4.457 eV

```
 Zero-point correction=                           0.567075 (Hartree/Particle)
 Thermal correction to Energy=                    0.597867
 Thermal correction to Enthalpy=                  0.598811
 Thermal correction to Gibbs Free Energy=         0.503405
 Sum of electronic and zero-point Energies=          -1469.843210
 Sum of electronic and thermal Energies=             -1469.812417
 Sum of electronic and thermal Enthalpies=           -1469.811473
 Sum of electronic and thermal Free Energies=        -1469.906879
```

en_Conf11b

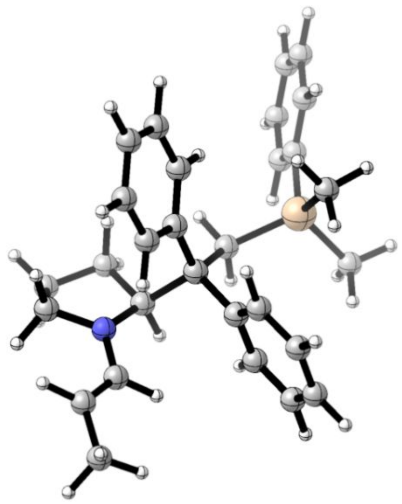

```
--------------------------------------------------------------------------------
    -- Stationary point found.
         Item               Value     Threshold  Converged?
 Maximum Force            0.000000     0.000450     YES
 RMS     Force            0.000000     0.000300     YES
 Maximum Displacement     0.000099     0.001800     YES
 RMS     Displacement     0.000014     0.001200     YES
 Predicted change in Energy=-1.381994D-12
 Optimization completed.

 SCF Done:  E(RB3LYP) =  -1470.41029218     A.U. after    6 cycles

     Eigenvalues ---    0.00053   0.00070   0.00138   0.00165   0.00214

                         Standard orientation:
```

Center Number	Atomic Number	Atomic Type	Coordinates (Angstroms) X	Y	Z
1	6	0	0.762629	-1.994508	-1.997258
2	6	0	1.405438	-0.691814	-1.453037
3	7	0	2.776048	-1.098480	-1.139351
4	6	0	3.027567	-2.511968	-1.400518
5	6	0	1.944530	-2.877465	-2.420954
6	6	0	3.809619	-0.203407	-0.930653
7	6	0	5.071171	-0.493623	-0.554067
8	6	0	6.144950	0.549299	-0.413761
9	6	0	0.558584	-0.028576	-0.258234
10	6	0	0.532752	-1.017289	0.921503
11	6	0	0.556301	-2.945313	3.007960
12	6	0	1.716078	-1.331080	1.620370
13	6	0	-0.631106	-1.705024	1.300663
14	6	0	-0.623614	-2.652435	2.329042
15	6	0	1.727068	-2.277028	2.645508
16	6	0	1.158234	1.353484	0.115289
17	6	0	2.163354	3.943358	0.716504
18	6	0	1.348595	2.324389	-0.887193
19	6	0	1.467557	1.733314	1.429775
20	6	0	1.963789	3.005709	1.727144
21	6	0	1.847146	3.594336	-0.597343
22	6	0	-0.865422	0.234380	-0.851305
23	14	0	-2.232853	1.260254	0.050993
24	6	0	-3.857640	0.326717	-0.270705
25	6	0	-6.262509	-1.075971	-0.793542
26	6	0	-4.650856	-0.182513	0.773297
27	6	0	-4.310890	0.109332	-1.586889
28	6	0	-5.494598	-0.580464	-1.849800
29	6	0	-5.837665	-0.875224	0.519804
30	6	0	-2.034786	1.513322	1.914962
31	6	0	-2.347581	2.962508	-0.774858
32	1	0	0.198631	-2.494428	-1.203197
33	1	0	0.069847	-1.792428	-2.818478
34	1	0	1.428860	0.061250	-2.254184

35	1	0	2.945717	-3.120271	-0.486377
36	1	0	4.042509	-2.636411	-1.795146
37	1	0	1.697210	-3.944088	-2.421060
38	1	0	2.284386	-2.609551	-3.428571
39	1	0	3.532099	0.834367	-1.097240
40	1	0	5.353971	-1.522439	-0.338534
41	1	0	5.749643	1.554696	-0.600839
42	1	0	6.585987	0.550429	0.593508
43	1	0	6.976015	0.387529	-1.116352
44	1	0	0.565652	-3.681514	3.807409
45	1	0	2.641900	-0.828153	1.367270
46	1	0	-1.570535	-1.517770	0.793123
47	1	0	-1.548173	-3.160453	2.591245
48	1	0	2.659235	-2.489266	3.163202
49	1	0	2.553014	4.931199	0.946712
50	1	0	1.104924	2.095320	-1.920294
51	1	0	1.317223	1.032742	2.241923
52	1	0	2.193525	3.257951	2.759280
53	1	0	1.985052	4.311473	-1.402639
54	1	0	-0.724579	0.756941	-1.807817
55	1	0	-1.323456	-0.721388	-1.126437
56	1	0	-7.185781	-1.613635	-0.994174
57	1	0	-4.341933	-0.037321	1.805812
58	1	0	-3.731832	0.487259	-2.428231
59	1	0	-5.819439	-0.730706	-2.876585
60	1	0	-6.429831	-1.256647	1.348212
61	1	0	-2.921435	2.024219	2.311031
62	1	0	-1.907545	0.576614	2.466316
63	1	0	-1.166825	2.144489	2.128084
64	1	0	-3.157572	3.555242	-0.333544
65	1	0	-1.412800	3.521323	-0.651966
66	1	0	-2.548213	2.880541	-1.850139

Alpha occ. eigenvalues --   -0.24336 -0.24273 -0.24156 -0.23428 -0.17006

Alpha virt. eigenvalues --   -0.00716 -0.00646 -0.00334   0.00143   0.00436

LUMO E: -0.00716 au = -0.195 eV

```
HOMO  E: -0.17006 au = -4.628 eV
DELTA E: +0.16290 au = +4.433 eV

 Zero-point correction=                           0.567098 (Hartree/Particle)
 Thermal correction to Energy=                    0.597895
 Thermal correction to Enthalpy=                  0.598839
 Thermal correction to Gibbs Free Energy=         0.503558
 Sum of electronic and zero-point Energies=          -1469.843194
 Sum of electronic and thermal Energies=             -1469.812397
 Sum of electronic and thermal Enthalpies=           -1469.811453
 Sum of electronic and thermal Free Energies=        -1469.906734
```

en_Conf1b

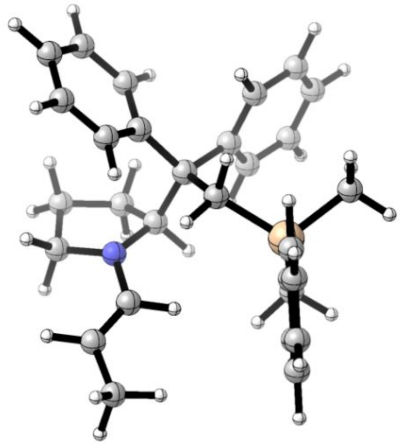

```
--------------------------------------------------------------------------------
    -- Stationary point found.
         Item               Value     Threshold  Converged?
 Maximum Force            0.000000     0.000450     YES
 RMS     Force            0.000000     0.000300     YES
 Maximum Displacement     0.000021     0.001800     YES
 RMS     Displacement     0.000005     0.001200     YES
 Predicted change in Energy=-1.478475D-12
 Optimization completed.

 SCF Done:  E(RB3LYP) =  -1470.41075003     A.U. after    6 cycles

    Eigenvalues ---    0.00066   0.00119   0.00165   0.00200   0.00224

                         Standard orientation:
 ---------------------------------------------------------------------
```

Center Number	Atomic Number	Atomic Type	Coordinates (Angstroms) X	Y	Z
1	6	0	-2.475780	-1.143547	-1.916523
2	6	0	-1.102717	-0.793454	-1.286522
3	7	0	-0.507301	-2.115954	-1.034275
4	6	0	-1.445572	-3.219329	-1.241501
5	6	0	-2.815442	-2.553165	-1.402685
6	6	0	0.845115	-2.357367	-1.234933
7	6	0	1.456168	-3.555927	-1.296375
8	6	0	2.937328	-3.711777	-1.502661
9	6	0	-1.168541	0.139820	-0.000587
10	6	0	-1.733736	1.538648	-0.337969
11	6	0	-2.655741	4.177612	-0.828429
12	6	0	-1.937759	2.010536	-1.643248
13	6	0	-2.005627	2.432097	0.715296
14	6	0	-2.461266	3.726887	0.479582
15	6	0	-2.390038	3.312174	-1.886546
16	6	0	-2.046769	-0.556834	1.067419
17	6	0	-3.676580	-1.944760	2.928140
18	6	0	-1.514473	-1.548170	1.908964
19	6	0	-3.420312	-0.282569	1.186991
20	6	0	-4.224759	-0.963361	2.102341
21	6	0	-2.315735	-2.232405	2.824927
22	6	0	0.282339	0.353245	0.555070
23	14	0	1.606553	1.488015	-0.264534
24	6	0	3.257065	0.874181	0.449392
25	6	0	5.701690	-0.035498	1.553750
26	6	0	4.298138	0.397407	-0.367686
27	6	0	3.483288	0.881692	1.839614
28	6	0	4.685577	0.434623	2.388375
29	6	0	5.505479	-0.052578	0.172774
30	6	0	1.710651	1.436502	-2.158472
31	6	0	1.385619	3.297057	0.250194
32	1	0	-3.253874	-0.415432	-1.677941
33	1	0	-2.367209	-1.160818	-3.008037
34	1	0	-0.477968	-0.266877	-2.015610
35	1	0	-1.418826	-3.923636	-0.400075

36	1	0	-1.156590	-3.780634	-2.145714
37	1	0	-3.333835	-2.499158	-0.442833
38	1	0	-3.456271	-3.104836	-2.097831
39	1	0	1.445015	-1.457978	-1.340662
40	1	0	0.879868	-4.470221	-1.172802
41	1	0	3.437805	-2.738037	-1.560681
42	1	0	3.407537	-4.270716	-0.680811
43	1	0	3.170627	-4.261309	-2.426525
44	1	0	-3.011192	5.186984	-1.017008
45	1	0	-1.750778	1.369801	-2.497229
46	1	0	-1.867721	2.100631	1.740323
47	1	0	-2.663165	4.385770	1.320274
48	1	0	-2.537726	3.641185	-2.912025
49	1	0	-4.299668	-2.474411	3.643990
50	1	0	-0.463025	-1.801504	1.850210
51	1	0	-3.873112	0.479652	0.561860
52	1	0	-5.282518	-0.720881	2.168400
53	1	0	-1.868820	-2.992847	3.460481
54	1	0	0.190648	0.757520	1.573587
55	1	0	0.760703	-0.621816	0.684526
56	1	0	6.639688	-0.385000	1.977696
57	1	0	4.172035	0.377134	-1.447730
58	1	0	2.708389	1.244666	2.512949
59	1	0	4.830522	0.452911	3.465859
60	1	0	6.291755	-0.415085	-0.484977
61	1	0	2.545655	2.064834	-2.493232
62	1	0	1.868094	0.434297	-2.571416
63	1	0	0.799520	1.846045	-2.607199
64	1	0	2.244339	3.888017	-0.091094
65	1	0	0.478523	3.735920	-0.177134
66	1	0	1.325282	3.404853	1.339665

Alpha occ. eigenvalues --   -0.24326 -0.24233 -0.24023 -0.23099 -0.17353

Alpha virt. eigenvalues --   -0.00768 -0.00552 -0.00304   0.00345   0.00550

LUMO E: -0.00768 au = -0.209 eV

HOMO E: -0.17353 au = -4.722 eV

```
DELTA E: +0.16585 au = +4.513 eV

 Zero-point correction=                           0.567095 (Hartree/Particle)
 Thermal correction to Energy=                    0.597855
 Thermal correction to Enthalpy=                  0.598799
 Thermal correction to Gibbs Free Energy=         0.504474
 Sum of electronic and zero-point Energies=          -1469.843655
 Sum of electronic and thermal Energies=             -1469.812895
 Sum of electronic and thermal Enthalpies=           -1469.811951
 Sum of electronic and thermal Free Energies=        -1469.906276
```

en_Conf18b

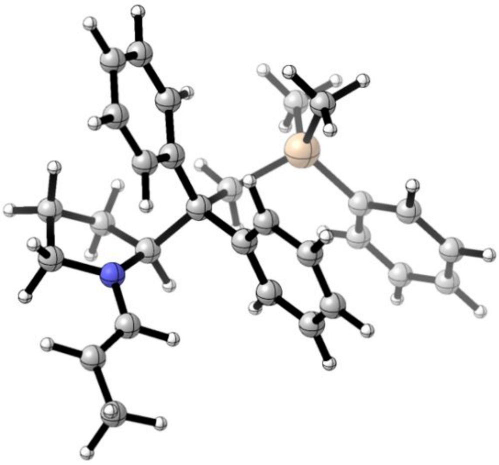

```
--------------------------------------------------------------------------------
    -- Stationary point found.
         Item               Value     Threshold  Converged?
 Maximum Force            0.000000     0.000450     YES
 RMS     Force            0.000000     0.000300     YES
 Maximum Displacement     0.000041     0.001800     YES
 RMS     Displacement     0.000008     0.001200     YES
 Predicted change in Energy=-7.474943D-13
 Optimization completed.

 SCF Done:  E(RB3LYP) =  -1470.40927217     A.U. after    7 cycles

    Eigenvalues ---    0.00071   0.00094   0.00126   0.00170   0.00204

                         Standard orientation:
 ---------------------------------------------------------------------
 Center     Atomic      Atomic             Coordinates (Angstroms)
```

Number	Number	Type	X	Y	Z
1	6	0	2.158306	-0.744046	-2.491873
2	6	0	1.578849	0.270556	-1.471295
3	7	0	2.777436	0.962536	-0.988264
4	6	0	4.025486	0.367477	-1.458103
5	6	0	3.613206	-0.980031	-2.056062
6	6	0	2.769231	2.294546	-0.602143
7	6	0	3.845292	3.073724	-0.378419
8	6	0	3.739763	4.511075	0.049850
9	6	0	0.637746	-0.374449	-0.335153
10	6	0	1.471053	-1.405330	0.444502
11	6	0	3.150292	-3.278160	1.765364
12	6	0	1.274839	-2.788649	0.313925
13	6	0	2.541994	-0.987470	1.261207
14	6	0	3.365406	-1.906290	1.911102
15	6	0	2.098543	-3.713572	0.962787
16	6	0	0.063325	0.755613	0.562830
17	6	0	-1.107665	2.820929	2.124479
18	6	0	-0.624429	1.831932	-0.029658
19	6	0	0.118360	0.736168	1.964577
20	6	0	-0.454728	1.752513	2.734384
21	6	0	-1.194772	2.850342	0.732329
22	6	0	-0.577850	-1.016154	-1.081553
23	14	0	-2.172314	-1.639571	-0.193125
24	6	0	-3.530680	-0.324153	-0.344289
25	6	0	-5.580992	1.608415	-0.619996
26	6	0	-4.152835	0.246955	0.779403
27	6	0	-3.971594	0.102944	-1.611784
28	6	0	-4.980581	1.055948	-1.753500
29	6	0	-5.164618	1.200889	0.647610
30	6	0	-2.729762	-3.158135	-1.190230
31	6	0	-1.991964	-2.154139	1.617681
32	1	0	1.589878	-1.673815	-2.553375
33	1	0	2.136863	-0.284926	-3.487496
34	1	0	0.938692	0.984920	-2.003105
35	1	0	4.743209	0.258560	-0.634057
36	1	0	4.493169	1.025100	-2.210592

37	1	0	3.665521	-1.768007	-1.300289
38	1	0	4.257371	-1.272511	-2.891304
39	1	0	1.774945	2.707823	-0.462131
40	1	0	4.849028	2.669779	-0.491911
41	1	0	4.226439	4.688640	1.020145
42	1	0	4.220839	5.191605	-0.667980
43	1	0	2.692962	4.822105	0.147204
44	1	0	3.791550	-3.994546	2.271967
45	1	0	0.474024	-3.171362	-0.307900
46	1	0	2.736172	0.070957	1.385078
47	1	0	4.179702	-1.544866	2.534173
48	1	0	1.911136	-4.776721	0.834765
49	1	0	-1.550099	3.613342	2.722059
50	1	0	-0.731811	1.882809	-1.108816
51	1	0	0.609856	-0.083358	2.474406
52	1	0	-0.384821	1.701058	3.818099
53	1	0	-1.717182	3.662062	0.233162
54	1	0	-0.947724	-0.272212	-1.800888
55	1	0	-0.232581	-1.846723	-1.706654
56	1	0	-6.368789	2.350174	-0.725402
57	1	0	-3.841278	-0.049238	1.777757
58	1	0	-3.523164	-0.316159	-2.511653
59	1	0	-5.300771	1.365324	-2.745598
60	1	0	-5.626265	1.626372	1.535389
61	1	0	-3.681191	-3.548048	-0.810005
62	1	0	-2.873140	-2.920114	-2.251245
63	1	0	-1.992147	-3.968836	-1.132144
64	1	0	-2.938398	-2.577536	1.976592
65	1	0	-1.217440	-2.918372	1.738629
66	1	0	-1.729521	-1.313731	2.266875

Alpha occ. eigenvalues -- -0.24357 -0.24178 -0.23816 -0.23193 -0.17045

Alpha virt. eigenvalues -- -0.00856 -0.00283 -0.00092 0.00158 0.00563

LUMO E: -0.00856 au = -0.233 eV

HOMO E: -0.17045 au = -4.638 eV

DELTA E: +0.16189 au = +4.405 eV

```
Zero-point correction=                           0.566879 (Hartree/Particle)
Thermal correction to Energy=                    0.597726
Thermal correction to Enthalpy=                  0.598670
Thermal correction to Gibbs Free Energy=         0.503392
Sum of electronic and zero-point Energies=          -1469.842393
Sum of electronic and thermal Energies=             -1469.811547
Sum of electronic and thermal Enthalpies=           -1469.810602
Sum of electronic and thermal Free Energies=        -1469.905880
```

en_Conf16b

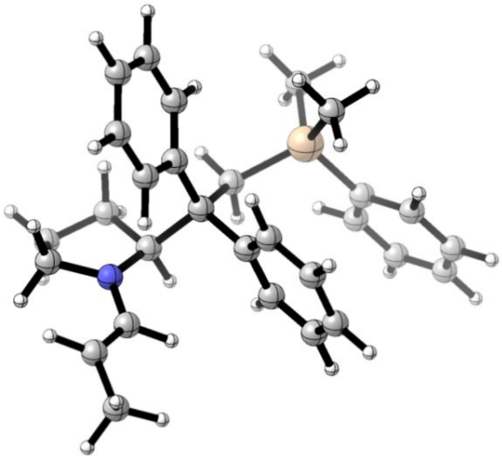

```
--------------------------------------------------------------------------------
   -- Stationary point found.
         Item               Value     Threshold  Converged?
Maximum Force            0.000000     0.000450     YES
RMS     Force            0.000000     0.000300     YES
Maximum Displacement     0.000061     0.001800     YES
RMS     Displacement     0.000013     0.001200     YES
Predicted change in Energy=-4.696444D-12
Optimization completed.

SCF Done:  E(RB3LYP) =  -1470.40930929     A.U. after    6 cycles

    Eigenvalues ---    0.00084   0.00127   0.00136   0.00171   0.00214

                        Standard orientation:
---------------------------------------------------------------------
Center     Atomic      Atomic             Coordinates (Angstroms)
Number     Number       Type             X           Y           Z
```

1	6	0	-1.929371	-0.957991	2.518170
2	6	0	-1.510084	0.166954	1.535980
3	7	0	-2.780903	0.810793	1.200604
4	6	0	-3.928721	0.185047	1.848595
5	6	0	-3.304761	-0.532532	3.050174
6	6	0	-2.871995	2.057415	0.607971
7	6	0	-3.994443	2.678096	0.192817
8	6	0	-3.996854	4.065220	-0.387080
9	6	0	-0.624744	-0.374778	0.308432
10	6	0	-1.480738	-1.358994	-0.510397
11	6	0	-3.168067	-3.160858	-1.919278
12	6	0	-1.221420	-2.738064	-0.544021
13	6	0	-2.621334	-0.909030	-1.206472
14	6	0	-3.448974	-1.793620	-1.897962
15	6	0	-2.047104	-3.627708	-1.238123
16	6	0	-0.098757	0.818245	-0.533730
17	6	0	0.965717	3.005342	-2.000875
18	6	0	0.619721	1.851625	0.097391
19	6	0	-0.242096	0.905195	-1.926687
20	6	0	0.278935	1.981661	-2.649878
21	6	0	1.138706	2.929588	-0.618447
22	6	0	0.617948	-1.061074	0.967253
23	14	0	2.196749	-1.592343	-0.006526
24	6	0	3.565194	-0.311829	0.291131
25	6	0	5.629345	1.563268	0.780617
26	6	0	4.186539	0.383021	-0.761166
27	6	0	4.013866	-0.037734	1.597655
28	6	0	5.029699	0.886169	1.844761
29	6	0	5.205170	1.308885	-0.523886
30	6	0	2.754519	-3.222836	0.794781
31	6	0	2.001861	-1.881286	-1.865095
32	1	0	-2.022595	-1.907828	1.981480
33	1	0	-1.192449	-1.097526	3.313634
34	1	0	-0.870159	0.888988	2.063915
35	1	0	-4.445840	-0.519743	1.178913
36	1	0	-4.649460	0.956894	2.142138
37	1	0	-3.903035	-1.379119	3.402724

38	1	0	-3.194772	0.172979	3.882465
39	1	0	-1.913274	2.553406	0.479669
40	1	0	-4.957424	2.176725	0.271992
41	1	0	-2.978389	4.461852	-0.473044
42	1	0	-4.445868	4.088924	-1.390542
43	1	0	-4.573651	4.771980	0.227963
44	1	0	-3.812791	-3.849017	-2.459550
45	1	0	-0.366203	-3.146300	-0.018587
46	1	0	-2.865623	0.146427	-1.216184
47	1	0	-4.317776	-1.406891	-2.424815
48	1	0	-1.806893	-4.687937	-1.239065
49	1	0	1.367778	3.844489	-2.562154
50	1	0	0.790854	1.821325	1.168969
51	1	0	-0.764221	0.123689	-2.464917
52	1	0	0.141608	2.013220	-3.727870
53	1	0	1.687678	3.705626	-0.091733
54	1	0	1.001725	-0.370889	1.731284
55	1	0	0.287523	-1.938447	1.534869
56	1	0	6.422406	2.282886	0.968044
57	1	0	3.869329	0.207010	-1.785842
58	1	0	3.566343	-0.556065	2.444758
59	1	0	5.355723	1.075919	2.864658
60	1	0	5.666073	1.832131	-1.358211
61	1	0	3.698749	-3.569316	0.358623
62	1	0	2.912277	-3.113647	1.874667
63	1	0	2.011387	-4.017460	0.650185
64	1	0	2.940375	-2.273317	-2.276637
65	1	0	1.215036	-2.613386	-2.073681
66	1	0	1.750848	-0.964722	-2.406771

Alpha occ. eigenvalues -- -0.24286 -0.24126 -0.23806 -0.23254 -0.16966

Alpha virt. eigenvalues -- -0.00880 -0.00257 -0.00023 0.00262 0.00548

LUMO E: -0.00880 au = -0.239 eV

HOMO E: -0.16966 au = -4.617 eV

DELTA E: +0.16086 au = +4.377 eV

```
Zero-point correction=                           0.566900 (Hartree/Particle)
Thermal correction to Energy=                    0.597738
Thermal correction to Enthalpy=                  0.598682
Thermal correction to Gibbs Free Energy=         0.503674
Sum of electronic and zero-point Energies=          -1469.842409
Sum of electronic and thermal Energies=             -1469.811571
Sum of electronic and thermal Enthalpies=           -1469.810627
Sum of electronic and thermal Free Energies=        -1469.905635
```

en_Conf2

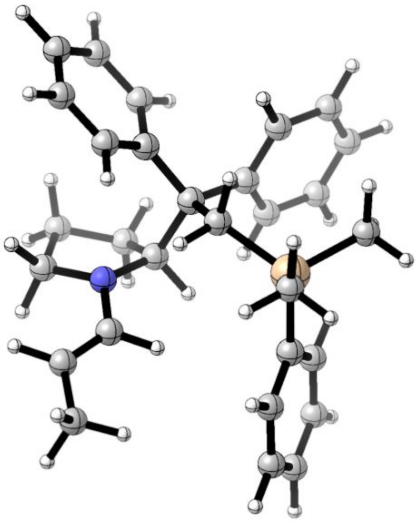

```
--------------------------------------------------------------------------------
   -- Stationary point found.
        Item               Value     Threshold  Converged?
Maximum Force            0.000006     0.000450     YES
RMS     Force            0.000001     0.000300     YES
Maximum Displacement     0.000250     0.001800     YES
RMS     Displacement     0.000054     0.001200     YES
Predicted change in Energy=-1.844946D-09
Optimization completed.

SCF Done:  E(RB3LYP) =  -1470.41103831     A.U. after    6 cycles

    Eigenvalues ---    0.00140   0.00156   0.00409   0.00543   0.00601

                        Standard orientation:
---------------------------------------------------------------------
Center     Atomic      Atomic             Coordinates (Angstroms)
Number     Number       Type             X           Y           Z
```

1	6	0	1.305281	0.729292	-2.362097
2	6	0	0.476595	0.703435	-1.052248
3	7	0	0.420147	2.121726	-0.671258
4	6	0	1.357783	2.961593	-1.419214
5	6	0	2.186755	1.988011	-2.267883
6	6	0	-0.749599	2.698362	-0.196398
7	6	0	-0.972326	4.009865	0.016274
8	6	0	-2.286597	4.551998	0.505658
9	6	0	1.046098	-0.244768	0.091864
10	6	0	1.030118	-1.730093	-0.333783
11	6	0	0.983164	-4.499311	-0.953771
12	6	0	0.386348	-2.209751	-1.483955
13	6	0	1.647988	-2.682517	0.498436
14	6	0	1.629826	-4.042056	0.196970
15	6	0	0.361529	-3.575305	-1.790221
16	6	0	2.491353	0.201302	0.427909
17	6	0	5.125470	1.117705	0.947277
18	6	0	2.734473	1.268891	1.308515
19	6	0	3.608964	-0.392412	-0.184262
20	6	0	4.906290	0.055417	0.070282
21	6	0	4.030196	1.721675	1.564309
22	6	0	0.142857	-0.112327	1.366907
23	14	0	-1.587986	-0.906871	1.642056
24	6	0	-2.919548	-0.517622	0.341562
25	6	0	-4.964808	0.017592	-1.546480
26	6	0	-3.830297	0.540978	0.523838
27	6	0	-3.079987	-1.309694	-0.812244
28	6	0	-4.083909	-1.046764	-1.746182
29	6	0	-4.837829	0.810234	-0.405233
30	6	0	-1.517347	-2.784427	1.867922
31	6	0	-2.140397	-0.150468	3.293914
32	1	0	1.894886	-0.176961	-2.516504
33	1	0	0.614446	0.818927	-3.209397
34	1	0	-0.545106	0.361901	-1.251977
35	1	0	1.985725	3.559730	-0.746236
36	1	0	0.785878	3.666431	-2.043707
37	1	0	3.135488	1.759157	-1.777944

38	1	0	2.414923	2.405697	-3.253703
39	1	0	-1.547063	1.985251	-0.003316
40	1	0	-0.177915	4.733310	-0.155663
41	1	0	-2.185157	5.078843	1.466076
42	1	0	-2.721757	5.274723	-0.199922
43	1	0	-3.021286	3.750117	0.644058
44	1	0	0.968143	-5.558928	-1.194109
45	1	0	-0.108600	-1.526609	-2.163906
46	1	0	2.164630	-2.348517	1.393548
47	1	0	2.121545	-4.746009	0.863561
48	1	0	-0.143078	-3.909022	-2.693529
49	1	0	6.134601	1.466775	1.149760
50	1	0	1.906179	1.764726	1.799842
51	1	0	3.469776	-1.223302	-0.867562
52	1	0	5.745771	-0.431958	-0.419373
53	1	0	4.179593	2.550186	2.252237
54	1	0	0.714911	-0.507218	2.219959
55	1	0	0.007169	0.951417	1.591167
56	1	0	-5.748283	0.223331	-2.271369
57	1	0	-3.760891	1.166352	1.410796
58	1	0	-2.413855	-2.151043	-0.984729
59	1	0	-4.182573	-1.676807	-2.626983
60	1	0	-5.525045	1.635337	-0.235111
61	1	0	-2.491622	-3.146791	2.218781
62	1	0	-1.269131	-3.321826	0.948138
63	1	0	-0.768508	-3.060678	2.619412
64	1	0	-2.188855	0.944400	3.261256
65	1	0	-3.129014	-0.520564	3.589801
66	1	0	-1.435805	-0.419276	4.090678

Alpha occ. eigenvalues -- -0.24515 -0.24338 -0.24021 -0.23121 -0.17137

Alpha virt. eigenvalues -- -0.01080 -0.00811 -0.00360 0.00065 0.00420

LUMO E: -0.01080 au = -0.294 eV

HOMO E: -0.17137 au = -4.663 eV

DELTA E: +0.16057 au = +4.369 eV

```
Zero-point correction=                           0.567243 (Hartree/Particle)
Thermal correction to Energy=                    0.597908
Thermal correction to Enthalpy=                  0.598852
Thermal correction to Gibbs Free Energy=         0.505622
Sum of electronic and zero-point Energies=          -1469.843795
Sum of electronic and thermal Energies=             -1469.813131
Sum of electronic and thermal Enthalpies=           -1469.812187
Sum of electronic and thermal Free Energies=        -1469.905416
```

en_Conf8b

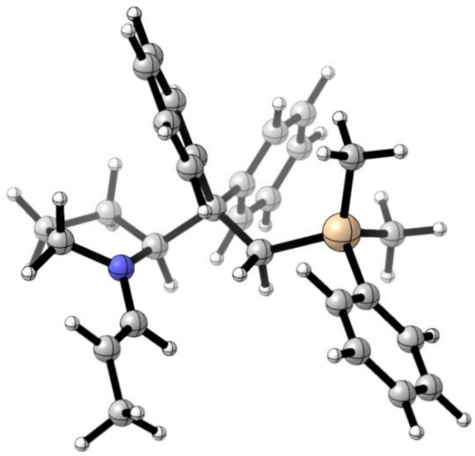

```
--------------------------------------------------------------------------------
    -- Stationary point found.
         Item               Value     Threshold  Converged?
 Maximum Force            0.000000     0.000450     YES
 RMS     Force            0.000000     0.000300     YES
 Maximum Displacement     0.000104     0.001800     YES
 RMS     Displacement     0.000014     0.001200     YES
 Predicted change in Energy=-2.462396D-12
 Optimization completed.

 SCF Done:  E(RB3LYP) =  -1470.40784238     A.U. after    6 cycles

    Eigenvalues ---    0.00072   0.00087   0.00125   0.00146   0.00216

                        Standard orientation:
 ---------------------------------------------------------------------
 Center     Atomic      Atomic             Coordinates (Angstroms)
 Number     Number       Type             X           Y           Z
 ---------------------------------------------------------------------
```

1	6	0	3.186275	0.999551	-1.392335
2	6	0	1.638008	0.914024	-1.286804
3	7	0	1.219758	2.275956	-0.946856
4	6	0	2.363082	3.122672	-0.607112
5	6	0	3.512176	2.502820	-1.413757
6	6	0	0.036474	2.815042	-1.431889
7	6	0	-0.483882	4.027495	-1.163742
8	6	0	-1.743893	4.541254	-1.802699
9	6	0	1.099043	-0.201925	-0.265647
10	6	0	1.673565	-1.587980	-0.646069
11	6	0	2.554610	-4.188449	-1.384361
12	6	0	1.875141	-2.597840	0.310751
13	6	0	1.906468	-1.934706	-1.988599
14	6	0	2.342702	-3.209335	-2.353780
15	6	0	2.312668	-3.874208	-0.047495
16	6	0	1.485945	0.260232	1.154990
17	6	0	2.264607	1.306067	3.682066
18	6	0	0.662080	1.145156	1.872054
19	6	0	2.721441	-0.068559	1.742625
20	6	0	3.104841	0.439348	2.985493
21	6	0	1.040612	1.658058	3.113848
22	6	0	-0.451717	-0.287025	-0.464308
23	14	0	-1.620181	-1.536859	0.412155
24	6	0	-3.350911	-0.774138	0.214780
25	6	0	-5.917217	0.393785	-0.056878
26	6	0	-3.690106	0.419105	0.881875
27	6	0	-4.341936	-1.361996	-0.591515
28	6	0	-5.608833	-0.789108	-0.728815
29	6	0	-4.952360	0.998607	0.751630
30	6	0	-1.606889	-3.215479	-0.463168
31	6	0	-1.305935	-1.787322	2.263810
32	1	0	3.657074	0.530261	-0.525969
33	1	0	3.555869	0.476921	-2.278439
34	1	0	1.205628	0.668666	-2.265705
35	1	0	2.571414	3.105330	0.471660
36	1	0	2.150046	4.156410	-0.895247
37	1	0	4.500234	2.727146	-0.997881
38	1	0	3.483311	2.888352	-2.439925

39	1	0	-0.501243	2.147155	-2.105096
40	1	0	0.012811	4.686426	-0.453602
41	1	0	-2.196871	3.785551	-2.455406
42	1	0	-2.497062	4.823842	-1.053196
43	1	0	-1.563719	5.436916	-2.415452
44	1	0	2.897742	-5.180182	-1.666179
45	1	0	1.690699	-2.387718	1.357924
46	1	0	1.743042	-1.209155	-2.777871
47	1	0	2.515187	-3.433273	-3.403459
48	1	0	2.461531	-4.623240	0.726255
49	1	0	2.558649	1.701991	4.650469
50	1	0	-0.286477	1.462382	1.456891
51	1	0	3.398029	-0.745846	1.233408
52	1	0	4.065874	0.152579	3.405016
53	1	0	0.372534	2.339438	3.634641
54	1	0	-0.892188	0.701242	-0.298739
55	1	0	-0.625630	-0.486512	-1.532873
56	1	0	-6.902111	0.842049	-0.161288
57	1	0	-2.957000	0.910053	1.520221
58	1	0	-4.127287	-2.285510	-1.123916
59	1	0	-6.354223	-1.267393	-1.359789
60	1	0	-5.184019	1.920228	1.280051
61	1	0	-2.381115	-3.875243	-0.052634
62	1	0	-1.791870	-3.114225	-1.539249
63	1	0	-0.641837	-3.717228	-0.344008
64	1	0	-2.077049	-2.450008	2.675822
65	1	0	-0.331894	-2.247486	2.459329
66	1	0	-1.340208	-0.846462	2.822361

Alpha occ. eigenvalues -- -0.24299 -0.24134 -0.23925 -0.23274 -0.17259

Alpha virt. eigenvalues -- -0.00736 -0.00445 -0.00229 0.00189 0.00660

LUMO E: -0.00736 au = -0.200 eV

HOMO E: -0.17259 au = -4.696 eV

DELTA E: +0.16523 au = +4.496 eV

Zero-point correction= 0.566780 (Hartree/Particle)

Thermal correction to Energy= 0.597736

Thermal correction to Enthalpy= 0.598680

Thermal correction to Gibbs Free Energy= 0.503108

Sum of electronic and zero-point Energies= -1469.841062

Sum of electronic and thermal Energies= -1469.810107

Sum of electronic and thermal Enthalpies= -1469.809162

Sum of electronic and thermal Free Energies= -1469.904735

en_Conf4b

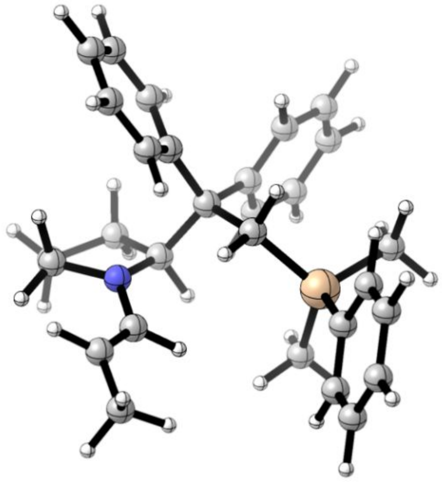

-- Stationary point found.

Item		Value	Threshold	Converged?
Maximum	Force	0.000000	0.000450	YES
RMS	Force	0.000000	0.000300	YES
Maximum	Displacement	0.000057	0.001800	YES
RMS	Displacement	0.000007	0.001200	YES

Predicted change in Energy=-8.226349D-13

Optimization completed.

SCF Done: E(RB3LYP) = -1470.40812971 A.U. after 6 cycles

Eigenvalues --- 0.00065 0.00105 0.00128 0.00180 0.00210

Standard orientation:

Center Number	Atomic Number	Atomic Type	Coordinates (Angstroms) X	Y	Z
1	6	0	-2.625906	-0.911049	-1.903944

2	6	0	-1.203485	-0.729200	-1.294360
3	7	0	-0.739600	-2.103554	-1.066822
4	6	0	-1.810418	-3.082426	-1.239492
5	6	0	-2.745703	-2.405930	-2.248308
6	6	0	0.594968	-2.461313	-1.174884
7	6	0	1.146936	-3.666171	-0.934602
8	6	0	2.606865	-3.957514	-1.144317
9	6	0	-1.153779	0.175898	0.017203
10	6	0	-1.685448	1.603910	-0.236277
11	6	0	-2.509397	4.297447	-0.585126
12	6	0	-1.864069	2.154115	-1.513879
13	6	0	-1.922098	2.450469	0.863330
14	6	0	-2.330186	3.771676	0.696614
15	6	0	-2.270957	3.481197	-1.688092
16	6	0	-1.980910	-0.535917	1.117967
17	6	0	-3.520270	-1.944377	3.042344
18	6	0	-3.343075	-0.252225	1.321308
19	6	0	-1.414444	-1.549482	1.909560
20	6	0	-2.170591	-2.243399	2.855956
21	6	0	-4.102737	-0.942263	2.267868
22	6	0	0.335605	0.328528	0.486208
23	14	0	1.653154	1.432157	-0.379677
24	6	0	3.305264	0.809591	0.323005
25	6	0	5.747362	-0.123035	1.413525
26	6	0	4.282550	0.198970	-0.483697
27	6	0	3.594198	0.939327	1.695350
28	6	0	4.795454	0.481224	2.237368
29	6	0	5.488335	-0.262795	0.049917
30	6	0	1.726463	1.323009	-2.272821
31	6	0	1.469177	3.256311	0.092686
32	1	0	-3.392672	-0.631169	-1.178528
33	1	0	-2.772974	-0.280570	-2.784434
34	1	0	-0.534847	-0.260115	-2.026855
35	1	0	-2.322989	-3.295866	-0.291221
36	1	0	-1.389413	-4.019892	-1.615803
37	1	0	-3.776995	-2.769583	-2.189443
38	1	0	-2.380264	-2.595055	-3.264643
39	1	0	1.239456	-1.645174	-1.498083

40	1	0	0.529083	-4.483652	-0.567304
41	1	0	3.158428	-3.051570	-1.422526
42	1	0	3.075886	-4.359313	-0.235021
43	1	0	2.774655	-4.702190	-1.936729
44	1	0	-2.829402	5.327169	-0.719381
45	1	0	-1.686107	1.555951	-2.399234
46	1	0	-1.793520	2.061844	1.869135
47	1	0	-2.506934	4.392500	1.571278
48	1	0	-2.402618	3.870062	-2.694662
49	1	0	-4.107524	-2.481809	3.782299
50	1	0	-3.820090	0.534074	0.745621
51	1	0	-0.371572	-1.815528	1.787997
52	1	0	-1.696549	-3.020627	3.450214
53	1	0	-5.151947	-0.689113	2.398276
54	1	0	0.318621	0.713399	1.516409
55	1	0	0.792478	-0.662061	0.567216
56	1	0	6.684604	-0.480957	1.832123
57	1	0	4.107298	0.081920	-1.550715
58	1	0	2.870656	1.409541	2.359354
59	1	0	4.989901	0.596154	3.301002
60	1	0	6.224772	-0.729793	-0.599609
61	1	0	2.575515	1.913793	-2.638983
62	1	0	1.847293	0.303329	-2.654696
63	1	0	0.821755	1.744525	-2.723669
64	1	0	2.327286	3.826423	-0.283960
65	1	0	0.558209	3.696880	-0.324468
66	1	0	1.435352	3.393684	1.179806

Alpha occ. eigenvalues -- -0.24278 -0.24146 -0.23954 -0.23067 -0.17165

Alpha virt. eigenvalues -- -0.00687 -0.00516 -0.00190 0.00446 0.00591

LUMO E: -0.00687 au = -0.187 eV

HOMO E: -0.17165 au = -4.671 eV

DELTA E: +0.16478 au = +4.484 eV

Zero-point correction= 0.566941 (Hartree/Particle)

Thermal correction to Energy= 0.597783

Thermal correction to Enthalpy= 0.598728

Thermal correction to Gibbs Free Energy= 0.503885

Sum of electronic and zero-point Energies= -1469.841189

Sum of electronic and thermal Energies= -1469.810346

Sum of electronic and thermal Enthalpies= -1469.809402

Sum of electronic and thermal Free Energies= -1469.904245

en_Conf3b

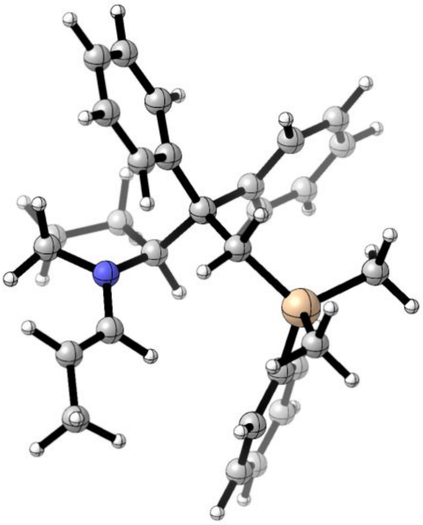

-- Stationary point found.

Item	Value	Threshold	Converged?
Maximum Force	0.000000	0.000450	YES
RMS Force	0.000000	0.000300	YES
Maximum Displacement	0.000164	0.001800	YES
RMS Displacement	0.000037	0.001200	YES

Predicted change in Energy=-4.142059D-11

Optimization completed.

SCF Done: E(RB3LYP) = -1470.40877954 A.U. after 6 cycles

Eigenvalues --- 0.00095 0.00119 0.00162 0.00167 0.00223

Standard orientation:

Center Number	Atomic Number	Atomic Type	Coordinates (Angstroms) X	Y	Z
1	6	0	-1.224596	-0.481733	-2.425810

2	6	0	-0.483073	-0.639201	-1.062920
3	7	0	-0.640660	-2.061792	-0.731723
4	6	0	-1.640625	-2.707300	-1.581955
5	6	0	-1.559012	-1.911109	-2.890264
6	6	0	0.386185	-2.788719	-0.146693
7	6	0	0.352906	-4.073803	0.255673
8	6	0	1.550516	-4.793774	0.810427
9	6	0	-1.006265	0.322238	0.096403
10	6	0	-0.898407	1.814822	-0.283440
11	6	0	-0.659554	4.593011	-0.810261
12	6	0	-0.138862	2.290444	-1.361787
13	6	0	-1.525424	2.775855	0.531935
14	6	0	-1.412988	4.140320	0.275368
15	6	0	-0.021885	3.659855	-1.624473
16	6	0	-2.478083	-0.056761	0.404262
17	6	0	-5.163443	-0.850644	0.870493
18	6	0	-3.555313	0.576029	-0.241145
19	6	0	-2.790229	-1.099837	1.292606
20	6	0	-4.110178	-1.491952	1.522472
21	6	0	-4.877084	0.188357	-0.013794
22	6	0	-0.126066	0.107793	1.376504
23	14	0	1.653002	0.778200	1.665103
24	6	0	2.943529	0.331811	0.341951
25	6	0	4.921282	-0.313124	-1.583764
26	6	0	3.247398	1.205517	-0.720137
27	6	0	3.677922	-0.867912	0.414390
28	6	0	4.650477	-1.191749	-0.533918
29	6	0	4.218778	0.889759	-1.672341
30	6	0	1.698384	2.648580	1.947469
31	6	0	2.167382	-0.059977	3.289535
32	1	0	-2.140841	0.099446	-2.303662
33	1	0	-0.608517	0.043835	-3.159966
34	1	0	0.587627	-0.433463	-1.183385
35	1	0	-2.643818	-2.654116	-1.138573
36	1	0	-1.381494	-3.762153	-1.711316
37	1	0	-2.481133	-1.957052	-3.479432
38	1	0	-0.745965	-2.311717	-3.507092
39	1	0	1.307414	-2.219946	-0.027258

40	1	0	-0.571878	-4.643506	0.181215
41	1	0	2.419345	-4.128014	0.878566
42	1	0	1.360057	-5.195028	1.816542
43	1	0	1.846548	-5.649821	0.186151
44	1	0	-0.572702	5.656383	-1.016498
45	1	0	0.382635	1.599511	-2.012250
46	1	0	-2.122954	2.446227	1.376755
47	1	0	-1.915586	4.851512	0.926004
48	1	0	0.569388	3.990369	-2.474905
49	1	0	-6.191308	-1.153295	1.052505
50	1	0	-3.365854	1.401217	-0.919583
51	1	0	-1.998395	-1.626956	1.810420
52	1	0	-4.311612	-2.303351	2.217509
53	1	0	-5.681944	0.706495	-0.529220
54	1	0	-0.676380	0.530667	2.230525
55	1	0	-0.062511	-0.965298	1.588009
56	1	0	5.678883	-0.560611	-2.322983
57	1	0	2.720936	2.152432	-0.805792
58	1	0	3.497706	-1.563272	1.231228
59	1	0	5.199306	-2.126466	-0.449691
60	1	0	4.431547	1.586008	-2.480107
61	1	0	2.700393	2.949158	2.277765
62	1	0	1.447497	3.228922	1.054968
63	1	0	0.989494	2.936682	2.732779
64	1	0	3.177976	0.242428	3.588078
65	1	0	1.484442	0.223930	4.099838
66	1	0	2.152246	-1.154191	3.222756

Alpha occ. eigenvalues -- -0.24486 -0.24303 -0.23925 -0.23071 -0.17083

Alpha virt. eigenvalues -- -0.01046 -0.00738 -0.00343 0.00210 0.00475

LUMO E: -0.01046 au = -0.285 eV

HOMO E: -0.17083 au = -4.649 eV

DELTA E: +0.16037 au = +4.364 eV

Zero-point correction= 0.567039 (Hartree/Particle)

Thermal correction to Energy= 0.597819

```
Thermal correction to Enthalpy=                  0.598763
Thermal correction to Gibbs Free Energy=         0.504818
Sum of electronic and zero-point Energies=          -1469.841740
Sum of electronic and thermal Energies=             -1469.810961
Sum of electronic and thermal Enthalpies=           -1469.810016
Sum of electronic and thermal Free Energies=        -1469.903962
```

en_Conf13b

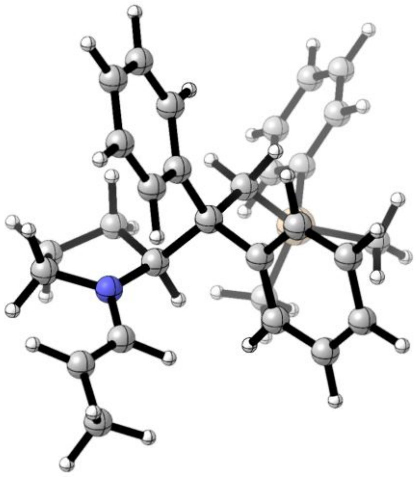

```
--------------------------------------------------------------------------------
    -- Stationary point found.
         Item               Value     Threshold  Converged?
 Maximum Force            0.000001     0.000450     YES
 RMS     Force            0.000000     0.000300     YES
 Maximum Displacement     0.000128     0.001800     YES
 RMS     Displacement     0.000020     0.001200     YES
 Predicted change in Energy=-1.385661D-11
 Optimization completed.

 SCF Done:  E(RB3LYP) =  -1470.40711510     A.U. after    6 cycles

    Eigenvalues ---    0.00038   0.00094   0.00170   0.00212   0.00231

                        Standard orientation:
 ---------------------------------------------------------------------
 Center     Atomic      Atomic             Coordinates (Angstroms)
 Number     Number       Type             X           Y           Z
 ---------------------------------------------------------------------
      1          6           0        0.273298   -1.859014   -1.665954
      2          6           0        0.936155   -0.526990   -1.220429
```

3	7	0	2.364040	-0.771093	-1.477073
4	6	0	2.627680	-2.113022	-1.985517
5	6	0	1.288462	-2.522532	-2.603234
6	6	0	3.332399	0.214056	-1.533801
7	6	0	4.660743	0.045718	-1.694437
8	6	0	5.623414	1.195780	-1.803303
9	6	0	0.535542	-0.124480	0.269995
10	6	0	1.179740	-1.152375	1.234355
11	6	0	2.441933	-3.059333	2.913143
12	6	0	2.567506	-1.117140	1.470437
13	6	0	0.447012	-2.164018	1.872960
14	6	0	1.066912	-3.105434	2.701313
15	6	0	3.188906	-2.055432	2.292464
16	6	0	0.964510	1.312689	0.664295
17	6	0	1.553089	3.964531	1.494467
18	6	0	1.094067	2.360354	-0.261172
19	6	0	1.115480	1.642452	2.022439
20	6	0	1.404149	2.943420	2.433535
21	6	0	1.393637	3.664253	0.142845
22	6	0	-1.027609	-0.158115	0.388032
23	14	0	-2.269023	1.045908	-0.450544
24	6	0	-3.932458	0.140810	-0.282522
25	6	0	-6.387199	-1.241896	0.010645
26	6	0	-4.563191	-0.474299	-1.379308
27	6	0	-4.574883	0.041102	0.966773
28	6	0	-5.783797	-0.639663	1.116453
29	6	0	-5.773625	-1.157147	-1.239541
30	6	0	-2.407785	2.686631	0.481967
31	6	0	-1.971510	1.390553	-2.292204
32	1	0	0.111803	-2.505352	-0.797391
33	1	0	-0.697781	-1.691951	-2.140139
34	1	0	0.582626	0.277201	-1.877976
35	1	0	2.934209	-2.803350	-1.184202
36	1	0	3.438712	-2.073131	-2.721222
37	1	0	1.157570	-3.607688	-2.667949
38	1	0	1.209111	-2.110518	-3.616490
39	1	0	2.945897	1.222221	-1.426527
40	1	0	5.084495	-0.954920	-1.751574

41	1	0	5.111107	2.157577	-1.680849
42	1	0	6.413053	1.143821	-1.039811
43	1	0	6.134782	1.219481	-2.777230
44	1	0	2.926189	-3.789326	3.556556
45	1	0	3.172907	-0.342812	1.013577
46	1	0	-0.626522	-2.230835	1.739239
47	1	0	0.463814	-3.872805	3.180213
48	1	0	4.262856	-1.996681	2.450880
49	1	0	1.787014	4.977261	1.811194
50	1	0	0.961461	2.176322	-1.321411
51	1	0	1.012204	0.865222	2.772568
52	1	0	1.516301	3.155856	3.493715
53	1	0	1.499194	4.443929	-0.607341
54	1	0	-1.391837	-1.154934	0.116627
55	1	0	-1.273353	-0.050954	1.454821
56	1	0	-7.329934	-1.771424	0.122861
57	1	0	-4.107852	-0.419823	-2.365669
58	1	0	-4.128943	0.506872	1.843795
59	1	0	-6.256514	-0.697904	2.093926
60	1	0	-6.237935	-1.620364	-2.106845
61	1	0	-3.223788	3.289591	0.065331
62	1	0	-2.626590	2.527260	1.544428
63	1	0	-1.485307	3.271748	0.421779
64	1	0	-2.837221	1.916287	-2.713590
65	1	0	-1.097374	2.032004	-2.445645
66	1	0	-1.821788	0.478302	-2.880697

Alpha occ. eigenvalues --   -0.24487 -0.24286 -0.24076 -0.22990 -0.16970

Alpha virt. eigenvalues --   -0.00813 -0.00475 -0.00239   0.00260   0.00541

LUMO E: -0.00813 au = -0.221 eV

HOMO E: -0.16970 au = -4.618 eV

DELTA E: +0.16157 au = +4.397 eV

Zero-point correction=   0.566897 (Hartree/Particle)

Thermal correction to Energy=   0.597799

Thermal correction to Enthalpy=   0.598743

```
Thermal correction to Gibbs Free Energy=         0.503410
Sum of electronic and zero-point Energies=          -1469.840218
Sum of electronic and thermal Energies=             -1469.809316
Sum of electronic and thermal Enthalpies=           -1469.808372
Sum of electronic and thermal Free Energies=        -1469.903705
```

en_Conf6b

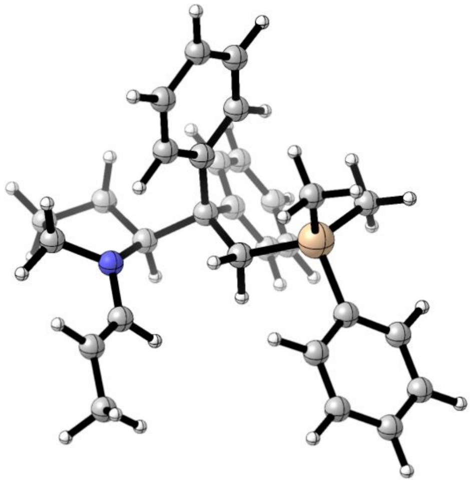

```
--------------------------------------------------------------------------------
   -- Stationary point found.
        Item               Value     Threshold  Converged?
Maximum Force            0.000000     0.000450     YES
RMS     Force            0.000000     0.000300     YES
Maximum Displacement     0.000058     0.001800     YES
RMS     Displacement     0.000007     0.001200     YES
Predicted change in Energy=-8.016267D-13
Optimization completed.

SCF Done:  E(RB3LYP) =  -1470.40707974     A.U. after    6 cycles

    Eigenvalues ---    0.00021   0.00115   0.00150   0.00207   0.00235

                        Standard orientation:
---------------------------------------------------------------------
Center     Atomic      Atomic             Coordinates (Angstroms)
Number     Number       Type             X           Y           Z
---------------------------------------------------------------------
     1          6           0        3.269698    0.936571   -1.174293
     2          6           0        1.726220    1.012799   -1.058112
     3          7           0        1.479305    2.380386   -0.558661
```

4	6	0	2.731886	3.131650	-0.390802
5	6	0	3.726216	2.395013	-1.293699
6	6	0	0.318597	3.059817	-0.926558
7	6	0	-0.039104	4.309576	-0.580700
8	6	0	-1.290485	4.976201	-1.080470
9	6	0	1.062122	-0.203495	-0.239495
10	6	0	1.264285	-1.417248	-1.182290
11	6	0	1.709819	-3.501252	-3.065270
12	6	0	0.271786	-1.811779	-2.093793
13	6	0	2.496964	-2.092741	-1.260448
14	6	0	2.717005	-3.119095	-2.178685
15	6	0	0.486402	-2.837177	-3.018556
16	6	0	1.680110	-0.405724	1.164528
17	6	0	2.642422	-0.719410	3.818918
18	6	0	1.961198	-1.674214	1.698668
19	6	0	1.867832	0.699716	2.014939
20	6	0	2.349900	0.548494	3.315046
21	6	0	2.435962	-1.830649	3.003631
22	6	0	-0.447568	0.121308	-0.020055
23	14	0	-1.660874	-1.002357	0.965461
24	6	0	-3.381851	-0.466001	0.361435
25	6	0	-5.935342	0.374793	-0.530895
26	6	0	-3.779330	0.883670	0.437775
27	6	0	-4.306749	-1.378135	-0.177598
28	6	0	-5.567493	-0.967925	-0.618622
29	6	0	-5.036032	1.302396	-0.000357
30	6	0	-1.481206	-2.870398	0.713128
31	6	0	-1.539805	-0.628681	2.818238
32	1	0	3.692474	0.494265	-0.265801
33	1	0	3.581006	0.321137	-2.020516
34	1	0	1.280561	0.930466	-2.061151
35	1	0	3.069106	3.126694	0.656172
36	1	0	2.574426	4.173137	-0.686493
37	1	0	4.769677	2.541602	-0.995150
38	1	0	3.616952	2.748940	-2.326248
39	1	0	-0.345517	2.479109	-1.565126
40	1	0	0.588443	4.887418	0.096022
41	1	0	-1.880599	4.297854	-1.707952

42	1	0	-1.934441	5.307799	-0.253333
43	1	0	-1.071382	5.870856	-1.681387
44	1	0	1.878092	-4.301232	-3.781188
45	1	0	-0.693632	-1.319849	-2.097586
46	1	0	3.302258	-1.820106	-0.587795
47	1	0	3.682333	-3.618640	-2.199252
48	1	0	-0.312017	-3.112138	-3.703123
49	1	0	3.015134	-0.839216	4.832708
50	1	0	1.807007	-2.561238	1.095726
51	1	0	1.615296	1.689658	1.654765
52	1	0	2.486373	1.428334	3.939041
53	1	0	2.641795	-2.830129	3.378668
54	1	0	-0.491322	1.074822	0.520248
55	1	0	-0.924349	0.332692	-0.985005
56	1	0	-6.915588	0.697377	-0.872907
57	1	0	-3.097014	1.627537	0.845928
58	1	0	-4.043838	-2.430439	-0.256797
59	1	0	-6.261351	-1.697132	-1.030176
60	1	0	-5.314376	2.351050	0.072054
61	1	0	-2.255932	-3.396924	1.284710
62	1	0	-1.566456	-3.174595	-0.334523
63	1	0	-0.511308	-3.222074	1.079008
64	1	0	-2.311808	-1.181404	3.367396
65	1	0	-0.563765	-0.911658	3.226108
66	1	0	-1.689877	0.437845	3.023479

```
 Alpha  occ. eigenvalues --   -0.24348  -0.24137  -0.23933  -0.23324  -0.17899
 Alpha virt. eigenvalues --   -0.00700  -0.00318  -0.00196   0.00050   0.00701

LUMO  E: -0.00700 au = -0.190 eV
HOMO  E: -0.17899 au = -4.871 eV
DELTA E: +0.17199 au = +4.680 eV

 Zero-point correction=                           0.567134 (Hartree/Particle)
 Thermal correction to Energy=                    0.597905
 Thermal correction to Enthalpy=                  0.598849
 Thermal correction to Gibbs Free Energy=         0.503401
```

Sum of electronic and zero-point Energies=   -1469.839946
Sum of electronic and thermal Energies=   -1469.809175
Sum of electronic and thermal Enthalpies=   -1469.808230
Sum of electronic and thermal Free Energies=   -1469.903678

en_Conf5b

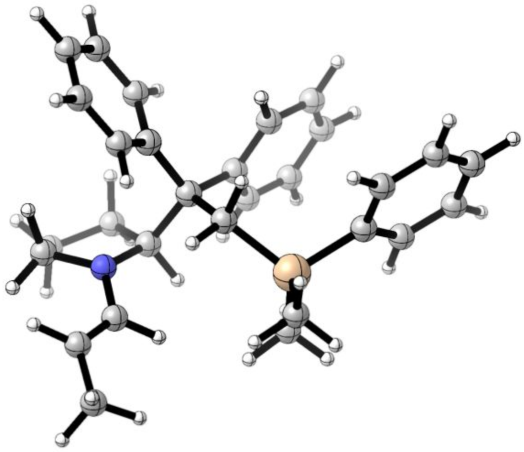

-- Stationary point found.

Item		Value	Threshold	Converged?
Maximum	Force	0.000000	0.000450	YES
RMS	Force	0.000000	0.000300	YES
Maximum	Displacement	0.000021	0.001800	YES
RMS	Displacement	0.000004	0.001200	YES

Predicted change in Energy=-6.084110D-13

Optimization completed.

SCF Done: E(RB3LYP) = -1470.40706799   A.U. after   6 cycles

Eigenvalues ---   0.00107   0.00123   0.00139   0.00197   0.00218

Standard orientation:

Center Number	Atomic Number	Atomic Type	Coordinates (Angstroms) X	Y	Z
1	6	0	-2.361916	-0.938738	2.096741
2	6	0	-1.665052	0.103744	1.170582
3	7	0	-2.780129	0.847572	0.565975
4	6	0	-4.066354	0.196912	0.819739

5	6	0	-3.842490	-0.522832	2.154866
6	6	0	-2.699518	2.214644	0.342814
7	6	0	-3.623604	3.014438	-0.222802
8	6	0	-3.454839	4.502501	-0.354471
9	6	0	-0.672772	-0.518022	0.090924
10	6	0	0.499787	-1.280628	0.748026
11	6	0	2.753109	-2.631904	1.817943
12	6	0	1.363537	-2.034110	-0.068871
13	6	0	0.809300	-1.216501	2.114183
14	6	0	1.919097	-1.883594	2.643923
15	6	0	2.468857	-2.700825	0.452522
16	6	0	-1.490935	-1.459479	-0.830335
17	6	0	-3.092519	-3.152620	-2.450435
18	6	0	-2.218005	-0.955207	-1.922197
19	6	0	-1.592340	-2.838784	-0.577229
20	6	0	-2.378628	-3.674369	-1.372667
21	6	0	-3.006842	-1.786586	-2.719453
22	6	0	-0.050944	0.641049	-0.762622
23	14	0	1.333295	1.831843	-0.162966
24	6	0	3.049061	1.087357	-0.473118
25	6	0	5.625435	0.039872	-1.001034
26	6	0	3.440900	0.723672	-1.776007
27	6	0	3.991520	0.911877	0.554649
28	6	0	5.263628	0.394885	0.298461
29	6	0	4.708490	0.205744	-2.041462
30	6	0	1.159398	3.354480	-1.284342
31	6	0	1.230882	2.421752	1.636352
32	1	0	-2.269828	-1.944576	1.681863
33	1	0	-1.911131	-0.960491	3.092071
34	1	0	-1.074448	0.807232	1.770543
35	1	0	-4.324922	-0.508429	0.018907
36	1	0	-4.853840	0.954212	0.873890
37	1	0	-4.513773	-1.375644	2.301187
38	1	0	-4.009982	0.181593	2.978348
39	1	0	-1.762158	2.655456	0.681460
40	1	0	-4.547088	2.588192	-0.610688
41	1	0	-3.536103	4.833294	-1.399879
42	1	0	-4.219407	5.058770	0.207622

43	1	0	-2.476569	4.828505	0.019569
44	1	0	3.614304	-3.151995	2.228592
45	1	0	1.159516	-2.106257	-1.132924
46	1	0	0.188996	-0.643924	2.792763
47	1	0	2.122559	-1.814999	3.709719
48	1	0	3.113227	-3.270293	-0.211874
49	1	0	-3.702817	-3.800600	-3.074041
50	1	0	-2.180475	0.101420	-2.157348
51	1	0	-1.033858	-3.276143	0.243680
52	1	0	-2.426362	-4.736766	-1.146945
53	1	0	-3.553740	-1.359862	-3.556639
54	1	0	0.377124	0.190674	-1.669622
55	1	0	-0.856146	1.288723	-1.123955
56	1	0	6.615370	-0.361390	-1.203593
57	1	0	2.746370	0.847198	-2.605741
58	1	0	3.732360	1.175699	1.576815
59	1	0	5.971161	0.269072	1.114527
60	1	0	4.982697	-0.065342	-3.058215
61	1	0	1.940409	4.092457	-1.066707
62	1	0	1.245723	3.088789	-2.344841
63	1	0	0.186702	3.842929	-1.147334
64	1	0	2.033803	3.143633	1.831210
65	1	0	0.283231	2.932669	1.841976
66	1	0	1.336424	1.605019	2.357211

Alpha occ. eigenvalues -- -0.24099 -0.23953 -0.23883 -0.22741 -0.17474

Alpha virt. eigenvalues -- -0.00435 -0.00313 0.00152 0.00490 0.00766

LUMO E: -0.00435 au = -0.118 eV

HOMO E: -0.17474 au = -4.755 eV

DELTA E: +0.17039 au = +4.637 eV

Zero-point correction= 0.566677 (Hartree/Particle)

Thermal correction to Energy= 0.597582

Thermal correction to Enthalpy= 0.598527

Thermal correction to Gibbs Free Energy= 0.503793

Sum of electronic and zero-point Energies= -1469.840391

```
Sum of electronic and thermal Energies=          -1469.809486
Sum of electronic and thermal Enthalpies=        -1469.808541
Sum of electronic and thermal Free Energies=     -1469.903275
```

en_Conf9b

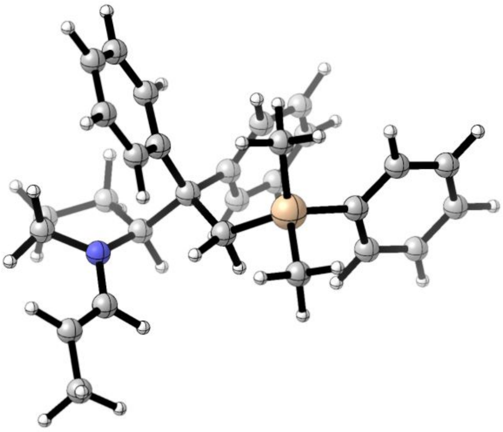

```
--------------------------------------------------------------------------------
    -- Stationary point found.
         Item               Value     Threshold  Converged?
Maximum Force            0.000004     0.000450     YES
RMS     Force            0.000000     0.000300     YES
Maximum Displacement     0.000324     0.001800     YES
RMS     Displacement     0.000067     0.001200     YES
Predicted change in Energy=-2.230116D-10
Optimization completed.

SCF Done:  E(RB3LYP) =  -1470.40664863     A.U. after    6 cycles

    Eigenvalues ---    0.00069   0.00109   0.00125   0.00133   0.00227

                        Standard orientation:
---------------------------------------------------------------------
Center     Atomic      Atomic             Coordinates (Angstroms)
Number     Number       Type             X           Y           Z
---------------------------------------------------------------------
     1          6           0        2.388502   -1.779127   -1.752617
     2          6           0        1.793579   -0.390875   -1.383959
     3          7           0        2.958443    0.414990   -1.004968
     4          6           0        4.166317   -0.402987   -0.887198
     5          6           0        3.906814   -1.558448   -1.862798
     6          6           0        3.029164    1.768375   -1.303575
```

7	6	0	4.010059	2.631347	-0.977608
8	6	0	4.020349	4.069309	-1.415781
9	6	0	0.645431	-0.436953	-0.266007
10	6	0	-0.517550	-1.345010	-0.732637
11	6	0	-2.772538	-2.857546	-1.567841
12	6	0	-0.807791	-1.542810	-2.092903
13	6	0	-1.409116	-1.912838	0.193574
14	6	0	-2.514665	-2.659557	-0.212136
15	6	0	-1.913222	-2.289457	-2.505841
16	6	0	1.305943	-0.912815	1.044943
17	6	0	2.713966	-1.780750	3.355493
18	6	0	1.998180	-0.007104	1.868004
19	6	0	1.356509	-2.269553	1.411597
20	6	0	2.043519	-2.698721	2.549105
21	6	0	2.688930	-0.430906	3.004599
22	6	0	0.064636	1.011518	-0.143461
23	14	0	-1.351234	1.561583	1.031620
24	6	0	-3.065118	1.153659	0.328103
25	6	0	-5.660760	0.680167	-0.700640
26	6	0	-3.363123	1.354588	-1.032574
27	6	0	-4.111709	0.708267	1.155712
28	6	0	-5.393242	0.473867	0.652968
29	6	0	-4.639939	1.121940	-1.544361
30	6	0	-1.229440	0.932295	2.813526
31	6	0	-1.189324	3.454478	1.075360
32	1	0	2.174252	-2.507367	-0.967064
33	1	0	1.955230	-2.169468	-2.676908
34	1	0	1.341616	0.076892	-2.268783
35	1	0	4.307930	-0.766001	0.140010
36	1	0	5.040409	0.196037	-1.159442
37	1	0	4.483053	-2.459131	-1.625816
38	1	0	4.176993	-1.243327	-2.877837
39	1	0	2.171621	2.133834	-1.869349
40	1	0	4.848206	2.295595	-0.369425
41	1	0	3.112286	4.322158	-1.976335
42	1	0	4.083766	4.757748	-0.560760
43	1	0	4.878300	4.300003	-2.064363
44	1	0	-3.633198	-3.438655	-1.887514

45	1	0	-0.175199	-1.110643	-2.859789
46	1	0	-1.235431	-1.774879	1.254998
47	1	0	-3.179164	-3.079754	0.538193
48	1	0	-2.098926	-2.422575	-3.568715
49	1	0	3.248332	-2.110980	4.242343
50	1	0	2.019759	1.046836	1.619156
51	1	0	0.840398	-3.009120	0.809301
52	1	0	2.051412	-3.756516	2.799811
53	1	0	3.210868	0.301784	3.615083
54	1	0	0.882565	1.709228	0.067592
55	1	0	-0.286065	1.298803	-1.146660
56	1	0	-6.657003	0.498190	-1.096052
57	1	0	-2.585922	1.695683	-1.713852
58	1	0	-3.928438	0.536512	2.213843
59	1	0	-6.181624	0.129363	1.318088
60	1	0	-4.838373	1.283667	-2.601155
61	1	0	-1.989609	1.420259	3.436285
62	1	0	-1.369647	-0.149713	2.901633
63	1	0	-0.250532	1.167711	3.243239
64	1	0	-1.990871	3.900843	1.675638
65	1	0	-0.231487	3.766068	1.510551
66	1	0	-1.253515	3.887703	0.069885

Alpha occ. eigenvalues -- -0.24243 -0.23836 -0.23661 -0.22849 -0.17417
Alpha virt. eigenvalues -- -0.00704 -0.00215 0.00166 0.00474 0.00810

LUMO E: -0.00704 au = -0.192 eV
HOMO E: -0.17417 au = -4.739 eV
DELTA E: +0.16713 au = +4.548 eV

Zero-point correction= 0.566580 (Hartree/Particle)
Thermal correction to Energy= 0.597529
Thermal correction to Enthalpy= 0.598473
Thermal correction to Gibbs Free Energy= 0.502999
Sum of electronic and zero-point Energies= -1469.840069
Sum of electronic and thermal Energies= -1469.809120
Sum of electronic and thermal Enthalpies= -1469.808176

Sum of electronic and thermal Free Energies=   -1469.903650

en_Conf17b

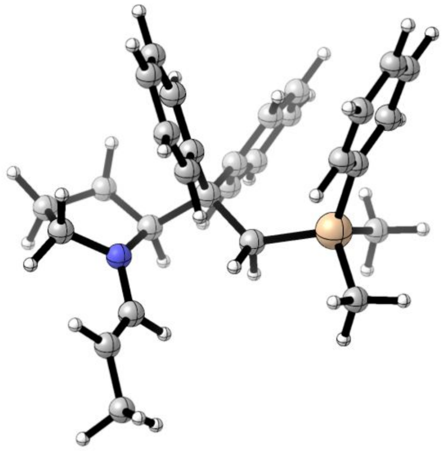

-- Stationary point found.

Item	Value	Threshold	Converged?
Maximum Force	0.000000	0.000450	YES
RMS Force	0.000000	0.000300	YES
Maximum Displacement	0.000042	0.001800	YES
RMS Displacement	0.000008	0.001200	YES

Predicted change in Energy=-2.360996D-12

Optimization completed.

SCF Done: E(RB3LYP) = -1470.40656433   A.U. after   6 cycles

Eigenvalues ---   0.00117   0.00120   0.00125   0.00170   0.00233

Standard orientation:

Center Number	Atomic Number	Atomic Type	Coordinates (Angstroms) X	Y	Z
1	6	0	-2.827119	1.882442	1.013192
2	6	0	-2.260494	0.997741	-0.132080
3	7	0	-3.089671	-0.209884	-0.102320
4	6	0	-3.946250	-0.250096	1.082638
5	6	0	-4.164638	1.233392	1.408929
6	6	0	-3.421565	-0.893265	-1.262321
7	6	0	-4.124985	-2.036579	-1.369824

8	6	0	-4.493121	-2.654940	-2.689671
9	6	0	-0.685965	0.703808	-0.032540
10	6	0	0.103781	2.033911	0.001077
11	6	0	1.644065	4.419748	-0.091279
12	6	0	1.381098	2.108190	0.584272
13	6	0	-0.364767	3.187718	-0.650012
14	6	0	0.388725	4.362682	-0.694129
15	6	0	2.137455	3.280419	0.545051
16	6	0	-0.468042	-0.169554	1.221510
17	6	0	-0.311338	-1.779920	3.558501
18	6	0	-0.641438	-1.562972	1.168229
19	6	0	-0.225239	0.389623	2.489430
20	6	0	-0.142619	-0.398823	3.638457
21	6	0	-0.564046	-2.356002	2.314133
22	6	0	-0.276084	-0.029664	-1.352400
23	14	0	1.513661	-0.431318	-1.932881
24	6	0	2.720003	-1.116389	-0.639266
25	6	0	4.588440	-2.192627	1.196699
26	6	0	2.564904	-2.410617	-0.108001
27	6	0	3.847895	-0.383096	-0.226046
28	6	0	4.770992	-0.909466	0.680493
29	6	0	3.481192	-2.944084	0.798503
30	6	0	1.264956	-1.773280	-3.254130
31	6	0	2.282815	1.075542	-2.784149
32	1	0	-2.147699	1.883656	1.868157
33	1	0	-2.943916	2.921917	0.695825
34	1	0	-2.421180	1.486601	-1.102314
35	1	0	-3.460183	-0.779454	1.913783
36	1	0	-4.876057	-0.774732	0.842786
37	1	0	-4.425341	1.409089	2.458023
38	1	0	-4.980438	1.624468	0.789196
39	1	0	-3.053035	-0.413409	-2.169328
40	1	0	-4.455818	-2.556563	-0.472508
41	1	0	-4.070559	-2.087552	-3.527728
42	1	0	-4.129243	-3.689194	-2.773679
43	1	0	-5.581982	-2.695903	-2.840543
44	1	0	2.229711	5.334665	-0.120321
45	1	0	1.797819	1.235233	1.073216

46	1	0	-1.330985	3.186892	-1.142106
47	1	0	-0.012859	5.234636	-1.204239
48	1	0	3.117784	3.297460	1.014891
49	1	0	-0.246718	-2.397334	4.450555
50	1	0	-0.859375	-2.049664	0.225680
51	1	0	-0.082685	1.460044	2.588288
52	1	0	0.054153	0.074811	4.596979
53	1	0	-0.704158	-3.430689	2.228274
54	1	0	-0.838070	-0.965245	-1.437486
55	1	0	-0.649222	0.588490	-2.184655
56	1	0	5.305015	-2.606124	1.901962
57	1	0	1.713473	-3.019172	-0.404233
58	1	0	4.013534	0.617162	-0.618444
59	1	0	5.633496	-0.318868	0.980110
60	1	0	3.332268	-3.945992	1.193534
61	1	0	2.212926	-2.015381	-3.748763
62	1	0	0.867628	-2.702891	-2.829339
63	1	0	0.561672	-1.439967	-4.027600
64	1	0	3.253368	0.819017	-3.225878
65	1	0	1.631932	1.415545	-3.599241
66	1	0	2.429524	1.923030	-2.107878

Alpha occ. eigenvalues -- -0.24464 -0.24014 -0.23548 -0.22885 -0.17305

Alpha virt. eigenvalues -- -0.01000 -0.00266 0.00020 0.00241 0.00750

LUMO E: -0.01000 au = -0.272 eV

HOMO E: -0.17305 au = -4.709 eV

DELTA E: +0.16305 au = +4.437 eV

Zero-point correction= 0.566784 (Hartree/Particle)

Thermal correction to Energy= 0.597650

Thermal correction to Enthalpy= 0.598594

Thermal correction to Gibbs Free Energy= 0.503919

Sum of electronic and zero-point Energies= -1469.839780

Sum of electronic and thermal Energies= -1469.808914

Sum of electronic and thermal Enthalpies= -1469.807970

Sum of electronic and thermal Free Energies= -1469.902645

en_Conf19

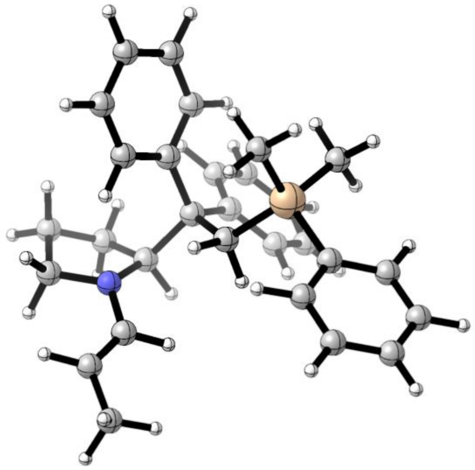

-- Stationary point found.

Item		Value	Threshold	Converged?
Maximum	Force	0.000012	0.000450	YES
RMS	Force	0.000002	0.000300	YES
Maximum	Displacement	0.000940	0.001800	YES
RMS	Displacement	0.000209	0.001200	YES

Predicted change in Energy=-6.303116D-09

Optimization completed.

SCF Done: E(RB3LYP) = -1470.40625165   A.U. after   6 cycles

Eigenvalues ---   0.00059   0.00124   0.00184   0.00417   0.00458

Standard orientation:

Center Number	Atomic Number	Atomic Type	Coordinates (Angstroms) X	Y	Z
1	6	0	3.194643	0.993068	-1.388946
2	6	0	1.664558	1.013661	-1.142247
3	7	0	1.432851	2.374406	-0.624229
4	6	0	2.677041	3.105467	-0.365392
5	6	0	3.779170	2.045380	-0.434768
6	6	0	0.317912	3.107061	-1.021903
7	6	0	0.120217	4.429011	-0.865377
8	6	0	-1.122762	5.134285	-1.332304

9	6	0	1.053522	-0.211738	-0.282054
10	6	0	1.210916	-1.425039	-1.234108
11	6	0	1.558107	-3.500024	-3.153362
12	6	0	0.206491	-1.752365	-2.161260
13	6	0	2.404558	-2.167060	-1.317887
14	6	0	2.576010	-3.188298	-2.252913
15	6	0	0.372101	-2.770919	-3.102510
16	6	0	1.709166	-0.413301	1.105285
17	6	0	2.714812	-0.723428	3.747752
18	6	0	1.893302	0.690007	1.960186
19	6	0	2.004462	-1.679981	1.637566
20	6	0	2.503244	-1.834410	2.933853
21	6	0	2.397650	0.541677	3.252250
22	6	0	-0.448031	0.121277	-0.009782
23	14	0	-1.635428	-1.011831	0.995876
24	6	0	-3.372387	-0.458041	0.457725
25	6	0	-5.944850	0.411831	-0.347738
26	6	0	-4.277403	-1.331300	-0.171983
27	6	0	-3.800068	0.867315	0.670797
28	6	0	-5.066076	1.300530	0.275614
29	6	0	-5.547371	-0.906639	-0.570663
30	6	0	-1.467950	-2.873455	0.695076
31	6	0	-1.458679	-0.677580	2.851594
32	1	0	3.645285	0.010750	-1.255085
33	1	0	3.380385	1.291561	-2.427639
34	1	0	1.142894	0.919221	-2.102667
35	1	0	2.643077	3.617425	0.606097
36	1	0	2.812049	3.883429	-1.134529
37	1	0	3.962764	1.611741	0.552034
38	1	0	4.723733	2.462586	-0.797709
39	1	0	-0.453905	2.509947	-1.500985
40	1	0	0.870143	5.039029	-0.366155
41	1	0	-1.654398	5.622484	-0.502571
42	1	0	-0.897123	5.923463	-2.063968
43	1	0	-1.823478	4.437318	-1.806990
44	1	0	1.688567	-4.295167	-3.882367
45	1	0	-0.731818	-1.210440	-2.164005
46	1	0	3.218451	-1.959271	-0.632736

47	1	0	3.513217	-3.738680	-2.274264
48	1	0	-0.435082	-2.989706	-3.796988
49	1	0	3.105422	-0.841490	4.754941
50	1	0	1.616792	1.678157	1.613132
51	1	0	1.837035	-2.570002	1.043445
52	1	0	2.720882	-2.833229	3.303933
53	1	0	2.530167	1.421569	3.877007
54	1	0	-0.467568	1.071407	0.538253
55	1	0	-0.956113	0.343101	-0.956123
56	1	0	-6.932498	0.745473	-0.656163
57	1	0	-3.991699	-2.364507	-0.355527
58	1	0	-3.135118	1.579939	1.156063
59	1	0	-5.367996	2.329713	0.454507
60	1	0	-6.225659	-1.605800	-1.054029
61	1	0	-2.237804	-3.411040	1.262805
62	1	0	-1.567045	-3.150300	-0.358956
63	1	0	-0.494446	-3.237877	1.038527
64	1	0	-2.216512	-1.239812	3.410763
65	1	0	-0.472440	-0.972615	3.224636
66	1	0	-1.597929	0.384463	3.085646

Alpha occ. eigenvalues -- -0.24452 -0.24183 -0.24017 -0.23342 -0.17838

Alpha virt. eigenvalues -- -0.00780 -0.00438 -0.00264 -0.00014 0.00577

LUMO E: -0.00780 au = -0.212 eV

HOMO E: -0.17838 au = -4.854 eV

DELTA E: +0.17058 au = +4.642 eV

Zero-point correction= 0.567406 (Hartree/Particle)

Thermal correction to Energy= 0.598126

Thermal correction to Enthalpy= 0.599070

Thermal correction to Gibbs Free Energy= 0.504310

Sum of electronic and zero-point Energies= -1469.838846

Sum of electronic and thermal Energies= -1469.808125

Sum of electronic and thermal Enthalpies= -1469.807181

Sum of electronic and thermal Free Energies= -1469.901941

en_Conf12b

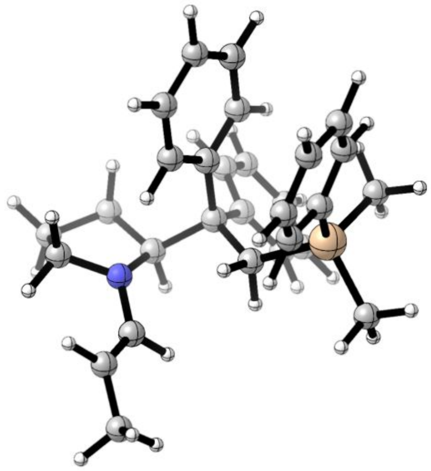

```
   -- Stationary point found.
         Item               Value     Threshold  Converged?
Maximum Force            0.000001     0.000450     YES
RMS     Force            0.000000     0.000300     YES
Maximum Displacement     0.000396     0.001800     YES
RMS     Displacement     0.000058     0.001200     YES
Predicted change in Energy=-9.256895D-11
Optimization completed.

SCF Done:  E(RB3LYP) =  -1470.40541857     A.U. after    7 cycles

    Eigenvalues ---    0.00089   0.00130   0.00169   0.00187   0.00239

                        Standard orientation:
---------------------------------------------------------------------
Center     Atomic      Atomic             Coordinates (Angstroms)
Number     Number       Type             X           Y           Z
---------------------------------------------------------------------
     1          6           0        3.006540    0.983827    1.457600
     2          6           0        2.106852    1.052287    0.196831
     3          7           0        1.511382    2.401480    0.263065
     4          6           0        1.974527    3.141711    1.445588
     5          6           0        3.286804    2.446580    1.821398
     6          6           0        1.225780    3.096012   -0.911235
     7          6           0        0.708861    4.332754   -1.023900
     8          6           0        0.493912    5.018670   -2.344206
```

9	6	0	1.093207	-0.188685	0.033272
10	6	0	2.028994	-1.371757	-0.323808
11	6	0	3.905347	-3.402223	-0.996522
12	6	0	2.797621	-2.025289	0.658480
13	6	0	2.249226	-1.762256	-1.654409
14	6	0	3.167730	-2.761123	-1.988674
15	6	0	3.714420	-3.024394	0.332907
16	6	0	0.236135	-0.439723	1.297779
17	6	0	-1.470016	-0.834705	3.533349
18	6	0	-0.450147	0.631974	1.898745
19	6	0	0.013183	-1.718485	1.833984
20	6	0	-0.824320	-1.914352	2.935392
21	6	0	-1.280574	0.441226	3.002284
22	6	0	0.102744	0.141837	-1.124120
23	14	0	-1.353214	-1.001015	-1.651205
24	6	0	-2.924050	-0.495157	-0.717032
25	6	0	-5.301504	0.289494	0.603840
26	6	0	-3.667402	-1.406302	0.052840
27	6	0	-3.410865	0.823495	-0.803826
28	6	0	-4.581620	1.215258	-0.154632
29	6	0	-4.840872	-1.023075	0.706601
30	6	0	-1.625715	-0.614944	-3.491772
31	6	0	-1.092710	-2.867699	-1.477981
32	1	0	2.465263	0.501005	2.278122
33	1	0	3.916319	0.409044	1.273650
34	1	0	2.736419	1.001957	-0.704608
35	1	0	1.246570	3.084817	2.267768
36	1	0	2.106719	4.196324	1.186678
37	1	0	3.559121	2.579541	2.873847
38	1	0	4.103233	2.846579	1.207511
39	1	0	1.462580	2.539295	-1.817217
40	1	0	0.419410	4.884532	-0.130995
41	1	0	0.770203	4.367429	-3.182033
42	1	0	-0.555745	5.313777	-2.485548
43	1	0	1.090145	5.938307	-2.434988
44	1	0	4.618152	-4.181272	-1.252983
45	1	0	2.674520	-1.758437	1.701716
46	1	0	1.702901	-1.287102	-2.460408

47	1	0	3.302039	-3.033314	-3.032536
48	1	0	4.281080	-3.506917	1.125299
49	1	0	-2.120440	-0.985390	4.390822
50	1	0	-0.342551	1.626694	1.483424
51	1	0	0.493143	-2.582860	1.390933
52	1	0	-0.968852	-2.920593	3.321173
53	1	0	-1.792371	1.295390	3.438321
54	1	0	-0.394894	1.081428	-0.857803
55	1	0	0.667088	0.379824	-2.035311
56	1	0	-6.215125	0.590388	1.110653
57	1	0	-3.326883	-2.433935	0.151393
58	1	0	-2.868660	1.564907	-1.388878
59	1	0	-4.933221	2.240658	-0.240744
60	1	0	-5.393962	-1.749935	1.296713
61	1	0	-2.496432	-1.155531	-3.880977
62	1	0	-1.800159	0.454466	-3.661077
63	1	0	-0.756282	-0.906592	-4.094855
64	1	0	-1.957750	-3.399841	-1.893264
65	1	0	-0.203813	-3.198611	-2.024370
66	1	0	-0.974972	-3.181913	-0.436653

Alpha occ. eigenvalues -- -0.24122 -0.23708 -0.23656 -0.22904 -0.17848

Alpha virt. eigenvalues -- -0.00605 -0.00319 0.00038 0.00447 0.00893

LUMO E: -0.00605 au = -0.165 eV

HOMO E: -0.17848 au = -4.857 eV

DELTA E: +0.17243 au = +4.692 eV

Zero-point correction= 0.567063 (Hartree/Particle)

Thermal correction to Energy= 0.597813

Thermal correction to Enthalpy= 0.598757

Thermal correction to Gibbs Free Energy= 0.504061

Sum of electronic and zero-point Energies= -1469.838355

Sum of electronic and thermal Energies= -1469.807606

Sum of electronic and thermal Enthalpies= -1469.806661

Sum of electronic and thermal Free Energies= -1469.901358

en_Conf14b

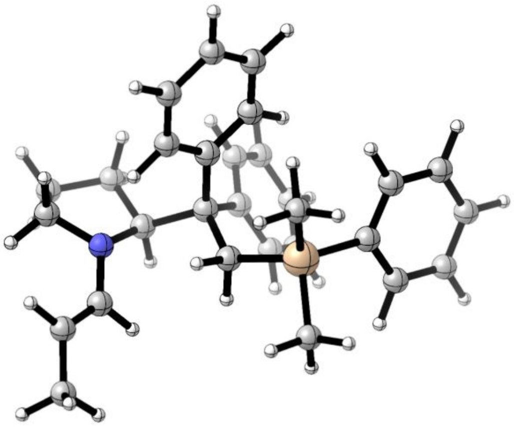

-- Stationary point found.

```
         Item               Value     Threshold  Converged?
Maximum Force            0.000008     0.000450     YES
RMS     Force            0.000002     0.000300     YES
Maximum Displacement     0.001410     0.001800     YES
RMS     Displacement     0.000246     0.001200     YES
Predicted change in Energy=-9.870599D-09
Optimization completed.

SCF Done:  E(RB3LYP) =  -1470.40524800     A.U. after   14 cycles

    Eigenvalues ---    0.00088   0.00137   0.00140   0.00238   0.00270
```

Standard orientation:

Center Number	Atomic Number	Atomic Type	Coordinates (Angstroms) X	Y	Z
1	6	0	2.609932	-2.250712	-0.471288
2	6	0	2.082037	-0.832507	-0.806361
3	7	0	3.219517	0.052018	-0.477163
4	6	0	4.380644	-0.713967	0.000636
5	6	0	4.136278	-2.126919	-0.537719
6	6	0	3.430542	1.216612	-1.215121
7	6	0	4.428380	2.106460	-1.068467
8	6	0	4.599066	3.307003	-1.956907
9	6	0	0.655400	-0.478963	-0.148058

10	6	0	-0.328749	-1.389035	-0.926536
11	6	0	-1.955584	-3.119593	-2.488214
12	6	0	-1.032407	-0.925101	-2.047529
13	6	0	-0.459231	-2.757345	-0.620818
14	6	0	-1.259523	-3.608108	-1.381959
15	6	0	-1.835301	-1.771786	-2.815771
16	6	0	0.613276	-0.710164	1.380469
17	6	0	0.518846	-0.964658	4.206990
18	6	0	1.656408	-0.235785	2.196418
19	6	0	-0.493351	-1.284292	2.027919
20	6	0	-0.540015	-1.412963	3.418706
21	6	0	1.616172	-0.368713	3.584666
22	6	0	0.377162	1.038155	-0.381590
23	14	0	-1.122281	2.031102	0.301499
24	6	0	-2.848544	1.294400	0.021544
25	6	0	-5.459135	0.269398	-0.374762
26	6	0	-3.512363	0.569431	1.028577
27	6	0	-3.542625	1.499126	-1.185854
28	6	0	-4.827896	0.994320	-1.386982
29	6	0	-4.798027	0.059763	0.835996
30	6	0	-0.903458	2.416929	2.141197
31	6	0	-1.025079	3.675106	-0.645763
32	1	0	2.309902	-2.532782	0.543559
33	1	0	2.212656	-3.001443	-1.156876
34	1	0	1.897512	-0.760170	-1.888982
35	1	0	4.440619	-0.713887	1.098832
36	1	0	5.299090	-0.261249	-0.384307
37	1	0	4.652972	-2.902626	0.037435
38	1	0	4.482559	-2.189218	-1.576785
39	1	0	2.680875	1.388034	-1.986470
40	1	0	5.158532	1.978296	-0.270943
41	1	0	3.792368	3.374209	-2.696524
42	1	0	4.600484	4.243955	-1.381657
43	1	0	5.548875	3.280513	-2.510592
44	1	0	-2.582354	-3.779451	-3.082078
45	1	0	-0.969969	0.115942	-2.338740
46	1	0	0.065393	-3.169252	0.233793
47	1	0	-1.336692	-4.656874	-1.106194

48	1	0	-2.370779	-1.366329	-3.670237
49	1	0	0.485563	-1.068550	5.288303
50	1	0	2.502984	0.258230	1.734881
51	1	0	-1.340200	-1.628483	1.446129
52	1	0	-1.412471	-1.867084	3.882321
53	1	0	2.444979	0.005142	4.180887
54	1	0	1.223462	1.584246	0.054684
55	1	0	0.435287	1.262137	-1.455129
56	1	0	-6.461050	-0.124177	-0.526853
57	1	0	-3.020992	0.400406	1.983306
58	1	0	-3.077474	2.069298	-1.987406
59	1	0	-5.338614	1.170589	-2.330698
60	1	0	-5.285152	-0.496786	1.633125
61	1	0	-1.686495	3.107074	2.478357
62	1	0	-0.938598	1.527018	2.776420
63	1	0	0.064548	2.902195	2.314626
64	1	0	-1.797613	4.373236	-0.302779
65	1	0	-0.050716	4.153601	-0.487011
66	1	0	-1.151317	3.548128	-1.727756

Alpha occ. eigenvalues -- -0.24311 -0.23959 -0.23571 -0.23008 -0.17892

Alpha virt. eigenvalues -- -0.00860 -0.00237 -0.00053 0.00240 0.00811

LUMO E: -0.00860 au = -0.234 eV

HOMO E: -0.17892 au = -4.869 eV

DELTA E: +0.17032 au = +4.635 eV

Zero-point correction= 0.566993 (Hartree/Particle)

Thermal correction to Energy= 0.597719

Thermal correction to Enthalpy= 0.598663

Thermal correction to Gibbs Free Energy= 0.504393

Sum of electronic and zero-point Energies= -1469.838255

Sum of electronic and thermal Energies= -1469.807529

Sum of electronic and thermal Enthalpies= -1469.806585

Sum of electronic and thermal Free Energies= -1469.900855

Enamine (S)-16c

```
                              Gibbs Energies
-----------------------------------------------------------------------------------
en_Conf8.log : 1  G298 = -1661.58915800 au = -1042662.93877 Kcal/mol
en_Conf2.log : 2  G298 = -1661.58900200 au = -1042662.84088 Kcal/mol
en_Conf1.log : 3  G298 = -1661.58789200 au = -1042662.14435 Kcal/mol
en_Conf3.log : 4  G298 = -1661.58718500 au = -1042661.70070 Kcal/mol
en_Conf6.log : 5  G298 = -1661.58707000 au = -1042661.62853 Kcal/mol
en_Conf4.log : 6  G298 = -1661.58650900 au = -1042661.27650 Kcal/mol
en_Conf10.log: 7  G298 = -1661.58570000 au = -1042660.76885 Kcal/mol
en_Conf5.log : 8  G298 = -1661.58565700 au = -1042660.74186 Kcal/mol
en_Conf7.log : 9  G298 = -1661.58487000 au = -1042660.24801 Kcal/mol
en_Conf9.log : 10 G298 = -1661.58159300 au = -1042658.19166 Kcal/mol
-----------------------------------------------------------------------------------
```

	G298 (Kcal/mol)	eexp(-Ei/KT)	Ni	Excess (%)	Sum (%)
1	0.00000	1.00000	0.40580	40.58	40.6
2	0.09789	0.84771	0.34400	34.40	75.0
3	0.79443	0.26163	0.10617	10.62	85.6
4	1.23808	0.12373	0.05021	5.02	90.6
5	1.31024	0.10954	0.04445	4.45	95.1
6	1.66227	0.06047	0.02454	2.45	97.5
7	2.16993	0.02567	0.01042	1.04	98.6
8	2.19691	0.02453	0.00995	1.00	99.6
9	2.69076	0.01066	0.00432	0.43	100.0
10	4.74711	0.00033	0.00013	0.01	100.0

en_Conf8

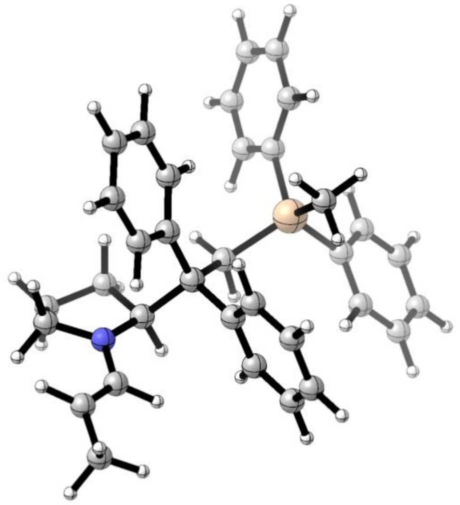

-- Stationary point found.

Item		Value	Threshold	Converged?
Maximum	Force	0.000011	0.000450	YES
RMS	Force	0.000002	0.000300	YES
Maximum	Displacement	0.001169	0.001800	YES
RMS	Displacement	0.000170	0.001200	YES

Predicted change in Energy=-6.372572D-09

Optimization completed.

SCF Done: E(RB3LYP) = -1662.14240191 A.U. after 9 cycles

Eigenvalues --- 0.00048 0.00196 0.00226 0.00320 0.00395

Standard orientation:

Center Number	Atomic Number	Atomic Type	Coordinates (Angstroms) X	Y	Z
1	6	0	1.530917	-1.360198	-2.485923
2	6	0	1.910842	-0.172251	-1.562219
3	7	0	3.351888	-0.343252	-1.370388
4	6	0	3.880066	-1.532160	-2.031188
5	6	0	2.850129	-1.806514	-3.131641
6	6	0	4.185370	0.652887	-0.894898
7	6	0	5.497028	0.543053	-0.603304
8	6	0	6.336231	1.705893	-0.151718
9	6	0	1.011422	-0.101820	-0.227878
10	6	0	1.270012	-1.378721	0.589855
11	6	0	1.827988	-3.787897	1.986871
12	6	0	0.308987	-2.392570	0.719268
13	6	0	2.530357	-1.613190	1.177201
14	6	0	2.803311	-2.796131	1.863673
15	6	0	0.578231	-3.578020	1.410741
16	6	0	1.336475	1.197199	0.557885
17	6	0	1.855276	3.631144	1.933589
18	6	0	1.624921	1.217908	1.930381
19	6	0	1.292163	2.442718	-0.098546

20	6	0	1.553338	3.638604	0.571014
21	6	0	1.881861	2.412677	2.608245
22	6	0	-0.467619	0.021275	-0.736384
23	14	0	-2.024991	0.216115	0.395218
24	6	0	-2.996572	1.733245	-0.212567
25	6	0	-4.427194	4.030067	-1.045103
26	6	0	-4.320267	1.640856	-0.679696
27	6	0	-2.413023	3.015270	-0.171588
28	6	0	-3.115382	4.149135	-0.580959
29	6	0	-5.028264	2.772357	-1.092735
30	6	0	-1.700258	0.422369	2.245447
31	6	0	-3.116697	-1.318745	0.129746
32	6	0	-4.755740	-3.591763	-0.287485
33	6	0	-3.672930	-2.029677	1.208934
34	6	0	-3.410579	-1.785826	-1.167070
35	6	0	-4.218382	-2.904331	-1.377620
36	6	0	-4.480737	-3.151376	1.007508
37	1	0	1.118651	-2.181477	-1.890892
38	1	0	0.777081	-1.074041	-3.224290
39	1	0	1.728813	0.771758	-2.095292
40	1	0	3.970212	-2.382920	-1.338177
41	1	0	4.878595	-1.316559	-2.428138
42	1	0	2.831386	-2.853661	-3.451040
43	1	0	3.077846	-1.189694	-4.009205
44	1	0	3.690323	1.610343	-0.754672
45	1	0	5.998087	-0.419084	-0.693205
46	1	0	6.817594	1.511794	0.817670
47	1	0	5.731646	2.614018	-0.042727
48	1	0	7.146260	1.934144	-0.860402
49	1	0	2.041625	-4.708126	2.524311
50	1	0	-0.672068	-2.279536	0.274483
51	1	0	3.308507	-0.862534	1.105069
52	1	0	3.785981	-2.939303	2.306305
53	1	0	-0.198872	-4.333783	1.490378
54	1	0	2.060537	4.559894	2.458856
55	1	0	1.645598	0.291031	2.489954
56	1	0	1.052489	2.493557	-1.156360
57	1	0	1.518091	4.576838	0.022937

58	1	0	2.103178	2.382160	3.672067
59	1	0	-0.499747	0.885887	-1.410570
60	1	0	-0.693509	-0.838217	-1.378169
61	1	0	-4.976241	4.912022	-1.365605
62	1	0	-4.809232	0.671428	-0.723017
63	1	0	-1.392922	3.135991	0.186510
64	1	0	-2.639630	5.126021	-0.536967
65	1	0	-6.049954	2.669754	-1.450540
66	1	0	-1.143223	1.341530	2.447952
67	1	0	-2.653742	0.488501	2.783102
68	1	0	-1.128478	-0.414473	2.660306
69	1	0	-5.384124	-4.464270	-0.447165
70	1	0	-3.471526	-1.708941	2.227819
71	1	0	-3.007788	-1.265587	-2.034437
72	1	0	-4.428274	-3.239105	-2.390518
73	1	0	-4.894202	-3.681008	1.862309

Alpha occ. eigenvalues --   -0.17000

Alpha virt. eigenvalues --   -0.01310 -0.00893 -0.00563 -0.00362 -0.00129

LUMO E: -0.01310 au = -0.356 eV

HOMO E: -0.17000 au = -4.626 eV

DELTA E: +0.15690 au = +4.269 eV

Zero-point correction= 0.621322 (Hartree/Particle)

Thermal correction to Energy= 0.655197

Thermal correction to Enthalpy= 0.656141

Thermal correction to Gibbs Free Energy= 0.553244

Sum of electronic and zero-point Energies= -1661.521080

Sum of electronic and thermal Energies= -1661.487205

Sum of electronic and thermal Enthalpies= -1661.486261

Sum of electronic and thermal Free Energies= -1661.589158

en_Conf2

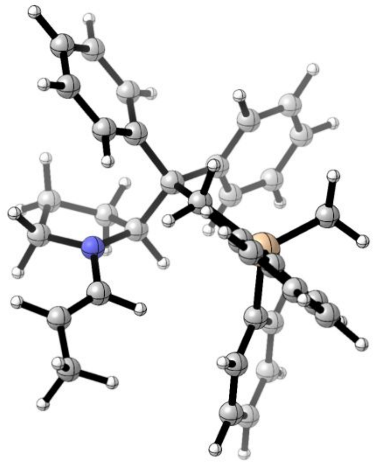

```
--------------------------------------------------------------------------------
   -- Stationary point found.
        Item               Value     Threshold  Converged?
Maximum Force            0.000010     0.000450     YES
RMS     Force            0.000002     0.000300     YES
Maximum Displacement     0.001608     0.001800     YES
RMS     Displacement     0.000299     0.001200     YES
Predicted change in Energy=-1.116292D-08
Optimization completed.

SCF Done:  E(RB3LYP) =  -1662.14315470     A.U. after    9 cycles

    Eigenvalues ---    0.00059   0.00166   0.00279   0.00496   0.00629

                        Standard orientation:
---------------------------------------------------------------------
Center     Atomic      Atomic             Coordinates (Angstroms)
Number     Number       Type             X           Y           Z
---------------------------------------------------------------------
     1          6           0        2.668511   -0.118251    2.175180
     2          6           0        1.387309   -0.328958    1.327768
     3          7           0        1.158586   -1.776631    1.433175
     4          6           0        2.297412   -2.506246    1.993350
     5          6           0        3.414304   -1.464593    2.140489
     6          6           0       -0.113877   -2.302280    1.610344
     7          6           0       -0.423452   -3.582621    1.892348
     8          6           0       -1.833873   -4.062940    2.093643
     9          6           0        1.473848    0.197911   -0.172396
```

10	6	0	1.682377	1.727738	-0.233604
11	6	0	1.993503	4.536763	-0.490109
12	6	0	1.563793	2.579204	0.874702
13	6	0	1.956036	2.328521	-1.476784
14	6	0	2.112618	3.706285	-1.606970
15	6	0	1.716369	3.964795	0.749314
16	6	0	2.643399	-0.531911	-0.880373
17	6	0	4.809707	-1.936229	-2.056875
18	6	0	2.474400	-1.816929	-1.423048
19	6	0	3.932109	0.026229	-0.943629
20	6	0	4.999510	-0.661579	-1.523310
21	6	0	3.539569	-2.509561	-2.001811
22	6	0	0.127879	-0.126866	-0.912844
23	14	0	-1.525025	0.837829	-0.727401
24	6	0	-2.798045	-0.159945	-1.730855
25	6	0	-4.760623	-1.511291	-3.264422
26	6	0	-2.474352	-1.287820	-2.506019
27	6	0	-4.140910	0.268282	-1.750492
28	6	0	-5.110516	-0.393621	-2.503576
29	6	0	-3.439249	-1.956942	-3.263486
30	6	0	-1.461634	2.566626	-1.495291
31	6	0	-2.204560	1.000629	1.041824
32	6	0	-3.248854	1.265523	3.662129
33	6	0	-3.080668	0.040088	1.583775
34	6	0	-1.882863	2.107384	1.851501
35	6	0	-2.393491	2.239540	3.144484
36	6	0	-3.594452	0.165311	2.876204
37	1	0	3.289433	0.706440	1.818954
38	1	0	2.369923	0.117679	3.203893
39	1	0	0.536545	0.188009	1.785369
40	1	0	2.595147	-3.342077	1.346986
41	1	0	2.004251	-2.934569	2.965249
42	1	0	4.096744	-1.506359	1.288996
43	1	0	4.004842	-1.631371	3.047014
44	1	0	-0.911601	-1.570153	1.517336
45	1	0	0.363784	-4.329591	1.971802
46	1	0	-2.552995	-3.241870	1.992068
47	1	0	-1.979821	-4.504609	3.090266

48	1	0	-2.113683	-4.838733	1.365849
49	1	0	2.117257	5.612021	-0.586411
50	1	0	1.348627	2.177559	1.857685
51	1	0	2.061425	1.701351	-2.357242
52	1	0	2.326940	4.131845	-2.583988
53	1	0	1.623041	4.592460	1.632083
54	1	0	5.638105	-2.473013	-2.511599
55	1	0	1.503503	-2.296050	-1.388959
56	1	0	4.109415	1.017367	-0.540276
57	1	0	5.980771	-0.194728	-1.557310
58	1	0	3.370384	-3.502037	-2.412071
59	1	0	0.324931	-0.061767	-1.992950
60	1	0	-0.119212	-1.178155	-0.732219
61	1	0	-5.513303	-2.030510	-3.852352
62	1	0	-1.453465	-1.659377	-2.526970
63	1	0	-4.440281	1.131625	-1.159248
64	1	0	-6.138341	-0.039014	-2.496292
65	1	0	-3.156810	-2.826923	-3.851276
66	1	0	-1.077058	2.519433	-2.520280
67	1	0	-2.474222	2.985169	-1.540640
68	1	0	-0.830067	3.264249	-0.937935
69	1	0	-3.648911	1.366966	4.667792
70	1	0	-3.380147	-0.814105	0.981341
71	1	0	-1.225643	2.883242	1.468095
72	1	0	-2.128390	3.106299	3.745254
73	1	0	-4.268278	-0.593367	3.266423

Alpha occ. eigenvalues --   -0.17118

Alpha virt. eigenvalues --   -0.01188 -0.00963 -0.00687 -0.00434 -0.00056

LUMO E: -0.01188 au = -0.323 eV

HOMO E: -0.17118 au = -4.658 eV

DELTA E: +0.15930 au = +4.335 eV

Zero-point correction=   0.621321 (Hartree/Particle)

Thermal correction to Energy=   0.655151

Thermal correction to Enthalpy=   0.656096

```
Thermal correction to Gibbs Free Energy=         0.554152
Sum of electronic and zero-point Energies=          -1661.521833
Sum of electronic and thermal Energies=             -1661.488003
Sum of electronic and thermal Enthalpies=           -1661.487059
Sum of electronic and thermal Free Energies=        -1661.589002
```

en_Conf1

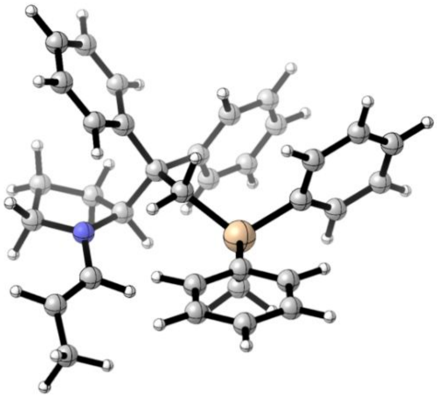

```
---------------------------------------------------------------------------------
    -- Stationary point found.
         Item               Value     Threshold  Converged?
 Maximum Force            0.000007     0.000450     YES
 RMS     Force            0.000001     0.000300     YES
 Maximum Displacement     0.000837     0.001800     YES
 RMS     Displacement     0.000176     0.001200     YES
 Predicted change in Energy=-4.466697D-09
 Optimization completed.

 SCF Done:  E(RB3LYP) =  -1662.14275806     A.U. after    6 cycles

     Eigenvalues ---    0.00129   0.00179   0.00473   0.00521   0.00602

                          Standard orientation:
 ---------------------------------------------------------------------
 Center     Atomic      Atomic             Coordinates (Angstroms)
 Number     Number       Type             X           Y           Z
 ---------------------------------------------------------------------
      1          6           0        3.324584   -0.364249   -1.749767
      2          6           0        2.015823    0.267000   -1.209432
      3          7           0        2.441600    1.618459   -0.812299
      4          6           0        3.898112    1.777942   -0.778157
```

5	6	0	4.464984	0.374155	-1.026630
6	6	0	1.665711	2.729594	-1.110486
7	6	0	2.023458	4.024317	-1.002523
8	6	0	1.120862	5.164513	-1.385483
9	6	0	1.296110	-0.554806	-0.053822
10	6	0	0.775296	-1.915147	-0.573539
11	6	0	-0.277852	-4.414450	-1.405198
12	6	0	0.775576	-2.294858	-1.924193
13	6	0	0.224757	-2.825956	0.347091
14	6	0	-0.290960	-4.054534	-0.056074
15	6	0	0.255469	-3.526958	-2.335672
16	6	0	2.307628	-0.771733	1.099986
17	6	0	4.256680	-1.097384	3.135073
18	6	0	3.081761	-1.941244	1.193617
19	6	0	2.538280	0.228975	2.059307
20	6	0	3.497921	0.070546	3.060863
21	6	0	4.041284	-2.104332	2.194200
22	6	0	0.066941	0.264180	0.477776
23	14	0	-1.625624	0.402142	-0.421606
24	6	0	-2.728108	-1.080387	0.003599
25	6	0	-4.392035	-3.265896	0.684442
26	6	0	-3.298264	-1.899961	-0.985276
27	6	0	-3.023777	-1.386533	1.346444
28	6	0	-3.841712	-2.463598	1.687057
29	6	0	-4.119679	-2.980214	-0.653265
30	6	0	-1.552361	0.583407	-2.308472
31	6	0	-2.478443	1.949323	0.289920
32	6	0	-3.818362	4.262319	1.230419
33	6	0	-1.776397	3.132559	0.591240
34	6	0	-3.874392	1.964899	0.477322
35	6	0	-4.538217	3.102931	0.938992
36	6	0	-2.433956	4.273768	1.055176
37	1	0	3.372404	-1.445056	-1.602788
38	1	0	3.383440	-0.176843	-2.829057
39	1	0	1.281621	0.366370	-2.016006
40	1	0	4.231854	2.196578	0.179826
41	1	0	4.199225	2.485673	-1.566975
42	1	0	4.706910	-0.118864	-0.082753

43	1	0	5.379606	0.409211	-1.627011
44	1	0	0.669940	2.487578	-1.473935
45	1	0	3.006946	4.285956	-0.617743
46	1	0	0.138725	4.806138	-1.715295
47	1	0	1.543468	5.765981	-2.203491
48	1	0	0.954381	5.855927	-0.546792
49	1	0	-0.679400	-5.372572	-1.723919
50	1	0	1.187223	-1.640016	-2.683423
51	1	0	0.208615	-2.570028	1.402142
52	1	0	-0.711949	-4.727803	0.685620
53	1	0	0.277124	-3.787684	-3.390888
54	1	0	5.000672	-1.223119	3.917273
55	1	0	2.930783	-2.744230	0.480293
56	1	0	1.970078	1.150746	2.028363
57	1	0	3.647799	0.865953	3.786702
58	1	0	4.618147	-3.024887	2.236469
59	1	0	-0.204762	-0.144083	1.461210
60	1	0	0.398898	1.285720	0.683949
61	1	0	-5.031834	-4.105188	0.945713
62	1	0	-3.096469	-1.702304	-2.034804
63	1	0	-2.616524	-0.768589	2.144894
64	1	0	-4.053494	-2.675319	2.732365
65	1	0	-4.545519	-3.598133	-1.440093
66	1	0	-1.099685	-0.288176	-2.792665
67	1	0	-2.568523	0.699481	-2.704287
68	1	0	-0.989134	1.472571	-2.612180
69	1	0	-4.331733	5.149423	1.593022
70	1	0	-0.697614	3.178454	0.464675
71	1	0	-4.454358	1.069675	0.267740
72	1	0	-5.616901	3.082126	1.074595
73	1	0	-1.862758	5.171000	1.280632

Alpha occ. eigenvalues --   -0.17790

Alpha virt. eigenvalues --   -0.00777 -0.00589 -0.00393 -0.00033   0.00197

LUMO E: -0.00777 au = -0.211 eV

HOMO E: -0.17790 au = -4.841 eV

```
DELTA E: +0.17013 au = +4.629 eV

 Zero-point correction=                           0.621274 (Hartree/Particle)
 Thermal correction to Energy=                    0.655060
 Thermal correction to Enthalpy=                  0.656005
 Thermal correction to Gibbs Free Energy=         0.554866
 Sum of electronic and zero-point Energies=          -1661.521484
 Sum of electronic and thermal Energies=             -1661.487698
 Sum of electronic and thermal Enthalpies=           -1661.486753
 Sum of electronic and thermal Free Energies=        -1661.587892
```

en_Conf3

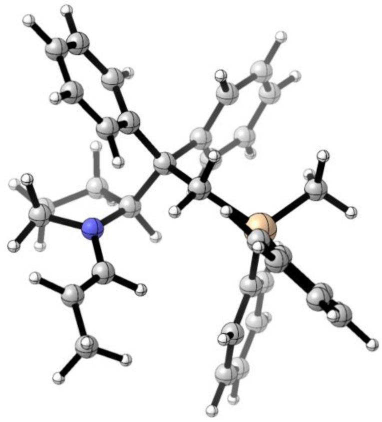

```
---------------------------------------------------------------------------------
    -- Stationary point found.
         Item               Value     Threshold  Converged?
 Maximum Force            0.000010     0.000450     YES
 RMS     Force            0.000002     0.000300     YES
 Maximum Displacement     0.001584     0.001800     YES
 RMS     Displacement     0.000220     0.001200     YES
 Predicted change in Energy=-5.253233D-09
 Optimization completed.

 SCF Done:  E(RB3LYP) =  -1662.14101611     A.U. after    6 cycles

     Eigenvalues ---    0.00075   0.00116   0.00242   0.00506   0.00565

                          Standard orientation:
 ---------------------------------------------------------------------
 Center     Atomic      Atomic             Coordinates (Angstroms)
 Number     Number       Type             X           Y           Z
```

1	6	0	2.653447	0.249764	2.134321
2	6	0	1.393646	-0.172370	1.317705
3	7	0	1.289007	-1.618271	1.549993
4	6	0	2.497508	-2.160818	2.167805
5	6	0	3.038349	-0.977865	2.979817
6	6	0	0.063575	-2.255645	1.673302
7	6	0	-0.160214	-3.577000	1.809580
8	6	0	-1.529151	-4.163656	2.014536
9	6	0	1.451355	0.200372	-0.232766
10	6	0	1.653284	1.712695	-0.469301
11	6	0	1.925141	4.477830	-1.045257
12	6	0	1.470727	2.686034	0.523251
13	6	0	1.962109	2.168378	-1.764895
14	6	0	2.099386	3.524627	-2.051281
15	6	0	1.607018	4.049819	0.241621
16	6	0	2.608379	-0.610719	-0.872424
17	6	0	4.752091	-2.153020	-1.914235
18	6	0	3.909884	-0.088185	-0.973697
19	6	0	2.415835	-1.932159	-1.309959
20	6	0	3.468863	-2.692016	-1.822268
21	6	0	4.966138	-0.843738	-1.486145
22	6	0	0.092854	-0.198182	-0.911357
23	14	0	-1.559470	0.772667	-0.769889
24	6	0	-2.863434	-0.338997	-1.598351
25	6	0	-4.873043	-1.862369	-2.890088
26	6	0	-2.546304	-1.488783	-2.343763
27	6	0	-4.223721	0.021931	-1.522065
28	6	0	-5.216516	-0.724661	-2.156665
29	6	0	-3.534463	-2.242845	-2.981827
30	6	0	-1.533271	2.401041	-1.733892
31	6	0	-2.166287	1.121318	0.998372
32	6	0	-3.093673	1.643225	3.624089
33	6	0	-2.905873	0.163995	1.720210
34	6	0	-1.922680	2.355869	1.630442
35	6	0	-2.376473	2.615650	2.925581
36	6	0	-3.360700	0.415493	3.016024
37	1	0	3.474311	0.520462	1.467024

38	1	0	2.452038	1.121123	2.762543
39	1	0	0.494404	0.306847	1.723981
40	1	0	3.222225	-2.500268	1.415369
41	1	0	2.231484	-3.017905	2.793699
42	1	0	4.116139	-1.040021	3.164181
43	1	0	2.532472	-0.945061	3.951885
44	1	0	-0.784070	-1.572114	1.666487
45	1	0	0.674368	-4.275493	1.778279
46	1	0	-1.776762	-4.907147	1.243195
47	1	0	-2.303980	-3.388351	1.984754
48	1	0	-1.620162	-4.678054	2.982741
49	1	0	2.035672	5.536625	-1.263330
50	1	0	1.208994	2.396870	1.533276
51	1	0	2.109895	1.444667	-2.561016
52	1	0	2.342751	3.836914	-3.063655
53	1	0	1.465963	4.774925	1.039355
54	1	0	5.571117	-2.743262	-2.316729
55	1	0	4.104342	0.934105	-0.667012
56	1	0	1.436465	-2.389913	-1.242885
57	1	0	3.279963	-3.711017	-2.150796
58	1	0	5.956755	-0.400798	-1.552270
59	1	0	0.267492	-0.225811	-1.997019
60	1	0	-0.148227	-1.229784	-0.635157
61	1	0	-5.643879	-2.447879	-3.384855
62	1	0	-1.512394	-1.811045	-2.434335
63	1	0	-4.517221	0.898632	-0.947952
64	1	0	-6.257503	-0.420809	-2.077433
65	1	0	-3.256862	-3.128039	-3.548866
66	1	0	-1.206638	2.226332	-2.765373
67	1	0	-2.544271	2.823729	-1.775014
68	1	0	-0.866003	3.151222	-1.300013
69	1	0	-3.448820	1.843441	4.631805
70	1	0	-3.147646	-0.789508	1.256335
71	1	0	-1.372183	3.131402	1.104765
72	1	0	-2.175049	3.579712	3.386540
73	1	0	-3.928365	-0.344247	3.547474

```
 Alpha  occ. eigenvalues --   -0.17025
 Alpha virt. eigenvalues --   -0.01203  -0.00899  -0.00631  -0.00421   0.00077

LUMO  E: -0.01203 au = -0.327 eV
HOMO  E: -0.17025 au = -4.633 eV
DELTA E: +0.15822 au = +4.305 eV

 Zero-point correction=                           0.621204 (Hartree/Particle)
 Thermal correction to Energy=                    0.655107
 Thermal correction to Enthalpy=                  0.656051
 Thermal correction to Gibbs Free Energy=         0.553831
 Sum of electronic and zero-point Energies=          -1661.519812
 Sum of electronic and thermal Energies=             -1661.485910
 Sum of electronic and thermal Enthalpies=           -1661.484965
 Sum of electronic and thermal Free Energies=        -1661.587185
```

en_Conf6

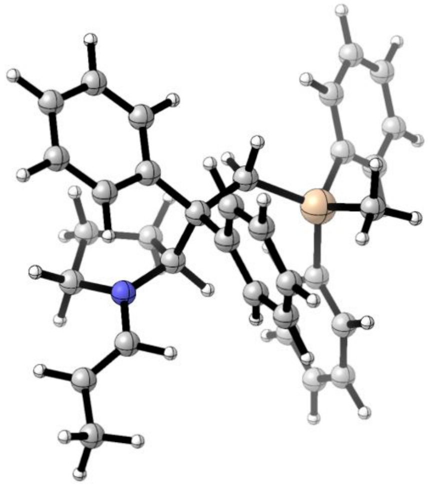

```
--------------------------------------------------------------------------------
    -- Stationary point found.
         Item               Value     Threshold  Converged?
 Maximum Force            0.000013     0.000450     YES
 RMS     Force            0.000003     0.000300     YES
 Maximum Displacement     0.001727     0.001800     YES
 RMS     Displacement     0.000411     0.001200     YES
 Predicted change in Energy=-1.710021D-08
 Optimization completed.

 SCF Done:  E(RB3LYP) =  -1662.14031570     A.U. after    7 cycles
```

Eigenvalues --- 0.00069 0.00115 0.00325 0.00453 0.00556

Standard orientation:

Center Number	Atomic Number	Atomic Type	Coordinates (Angstroms) X	Y	Z
1	6	0	0.633941	-0.258184	-2.421356
2	6	0	1.118073	0.283565	-1.049788
3	7	0	2.524201	0.612992	-1.330804
4	6	0	3.006058	0.090179	-2.606924
5	6	0	1.886136	-0.825184	-3.107748
6	6	0	3.200056	1.675734	-0.755464
7	6	0	4.407670	2.154034	-1.116562
8	6	0	5.067202	3.314422	-0.424110
9	6	0	0.878818	-0.703749	0.185517
10	6	0	1.742845	-1.968802	-0.033079
11	6	0	3.408355	-4.209928	-0.532111
12	6	0	1.207522	-3.204408	-0.424411
13	6	0	3.141593	-1.891981	0.118337
14	6	0	3.960930	-2.991562	-0.129782
15	6	0	2.026689	-4.310868	-0.672663
16	6	0	1.234462	-0.049683	1.548103
17	6	0	1.715991	1.053235	4.121185
18	6	0	1.609306	-0.856278	2.636590
19	6	0	1.077109	1.322505	1.799546
20	6	0	1.324500	1.869642	3.061464
21	6	0	1.846802	-0.318088	3.901363
22	6	0	-0.639936	-1.082718	0.274957
23	14	0	-2.097978	0.091142	0.716129
24	6	0	-2.093902	1.766982	-0.172852
25	6	0	-2.104818	4.265336	-1.505305
26	6	0	-2.253108	1.852499	-1.570497
27	6	0	-1.953298	2.975038	0.534934
28	6	0	-1.956267	4.209648	-0.119295
29	6	0	-2.256669	3.081720	-2.231140
30	6	0	-2.242495	0.344057	2.585560
31	6	0	-3.649071	-0.858456	0.153075

32	6	0	-6.001602	-2.263943	-0.566522
33	6	0	-4.786017	-0.185586	-0.332530
34	6	0	-3.732596	-2.259800	0.268674
35	6	0	-4.889298	-2.956341	-0.085168
36	6	0	-5.947262	-0.874977	-0.687965
37	1	0	-0.167990	-0.995001	-2.351385
38	1	0	0.243557	0.588065	-2.998468
39	1	0	0.565215	1.200255	-0.827304
40	1	0	3.957388	-0.443466	-2.479823
41	1	0	3.192537	0.927293	-3.300442
42	1	0	2.063581	-1.857972	-2.797888
43	1	0	1.804176	-0.812204	-4.199294
44	1	0	2.683752	2.135503	0.079625
45	1	0	4.959890	1.694948	-1.933321
46	1	0	6.038514	3.037394	0.011561
47	1	0	4.442467	3.701062	0.389967
48	1	0	5.262926	4.149518	-1.112839
49	1	0	4.046700	-5.068374	-0.724099
50	1	0	0.137237	-3.325461	-0.544673
51	1	0	3.593006	-0.960723	0.439954
52	1	0	5.036367	-2.895484	-0.002787
53	1	0	1.574328	-5.252201	-0.974746
54	1	0	1.910880	1.477110	5.102581
55	1	0	1.725096	-1.925363	2.492737
56	1	0	0.739343	1.990283	1.015659
57	1	0	1.205517	2.940044	3.210365
58	1	0	2.138638	-0.976664	4.715515
59	1	0	-0.954182	-1.571337	-0.653027
60	1	0	-0.721119	-1.864246	1.044831
61	1	0	-2.107275	5.224231	-2.017107
62	1	0	-2.388141	0.944810	-2.154783
63	1	0	-1.840147	2.956165	1.616026
64	1	0	-1.845662	5.126715	0.454075
65	1	0	-2.381803	3.116931	-3.310518
66	1	0	-1.385450	0.868537	3.017530
67	1	0	-3.153169	0.904642	2.827554
68	1	0	-2.318974	-0.630606	3.082173
69	1	0	-6.903599	-2.803303	-0.844447

70	1	0	-4.764666	0.895682	-0.442662
71	1	0	-2.882706	-2.826709	0.642991
72	1	0	-4.921725	-4.038647	0.014043
73	1	0	-6.808551	-0.326707	-1.061952

Alpha occ. eigenvalues --   -0.16824

Alpha virt. eigenvalues --   -0.01698 -0.00936 -0.00655 -0.00471 -0.00026

LUMO E: -0.01698 au = -0.462 eV

HOMO E: -0.16824 au = -4.578 eV

DELTA E: +0.15126 au = +4.116 eV

Zero-point correction=   0.621161 (Hartree/Particle)

Thermal correction to Energy=   0.655110

Thermal correction to Enthalpy=   0.656054

Thermal correction to Gibbs Free Energy=   0.553246

Sum of electronic and zero-point Energies=   -1661.519155

Sum of electronic and thermal Energies=   -1661.485206

Sum of electronic and thermal Enthalpies=   -1661.484262

Sum of electronic and thermal Free Energies=   -1661.587070

en_Conf4

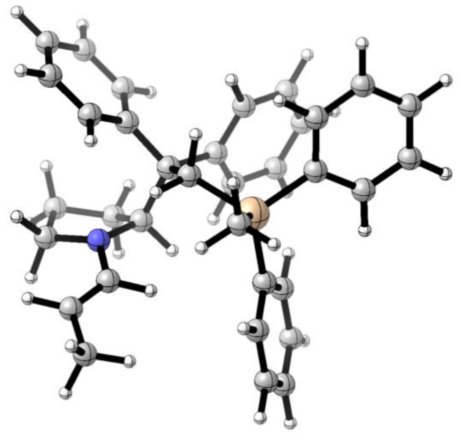

-- Stationary point found.

Item		Value	Threshold	Converged?
Maximum	Force	0.000013	0.000450	YES
RMS	Force	0.000002	0.000300	YES
Maximum	Displacement	0.000685	0.001800	YES
RMS	Displacement	0.000155	0.001200	YES

Predicted change in Energy=-6.644376D-09

Optimization completed.

SCF Done: E(RB3LYP) = -1662.14132557 A.U. after 6 cycles

Eigenvalues --- 0.00099 0.00175 0.00455 0.00522 0.00575

Standard orientation:

Center Number	Atomic Number	Atomic Type	Coordinates (Angstroms) X	Y	Z
1	6	0	-2.677218	0.227678	2.068834
2	6	0	-1.700813	0.580789	0.917330
3	7	0	-2.497509	1.489132	0.078390
4	6	0	-3.920830	1.498176	0.425154
5	6	0	-4.087950	0.393264	1.476324
6	6	0	-1.939568	2.646280	-0.449742
7	6	0	-2.593824	3.652330	-1.061322
8	6	0	-1.905946	4.885676	-1.577783
9	6	0	-1.102372	-0.666273	0.130840
10	6	0	-0.153536	-1.496845	1.025347
11	6	0	1.628968	-3.098898	2.545625
12	6	0	0.432429	-2.659233	0.491093
13	6	0	0.180614	-1.165331	2.346942
14	6	0	1.059396	-1.954924	3.097773
15	6	0	1.308948	-3.447355	1.231833
16	6	0	-2.275283	-1.554946	-0.358975
17	6	0	-4.507625	-3.093822	-1.193150
18	6	0	-2.989027	-1.235901	-1.527203
19	6	0	-2.712712	-2.670284	0.375794
20	6	0	-3.811146	-3.429076	-0.032215
21	6	0	-4.088314	-1.991758	-1.938316
22	6	0	-0.297753	-0.152097	-1.112634
23	14	0	1.456387	0.637351	-1.155388
24	6	0	1.846263	1.973669	0.137041
25	6	0	2.464965	4.010961	2.009485
26	6	0	2.074831	1.678202	1.496112

27	6	0	1.950306	3.323762	-0.251435
28	6	0	2.251658	4.330775	0.668626
29	6	0	2.378918	2.679586	2.420301
30	6	0	1.493478	1.445921	-2.873414
31	6	0	2.817442	-0.686352	-1.149086
32	6	0	4.866224	-2.635594	-1.298504
33	6	0	3.954027	-0.616495	-0.325454
34	6	0	2.746508	-1.759498	-2.059310
35	6	0	3.752241	-2.722373	-2.136735
36	6	0	4.965060	-1.578441	-0.394488
37	1	0	-2.518802	-0.772247	2.477579
38	1	0	-2.527567	0.945047	2.885281
39	1	0	-0.839383	1.138040	1.301874
40	1	0	-4.550146	1.327717	-0.457933
41	1	0	-4.185296	2.489297	0.827215
42	1	0	-4.422494	-0.536789	1.011600
43	1	0	-4.825342	0.668129	2.237269
44	1	0	-0.861426	2.713502	-0.324190
45	1	0	-3.670024	3.592519	-1.210774
46	1	0	-2.038085	5.009413	-2.662813
47	1	0	-0.828612	4.854261	-1.376478
48	1	0	-2.298557	5.800927	-1.111295
49	1	0	2.311423	-3.711875	3.128113
50	1	0	0.189335	-2.958349	-0.523801
51	1	0	-0.236938	-0.283687	2.818033
52	1	0	1.291050	-1.667434	4.120404
53	1	0	1.747116	-4.332107	0.778094
54	1	0	-5.360979	-3.684985	-1.514881
55	1	0	-2.695206	-0.381884	-2.125361
56	1	0	-2.185604	-2.959526	1.278483
57	1	0	-4.117961	-4.286882	0.561119
58	1	0	-4.615897	-1.715213	-2.847859
59	1	0	-0.190389	-0.995772	-1.809673
60	1	0	-0.925488	0.567972	-1.647427
61	1	0	2.700549	4.791856	2.728013
62	1	0	2.020710	0.649741	1.840702
63	1	0	1.797503	3.600730	-1.291071
64	1	0	2.322688	5.363333	0.335989

65	1	0	2.551278	2.418955	3.461794
66	1	0	0.719095	2.213885	-2.987132
67	1	0	2.466197	1.905652	-3.081267
68	1	0	1.318864	0.690095	-3.648617
69	1	0	5.652705	-3.384060	-1.354866
70	1	0	4.057820	0.201645	0.381595
71	1	0	1.891046	-1.850831	-2.726809
72	1	0	3.668811	-3.538347	-2.850650
73	1	0	5.831001	-1.499017	0.258301

Alpha occ. eigenvalues -- -0.17302

Alpha virt. eigenvalues -- -0.01622 -0.00601 -0.00547 -0.00225 0.00053

LUMO E: -0.01622 au = -0.441 eV

HOMO E: -0.17302 au = -4.708 eV

DELTA E: +0.15680 au = +4.267 eV

Zero-point correction= 0.621411 (Hartree/Particle)

Thermal correction to Energy= 0.655171

Thermal correction to Enthalpy= 0.656115

Thermal correction to Gibbs Free Energy= 0.554817

Sum of electronic and zero-point Energies= -1661.519915

Sum of electronic and thermal Energies= -1661.486154

Sum of electronic and thermal Enthalpies= -1661.485210

Sum of electronic and thermal Free Energies= -1661.586509

en_Conf10

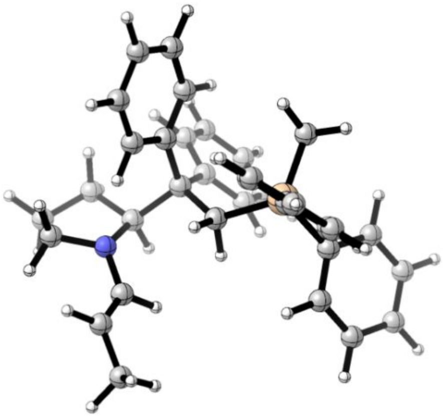

-- Stationary point found.

Item	Value	Threshold	Converged?
Maximum Force	0.000008	0.000450	YES
RMS Force	0.000001	0.000300	YES
Maximum Displacement	0.001495	0.001800	YES
RMS Displacement	0.000198	0.001200	YES

Predicted change in Energy=-2.327400D-09

Optimization completed.

SCF Done: E(RB3LYP) = -1662.13883147 A.U. after 6 cycles

Eigenvalues --- 0.00058 0.00158 0.00251 0.00304 0.00383

Standard orientation:

Center Number	Atomic Number	Atomic Type	Coordinates (Angstroms) X	Y	Z
1	6	0	3.869450	0.598007	0.877304
2	6	0	2.372499	0.353082	1.197748
3	7	0	2.377271	-0.923298	1.938851
4	6	0	3.738884	-1.455919	2.093423
5	6	0	4.634826	-0.226095	1.918775
6	6	0	1.435255	-1.156879	2.940270
7	6	0	1.317230	-2.255209	3.707033
8	6	0	0.299945	-2.388232	4.805576
9	6	0	1.394322	0.461520	-0.079098
10	6	0	1.419689	1.970711	-0.424505
11	6	0	1.551140	4.774268	-0.882036
12	6	0	0.425717	2.842074	0.048605
13	6	0	2.495845	2.557178	-1.118052
14	6	0	2.560647	3.931044	-1.348194
15	6	0	0.484219	4.219827	-0.179553
16	6	0	1.839488	-0.451328	-1.248102
17	6	0	2.558792	-2.225847	-3.349559
18	6	0	1.880376	-0.027845	-2.586366
19	6	0	2.139476	-1.805066	-1.000814
20	6	0	2.503648	-2.674822	-2.029525
21	6	0	2.234991	-0.898318	-3.620789

22	6	0	-0.025737	-0.016205	0.366054
23	14	0	-1.628710	0.115948	-0.706859
24	6	0	-2.379599	-1.622872	-0.848099
25	6	0	-3.500875	-4.200419	-1.173296
26	6	0	-3.747777	-1.858814	-0.619756
27	6	0	-1.589938	-2.719195	-1.245922
28	6	0	-2.140361	-3.991314	-1.407719
29	6	0	-4.304180	-3.130039	-0.778960
30	6	0	-1.444497	0.817628	-2.453851
31	6	0	-2.853703	1.203735	0.259447
32	6	0	-4.665039	2.806581	1.733669
33	6	0	-3.492528	2.312954	-0.323840
34	6	0	-3.154550	0.922999	1.607376
35	6	0	-4.046545	1.709606	2.337434
36	6	0	-4.386104	3.106492	0.400028
37	1	0	4.103954	0.225667	-0.125688
38	1	0	4.119725	1.660201	0.905658
39	1	0	2.009638	1.141132	1.875094
40	1	0	3.962656	-2.219336	1.333994
41	1	0	3.837307	-1.923030	3.077697
42	1	0	5.652775	-0.475499	1.600875
43	1	0	4.700747	0.318674	2.868628
44	1	0	0.738279	-0.333095	3.088407
45	1	0	1.973175	-3.108034	3.539527
46	1	0	-0.354634	-3.257899	4.651738
47	1	0	-0.339491	-1.499557	4.867003
48	1	0	0.769683	-2.521657	5.790942
49	1	0	1.598420	5.844760	-1.062929
50	1	0	-0.420232	2.458607	0.605806
51	1	0	3.297386	1.932931	-1.495721
52	1	0	3.406338	4.340743	-1.894858
53	1	0	-0.313712	4.853741	0.198274
54	1	0	2.839148	-2.902052	-4.152752
55	1	0	1.623625	0.993609	-2.838422
56	1	0	2.078012	-2.181032	0.013398
57	1	0	2.736498	-3.710586	-1.794550
58	1	0	2.253280	-0.528860	-4.643092
59	1	0	0.063146	-1.074710	0.629432

60	1	0	-0.286760	0.481884	1.308771
61	1	0	-3.931060	-5.191378	-1.296047
62	1	0	-4.390135	-1.039603	-0.306931
63	1	0	-0.526963	-2.584052	-1.433655
64	1	0	-1.506607	-4.819799	-1.714991
65	1	0	-5.364398	-3.283845	-0.592558
66	1	0	-0.828740	0.159392	-3.073381
67	1	0	-2.432177	0.883494	-2.926126
68	1	0	-0.992386	1.814842	-2.461665
69	1	0	-5.360176	3.421921	2.299272
70	1	0	-3.289897	2.568981	-1.360787
71	1	0	-2.691317	0.068787	2.098346
72	1	0	-4.260434	1.466469	3.375372
73	1	0	-4.863663	3.958272	-0.078234

Alpha occ. eigenvalues --   -0.17853

Alpha virt. eigenvalues --   -0.01054 -0.00669 -0.00487 -0.00190   0.00017

LUMO E: -0.01054 au = -0.287 eV

HOMO E: -0.17853 au = -4.858 eV

DELTA E: +0.16799 au = +4.571 eV

Zero-point correction=   0.621443 (Hartree/Particle)

Thermal correction to Energy=   0.655261

Thermal correction to Enthalpy=   0.656205

Thermal correction to Gibbs Free Energy=   0.553132

Sum of electronic and zero-point Energies=   -1661.517389

Sum of electronic and thermal Energies=   -1661.483570

Sum of electronic and thermal Enthalpies=   -1661.482626

Sum of electronic and thermal Free Energies=   -1661.585700

en_Conf5

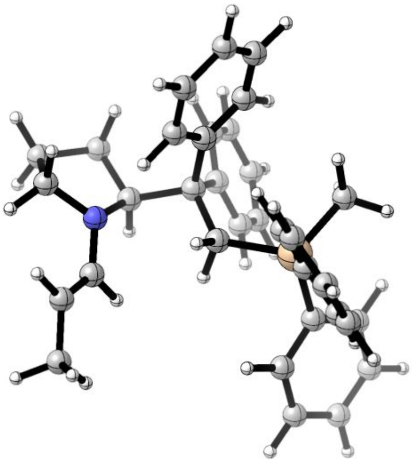

-- Stationary point found.

Item		Value	Threshold	Converged?
Maximum	Force	0.000029	0.000450	YES
RMS	Force	0.000006	0.000300	YES
Maximum	Displacement	0.001384	0.001800	YES
RMS	Displacement	0.000310	0.001200	YES

Predicted change in Energy=-1.324165D-09

Optimization completed.

SCF Done: E(RB3LYP) = -1662.13883125 A.U. after 7 cycles

Eigenvalues --- 0.00055 0.00157 0.00215 0.00271 0.00359

Standard orientation:

Center Number	Atomic Number	Atomic Type	Coordinates (Angstroms) X	Y	Z
1	6	0	-3.870035	0.596272	-0.875728
2	6	0	-2.373020	0.353004	-1.197041
3	7	0	-2.377081	-0.922642	-1.939502
4	6	0	-3.738451	-1.455719	-2.094874
5	6	0	-4.635033	-0.226627	-1.918412
6	6	0	-1.434900	-1.154674	-2.941127
7	6	0	-1.316301	-2.252109	-3.709083
8	6	0	-0.298876	-2.383427	-4.807697
9	6	0	-1.394535	0.460912	0.079586

10	6	0	-1.420406	1.969857	0.426019
11	6	0	-1.553282	4.772975	0.885440
12	6	0	-2.496737	2.555238	1.120201
13	6	0	-0.427002	2.842030	-0.046776
14	6	0	-0.486219	4.219596	0.182329
15	6	0	-2.562241	3.928909	1.351281
16	6	0	-1.839136	-0.452702	1.248136
17	6	0	-2.557732	-2.228577	3.348653
18	6	0	-2.139938	-1.806094	0.999937
19	6	0	-1.878840	-0.030278	2.586760
20	6	0	-2.233076	-0.901437	3.620726
21	6	0	-2.503809	-2.676517	2.028212
22	6	0	0.025670	-0.015930	-0.366076
23	14	0	1.628642	0.116289	0.706786
24	6	0	2.380003	-1.622267	0.847691
25	6	0	3.502843	-4.199007	1.173591
26	6	0	1.589569	-2.720429	1.238747
27	6	0	3.749747	-1.855924	0.626382
28	6	0	4.306936	-3.126743	0.785994
29	6	0	2.140778	-3.992179	1.400881
30	6	0	1.445151	0.818001	2.453862
31	6	0	2.853332	1.204154	-0.259842
32	6	0	4.663655	2.806790	-1.735480
33	6	0	3.490112	2.315313	0.321946
34	6	0	3.155708	0.921324	-1.607004
35	6	0	4.047190	1.707833	-2.337752
36	6	0	4.383200	3.108773	-0.402635
37	1	0	-4.103849	0.221939	0.126703
38	1	0	-4.121130	1.658318	-0.902148
39	1	0	-2.011081	1.142069	-1.873701
40	1	0	-3.961901	-2.220305	-1.336518
41	1	0	-3.836628	-1.921511	-3.079793
42	1	0	-5.652859	-0.477038	-1.600914
43	1	0	-4.701209	0.319565	-2.867431
44	1	0	-0.738277	-0.330431	-3.088301
45	1	0	-1.971841	-3.105436	-3.542555
46	1	0	0.356153	-3.252913	-4.654753
47	1	0	0.340092	-1.494348	-4.868130

48	1	0	-0.768487	-2.516047	-5.793231
49	1	0	-1.601105	5.843323	1.067034
50	1	0	-3.297811	1.930195	1.497553
51	1	0	0.419021	2.459365	-0.604437
52	1	0	0.311262	4.854217	-0.195263
53	1	0	-3.408032	4.337810	1.898388
54	1	0	-2.837847	-2.905292	4.151495
55	1	0	-2.079209	-2.181224	-0.014629
56	1	0	-1.621548	0.990899	2.839417
57	1	0	-2.250411	-0.532812	4.643344
58	1	0	-2.737393	-3.711964	1.792561
59	1	0	-0.062771	-1.074288	-0.630259
60	1	0	0.286376	0.483005	-1.308420
61	1	0	3.933623	-5.189677	1.296575
62	1	0	0.525364	-2.587049	1.420764
63	1	0	4.392704	-1.035248	0.318644
64	1	0	5.368353	-3.278800	0.605060
65	1	0	1.506413	-4.822160	1.702814
66	1	0	0.830856	0.159005	3.074042
67	1	0	2.433331	0.884848	2.924981
68	1	0	0.992113	1.814779	2.462243
69	1	0	5.358432	3.422037	-2.301630
70	1	0	3.286265	2.572913	1.358272
71	1	0	2.694136	0.065519	-2.096755
72	1	0	4.262317	1.463079	-3.375056
73	1	0	4.859213	3.962067	0.074469

Alpha occ. eigenvalues --   -0.17854

Alpha virt. eigenvalues --   -0.01051 -0.00670 -0.00485 -0.00191   0.00015

LUMO E: -0.01051 au = -0.286 eV

HOMO E: -0.17854 au = -4.858 eV

DELTA E: +0.16803 au = +4.572 eV

Zero-point correction= 0.621445 (Hartree/Particle)

Thermal correction to Energy= 0.655261

Thermal correction to Enthalpy= 0.656205

```
Thermal correction to Gibbs Free Energy=         0.553175
Sum of electronic and zero-point Energies=          -1661.517386
Sum of electronic and thermal Energies=             -1661.483570
Sum of electronic and thermal Enthalpies=           -1661.482626
Sum of electronic and thermal Free Energies=        -1661.585657
```

en_Conf7

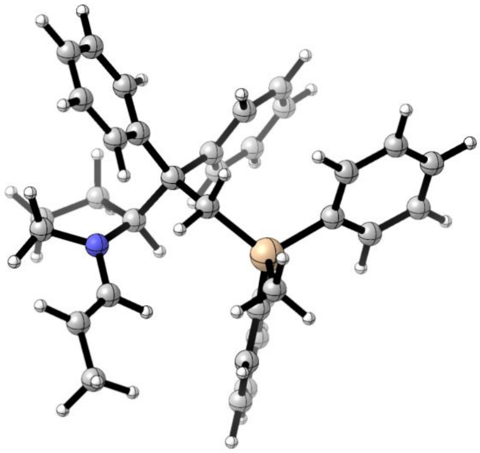

```
--------------------------------------------------------------------------------
    -- Stationary point found.
         Item               Value     Threshold  Converged?
 Maximum Force            0.000009     0.000450     YES
 RMS     Force            0.000002     0.000300     YES
 Maximum Displacement     0.000910     0.001800     YES
 RMS     Displacement     0.000183     0.001200     YES
 Predicted change in Energy=-5.701705D-09
 Optimization completed.

 SCF Done:  E(RB3LYP) =  -1662.13839986     A.U. after    6 cycles

     Eigenvalues ---    0.00050   0.00098   0.00255   0.00354   0.00442

                         Standard orientation:
 ---------------------------------------------------------------------
 Center     Atomic      Atomic             Coordinates (Angstroms)
 Number     Number       Type             X           Y           Z
 ---------------------------------------------------------------------
      1          6           0       -2.514528    0.066091    2.192887
      2          6           0       -1.684870    0.470807    0.935280
      3          7           0       -2.624950    1.254990    0.119433
```

4	6	0	-4.000500	1.114788	0.601849
5	6	0	-3.827757	0.864989	2.104886
6	6	0	-2.210964	2.411293	-0.530579
7	6	0	-2.945414	3.212514	-1.325733
8	6	0	-2.415371	4.485216	-1.925766
9	6	0	-1.043762	-0.746677	0.131047
10	6	0	-0.044818	-1.557023	0.986288
11	6	0	1.854616	-3.098712	2.424488
12	6	0	0.399404	-1.167955	2.257584
13	6	0	0.497336	-2.742231	0.455223
14	6	0	1.429494	-3.501670	1.156886
15	6	0	1.334601	-1.928079	2.969225
16	6	0	-2.195195	-1.666032	-0.358424
17	6	0	-4.378797	-3.275704	-1.195705
18	6	0	-2.901854	-1.385588	-1.540557
19	6	0	-2.617988	-2.779433	0.389030
20	6	0	-3.691359	-3.572909	-0.019880
21	6	0	-3.976241	-2.175915	-1.953267
22	6	0	-0.271403	-0.184041	-1.112296
23	14	0	1.427440	0.718184	-1.145042
24	6	0	1.702958	2.089191	0.141437
25	6	0	2.124238	4.181902	2.007942
26	6	0	1.586898	3.442734	-0.231162
27	6	0	2.050595	1.820983	1.480741
28	6	0	2.257962	2.849356	2.402079
29	6	0	1.789730	4.476535	0.686172
30	6	0	1.423989	1.520088	-2.866959
31	6	0	2.884046	-0.499071	-1.120075
32	6	0	5.094427	-2.263117	-1.280349
33	6	0	2.856187	-1.640213	-1.945630
34	6	0	4.060239	-0.266968	-0.385756
35	6	0	5.150943	-1.137058	-0.459871
36	6	0	3.941753	-2.511584	-2.028676
37	1	0	-2.722671	-1.005854	2.192830
38	1	0	-1.976307	0.287765	3.118022
39	1	0	-0.854276	1.127061	1.222104
40	1	0	-4.515050	0.278840	0.110779
41	1	0	-4.557284	2.031130	0.386733

42	1	0	-4.674508	0.333649	2.552336
43	1	0	-3.724010	1.826614	2.621305
44	1	0	-1.167616	2.664504	-0.345329
45	1	0	-3.977494	2.953401	-1.556255
46	1	0	-2.487959	4.484296	-3.023120
47	1	0	-1.362165	4.640665	-1.662712
48	1	0	-2.970271	5.369544	-1.579176
49	1	0	2.580422	-3.689707	2.976437
50	1	0	0.026429	-0.260976	2.715650
51	1	0	0.172559	-3.082636	-0.523461
52	1	0	1.827765	-4.408344	0.709386
53	1	0	1.649746	-1.596711	3.955646
54	1	0	-5.212403	-3.893451	-1.519298
55	1	0	-2.623656	-0.535110	-2.150630
56	1	0	-2.090540	-3.046694	1.298593
57	1	0	-3.984058	-4.427912	0.584455
58	1	0	-4.497250	-1.927696	-2.874695
59	1	0	-0.112595	-1.020346	-1.808496
60	1	0	-0.936133	0.499799	-1.650007
61	1	0	2.284759	4.983744	2.724092
62	1	0	1.338264	3.701736	-1.257020
63	1	0	2.168048	0.792910	1.810331
64	1	0	2.527740	2.609559	3.427802
65	1	0	1.690358	5.510722	0.366071
66	1	0	0.588109	2.216990	-3.001114
67	1	0	2.356954	2.062164	-3.058123
68	1	0	1.331431	0.747605	-3.639900
69	1	0	5.942684	-2.940386	-1.341062
70	1	0	1.972067	-1.858480	-2.541948
71	1	0	4.132975	0.608255	0.253604
72	1	0	6.045612	-0.931781	0.123003
73	1	0	3.889250	-3.383408	-2.676439

Alpha occ. eigenvalues -- -0.17229

Alpha virt. eigenvalues -- -0.01598 -0.00732 -0.00409 -0.00225 0.00154

LUMO E: -0.01598 au = -0.435 eV

```
HOMO  E: -0.17229 au = -4.688 eV
DELTA E: +0.15631 au = +4.253 eV

 Zero-point correction=                           0.621210 (Hartree/Particle)
 Thermal correction to Energy=                    0.655114
 Thermal correction to Enthalpy=                  0.656058
 Thermal correction to Gibbs Free Energy=         0.553530
 Sum of electronic and zero-point Energies=          -1661.517190
 Sum of electronic and thermal Energies=             -1661.483286
 Sum of electronic and thermal Enthalpies=           -1661.482342
 Sum of electronic and thermal Free Energies=        -1661.584870
```

en_Conf9

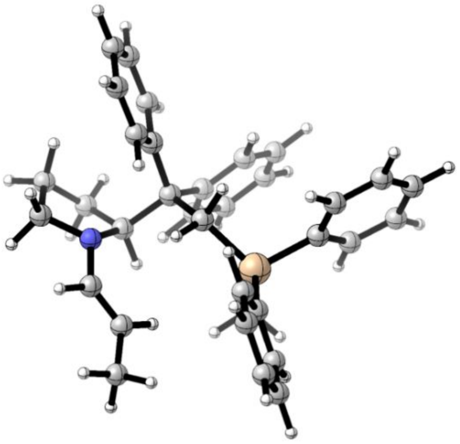

```
--------------------------------------------------------------------------------
    -- Stationary point found.
         Item               Value     Threshold  Converged?
 Maximum Force            0.000002     0.000450     YES
 RMS     Force            0.000000     0.000300     YES
 Maximum Displacement     0.000511     0.001800     YES
 RMS     Displacement     0.000072     0.001200     YES
 Predicted change in Energy=-5.762287D-10
 Optimization completed.

 SCF Done:  E(RB3LYP) =  -1662.13557486     A.U. after    6 cycles

    Eigenvalues ---    0.00093   0.00128   0.00270   0.00353   0.00428

                        Standard orientation:
 ---------------------------------------------------------------------
```

Center Number	Atomic Number	Atomic Type	Coordinates (Angstroms) X	Y	Z
1	6	0	-3.418006	-0.904885	-1.603579
2	6	0	-1.967403	-1.078309	-1.080005
3	7	0	-2.002393	-2.394569	-0.415584
4	6	0	-3.381898	-2.904098	-0.255044
5	6	0	-4.296885	-1.747194	-0.664999
6	6	0	-1.044942	-3.381587	-0.659954
7	6	0	0.119511	-3.319755	-1.326927
8	6	0	1.050006	-4.493066	-1.464035
9	6	0	-1.454244	0.100796	-0.140750
10	6	0	-1.343151	1.432632	-0.918694
11	6	0	-1.059407	3.934438	-2.227230
12	6	0	-0.976580	2.594667	-0.215133
13	6	0	-1.556015	1.563227	-2.299572
14	6	0	-1.416765	2.796142	-2.945977
15	6	0	-0.836563	3.824982	-0.853201
16	6	0	-2.453863	0.258529	1.034552
17	6	0	-4.343255	0.440453	3.141649
18	6	0	-3.490813	1.205929	1.008158
19	6	0	-2.390301	-0.594267	2.150270
20	6	0	-3.319946	-0.506920	3.187474
21	6	0	-4.422115	1.297797	2.044652
22	6	0	-0.038721	-0.269584	0.440347
23	14	0	1.651606	0.106866	-0.401801
24	6	0	2.335745	1.746619	0.277322
25	6	0	3.394607	4.153773	1.327472
26	6	0	2.733187	2.799616	-0.564734
27	6	0	2.493208	1.936381	1.663517
28	6	0	3.011056	3.121479	2.186404
29	6	0	3.256602	3.988446	-0.050857
30	6	0	1.701053	0.188354	-2.293329
31	6	0	2.838150	-1.261708	0.182704
32	6	0	4.671837	-3.231759	1.071888
33	6	0	3.936248	-1.652457	-0.605802
34	6	0	2.690673	-1.892720	1.432487
35	6	0	3.592005	-2.862110	1.875374

36	6	0	4.841579	-2.623449	-0.172618
37	1	0	-3.740404	0.137311	-1.644740
38	1	0	-3.470968	-1.305461	-2.623675
39	1	0	-1.269632	-1.136574	-1.922920
40	1	0	-3.559083	-3.229108	0.777702
41	1	0	-3.539706	-3.772943	-0.910567
42	1	0	-4.601179	-1.164088	0.206148
43	1	0	-5.205163	-2.106108	-1.159578
44	1	0	-1.324442	-4.332844	-0.206622
45	1	0	0.442626	-2.391186	-1.786132
46	1	0	1.231162	-4.746926	-2.518474
47	1	0	0.640665	-5.385937	-0.976538
48	1	0	2.031319	-4.287766	-1.015605
49	1	0	-0.954150	4.892872	-2.728530
50	1	0	-0.801776	2.535773	0.854732
51	1	0	-1.828879	0.704240	-2.901020
52	1	0	-1.592405	2.858006	-4.017073
53	1	0	-0.545727	4.697133	-0.274108
54	1	0	-5.064803	0.512558	3.951198
55	1	0	-3.573422	1.890464	0.171021
56	1	0	-1.612178	-1.345797	2.207703
57	1	0	-3.239568	-1.182637	4.035483
58	1	0	-5.208613	2.046500	1.991268
59	1	0	0.057302	0.235369	1.411180
60	1	0	-0.038868	-1.338962	0.670343
61	1	0	3.801281	5.077977	1.730467
62	1	0	2.627012	2.700744	-1.641863
63	1	0	2.216594	1.141717	2.354338
64	1	0	3.119976	3.238135	3.261961
65	1	0	3.554487	4.785562	-0.727899
66	1	0	1.069868	0.995445	-2.675202
67	1	0	2.725408	0.377619	-2.635985
68	1	0	1.371023	-0.744859	-2.761239
69	1	0	5.374420	-3.988157	1.412438
70	1	0	4.093510	-1.195532	-1.579952
71	1	0	1.853630	-1.632697	2.076688
72	1	0	3.449088	-3.331340	2.845805
73	1	0	5.678308	-2.904985	-0.807425

```
 -----------------------------------------------------------------------

 Alpha  occ. eigenvalues --   -0.17778
 Alpha virt. eigenvalues --   -0.01296  -0.00363  -0.00155   0.00217   0.00386

LUMO  E: -0.01296 au = -0.353 eV
HOMO  E: -0.17778 au = -4.838 eV
DELTA E: +0.16482 au = +4.485 eV

 Zero-point correction=                           0.621294 (Hartree/Particle)
 Thermal correction to Energy=                    0.655159
 Thermal correction to Enthalpy=                  0.656103
 Thermal correction to Gibbs Free Energy=         0.553982
 Sum of electronic and zero-point Energies=          -1661.514281
 Sum of electronic and thermal Energies=             -1661.480416
 Sum of electronic and thermal Enthalpies=           -1661.479472
 Sum of electronic and thermal Free Energies=        -1661.581593
```

Enamine (S)-16d

```
                                 Gibbs Energies
--------------------------------------------------------------------------------
en_Conf11b.log: 1 G298 = -1278.22222400 au = -802096.55562 Kcal/mol
en_Conf10b.log: 2 G298 = -1278.22206800 au = -802096.45772 Kcal/mol
en_Conf12b.log: 3 G298 = -1278.22205300 au = -802096.44831 Kcal/mol
en_Conf2b.log : 4 G298 = -1278.22040500 au = -802095.41418 Kcal/mol
en_Conf1.log  : 5 G298 = -1278.22025300 au = -802095.31880 Kcal/mol
en_Conf3b.log : 6 G298 = -1278.21918800 au = -802094.65050 Kcal/mol
en_Conf4b.log : 7 G298 = -1278.21773600 au = -802093.73935 Kcal/mol
en_Conf8b.log : 8 G298 = -1278.21767800 au = -802093.70296 Kcal/mol
en_Conf9b.log : 9 G298 = -1278.21589600 au = -802092.58474 Kcal/mol
en_Conf7.log  : 10      G298 = -1278.21325400 au = -802090.92686 Kcal/mol
en_Conf6b.log : 11      G298 = -1278.21271400 au = -802090.58800 Kcal/mol
--------------------------------------------------------------------------------
     G298 (Kcal/mol)  eexp(-Ei/KT)        Ni         Excess (%)      Sum (%)
     -------------- -------------- -------------- -------------- --------------
 1      0.00000        1.00000        0.33224        33.22           33.2
 2      0.09789        0.84771        0.28164        28.16           61.4
 3      0.10730        0.83434        0.27720        27.72           89.1
```

4	1.14144	0.14565	0.04839	4.84	93.9
5	1.23682	0.12399	0.04120	4.12	98.1
6	1.90512	0.04014	0.01333	1.33	99.4
7	2.81626	0.00862	0.00286	0.29	99.7
8	2.85266	0.00811	0.00269	0.27	100.0
9	3.97088	0.00123	0.00041	0.04	100.0
10	5.62876	0.00007	0.00002	0.00	100.0
11	5.96762	0.00004	0.00001	0.00	100.0

en_Conf11b

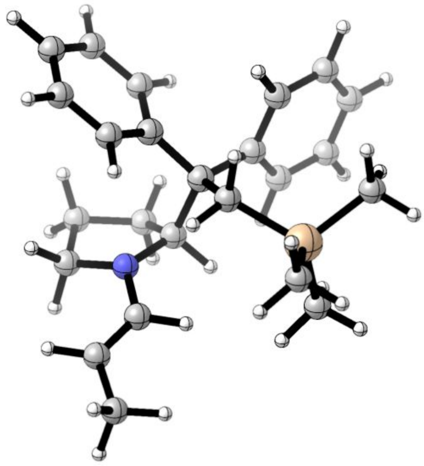

-- Stationary point found.

Item	Value	Threshold	Converged?
Maximum Force	0.000001	0.000450	YES
RMS Force	0.000000	0.000300	YES
Maximum Displacement	0.000273	0.001800	YES
RMS Displacement	0.000057	0.001200	YES

Predicted change in Energy=-9.264161D-11

Optimization completed.

SCF Done: E(RB3LYP) = -1278.67814322 A.U. after 6 cycles

Eigenvalues --- 0.00110 0.00127 0.00145 0.00173 0.00214

Standard orientation:

Center Number	Atomic Number	Atomic Type	Coordinates (Angstroms) X	Y	Z

1	6	0	0.722694	1.219410	2.247351
2	6	0	0.707014	0.109800	1.165129
3	7	0	2.120806	0.016832	0.766573
4	6	0	2.943066	1.105490	1.297446
5	6	0	1.949423	2.090524	1.924143
6	6	0	-0.289016	0.360318	-0.048607
7	6	0	0.073315	1.711357	-0.712598
8	6	0	0.843344	4.201415	-1.831877
9	6	0	-0.567248	2.908668	-0.348783
10	6	0	1.111420	1.799949	-1.655257
11	6	0	1.492113	3.024935	-2.206366
12	6	0	-0.191161	4.135360	-0.898533
13	6	0	-1.763652	0.364364	0.415366
14	6	0	-4.515845	0.296050	1.104185
15	6	0	-2.187654	-0.036050	1.691230
16	6	0	-2.763262	0.728687	-0.506320
17	6	0	-4.114865	0.699585	-0.171822
18	6	0	-3.544430	-0.072291	2.031454
19	6	0	-0.136807	-0.805738	-1.085037
20	14	0	-0.828501	-2.585907	-0.846791
21	6	0	-0.631192	-3.350088	0.881426
22	6	0	-2.659169	-2.699178	-1.321200
23	6	0	0.162923	-3.640417	-2.077051
24	6	0	2.726799	-1.207700	0.524436
25	6	0	4.044539	-1.449369	0.385043
26	6	0	4.605736	-2.820548	0.129263
27	1	0	-0.202010	1.799518	2.279033
28	1	0	0.847935	0.748788	3.230327
29	1	0	0.412778	-0.846759	1.609655
30	1	0	3.546828	1.569191	0.506762
31	1	0	3.642698	0.699126	2.046193
32	1	0	1.686957	2.878437	1.214906
33	1	0	2.363280	2.570136	2.816846
34	1	0	1.136361	5.154594	-2.264070
35	1	0	-1.379253	2.887061	0.370239
36	1	0	1.639865	0.906221	-1.963994
37	1	0	2.299440	3.054721	-2.933919
38	1	0	-0.712941	5.039674	-0.595251

39	1	0	-5.569296	0.272217	1.369624
40	1	0	-1.466722	-0.325298	2.447099
41	1	0	-2.473718	1.051668	-1.502013
42	1	0	-4.856663	0.991419	-0.910905
43	1	0	-3.834135	-0.386685	3.030913
44	1	0	-0.601348	-0.476981	-2.026159
45	1	0	0.924648	-0.926595	-1.325203
46	1	0	0.408513	-3.393415	1.224627
47	1	0	-1.215486	-2.812641	1.635976
48	1	0	-1.005403	-4.381875	0.855796
49	1	0	-3.307170	-2.189307	-0.601580
50	1	0	-2.848135	-2.258796	-2.307692
51	1	0	-2.968759	-3.751227	-1.366345
52	1	0	1.232721	-3.651499	-1.834896
53	1	0	-0.187340	-4.679953	-2.077474
54	1	0	0.063011	-3.260211	-3.101319
55	1	0	2.029264	-2.037301	0.444699
56	1	0	4.758431	-0.630313	0.438793
57	1	0	5.163733	-2.867590	-0.817299
58	1	0	5.305279	-3.133309	0.918033
59	1	0	3.811493	-3.575345	0.079528

Alpha occ. eigenvalues -- -0.24625 -0.24159 -0.23140 -0.17506

Alpha virt. eigenvalues -- -0.00672 -0.00226 0.00267 0.00968 0.05238

LUMO E: -0.00672 au = -0.183 eV

HOMO E: -0.17506 au = -4.764 eV

DELTA E: +0.16834 au = +4.581 eV

Zero-point correction= 0.512906 (Hartree/Particle)

Thermal correction to Energy= 0.540598

Thermal correction to Enthalpy= 0.541542

Thermal correction to Gibbs Free Energy= 0.455919

Sum of electronic and zero-point Energies= -1278.165237

Sum of electronic and thermal Energies= -1278.137546

Sum of electronic and thermal Enthalpies= -1278.136601

Sum of electronic and thermal Free Energies= -1278.222224

en_Conf10b

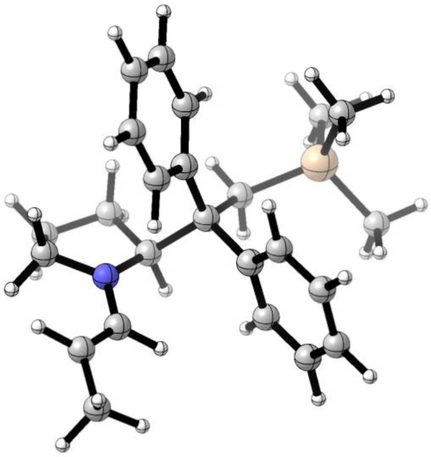

```
   -- Stationary point found.
        Item               Value     Threshold  Converged?
Maximum Force            0.000000     0.000450     YES
RMS     Force            0.000000     0.000300     YES
Maximum Displacement     0.000111     0.001800     YES
RMS     Displacement     0.000019     0.001200     YES
Predicted change in Energy=-9.765610D-12
Optimization completed.

SCF Done:  E(RB3LYP) =  -1278.67716060     A.U. after    6 cycles

    Eigenvalues ---    0.00054   0.00118   0.00121   0.00150   0.00216

                        Standard orientation:
---------------------------------------------------------------------
Center     Atomic      Atomic             Coordinates (Angstroms)
Number     Number       Type             X           Y           Z
---------------------------------------------------------------------
     1          6           0        0.960425   -1.699278   -2.239184
     2          6           0        0.989586   -0.339865   -1.492085
     3          7           0        2.356385   -0.271991   -0.970458
     4          6           0        3.147153   -1.457138   -1.285445
     5          6           0        2.432599   -2.039033   -2.509614
     6          6           0       -0.195923   -0.191339   -0.418549
     7          6           0       -0.239945    1.249741    0.154735
     8          6           0       -0.469526    3.911203    1.123356
```

9	6	0	-0.208157	2.357260	-0.713599
10	6	0	-0.415443	1.520760	1.520354
11	6	0	-0.528120	2.828921	1.998875
12	6	0	-0.311967	3.665294	-0.241408
13	6	0	0.019159	-1.261131	0.667970
14	6	0	0.562142	-3.292443	2.577682
15	6	0	1.101413	-1.157526	1.564723
16	6	0	-0.772548	-2.416135	0.758411
17	6	0	-0.510399	-3.417175	1.698611
18	6	0	1.367107	-2.154144	2.503514
19	6	0	-1.527139	-0.388847	-1.217844
20	14	0	-3.282809	-0.015130	-0.513822
21	6	0	-4.451370	-1.084719	-1.564368
22	6	0	-3.571740	-0.429410	1.312210
23	6	0	-3.742701	1.802303	-0.790894
24	6	0	2.946245	0.894503	-0.518648
25	6	0	4.161865	1.023274	0.049949
26	6	0	4.742426	2.350856	0.451203
27	1	0	0.520588	-2.470641	-1.599102
28	1	0	0.362919	-1.646285	-3.153264
29	1	0	0.841850	0.469667	-2.220956
30	1	0	3.167250	-2.172564	-0.448723
31	1	0	4.181004	-1.161826	-1.497921
32	1	0	2.602738	-3.113295	-2.635983
33	1	0	2.785588	-1.532262	-3.415799
34	1	0	-0.550819	4.928967	1.495183
35	1	0	-0.106835	2.208115	-1.784241
36	1	0	-0.464322	0.702398	2.228702
37	1	0	-0.660094	2.995242	3.064999
38	1	0	-0.275845	4.492551	-0.945748
39	1	0	0.769033	-4.068227	3.310173
40	1	0	1.744799	-0.285924	1.535111
41	1	0	-1.611594	-2.556673	0.087380
42	1	0	-1.150897	-4.294777	1.736171
43	1	0	2.209664	-2.036427	3.180390
44	1	0	-1.467002	0.256787	-2.106990
45	1	0	-1.561615	-1.405919	-1.625547
46	1	0	-4.332695	-0.879034	-2.635576

47	1	0	-4.269418	-2.156256	-1.413354
48	1	0	-5.500132	-0.891063	-1.307754
49	1	0	-3.379103	-1.480696	1.547665
50	1	0	-2.944409	0.179384	1.971053
51	1	0	-4.618807	-0.216866	1.564679
52	1	0	-3.607371	2.098422	-1.838441
53	1	0	-4.797532	1.967650	-0.536961
54	1	0	-3.140143	2.477602	-0.175078
55	1	0	2.328924	1.779037	-0.652482
56	1	0	4.774298	0.142838	0.235939
57	1	0	5.011214	2.375464	1.517162
58	1	0	5.660888	2.588462	-0.106016
59	1	0	4.031656	3.166306	0.272340

Alpha occ. eigenvalues -- -0.24269 -0.24255 -0.23358 -0.16966

Alpha virt. eigenvalues -- -0.00569 -0.00286 0.00264 0.00930 0.05742

LUMO E: -0.00569 au = -0.155 eV

HOMO E: -0.16966 au = -4.617 eV

DELTA E: +0.16397 au = +4.462 eV

Zero-point correction= 0.512743 (Hartree/Particle)

Thermal correction to Energy= 0.540511

Thermal correction to Enthalpy= 0.541455

Thermal correction to Gibbs Free Energy= 0.455093

Sum of electronic and zero-point Energies= -1278.164417

Sum of electronic and thermal Energies= -1278.136650

Sum of electronic and thermal Enthalpies= -1278.135706

Sum of electronic and thermal Free Energies= -1278.222068

en_Conf12b

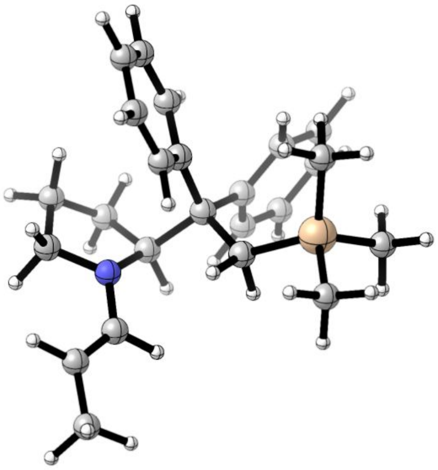

-- Stationary point found.

Item		Value	Threshold	Converged?
Maximum	Force	0.000002	0.000450	YES
RMS	Force	0.000000	0.000300	YES
Maximum	Displacement	0.000471	0.001800	YES
RMS	Displacement	0.000095	0.001200	YES

Predicted change in Energy=-2.848934D-10

Optimization completed.

SCF Done: E(RB3LYP) = -1278.67673518 A.U. after 6 cycles

Eigenvalues --- 0.00062 0.00096 0.00122 0.00150 0.00226

Standard orientation:

Center Number	Atomic Number	Atomic Type	Coordinates (Angstroms) X	Y	Z
1	6	0	1.190533	-2.122686	-1.616598
2	6	0	1.033630	-0.615386	-1.302894
3	7	0	2.357113	-0.235154	-0.793396
4	6	0	3.219639	-1.387072	-0.521118
5	6	0	2.316177	-2.616020	-0.689172
6	6	0	-0.181930	-0.237087	-0.330890
7	6	0	0.041898	-0.960692	1.012353
8	6	0	0.634544	-2.360359	3.411210
9	6	0	0.894309	-0.416835	1.988401

10	6	0	-0.492950	-2.235015	1.272257
11	6	0	-0.207508	-2.923498	2.452501
12	6	0	1.186113	-1.102703	3.169165
13	6	0	-1.530051	-0.614540	-0.995011
14	6	0	-4.061258	-1.146305	-2.178495
15	6	0	-2.691198	-0.776605	-0.217776
16	6	0	-1.685695	-0.716973	-2.387711
17	6	0	-2.927982	-0.979547	-2.970895
18	6	0	-3.933808	-1.041258	-0.793073
19	6	0	-0.162446	1.320122	-0.177165
20	14	0	-1.394318	2.394942	0.834715
21	6	0	-2.956315	2.786106	-0.165625
22	6	0	-1.876346	1.731456	2.544291
23	6	0	-0.461255	4.030069	1.095811
24	6	0	2.952430	0.963415	-1.165905
25	6	0	4.231654	1.331275	-0.962893
26	6	0	4.788441	2.652159	-1.416238
27	1	0	0.261626	-2.683295	-1.488541
28	1	0	1.494853	-2.233101	-2.664515
29	1	0	0.860143	-0.051836	-2.225921
30	1	0	3.657944	-1.327925	0.483349
31	1	0	4.053682	-1.391398	-1.241555
32	1	0	1.912361	-2.932363	0.274933
33	1	0	2.862568	-3.463597	-1.115010
34	1	0	0.857177	-2.893095	4.331900
35	1	0	1.353403	0.551117	1.829022
36	1	0	-1.147152	-2.702610	0.544580
37	1	0	-0.645947	-3.904533	2.617723
38	1	0	1.849390	-0.647382	3.900342
39	1	0	-5.027059	-1.354033	-2.631034
40	1	0	-2.622569	-0.705166	0.861829
41	1	0	-0.836435	-0.596146	-3.049913
42	1	0	-3.001757	-1.054518	-4.052867
43	1	0	-4.803287	-1.165024	-0.152300
44	1	0	0.829676	1.621669	0.175866
45	1	0	-0.233281	1.739491	-1.193242
46	1	0	-2.699313	3.229110	-1.135897
47	1	0	-3.565675	1.898307	-0.359490

48	1	0	-3.579369	3.512865	0.371093
49	1	0	-2.473709	0.815274	2.497144
50	1	0	-0.997763	1.516290	3.161394
51	1	0	-2.476028	2.487191	3.068142
52	1	0	-0.138450	4.466951	0.142607
53	1	0	-1.097264	4.770382	1.596687
54	1	0	0.434303	3.892625	1.714303
55	1	0	2.278016	1.651496	-1.671514
56	1	0	4.912124	0.668161	-0.432916
57	1	0	5.173108	3.247445	-0.575257
58	1	0	5.626386	2.527473	-2.117417
59	1	0	4.025204	3.254938	-1.922929

Alpha occ. eigenvalues -- -0.24588 -0.24342 -0.23258 -0.17505

Alpha virt. eigenvalues -- -0.00725 -0.00143 0.00026 0.00785 0.05275

LUMO E: -0.00725 au = -0.197 eV

HOMO E: -0.17505 au = -4.763 eV

DELTA E: +0.16780 au = +4.566 eV

Zero-point correction= 0.512658 (Hartree/Particle)

Thermal correction to Energy= 0.540551

Thermal correction to Enthalpy= 0.541495

Thermal correction to Gibbs Free Energy= 0.454682

Sum of electronic and zero-point Energies= -1278.164078

Sum of electronic and thermal Energies= -1278.136184

Sum of electronic and thermal Enthalpies= -1278.135240

Sum of electronic and thermal Free Energies= -1278.222053

en_Conf2b

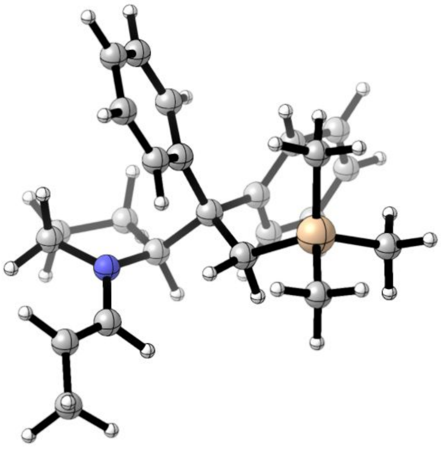

```
-------------------------------------------------------------------------------
   -- Stationary point found.
        Item               Value     Threshold  Converged?
 Maximum Force            0.000000     0.000450     YES
 RMS     Force            0.000000     0.000300     YES
 Maximum Displacement     0.000016     0.001800     YES
 RMS     Displacement     0.000003     0.001200     YES
 Predicted change in Energy=-7.150681D-13
 Optimization completed.

 SCF Done:  E(RB3LYP) =  -1278.67506750     A.U. after    6 cycles

    Eigenvalues ---    0.00083   0.00107   0.00122   0.00128   0.00229

                         Standard orientation:
 ---------------------------------------------------------------------
 Center     Atomic      Atomic             Coordinates (Angstroms)
 Number     Number       Type             X           Y           Z
 ---------------------------------------------------------------------
      1          6           0        1.065611   -2.407465   -1.278364
      2          6           0        0.970612   -0.857271   -1.216637
      3          7           0        2.314660   -0.424021   -0.824913
      4          6           0        3.145686   -1.555401   -0.412578
      5          6           0        2.569117   -2.727791   -1.217883
      6          6           0       -0.191188   -0.294729   -0.263645
      7          6           0        0.196503   -0.654160    1.186301
      8          6           0        1.106357   -1.385585    3.778713
```

9	6	0	1.047198	0.180961	1.931181
10	6	0	-0.170599	-1.875081	1.781447
11	6	0	0.270862	-2.235499	3.056136
12	6	0	1.493581	-0.174766	3.205091
13	6	0	-1.559951	-0.871715	-0.698903
14	6	0	-4.128589	-1.754069	-1.538998
15	6	0	-1.838676	-1.152309	-2.047906
16	6	0	-2.620382	-1.027210	0.210655
17	6	0	-3.881442	-1.464839	-0.197321
18	6	0	-3.097857	-1.589439	-2.462824
19	6	0	-0.261527	1.251504	-0.494764
20	14	0	-1.532686	2.448480	0.310001
21	6	0	-3.160543	2.466316	-0.661126
22	6	0	-1.892569	2.163660	2.149142
23	6	0	-0.733995	4.163540	0.130614
24	6	0	2.875394	0.740529	-1.329850
25	6	0	4.073295	1.274964	-1.026090
26	6	0	4.615380	2.509741	-1.690436
27	1	0	0.555577	-2.858141	-0.424455
28	1	0	0.588531	-2.801545	-2.179534
29	1	0	0.770567	-0.451154	-2.216862
30	1	0	3.073708	-1.734467	0.669021
31	1	0	4.192220	-1.346866	-0.653722
32	1	0	2.775717	-3.703671	-0.765663
33	1	0	3.004182	-2.724135	-2.224480
34	1	0	1.450622	-1.661724	4.771838
35	1	0	1.387360	1.121286	1.515523
36	1	0	-0.826831	-2.557241	1.252294
37	1	0	-0.043403	-3.185873	3.480256
38	1	0	2.150208	0.501167	3.747119
39	1	0	-5.108452	-2.097427	-1.859466
40	1	0	-1.071481	-1.026586	-2.803914
41	1	0	-2.461561	-0.806236	1.259800
42	1	0	-4.671511	-1.576716	0.541037
43	1	0	-3.268839	-1.799721	-3.515585
44	1	0	0.722898	1.688375	-0.295152
45	1	0	-0.415109	1.403033	-1.574550
46	1	0	-2.984316	2.683281	-1.721997

47	1	0	-3.691688	1.511229	-0.602719
48	1	0	-3.827505	3.246990	-0.273635
49	1	0	-2.418067	1.221521	2.335306
50	1	0	-0.977266	2.152314	2.749948
51	1	0	-2.529867	2.975041	2.524079
52	1	0	-0.488470	4.386592	-0.915173
53	1	0	-1.410235	4.951878	0.483700
54	1	0	0.194222	4.241581	0.710072
55	1	0	2.239207	1.249452	-2.054491
56	1	0	4.698715	0.809116	-0.266551
57	1	0	4.860133	3.294336	-0.959964
58	1	0	5.540068	2.308284	-2.251157
59	1	0	3.891983	2.932135	-2.398279

Alpha occ. eigenvalues -- -0.24554 -0.24208 -0.23281 -0.17324

Alpha virt. eigenvalues -- -0.00614 -0.00204 0.00211 0.00822 0.05218

LUMO E: -0.00614 au = -0.167 eV

HOMO E: -0.17324 au = -4.714 eV

DELTA E: +0.16710 au = +4.547 eV

Zero-point correction= 0.512613 (Hartree/Particle)

Thermal correction to Energy= 0.540492

Thermal correction to Enthalpy= 0.541436

Thermal correction to Gibbs Free Energy= 0.454662

Sum of electronic and zero-point Energies= -1278.162454

Sum of electronic and thermal Energies= -1278.134576

Sum of electronic and thermal Enthalpies= -1278.133632

Sum of electronic and thermal Free Energies= -1278.220405

en_Conf1

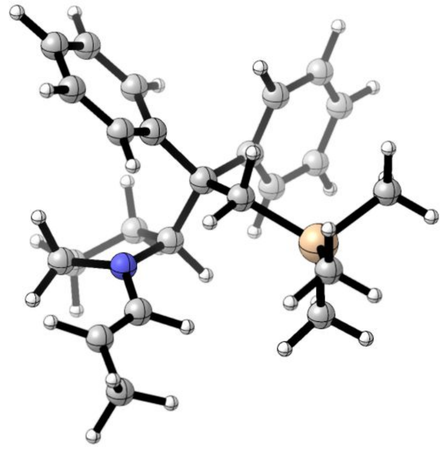

```
--------------------------------------------------------------------------------
    -- Stationary point found.
         Item               Value     Threshold  Converged?
 Maximum Force            0.000009     0.000450     YES
 RMS     Force            0.000002     0.000300     YES
 Maximum Displacement     0.000589     0.001800     YES
 RMS     Displacement     0.000126     0.001200     YES
 Predicted change in Energy=-3.845920D-09
 Optimization completed.

 SCF Done:  E(RB3LYP) =  -1278.67556445     A.U. after    6 cycles

     Eigenvalues ---    0.00172   0.00232   0.00345   0.00468   0.00477

                         Standard orientation:
 ---------------------------------------------------------------------
 Center     Atomic      Atomic             Coordinates (Angstroms)
 Number     Number       Type             X           Y           Z
 ---------------------------------------------------------------------
      1          6           0       -0.715845   -1.144033    2.303685
      2          6           0       -0.669644   -0.039025    1.205712
      3          7           0       -2.080175    0.168261    0.849522
      4          6           0       -2.942983   -0.866986    1.417330
      5          6           0       -2.198544   -1.287337    2.690187
      6          6           0        0.245686   -0.385354   -0.051599
      7          6           0       -0.352352   -1.634229   -0.748336
      8          6           0       -1.549341   -3.920876   -1.930120
```

9	6	0	-1.399573	-1.515328	-1.677714
10	6	0	0.075203	-2.935743	-0.431002
11	6	0	-0.510650	-4.062390	-1.011127
12	6	0	-1.989842	-2.638922	-2.259177
13	6	0	1.718008	-0.629586	0.346726
14	6	0	4.477272	-0.974989	0.913199
15	6	0	2.261263	-0.261528	1.586144
16	6	0	2.604058	-1.166797	-0.606117
17	6	0	3.958196	-1.341321	-0.330879
18	6	0	3.620894	-0.431730	1.867740
19	6	0	0.243675	0.831114	-1.041068
20	14	0	1.189769	2.482348	-0.766668
21	6	0	1.048167	3.256916	0.962573
22	6	0	3.028776	2.329574	-1.193765
23	6	0	0.394531	3.679534	-2.008811
24	6	0	-2.574550	1.422363	0.525322
25	6	0	-3.824918	1.740981	0.139111
26	6	0	-4.270379	3.152935	-0.121900
27	1	0	-0.337038	-2.090654	1.912920
28	1	0	-0.096935	-0.883137	3.165947
29	1	0	-0.284758	0.897683	1.627199
30	1	0	-3.070304	-1.710822	0.725844
31	1	0	-3.930907	-0.444845	1.624270
32	1	0	-2.451163	-2.300148	3.021435
33	1	0	-2.450925	-0.595271	3.502372
34	1	0	-2.005941	-4.795341	-2.386245
35	1	0	-1.772785	-0.536406	-1.952447
36	1	0	0.893918	-3.077045	0.266835
37	1	0	-0.147422	-5.051368	-0.742956
38	1	0	-2.797296	-2.506284	-2.975026
39	1	0	5.532783	-1.111396	1.132507
40	1	0	1.631902	0.168606	2.355758
41	1	0	2.221648	-1.462614	-1.578470
42	1	0	4.609794	-1.763322	-1.091833
43	1	0	4.003825	-0.138124	2.841862
44	1	0	0.636196	0.473430	-2.004416
45	1	0	-0.791751	1.119368	-1.250685
46	1	0	0.011924	3.439940	1.269166

47	1	0	1.525868	2.641929	1.732775
48	1	0	1.560080	4.228077	0.959924
49	1	0	3.574371	1.714136	-0.471714
50	1	0	3.175048	1.885010	-2.185492
51	1	0	3.493579	3.323831	-1.207664
52	1	0	-0.667344	3.846482	-1.790261
53	1	0	0.893942	4.656066	-1.990163
54	1	0	0.461884	3.296341	-3.034587
55	1	0	-1.833401	2.216318	0.609749
56	1	0	-4.568135	0.957394	0.002940
57	1	0	-4.672161	3.274839	-1.138129
58	1	0	-5.066336	3.472161	0.567016
59	1	0	-3.440361	3.860995	-0.008423

Alpha occ. eigenvalues -- -0.24582 -0.24028 -0.23114 -0.17340

Alpha virt. eigenvalues -- -0.00592 -0.00135 0.00362 0.01021 0.05248

LUMO E: -0.00592 au = -0.161 eV

HOMO E: -0.17340 au = -4.718 eV

DELTA E: +0.16748 au = +4.557 eV

Zero-point correction= 0.512692 (Hartree/Particle)

Thermal correction to Energy= 0.540480

Thermal correction to Enthalpy= 0.541424

Thermal correction to Gibbs Free Energy= 0.455311

Sum of electronic and zero-point Energies= -1278.162873

Sum of electronic and thermal Energies= -1278.135085

Sum of electronic and thermal Enthalpies= -1278.134140

Sum of electronic and thermal Free Energies= -1278.220253

en_Conf3b

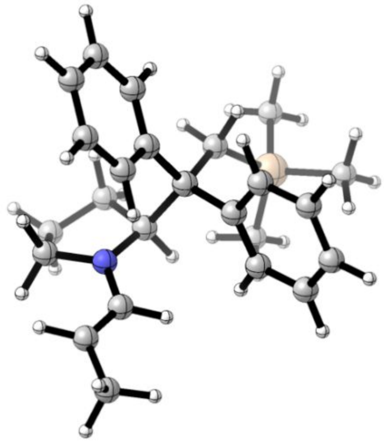

```
   -- Stationary point found.
         Item               Value     Threshold  Converged?
 Maximum Force            0.000002     0.000450     YES
 RMS     Force            0.000000     0.000300     YES
 Maximum Displacement     0.000079     0.001800     YES
 RMS     Displacement     0.000018     0.001200     YES
 Predicted change in Energy=-2.741085D-11
 Optimization completed.

 SCF Done:  E(RB3LYP) =  -1278.67438666     A.U. after    6 cycles

    Eigenvalues ---    0.00089   0.00123   0.00145   0.00210   0.00235

                         Standard orientation:
 ---------------------------------------------------------------------
 Center     Atomic      Atomic             Coordinates (Angstroms)
 Number     Number       Type             X           Y           Z
 ---------------------------------------------------------------------
      1          6           0        0.365319   -1.847486   -1.893480
      2          6           0        0.341873   -0.419349   -1.283565
      3          7           0        1.724965    0.042111   -1.479419
      4          6           0        2.578372   -0.968048   -2.095449
      5          6           0        1.580808   -1.869333   -2.827249
      6          6           0       -0.210293   -0.413248    0.212704
      7          6           0       -0.485246    1.008111    0.769151
      8          6           0       -1.174630    3.527939    1.883956
```

9	6	0	-0.500198	1.218835	2.158826
10	6	0	-0.851045	2.094533	-0.041327
11	6	0	-1.181132	3.338452	0.503125
12	6	0	-0.838346	2.454727	2.709531
13	6	0	0.822975	-1.141422	1.109061
14	6	0	2.800318	-2.450619	2.666965
15	6	0	2.044237	-0.514171	1.422550
16	6	0	0.622133	-2.436382	1.609682
17	6	0	1.595059	-3.084255	2.378059
18	6	0	3.017369	-1.157754	2.184837
19	6	0	-1.589483	-1.157763	0.233221
20	14	0	-3.231354	-0.563562	-0.568577
21	6	0	-4.130015	0.702456	0.514794
22	6	0	-3.104361	0.088700	-2.347709
23	6	0	-4.294297	-2.138198	-0.620120
24	6	0	2.134580	1.359061	-1.384133
25	6	0	3.394305	1.831278	-1.477797
26	6	0	3.724702	3.297111	-1.419810
27	1	0	0.507764	-2.590198	-1.101952
28	1	0	-0.569978	-2.084682	-2.408091
29	1	0	-0.335339	0.204846	-1.879699
30	1	0	3.159137	-1.528357	-1.346132
31	1	0	3.288310	-0.483020	-2.774709
32	1	0	1.961744	-2.880937	-3.001939
33	1	0	1.331259	-1.428439	-3.799867
34	1	0	-1.429888	4.494460	2.309841
35	1	0	-0.236282	0.402570	2.823208
36	1	0	-0.887147	1.987540	-1.119598
37	1	0	-1.445771	4.157695	-0.160682
38	1	0	-0.834622	2.576822	3.789708
39	1	0	3.558118	-2.951263	3.264008
40	1	0	2.237608	0.494073	1.074963
41	1	0	-0.305514	-2.961452	1.413279
42	1	0	1.400038	-4.087132	2.749819
43	1	0	3.948219	-0.641563	2.406176
44	1	0	-1.455155	-2.177676	-0.146263
45	1	0	-1.870309	-1.284535	1.289248
46	1	0	-4.269051	0.316738	1.532188

47	1	0	-3.588695	1.649959	0.591759
48	1	0	-5.126109	0.913845	0.105476
49	1	0	-2.605046	1.061131	-2.412026
50	1	0	-2.572927	-0.607455	-3.007293
51	1	0	-4.114609	0.216170	-2.757572
52	1	0	-4.396123	-2.587999	0.375307
53	1	0	-5.305198	-1.917858	-0.984566
54	1	0	-3.864247	-2.899278	-1.283049
55	1	0	1.327438	2.062401	-1.207092
56	1	0	4.229821	1.145224	-1.601948
57	1	0	4.441289	3.524902	-0.617454
58	1	0	4.180050	3.658281	-2.354058
59	1	0	2.827396	3.901221	-1.239903

Alpha occ. eigenvalues -- -0.24371 -0.24098 -0.22985 -0.16938

Alpha virt. eigenvalues -- -0.00423 -0.00260 0.00513 0.01131 0.05442

LUMO E: -0.00423 au = -0.115 eV

HOMO E: -0.16938 au = -4.609 eV

DELTA E: +0.16515 au = +4.494 eV

Zero-point correction= 0.512670 (Hartree/Particle)

Thermal correction to Energy= 0.540498

Thermal correction to Enthalpy= 0.541442

Thermal correction to Gibbs Free Energy= 0.455198

Sum of electronic and zero-point Energies= -1278.161717

Sum of electronic and thermal Energies= -1278.133889

Sum of electronic and thermal Enthalpies= -1278.132945

Sum of electronic and thermal Free Energies= -1278.219188

en_Conf4b

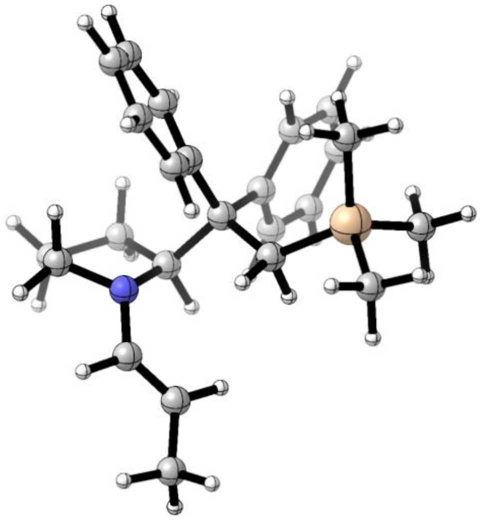

-- Stationary point found.

Item		Value	Threshold	Converged?
Maximum	Force	0.000000	0.000450	YES
RMS	Force	0.000000	0.000300	YES
Maximum	Displacement	0.000059	0.001800	YES
RMS	Displacement	0.000013	0.001200	YES

Predicted change in Energy=-9.286977D-12

Optimization completed.

SCF Done: E(RB3LYP) = -1278.67348425   A.U. after   6 cycles

Eigenvalues ---   0.00083   0.00110   0.00124   0.00170   0.00237

Standard orientation:

Center Number	Atomic Number	Atomic Type	Coordinates (Angstroms) X	Y	Z
1	6	0	1.625623	-1.456702	-1.934343
2	6	0	1.316199	-0.152318	-1.135254
3	7	0	2.516742	0.088879	-0.307050
4	6	0	3.323421	-1.143167	-0.280567
5	6	0	3.112773	-1.753475	-1.670435
6	6	0	-0.033194	-0.181852	-0.282874

7	6	0	0.155279	-1.211195	0.852486
8	6	0	0.709266	-3.117860	2.886009
9	6	0	0.736545	-0.834857	2.075378
10	6	0	-0.132456	-2.577643	0.681647
11	6	0	0.135786	-3.516620	1.679736
12	6	0	1.008613	-1.768885	3.076382
13	6	0	-1.237294	-0.501041	-1.201820
14	6	0	-3.521543	-0.907758	-2.843225
15	6	0	-2.402924	-1.109429	-0.705069
16	6	0	-1.263132	-0.086118	-2.544438
17	6	0	-2.382357	-0.287851	-3.354067
18	6	0	-3.525232	-1.314295	-1.509179
19	6	0	-0.250413	1.268418	0.262463
20	14	0	-1.742197	1.863816	1.319122
21	6	0	-3.190711	2.356744	0.199141
22	6	0	-2.367389	0.677922	2.660246
23	6	0	-1.111747	3.435240	2.181637
24	6	0	3.215047	1.297134	-0.396581
25	6	0	2.820798	2.495538	-0.862638
26	6	0	3.712800	3.706935	-0.847324
27	1	0	1.011263	-2.286214	-1.577856
28	1	0	1.405933	-1.331819	-2.998142
29	1	0	1.236206	0.687017	-1.831658
30	1	0	2.950149	-1.817248	0.499533
31	1	0	4.366959	-0.909176	-0.052665
32	1	0	3.346289	-2.822758	-1.713898
33	1	0	3.747844	-1.236812	-2.400261
34	1	0	0.917924	-3.845415	3.665884
35	1	0	0.999999	0.200071	2.252924
36	1	0	-0.591233	-2.920912	-0.239146
37	1	0	-0.107820	-4.562095	1.508039
38	1	0	1.458875	-1.435846	4.008199
39	1	0	-4.392568	-1.068421	-3.472711
40	1	0	-2.437244	-1.435140	0.328259
41	1	0	-0.404031	0.410902	-2.981398
42	1	0	-2.358494	0.044377	-4.388860
43	1	0	-4.404379	-1.793491	-1.085663
44	1	0	0.656580	1.583514	0.790509

45	1	0	-0.295727	1.927928	-0.616886
46	1	0	-2.878343	3.098894	-0.546030
47	1	0	-3.607411	1.500814	-0.341366
48	1	0	-3.998095	2.806415	0.790968
49	1	0	-2.802971	-0.236902	2.245694
50	1	0	-1.573311	0.378670	3.351880
51	1	0	-3.150297	1.178047	3.245241
52	1	0	-0.725784	4.164152	1.458361
53	1	0	-1.914786	3.925240	2.746008
54	1	0	-0.301484	3.212438	2.886896
55	1	0	4.218782	1.218454	0.019882
56	1	0	1.819545	2.638848	-1.259314
57	1	0	3.873637	4.110464	-1.857534
58	1	0	3.286520	4.525849	-0.249665
59	1	0	4.698487	3.473397	-0.427450

Alpha occ. eigenvalues -- -0.24526 -0.24272 -0.23224 -0.17794

Alpha virt. eigenvalues -- -0.00586 -0.00126 0.00153 0.00818 0.05730

LUMO E: -0.00586 au = -0.159 eV

HOMO E: -0.17794 au = -4.842 eV

DELTA E: +0.17208 au = +4.683 eV

Zero-point correction= 0.513011 (Hartree/Particle)

Thermal correction to Energy= 0.540707

Thermal correction to Enthalpy= 0.541651

Thermal correction to Gibbs Free Energy= 0.455748

Sum of electronic and zero-point Energies= -1278.160473

Sum of electronic and thermal Energies= -1278.132778

Sum of electronic and thermal Enthalpies= -1278.131834

Sum of electronic and thermal Free Energies= -1278.217736

en_Conf8b

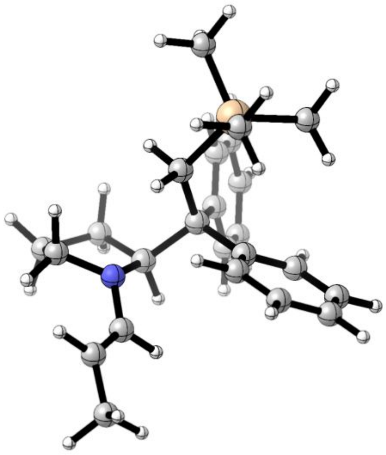

```
-------------------------------------------------------------------------------------
   -- Stationary point found.
         Item               Value     Threshold  Converged?
 Maximum Force            0.000001     0.000450     YES
 RMS     Force            0.000000     0.000300     YES
 Maximum Displacement     0.000222     0.001800     YES
 RMS     Displacement     0.000041     0.001200     YES
 Predicted change in Energy=-5.075052D-11
 Optimization completed.

 SCF Done:  E(RB3LYP) =  -1278.67291863     A.U. after    7 cycles

    Eigenvalues ---    0.00090   0.00121   0.00134   0.00159   0.00201

                         Standard orientation:
 ---------------------------------------------------------------------
 Center     Atomic      Atomic             Coordinates (Angstroms)
 Number     Number       Type             X           Y           Z
 ---------------------------------------------------------------------
      1          6           0       -0.419947   -2.705304   -0.953029
      2          6           0       -0.885705   -1.475687   -0.116540
      3          7           0       -2.125669   -1.025383   -0.774909
      4          6           0       -2.322454   -1.740983   -2.043470
      5          6           0       -1.632297   -3.090027   -1.811669
      6          6           0        0.262418   -0.361639    0.046744
      7          6           0        1.487404   -1.141256    0.573092
```

8	6	0	3.665453	-2.699220	1.524218
9	6	0	2.706553	-1.196947	-0.116177
10	6	0	1.391740	-1.903363	1.754831
11	6	0	2.458289	-2.667515	2.224994
12	6	0	3.781733	-1.959334	0.350427
13	6	0	-0.196723	0.739856	1.042660
14	6	0	-1.070512	2.811476	2.783716
15	6	0	0.429750	0.976676	2.277326
16	6	0	-1.263917	1.592208	0.699663
17	6	0	-1.698764	2.604581	1.555556
18	6	0	0.000657	1.993188	3.135087
19	6	0	0.519724	0.301256	-1.340004
20	14	0	1.586405	1.889561	-1.598538
21	6	0	0.440633	3.347023	-1.998806
22	6	0	2.722261	2.395756	-0.169558
23	6	0	2.657690	1.562105	-3.135835
24	6	0	-3.235473	-0.706650	0.011965
25	6	0	-4.479619	-0.401795	-0.396567
26	6	0	-5.603135	-0.096745	0.555078
27	1	0	0.425215	-2.440664	-1.595070
28	1	0	-0.080055	-3.517727	-0.306372
29	1	0	-1.144887	-1.802153	0.897207
30	1	0	-1.860539	-1.196564	-2.878823
31	1	0	-3.389606	-1.838151	-2.254831
32	1	0	-1.353853	-3.599840	-2.740366
33	1	0	-2.308103	-3.749662	-1.253840
34	1	0	4.499220	-3.293455	1.888170
35	1	0	2.832659	-0.649442	-1.042150
36	1	0	0.470118	-1.893833	2.329110
37	1	0	2.343474	-3.239586	3.142230
38	1	0	4.710229	-1.972654	-0.214707
39	1	0	-1.406098	3.601185	3.450713
40	1	0	1.280531	0.378261	2.578015
41	1	0	-1.773633	1.462755	-0.246194
42	1	0	-2.532936	3.233210	1.254321
43	1	0	0.517310	2.143657	4.079824
44	1	0	0.912885	-0.452662	-2.033495
45	1	0	-0.465573	0.563197	-1.745064

46	1	0	-0.223166	3.118159	-2.841953
47	1	0	-0.187515	3.610604	-1.141035
48	1	0	1.023897	4.235563	-2.271039
49	1	0	2.151888	2.679761	0.720238
50	1	0	3.416030	1.599678	0.120221
51	1	0	3.320288	3.264015	-0.475675
52	1	0	2.041898	1.288201	-4.001720
53	1	0	3.230537	2.456322	-3.410920
54	1	0	3.378668	0.750306	-2.976481
55	1	0	-3.013352	-0.684552	1.077750
56	1	0	-4.713635	-0.355803	-1.458843
57	1	0	-6.034743	0.897981	0.372694
58	1	0	-6.430084	-0.816017	0.463231
59	1	0	-5.261622	-0.122211	1.596539

Alpha occ. eigenvalues -- -0.24247 -0.23950 -0.23459 -0.17800

Alpha virt. eigenvalues -- -0.00738 -0.00054 0.00340 0.00861 0.05527

LUMO E: -0.00738 au = -0.201 eV

HOMO E: -0.17800 au = -4.844 eV

DELTA E: +0.17062 au = +4.643 eV

Zero-point correction= 0.512906 (Hartree/Particle)

Thermal correction to Energy= 0.540627

Thermal correction to Enthalpy= 0.541572

Thermal correction to Gibbs Free Energy= 0.455241

Sum of electronic and zero-point Energies= -1278.160013

Sum of electronic and thermal Energies= -1278.132291

Sum of electronic and thermal Enthalpies= -1278.131347

Sum of electronic and thermal Free Energies= -1278.217678

en_Conf14b

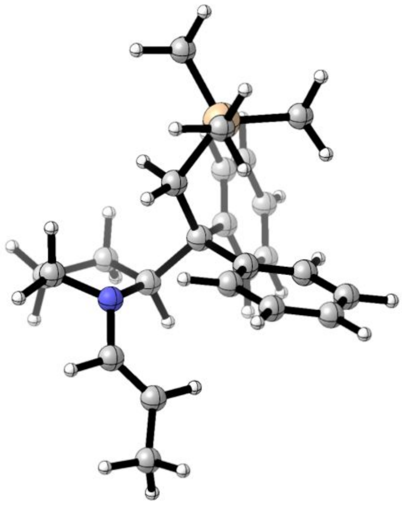

```
   -- Stationary point found.
         Item               Value     Threshold  Converged?
 Maximum Force            0.000000     0.000450     YES
 RMS     Force            0.000000     0.000300     YES
 Maximum Displacement     0.000021     0.001800     YES
 RMS     Displacement     0.000005     0.001200     YES
 Predicted change in Energy=-8.201265D-13
 Optimization completed.

 SCF Done:  E(RB3LYP) =  -1278.67179431     A.U. after    6 cycles

    Eigenvalues ---    0.00086   0.00113   0.00123   0.00132   0.00223

                         Standard orientation:
 ---------------------------------------------------------------------
 Center     Atomic      Atomic             Coordinates (Angstroms)
 Number     Number       Type             X           Y           Z
 ---------------------------------------------------------------------
      1          6           0       -0.738645   -2.518954   -1.246279
      2          6           0       -1.133908   -1.213304   -0.478848
      3          7           0       -2.121297   -0.537521   -1.356749
      4          6           0       -2.062854   -1.166829   -2.688816
      5          6           0       -1.751425   -2.637051   -2.395566
      6          6           0        0.129736   -0.332897   -0.043445
      7          6           0        1.040426   -1.326259    0.711483
      8          6           0        2.608093   -3.267735    2.067235
```

9	6	0	2.324824	-1.670022	0.269008
10	6	0	0.560010	-1.996790	1.853620
11	6	0	1.327196	-2.948606	2.522619
12	6	0	3.101150	-2.622882	0.935906
13	6	0	-0.307361	0.854127	0.860275
14	6	0	-1.074550	3.108267	2.409696
15	6	0	0.172094	1.044838	2.166394
16	6	0	-1.171729	1.839863	0.348233
17	6	0	-1.553569	2.944344	1.109387
18	6	0	-0.206325	2.152077	2.931261
19	6	0	0.805119	0.260305	-1.317648
20	14	0	2.205527	1.589244	-1.286880
21	6	0	1.468152	3.272827	-1.752644
22	6	0	3.173202	1.788609	0.329409
23	6	0	3.423544	1.085334	-2.657970
24	6	0	-3.414080	-0.291482	-0.861707
25	6	0	-3.827503	-0.143232	0.407149
26	6	0	-5.254427	0.164149	0.768873
27	1	0	0.275563	-2.444359	-1.649029
28	1	0	-0.747815	-3.384531	-0.579226
29	1	0	-1.658166	-1.491860	0.438486
30	1	0	-1.255507	-0.715108	-3.279825
31	1	0	-2.999777	-1.004134	-3.228413
32	1	0	-1.354453	-3.179954	-3.260225
33	1	0	-2.663908	-3.146556	-2.062798
34	1	0	3.209193	-4.009107	2.586692
35	1	0	2.737612	-1.202579	-0.616944
36	1	0	-0.428185	-1.762473	2.238505
37	1	0	0.921247	-3.441612	3.402326
38	1	0	4.093581	-2.859323	0.560594
39	1	0	-1.370456	3.969199	3.003444
40	1	0	0.864358	0.333482	2.599708
41	1	0	-1.562383	1.733693	-0.655864
42	1	0	-2.230938	3.678166	0.679743
43	1	0	0.189241	2.262632	3.937939
44	1	0	1.165537	-0.557794	-1.954170
45	1	0	0.003777	0.734917	-1.897387
46	1	0	0.941377	3.229076	-2.714192

47	1	0	0.754521	3.619262	-0.997262
48	1	0	2.255814	4.031255	-1.843090
49	1	0	2.538456	2.168295	1.135977
50	1	0	3.623778	0.851684	0.672505
51	1	0	3.984359	2.511826	0.173552
52	1	0	2.916535	0.976239	-3.624934
53	1	0	4.209040	1.840810	-2.783013
54	1	0	3.920578	0.131675	-2.438951
55	1	0	-4.144666	-0.166146	-1.660192
56	1	0	-3.123122	-0.207696	1.232006
57	1	0	-5.685448	-0.605782	1.424792
58	1	0	-5.340911	1.117762	1.309183
59	1	0	-5.890173	0.230083	-0.122136

Alpha occ. eigenvalues -- -0.24192 -0.23733 -0.23236 -0.18133

Alpha virt. eigenvalues -- -0.00656 0.00009 0.00500 0.01246 0.05567

LUMO E: -0.00656 au = -0.179 eV

HOMO E: -0.18133 au = -4.934 eV

DELTA E: +0.17477 au = +4.756 eV

Zero-point correction= 0.513159 (Hartree/Particle)

Thermal correction to Energy= 0.540732

Thermal correction to Enthalpy= 0.541676

Thermal correction to Gibbs Free Energy= 0.455898

Sum of electronic and zero-point Energies= -1278.158636

Sum of electronic and thermal Energies= -1278.131063

Sum of electronic and thermal Enthalpies= -1278.130119

Sum of electronic and thermal Free Energies= -1278.215896

en_Conf7

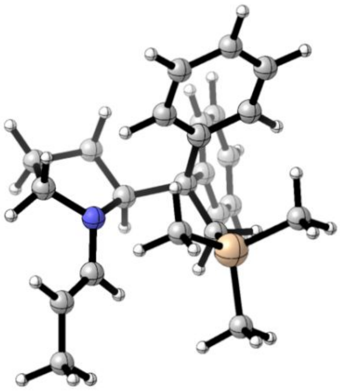

```
--------------------------------------------------------------------------------
   -- Stationary point found.
         Item               Value     Threshold  Converged?
 Maximum Force            0.000024     0.000450     YES
 RMS     Force            0.000004     0.000300     YES
 Maximum Displacement     0.000721     0.001800     YES
 RMS     Displacement     0.000174     0.001200     YES
 Predicted change in Energy=-1.576382D-08
 Optimization completed.

 SCF Done:  E(RB3LYP) =  -1278.66831801     A.U. after    7 cycles

    Eigenvalues ---    0.00080   0.00103   0.00254   0.00348   0.00463

                         Standard orientation:
 ---------------------------------------------------------------------
 Center     Atomic      Atomic             Coordinates (Angstroms)
 Number     Number       Type             X           Y           Z
 ---------------------------------------------------------------------
      1          6           0       -0.769369   -1.950676    1.775207
      2          6           0        0.002643   -1.443944    0.529088
      3          7           0        1.411394   -1.350480    0.980365
      4          6           0        1.546831   -1.781699    2.383090
      5          6           0        0.297021   -2.631382    2.641414
      6          6           0       -0.654799   -0.182293   -0.189533
      7          6           0       -0.930540    1.047675    0.699672
      8          6           0       -1.431614    3.399655    2.205800
      9          6           0       -1.853841    2.016728    0.265284
```

10	6	0	-0.251376	1.305524	1.898374
11	6	0	-0.501175	2.460500	2.645538
12	6	0	-2.103935	3.172816	1.003377
13	6	0	-1.998404	-0.766285	-0.725599
14	6	0	-4.395300	-1.979137	-1.639238
15	6	0	-3.172486	-0.744474	0.046962
16	6	0	-2.060512	-1.426405	-1.963130
17	6	0	-3.239311	-2.022739	-2.416503
18	6	0	-4.353354	-1.336506	-0.401532
19	6	0	0.231767	0.291193	-1.396086
20	14	0	1.617018	1.638739	-1.372408
21	6	0	2.720836	1.832059	0.150316
22	6	0	2.718008	1.170520	-2.849894
23	6	0	0.834547	3.326565	-1.749304
24	6	0	2.416538	-1.717336	0.082876
25	6	0	3.735313	-1.835506	0.322578
26	6	0	4.723251	-2.292417	-0.715019
27	1	0	-1.217381	-1.114248	2.319752
28	1	0	-1.580368	-2.624475	1.491473
29	1	0	-0.041071	-2.225116	-0.243999
30	1	0	1.596820	-0.923083	3.067925
31	1	0	2.473985	-2.348462	2.502951
32	1	0	0.021673	-2.680202	3.700279
33	1	0	0.475681	-3.656118	2.293067
34	1	0	-1.627912	4.296634	2.787005
35	1	0	-2.394113	1.859816	-0.663311
36	1	0	0.501838	0.613042	2.247767
37	1	0	0.041603	2.622100	3.573580
38	1	0	-2.827644	3.896244	0.636370
39	1	0	-5.315181	-2.437952	-1.991787
40	1	0	-3.171308	-0.246838	1.010729
41	1	0	-1.179975	-1.478871	-2.594227
42	1	0	-3.248689	-2.520108	-3.383146
43	1	0	-5.243186	-1.291945	0.221464
44	1	0	0.670794	-0.586969	-1.885956
45	1	0	-0.453152	0.710022	-2.147810
46	1	0	2.164844	2.199644	1.017342
47	1	0	3.212139	0.893931	0.421360

48	1	0	3.501553	2.569185	-0.080713
49	1	0	3.470698	1.945745	-3.039261
50	1	0	3.252851	0.230509	-2.667474
51	1	0	2.133774	1.048880	-3.770724
52	1	0	0.201234	3.677239	-0.927854
53	1	0	1.617107	4.078591	-1.911986
54	1	0	0.217341	3.294326	-2.655990
55	1	0	2.050471	-1.923758	-0.921786
56	1	0	4.136222	-1.592526	1.305193
57	1	0	4.234470	-2.475696	-1.679358
58	1	0	5.515142	-1.548492	-0.880555
59	1	0	5.227300	-3.224654	-0.421607

Alpha occ. eigenvalues --   -0.24349 -0.24226 -0.23177 -0.18277

Alpha virt. eigenvalues --   -0.00520 -0.00148   0.00204   0.00905   0.04396

LUMO E: -0.00520 au = -0.141 eV

HOMO E: -0.18277 au = -4.973 eV

DELTA E: +0.17757 au = +4.832 eV

Zero-point correction=   0.512746 (Hartree/Particle)

Thermal correction to Energy=   0.540468

Thermal correction to Enthalpy=   0.541412

Thermal correction to Gibbs Free Energy=   0.455064

Sum of electronic and zero-point Energies=   -1278.155572

Sum of electronic and thermal Energies=   -1278.127850

Sum of electronic and thermal Enthalpies=   -1278.126906

Sum of electronic and thermal Free Energies=   -1278.213254

en_Conf6b

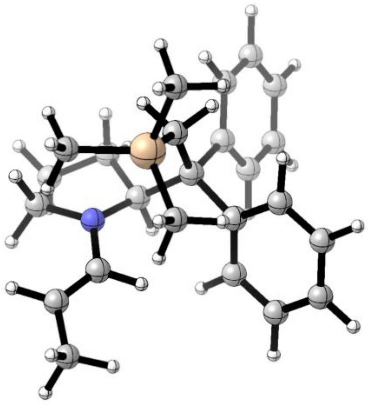

```
--------------------------------------------------------------------------------
   -- Stationary point found.
         Item               Value     Threshold  Converged?
 Maximum Force            0.000001     0.000450     YES
 RMS     Force            0.000000     0.000300     YES
 Maximum Displacement     0.000171     0.001800     YES
 RMS     Displacement     0.000029     0.001200     YES
 Predicted change in Energy=-4.578402D-11
 Optimization completed.

 SCF Done:  E(RB3LYP) =  -1278.66861536     A.U. after    6 cycles

    Eigenvalues ---    0.00069   0.00094   0.00126   0.00142   0.00236

                         Standard orientation:
 ---------------------------------------------------------------------
 Center     Atomic      Atomic             Coordinates (Angstroms)
 Number     Number       Type             X           Y           Z
 ---------------------------------------------------------------------
      1          6           0       -0.750409   -2.624062   -0.625086
      2          6           0       -0.176519   -1.217310   -0.913111
      3          7           0        1.283407   -1.460813   -0.913685
      4          6           0        1.601365   -2.892753   -1.013661
      5          6           0        0.255535   -3.575758   -1.273481
      6          6           0       -0.726073   -0.108120    0.079135
      7          6           0       -2.275503   -0.333268    0.115866
      8          6           0       -5.073605   -0.795752    0.069631
      9          6           0       -2.942087   -0.887251    1.218436
```

10	6	0	-3.054023	-0.014613	-1.011247
11	6	0	-4.428356	-0.245616	-1.039105
12	6	0	-4.321326	-1.112244	1.198433
13	6	0	-0.548008	1.350540	-0.424378
14	6	0	-0.436142	4.062589	-1.269599
15	6	0	-0.798803	2.412315	0.463500
16	6	0	-0.270388	1.695173	-1.756275
17	6	0	-0.206975	3.028693	-2.173145
18	6	0	-0.740452	3.743198	0.054799
19	6	0	-0.118139	-0.280665	1.505300
20	14	0	1.637506	0.184467	2.153549
21	6	0	2.832798	-1.284654	2.101446
22	6	0	2.487940	1.700186	1.404589
23	6	0	1.310980	0.565016	3.991099
24	6	0	2.196205	-0.546039	-1.422542
25	6	0	3.490425	-0.760452	-1.735416
26	6	0	4.389659	0.320140	-2.271364
27	1	0	-0.798590	-2.807935	0.453608
28	1	0	-1.762007	-2.733643	-1.019506
29	1	0	-0.510154	-0.940019	-1.924542
30	1	0	2.076470	-3.252984	-0.090522
31	1	0	2.308571	-3.063723	-1.834544
32	1	0	0.208939	-4.591444	-0.867975
33	1	0	0.072734	-3.640546	-2.353117
34	1	0	-6.146200	-0.969584	0.054096
35	1	0	-2.391768	-1.155906	2.112326
36	1	0	-2.585074	0.439090	-1.878379
37	1	0	-4.996704	0.015344	-1.928367
38	1	0	-4.803560	-1.539032	2.074409
39	1	0	-0.387692	5.099274	-1.591231
40	1	0	-1.061784	2.200992	1.494681
41	1	0	-0.098999	0.925223	-2.500419
42	1	0	0.019906	3.250196	-3.212913
43	1	0	-0.939796	4.532534	0.775171
44	1	0	-0.776180	0.248443	2.204448
45	1	0	-0.218822	-1.335044	1.796491
46	1	0	2.350295	-2.215543	2.424329
47	1	0	3.238955	-1.437864	1.097659

48	1	0	3.676489	-1.102826	2.779521
49	1	0	2.764816	1.544575	0.358375
50	1	0	1.866830	2.599368	1.460539
51	1	0	3.412372	1.894899	1.964268
52	1	0	0.822047	-0.275601	4.499979
53	1	0	2.249385	0.766589	4.522341
54	1	0	0.667243	1.444818	4.117489
55	1	0	1.797217	0.454696	-1.538584
56	1	0	3.941985	-1.739778	-1.594183
57	1	0	5.257953	0.494649	-1.619779
58	1	0	4.791256	0.065071	-3.262934
59	1	0	3.856325	1.273107	-2.369572

Alpha occ. eigenvalues -- -0.24779 -0.24501 -0.22978 -0.17627

Alpha virt. eigenvalues -- -0.00997 -0.00379 0.00168 0.01241 0.05070

LUMO E: -0.00997 au = -0.271 eV

HOMO E: -0.17627 au = -4.797 eV

DELTA E: +0.16630 au = +4.525 eV

Zero-point correction= 0.512992 (Hartree/Particle)

Thermal correction to Energy= 0.540694

Thermal correction to Enthalpy= 0.541639

Thermal correction to Gibbs Free Energy= 0.455902

Sum of electronic and zero-point Energies= -1278.155623

Sum of electronic and thermal Energies= -1278.127921

Sum of electronic and thermal Enthalpies= -1278.126977

Sum of electronic and thermal Free Energies= -1278.212714

8 Copies of ^{1}H and $^{13}C\{^{1}H\}$ NMR spectra

^{1}H NMR (600 MHz, $CDCl_3$) of **7b**

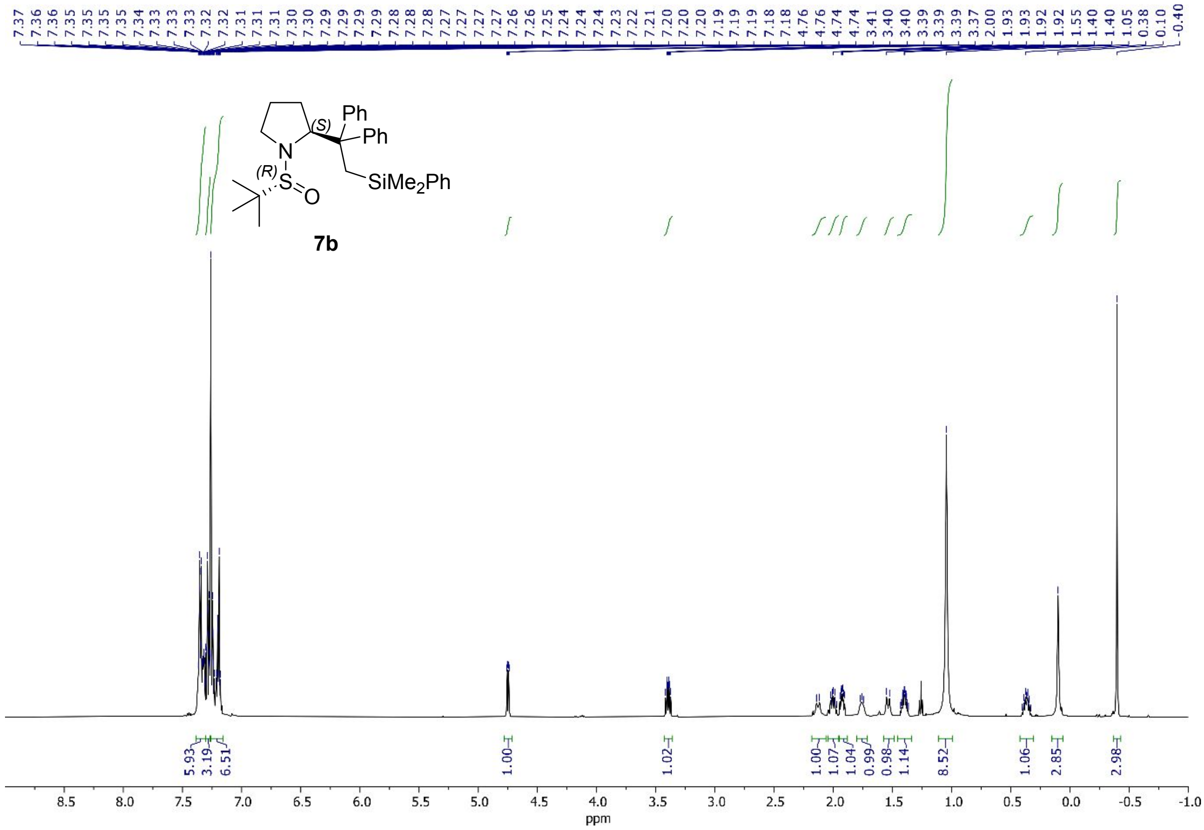

^{13}C{^{1}H} NMR (150 MHz, $CDCl_3$) of **7b**

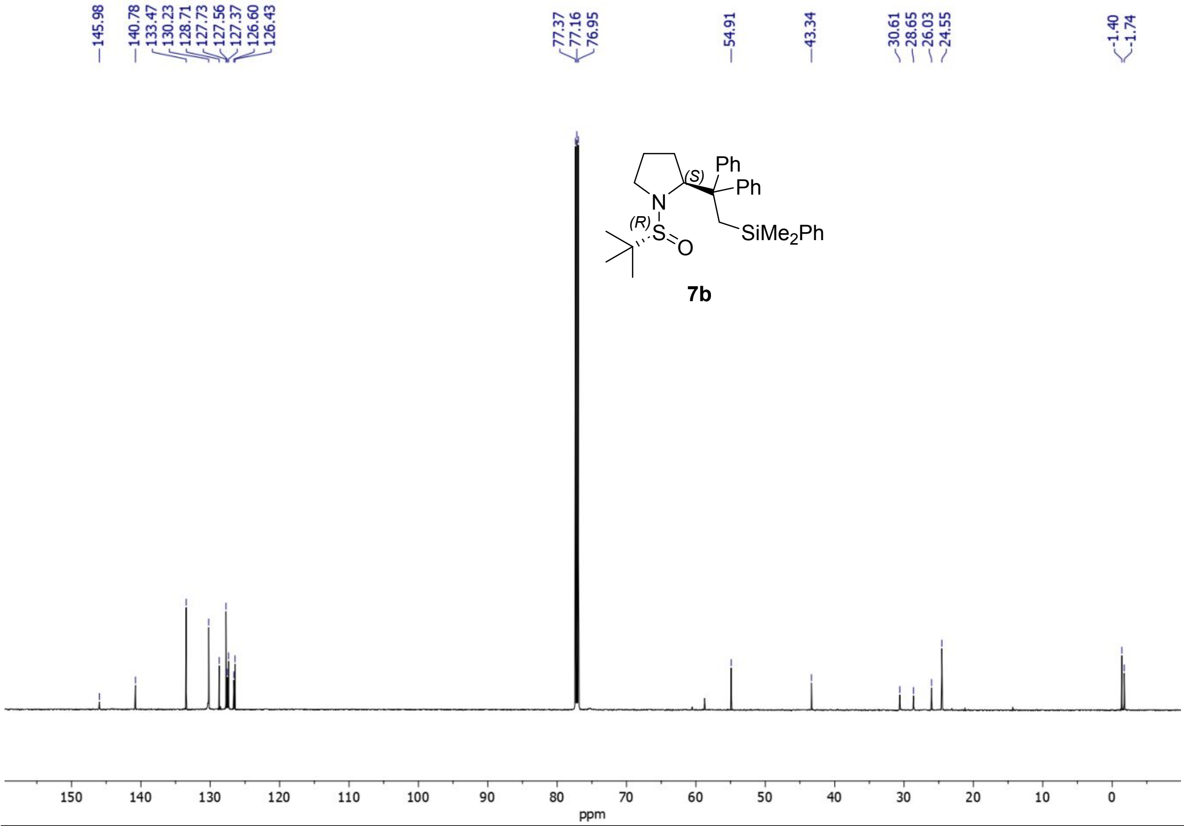

^{1}H NMR (600 MHz, $CDCl_3$) of **7c**

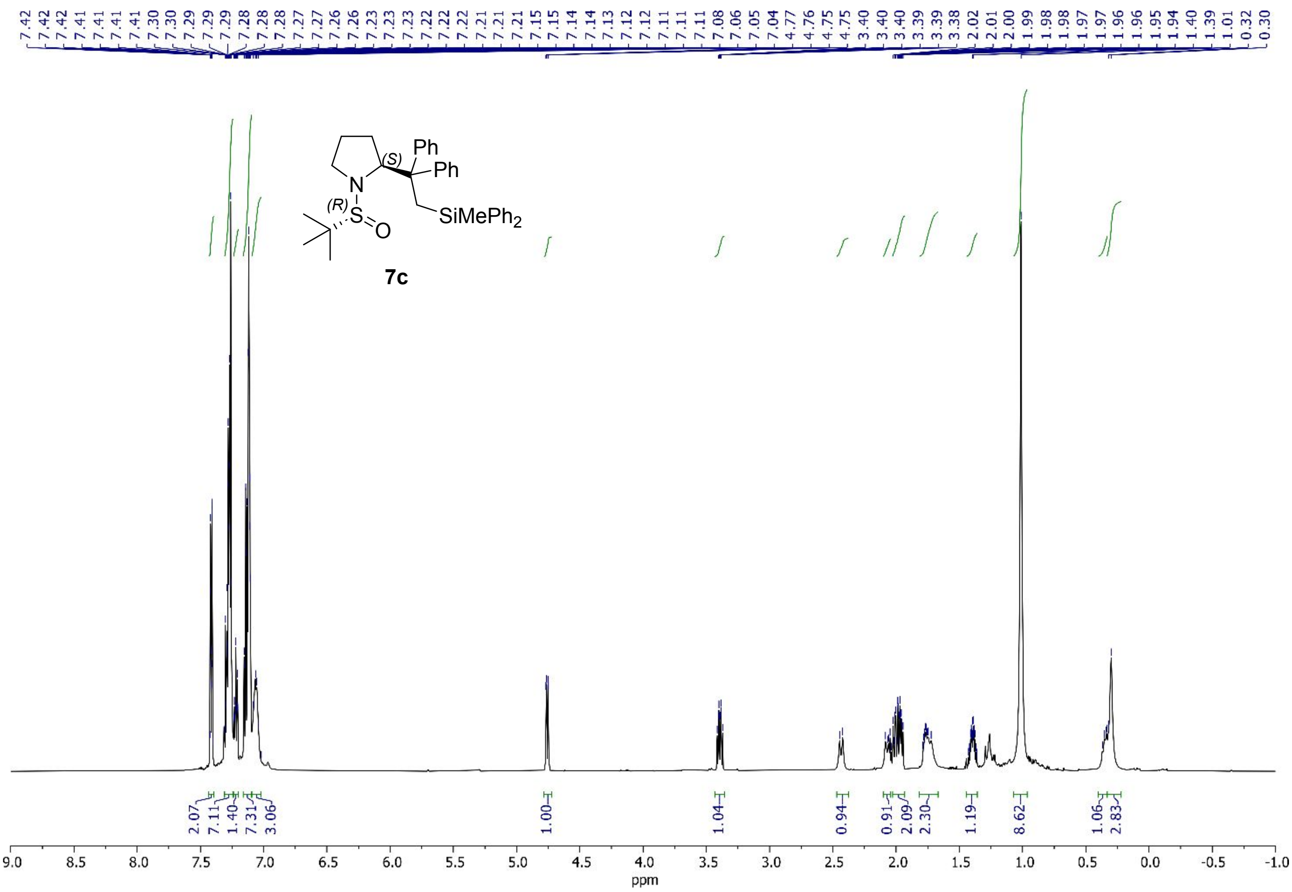

^{13}C{^{1}H} NMR (150 MHz, $CDCl_3$) of **7c**

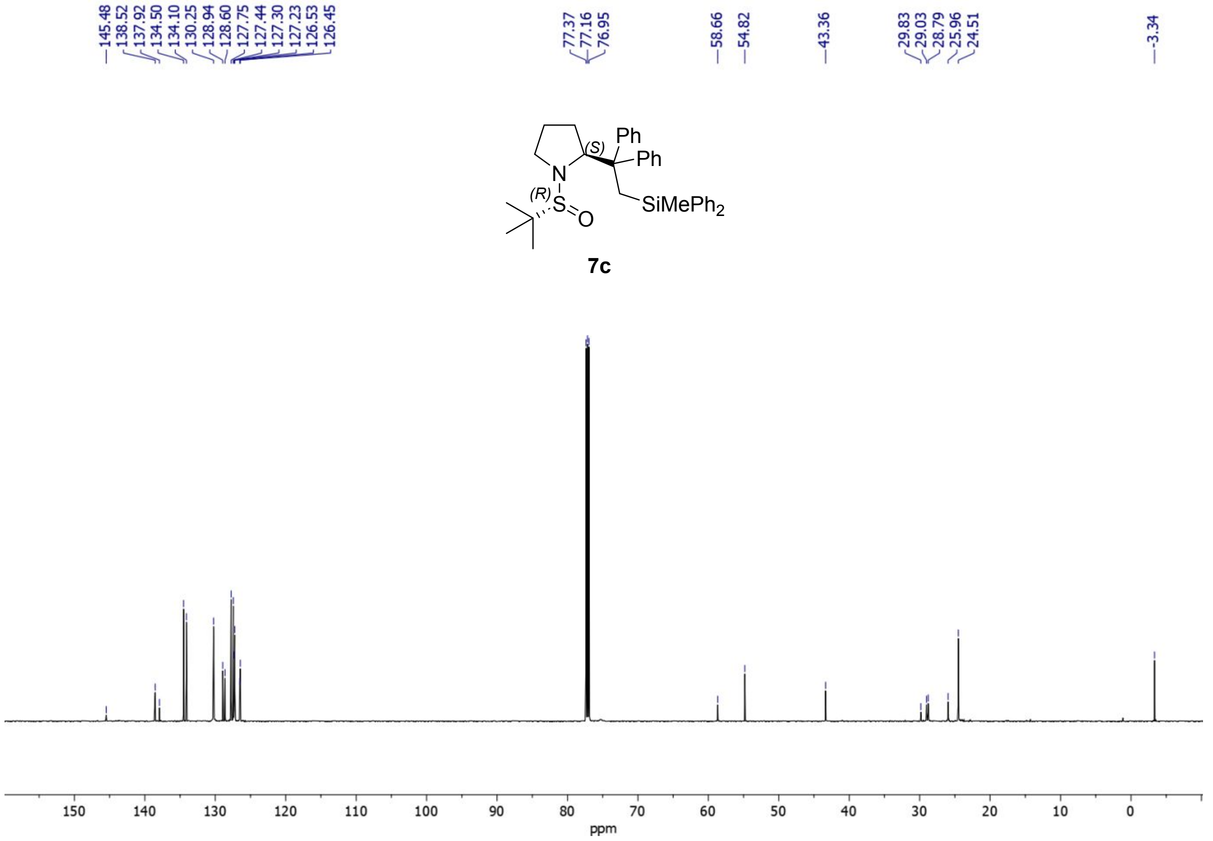

^{1}H NMR (600 MHz, $CDCl_3$) of **7d**

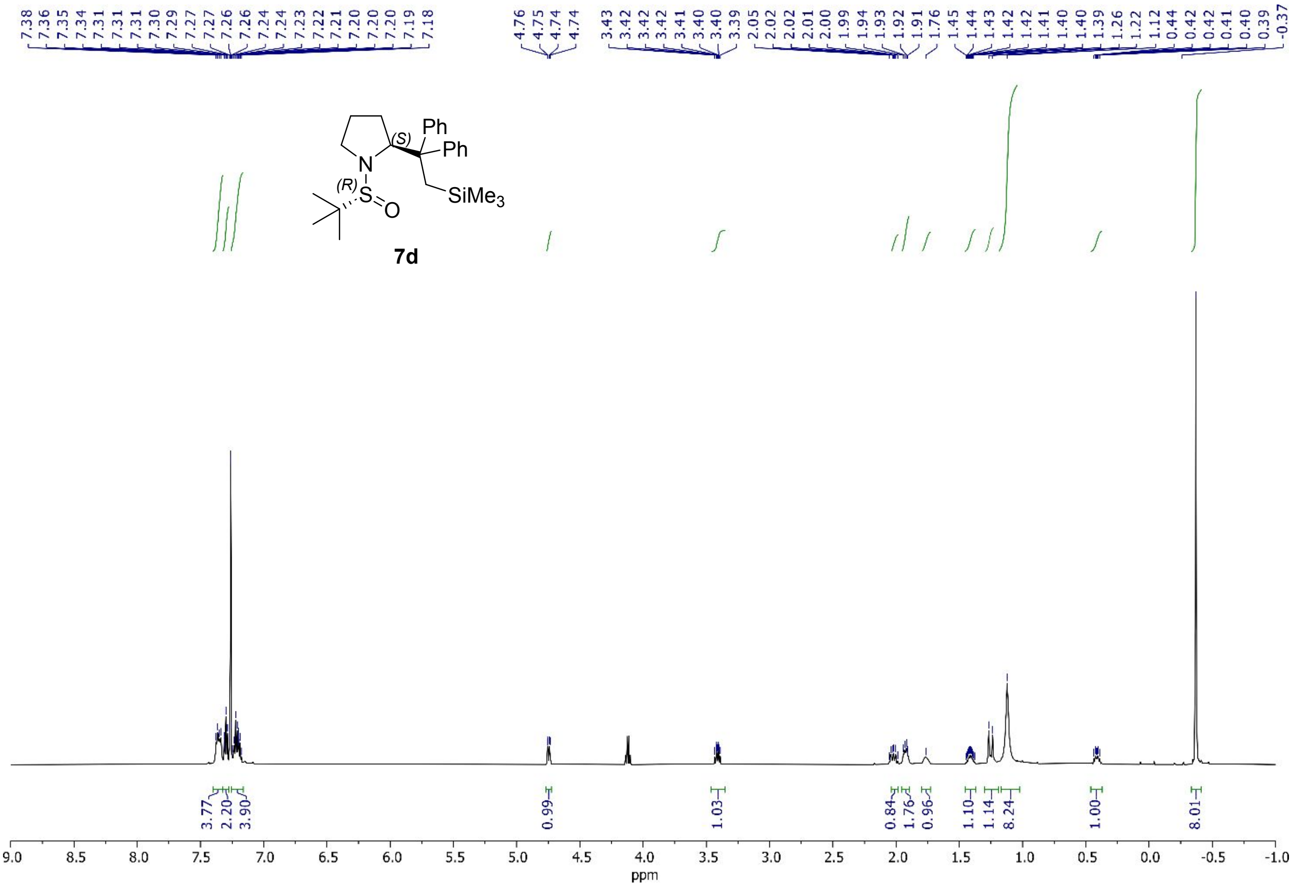

$^{13}C\{^{1}H\}$ NMR (150 MHz, $CDCl_3$) of **7d**

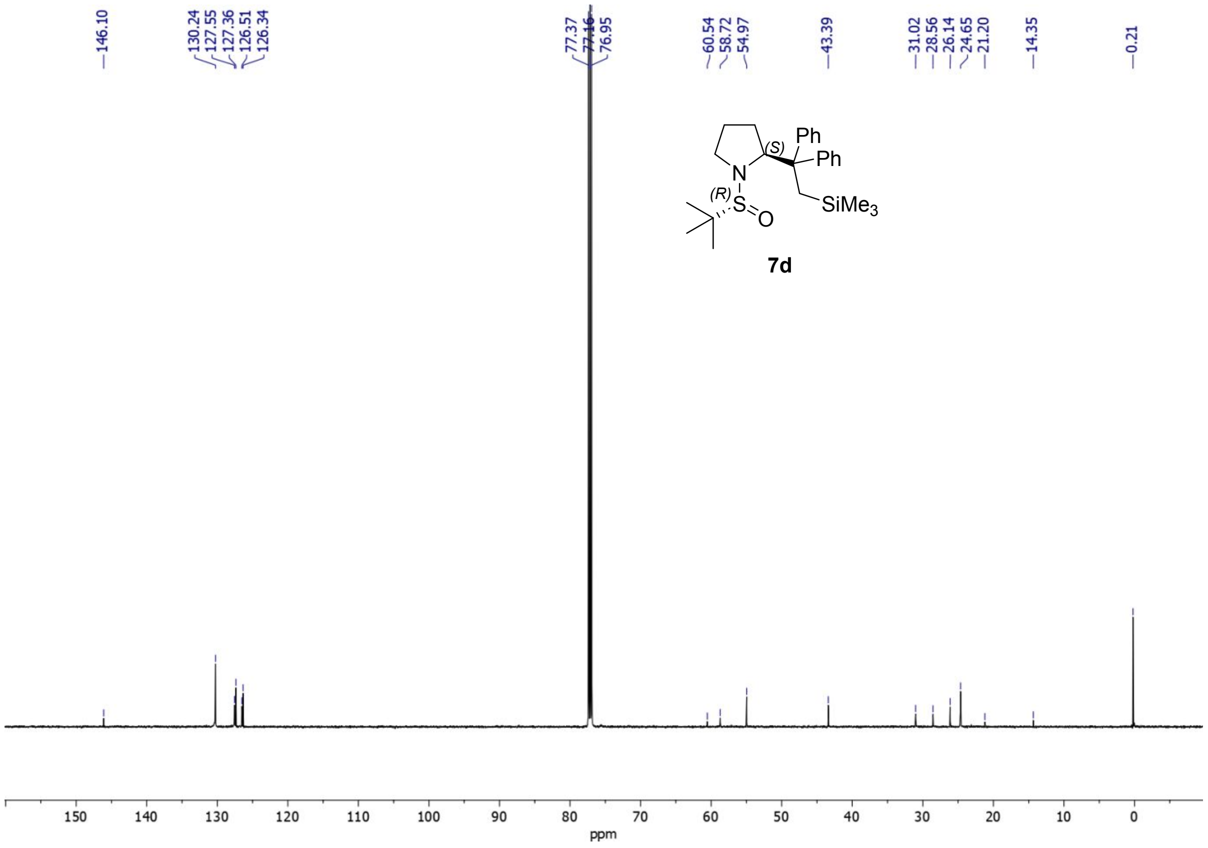

^{1}**H NMR** (600 MHz, CDCl$_3$) of **8b**

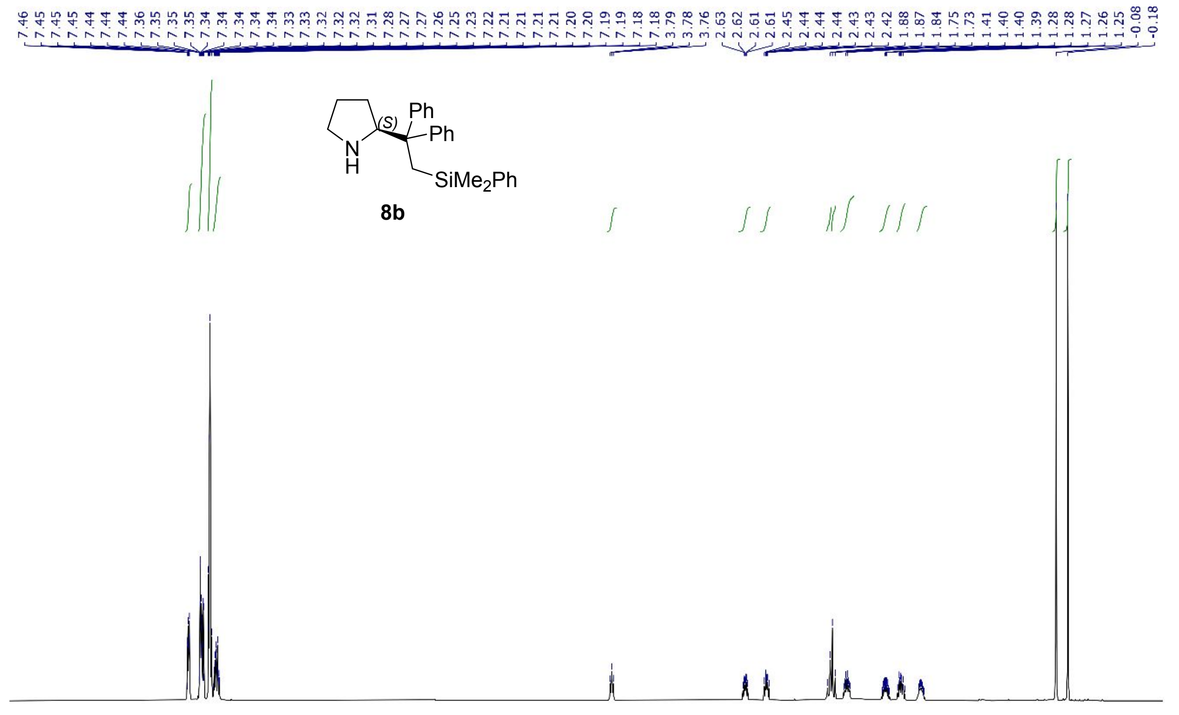

^{13}**C{^{1}H} NMR** (150 MHz, CDCl$_3$) of **8b**

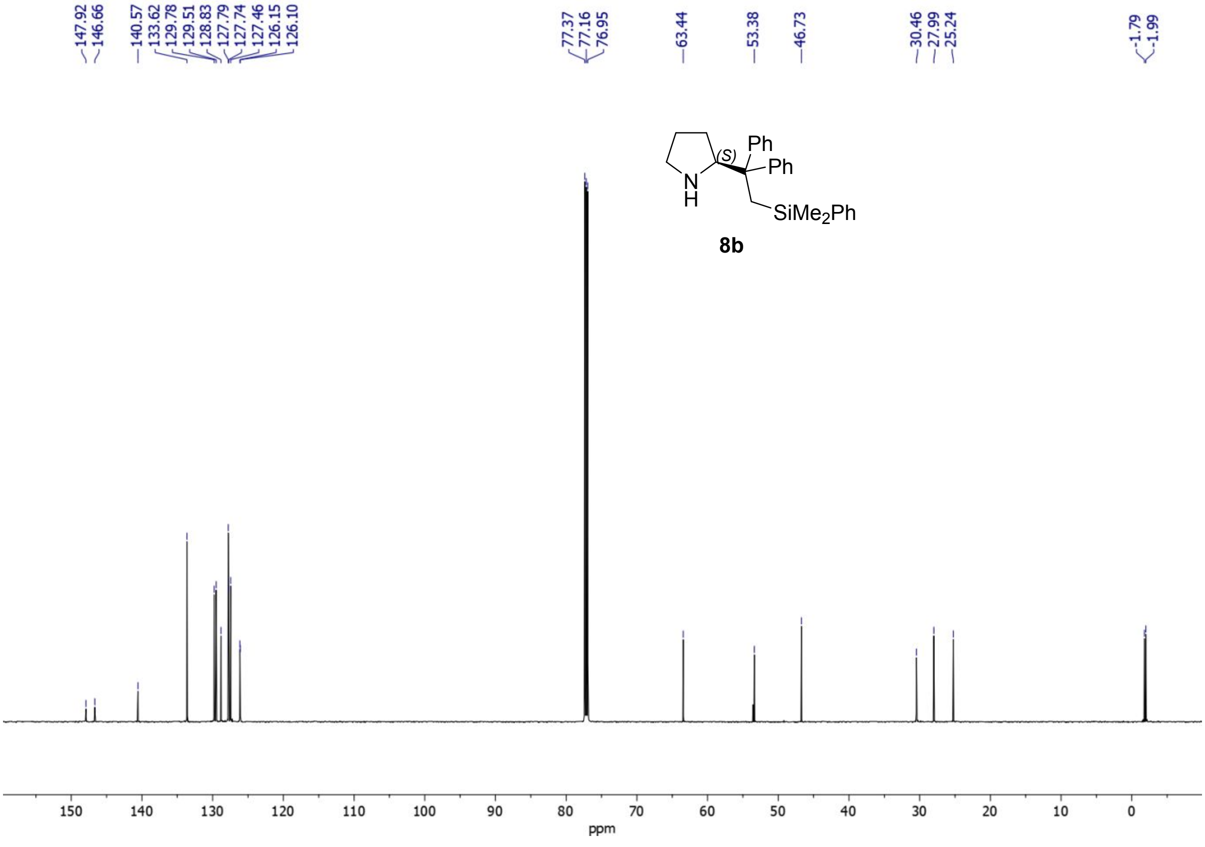
147.92
146.66
140.57
133.62
129.78
129.51
128.83
127.79
127.74
127.46
126.15
126.10
77.37
77.16
76.95
63.44
53.38
46.73
30.46
27.99
25.24
-1.79
-1.99
Ph
(S)
Ph
N
H
SiMe2Ph
8b
150
140
130
120
110
100
90
80
70
60
50
40
30
20
10
0
ppm

[1]**H NMR** (600 MHz, $CDCl_3$) of **8c**

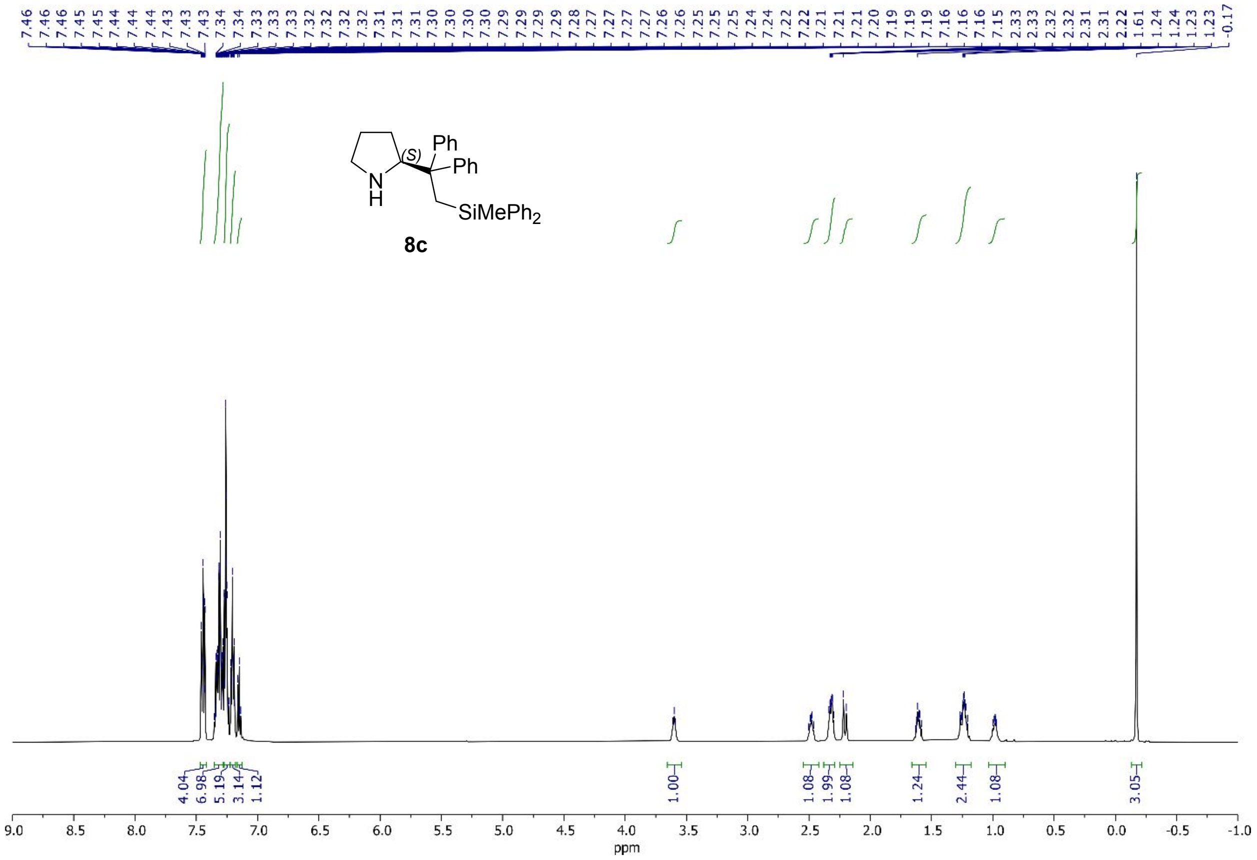

[13]**C{^{1}H} NMR** (150 MHz, $CDCl_3$) of **8c**

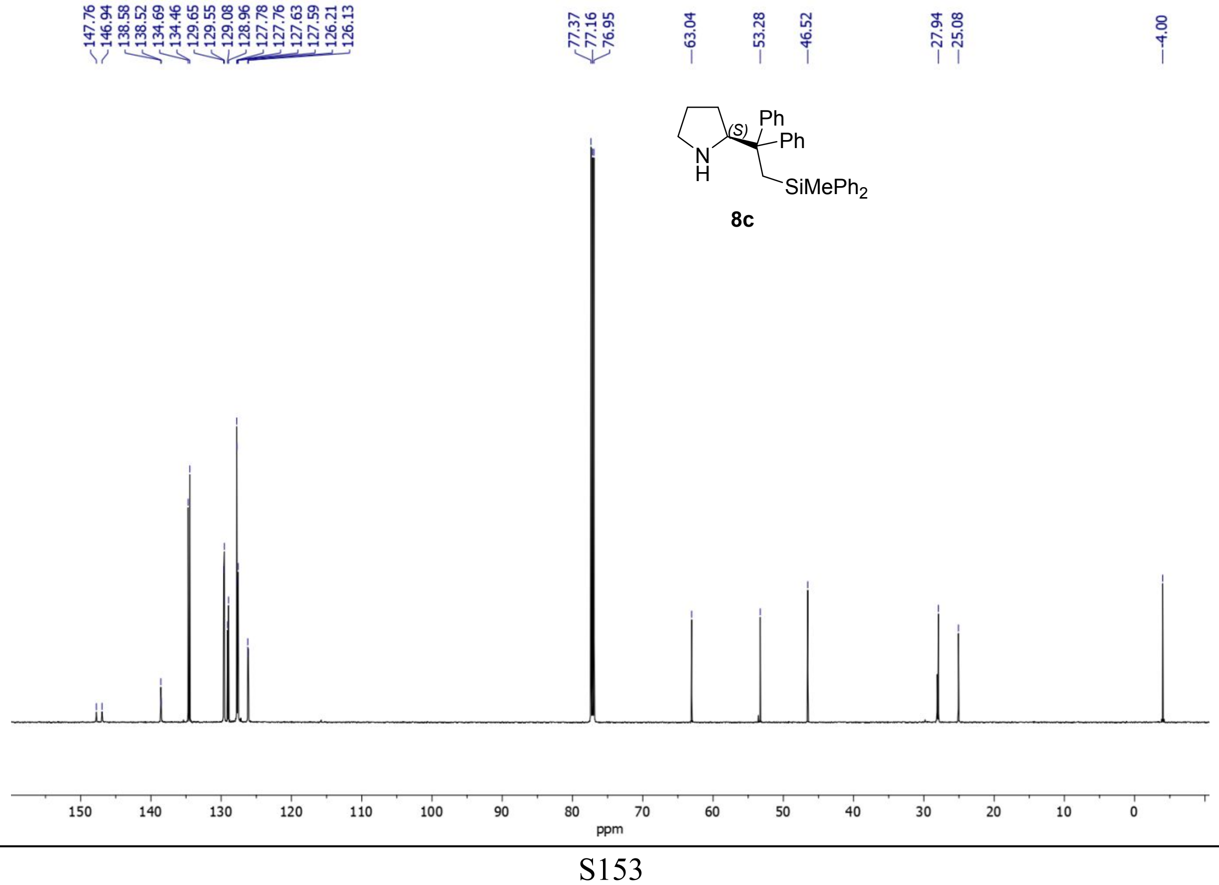

^{1}H NMR (600 MHz, $CDCl_3$) of **8d**

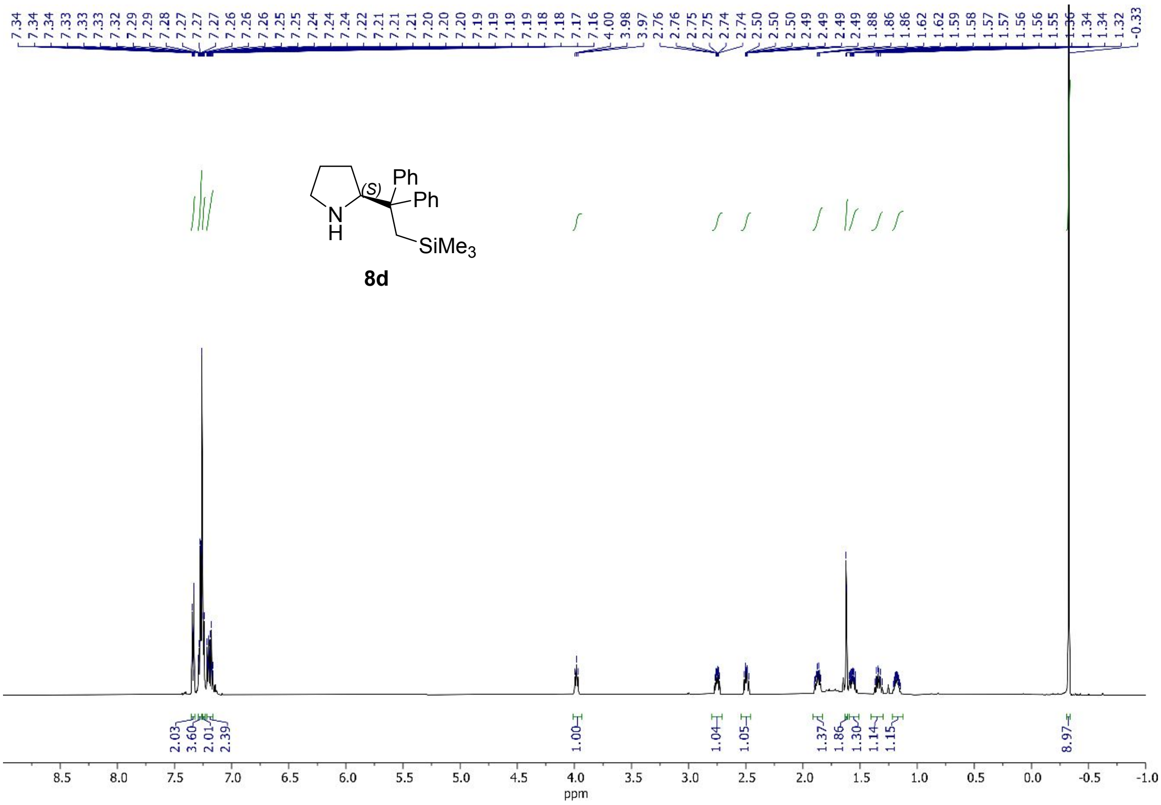

^{13}C{^{1}H} NMR (150 MHz, $CDCl_3$) of **8d**

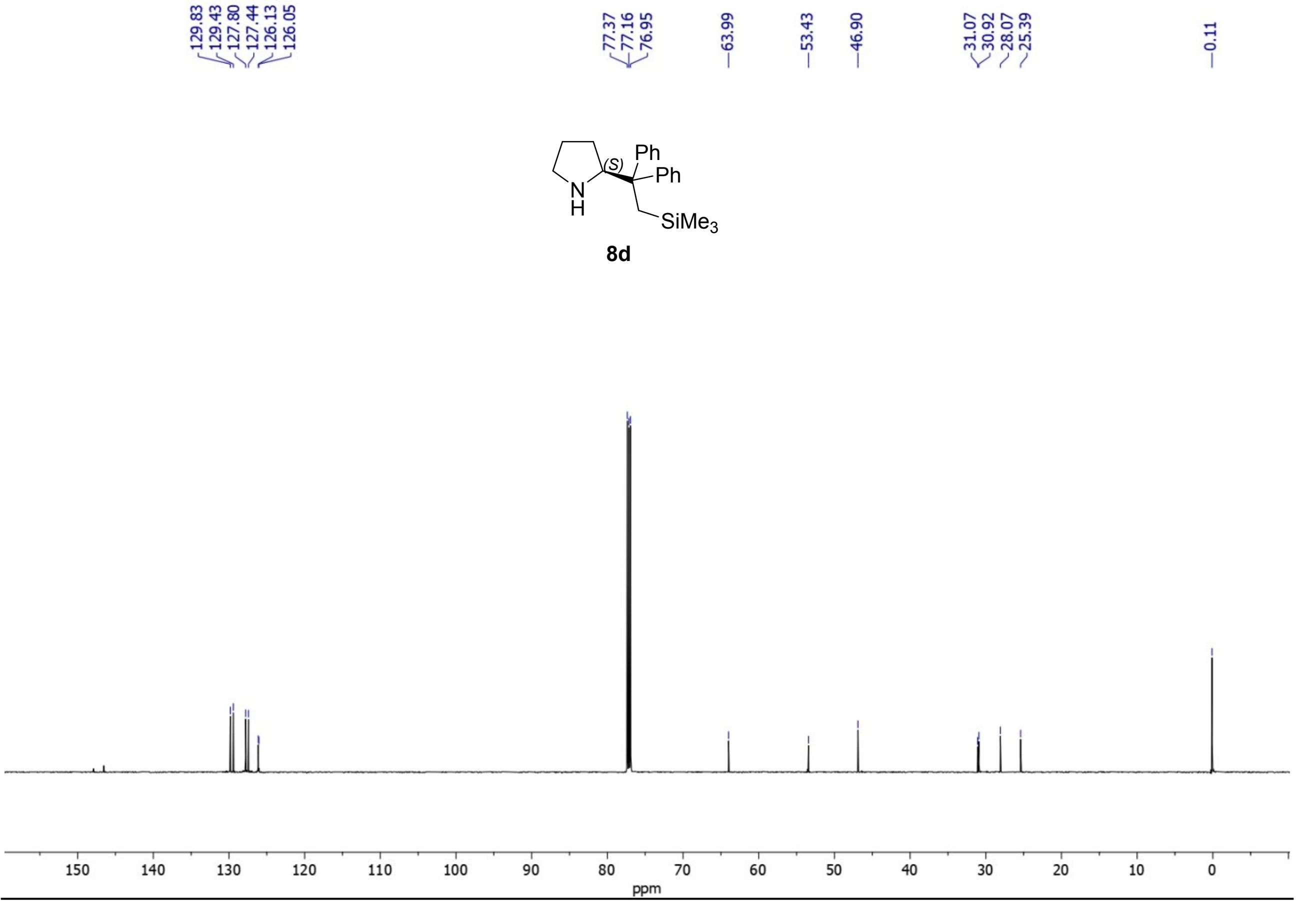

^{1}H NMR (600 MHz, $CDCl_3$) of **10**

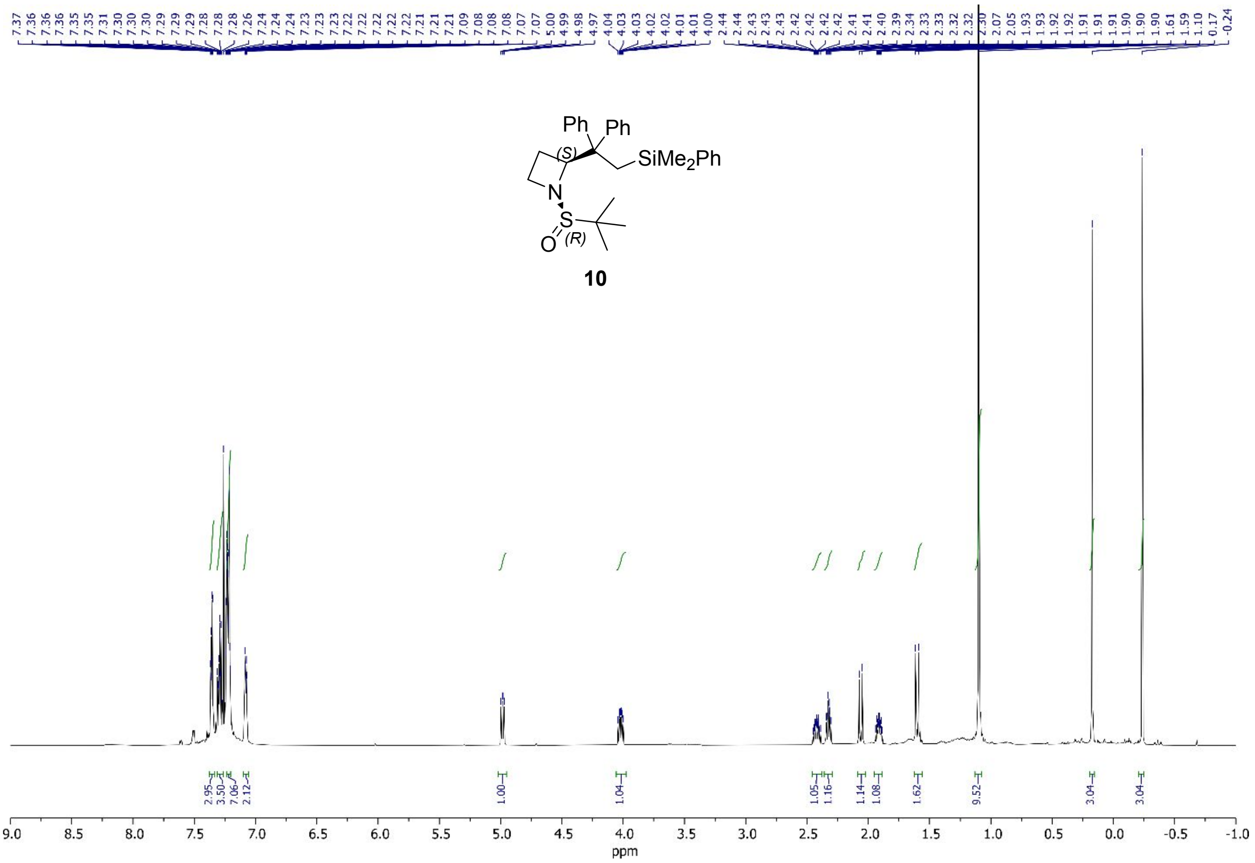

^{13}C{^{1}H} NMR (150 MHz, $CDCl_3$) of **10**

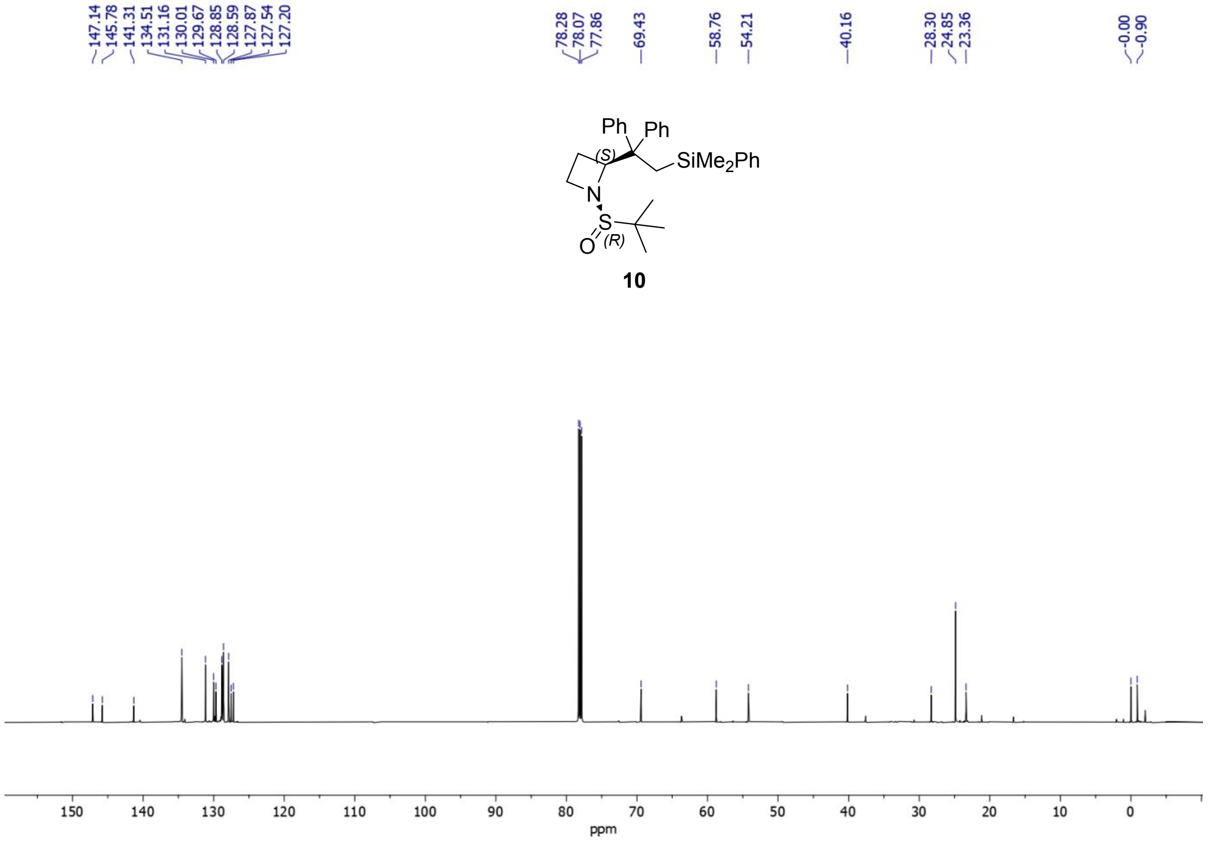

^{1}H NMR (600 MHz, $CDCl_3$) of **11**

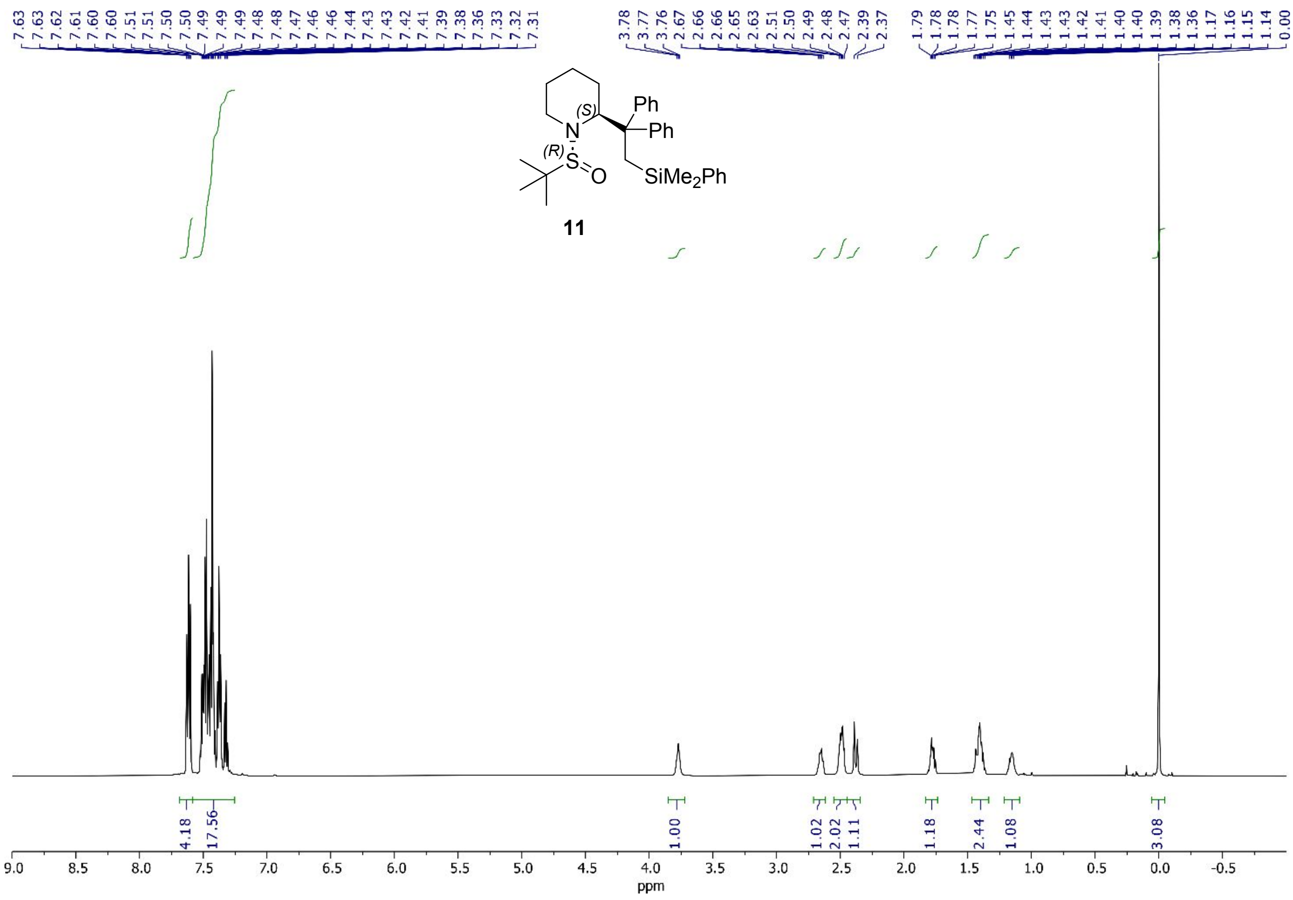

^{13}C{^{1}H} NMR (150 MHz, $CDCl_3$) of **11**

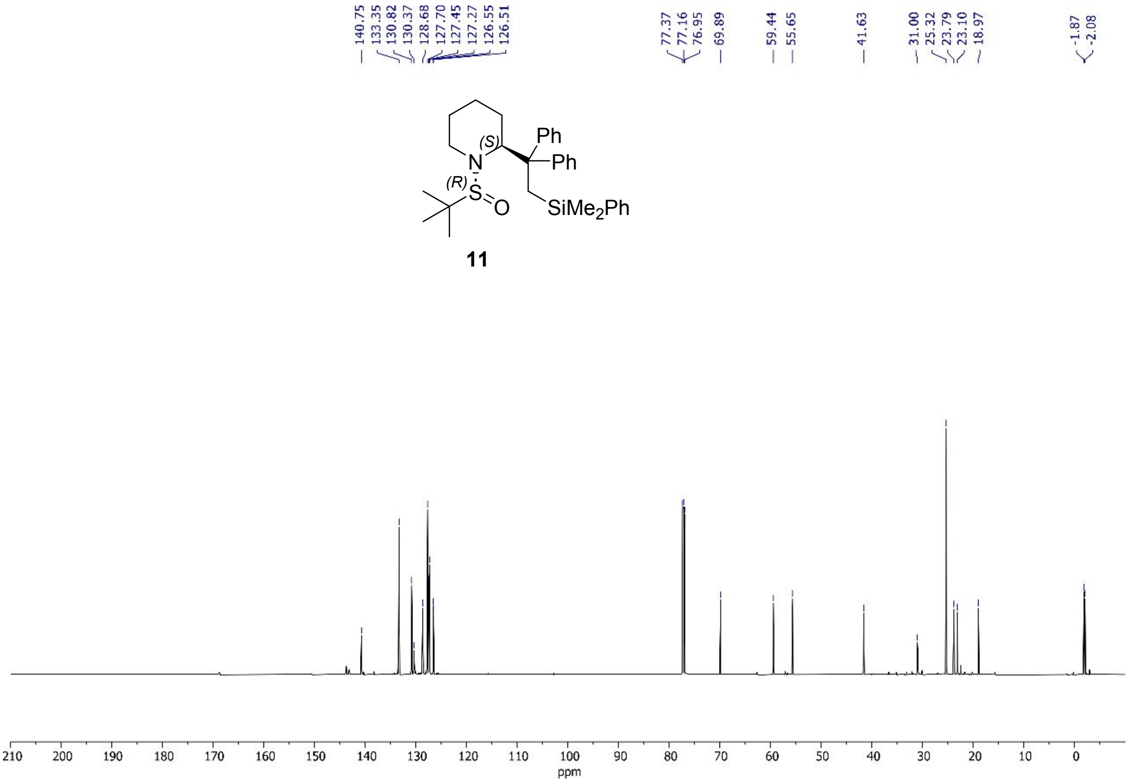

9 Copies of HPLC-MS spectra of products 10 and 11

HPLC-MS of product **10.** *d.r.* 99:1. τ_{max} = 16.71 min, τ_{min} = 18.57 min

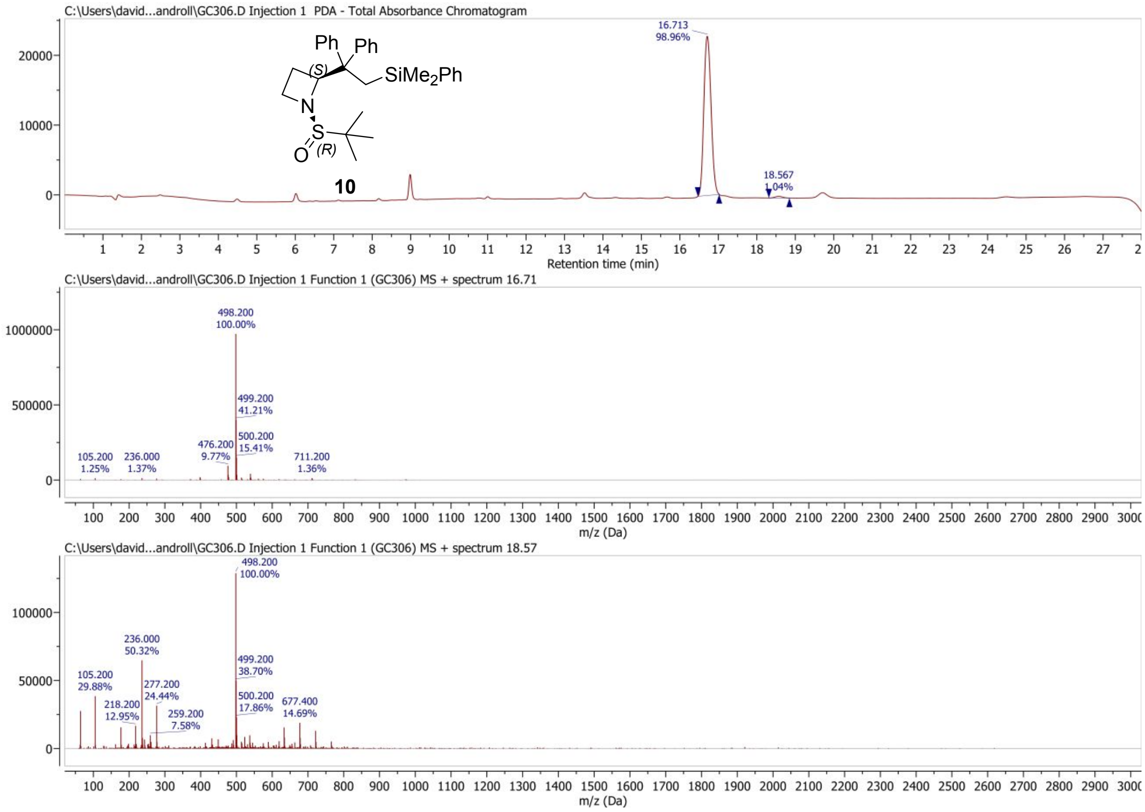

HPLC-MS of product **11.** *d.r.* 94:6. τ_{max} = 18.32 min, τ_{min} = 20.36 min

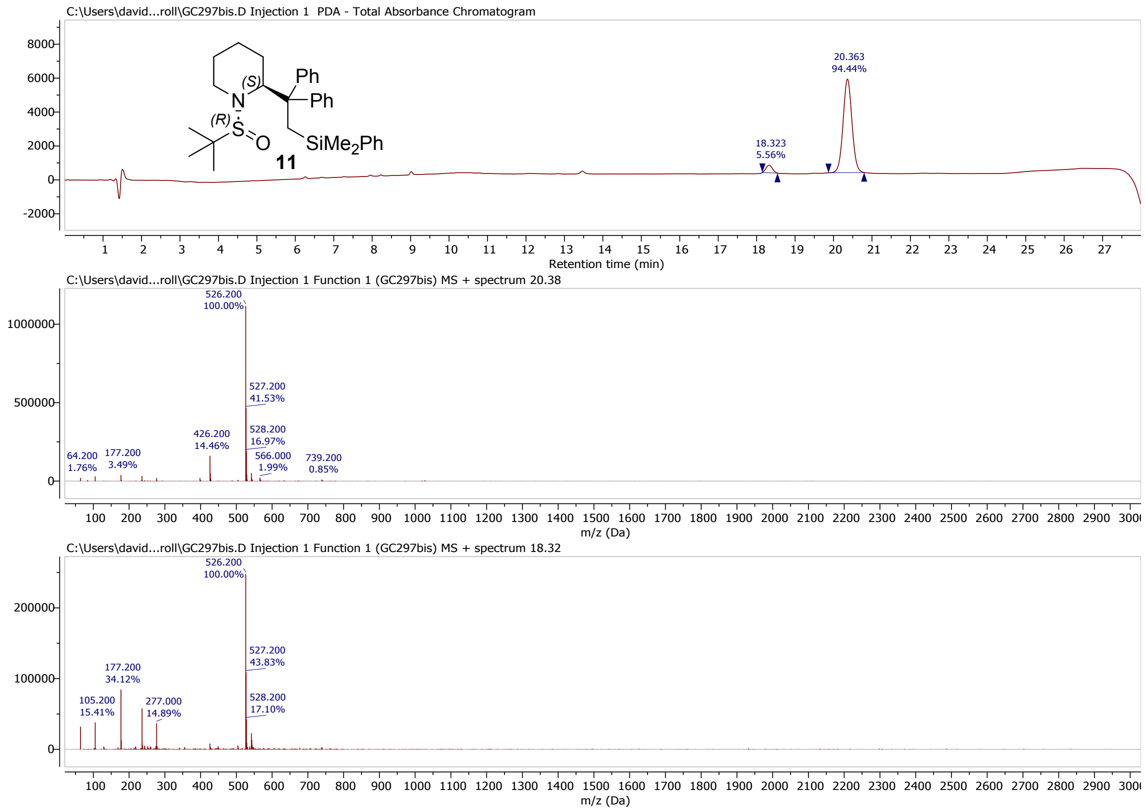

10 Chiral HPLC traces

10.1 Chiral HPLC traces of benzoylated **8b-8d**

The enantiomeric excess of products **8b-d** was determined after benzoylation according to general procedure D.

Benzoylated-8b: Daicel Chiralpak IC column: 90:10 hexane/IPA, flow rate = 0.8 mL/min, λ = 254 nm, τ_{major} = 6.17 min, τ_{minor} = 7.53 min.

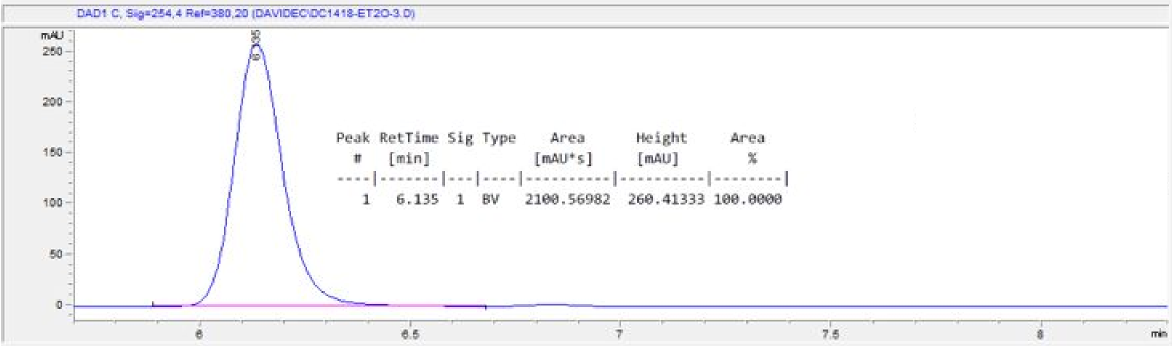

Peak #	RetTime [min]	Sig	Type	Area [mAU*s]	Height [mAU]	Area %
1	6.135	1	BV	2100.56982	260.41333	100.0000

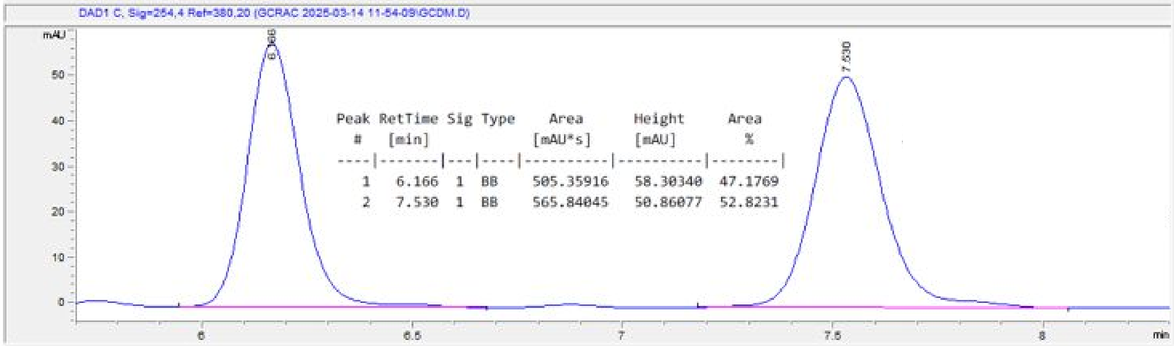

Peak #	RetTime [min]	Sig	Type	Area [mAU*s]	Height [mAU]	Area %
1	6.166	1	BB	505.35916	58.30340	47.1769
2	7.530	1	BB	565.84045	50.86077	52.8231

Benzoylated-8c: Daicel Chiralpak IC column: 90:10 hexane/IPA, flow rate = 0.8 mL/min, λ = 254 nm, τ_{major} = 6.42 min, τ_{minor} = 7.69 min

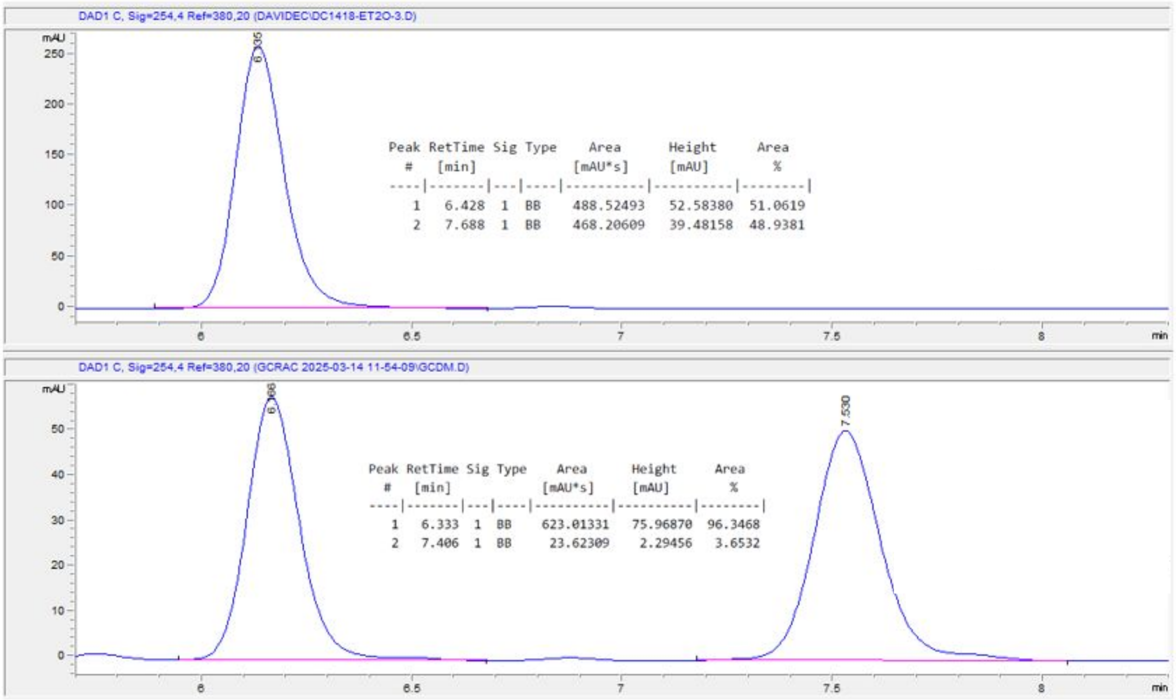

Benzoylated-8d: Daicel Chiralpak IC column: 90:10 hexane/IPA, flow rate = 0.8 mL/min, λ = 254 nm, τ_{major} = 5.75 min, τ_{minor} = 7.26 min.

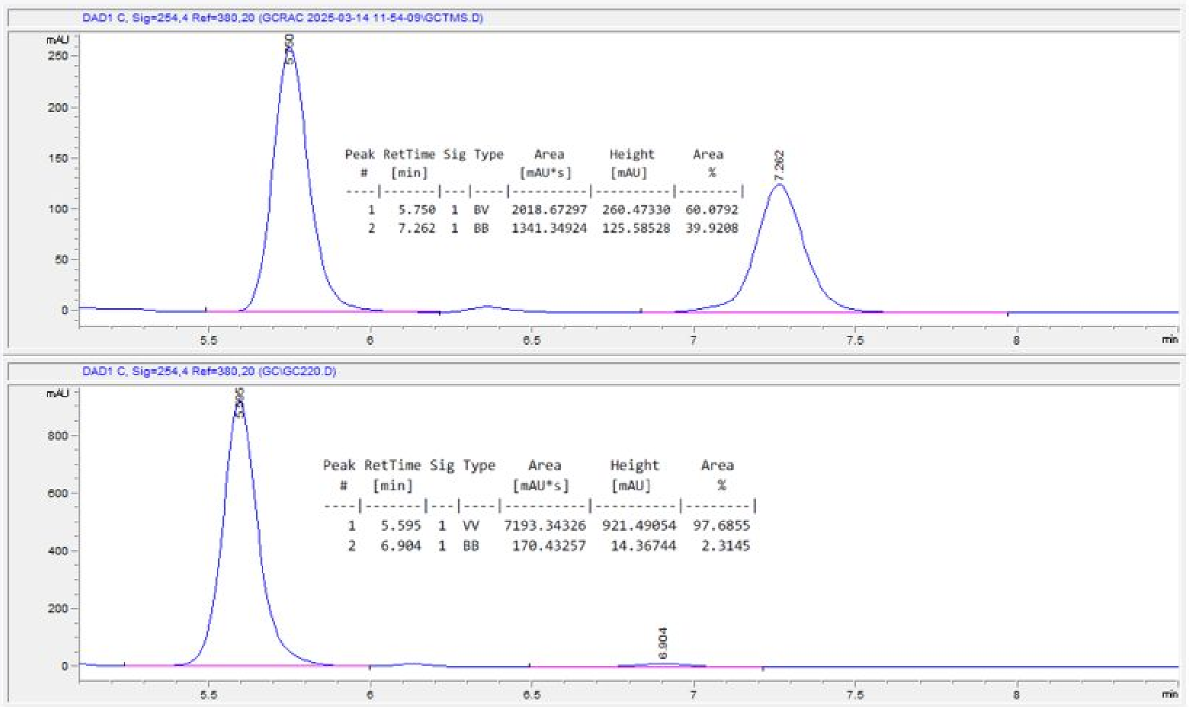

10.2 Chiral HPLC traces of product **14a**

14a: Prepared according to general procedure E using **8b** as organocatalyst. IC column, 90:10 Hex:IPA, 1 mL/min. Rt (min): 16.99 (*anti*), 25.32 (*syn*), 29.66 (*syn*), 33.72 (*anti*).

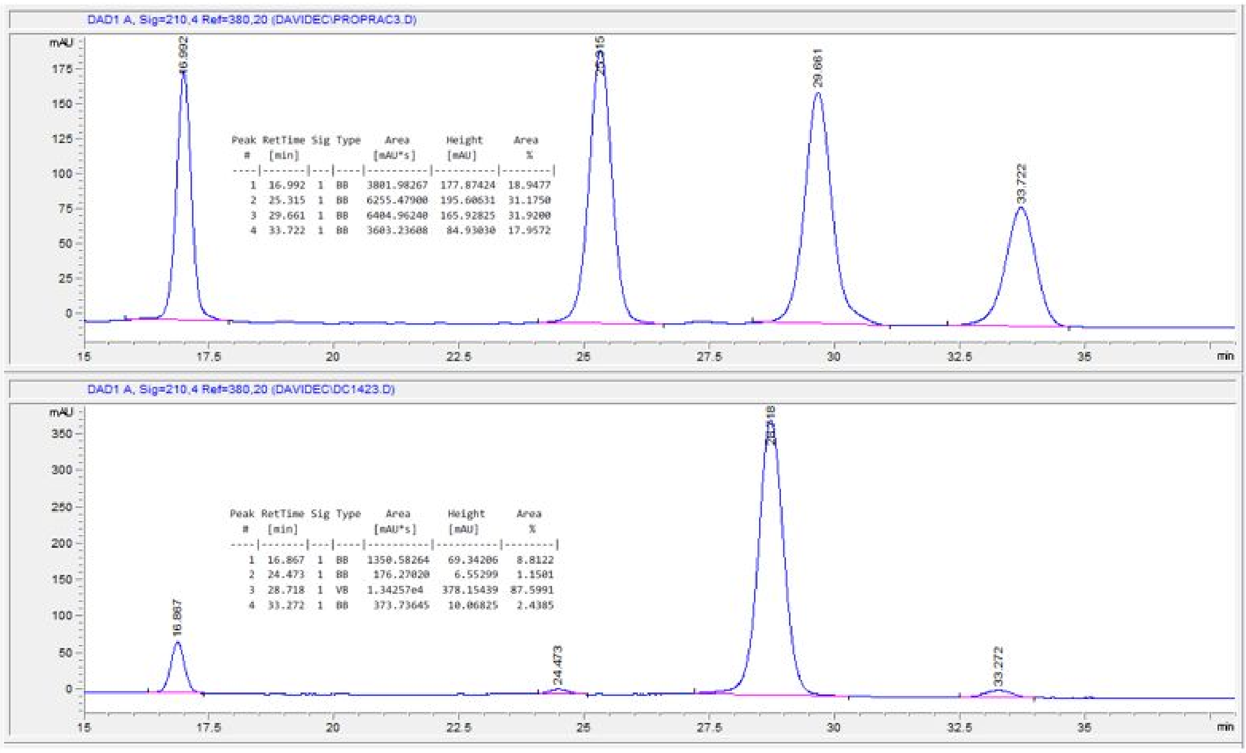

Peak #	RetTime [min]	Sig	Type	Area [mAU*s]	Height [mAU]	Area %
1	16.992	1	BB	3801.98267	177.87424	18.9477
2	25.315	1	BB	6255.47900	195.60631	31.1750
3	29.661	1	BB	6404.96240	165.92825	31.9200
4	33.722	1	BB	3603.23608	84.93030	17.9572

Peak #	RetTime [min]	Sig	Type	Area [mAU*s]	Height [mAU]	Area %
1	16.867	1	BB	1350.58264	69.34206	8.8122
2	24.473	1	BB	176.27020	6.55299	1.1501
3	28.718	1	VB	1.34257e4	378.15439	87.5991
4	33.272	1	BB	373.73645	10.06825	2.4385

14a: Prepared according to general procedure E using **8c** as organocatalyst. IC column, 90:10 Hex:IPA, 1 mL/min. Rt (min): 18.54 (*anti*), 28.85 (*syn*), 34.00 (*syn*), 39.73 (*anti*).

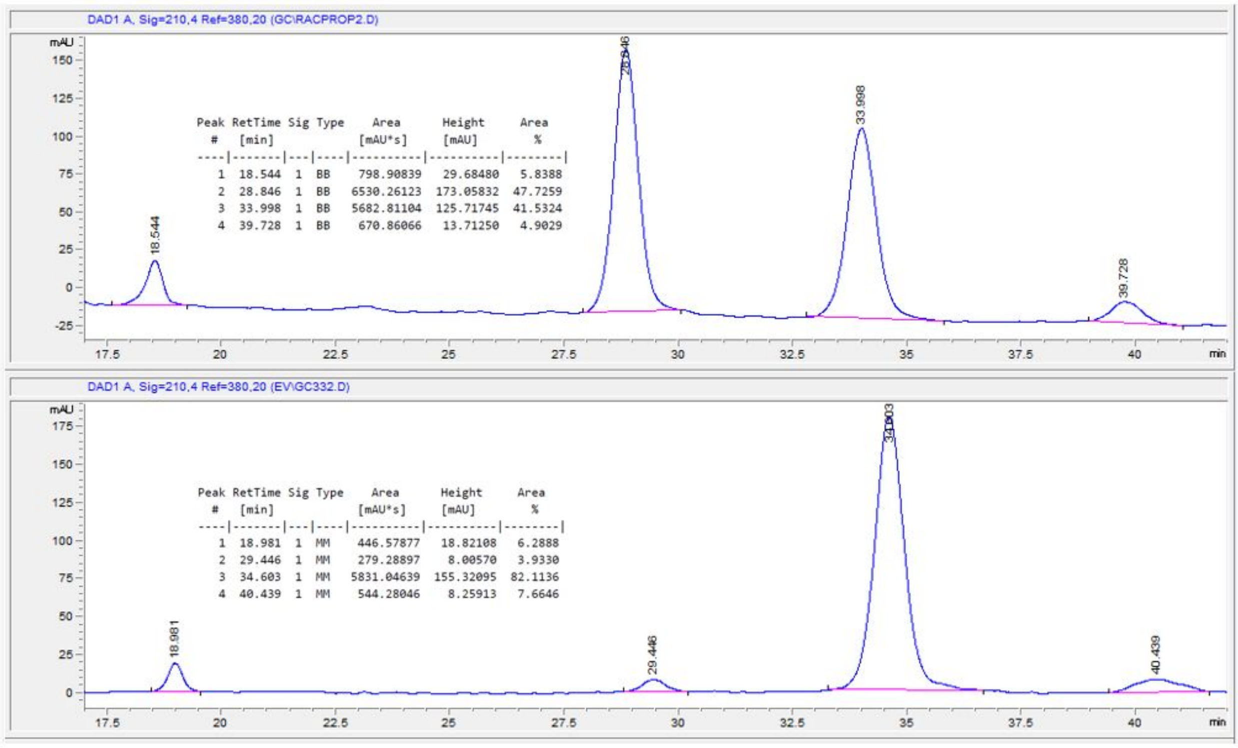

Peak #	RetTime [min]	Sig	Type	Area [mAU*s]	Height [mAU]	Area %
1	18.544	1	BB	798.90839	29.68480	5.8388
2	28.846	1	BB	6530.26123	173.05832	47.7259
3	33.998	1	BB	5682.81104	125.71745	41.5324
4	39.728	1	BB	670.86066	13.71250	4.9029

Peak #	RetTime [min]	Sig	Type	Area [mAU*s]	Height [mAU]	Area %
1	18.981	1	MM	446.57877	18.82108	6.2888
2	29.446	1	MM	279.28897	8.00570	3.9330
3	34.603	1	MM	5831.04639	155.32095	82.1136
4	40.439	1	MM	544.28046	8.25913	7.6646

14a: Prepared according to general procedure E using **8d** as organocatalyst. IC column, 90:10 Hex:IPA, 1 mL/min. Rt (min): 16.99 (*anti*), 25.32 (*syn*), 29.66 (*syn*), 33.72 (*anti*).

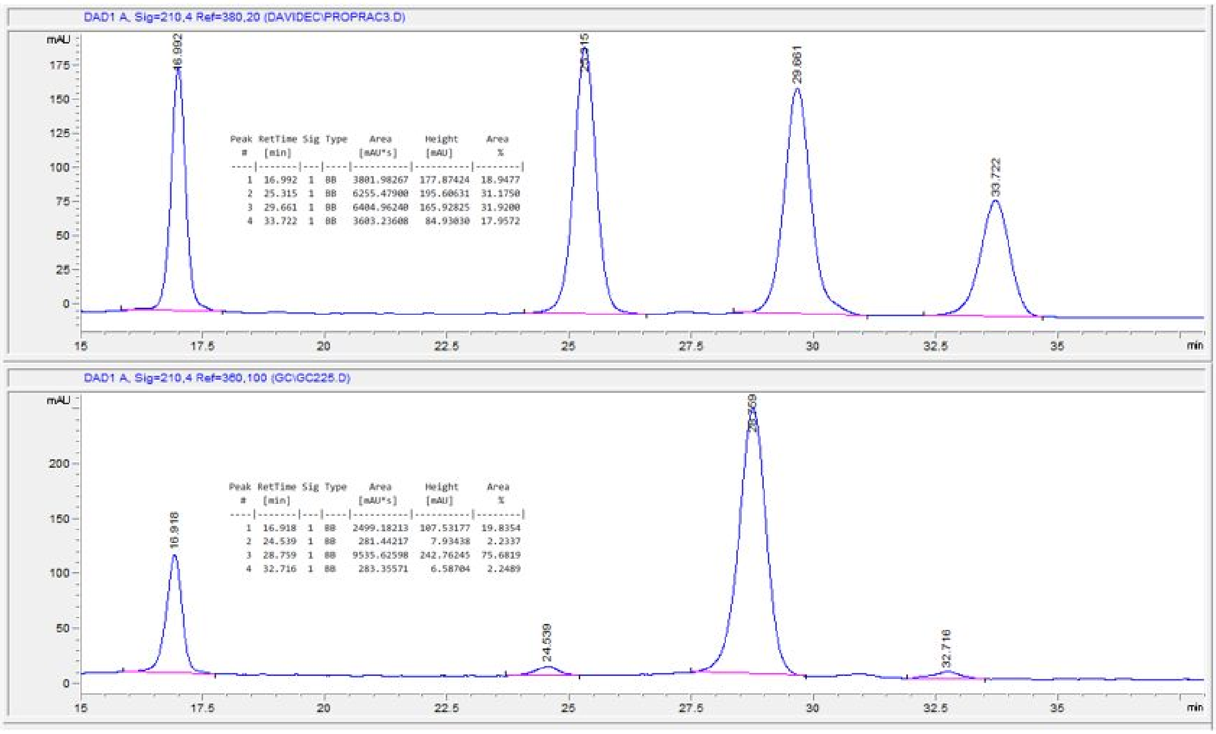

10.3 Chiral HPLC traces of product **14b**

14b: Prepared according to general procedure F using **8b** as organocatalyst. IC column, Hex:IPA 90:10, 0.8 mL/min, Rt (min): 15.57 (*anti*), 20.94 (*anti*), 22.83 (*syn*), 25.97 (*syn*).

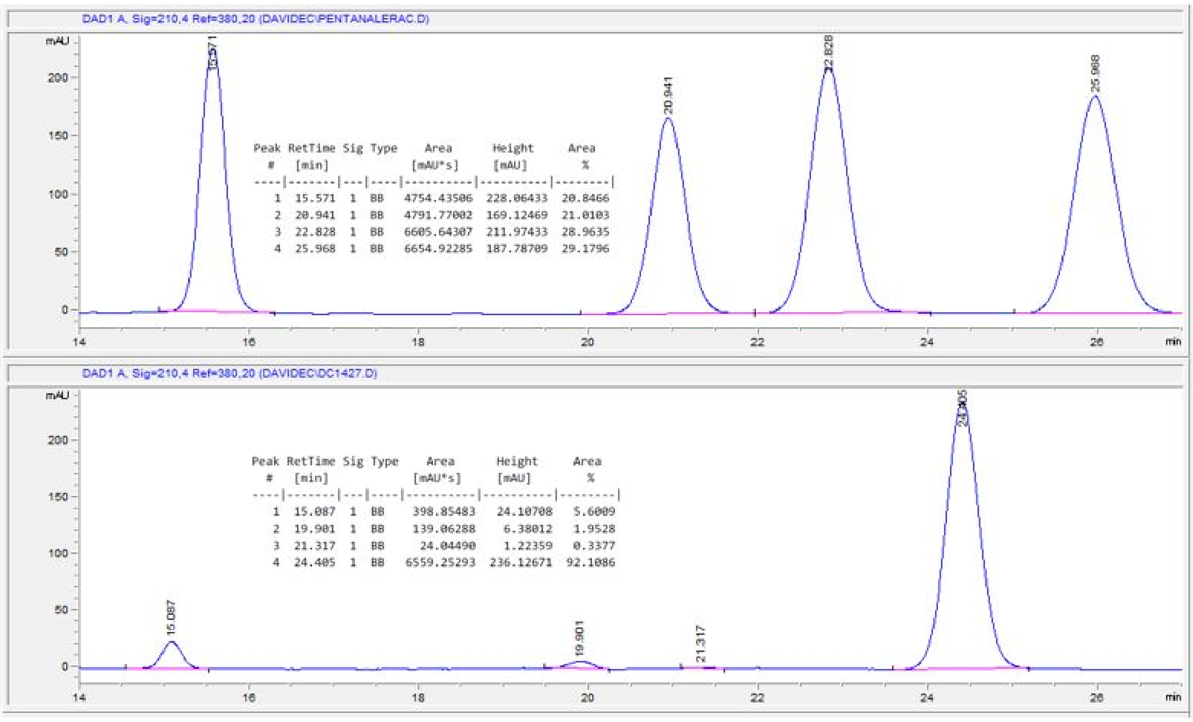

14b: Prepared according to general procedure F using **8c** as organocatalyst. IC column, Hex:IPA 90:10, 0.8 mL/min, Rt (min): 15.57 (*anti*), 20.94 (*anti*), 22.83 (*syn*), 25.97 (*syn*).

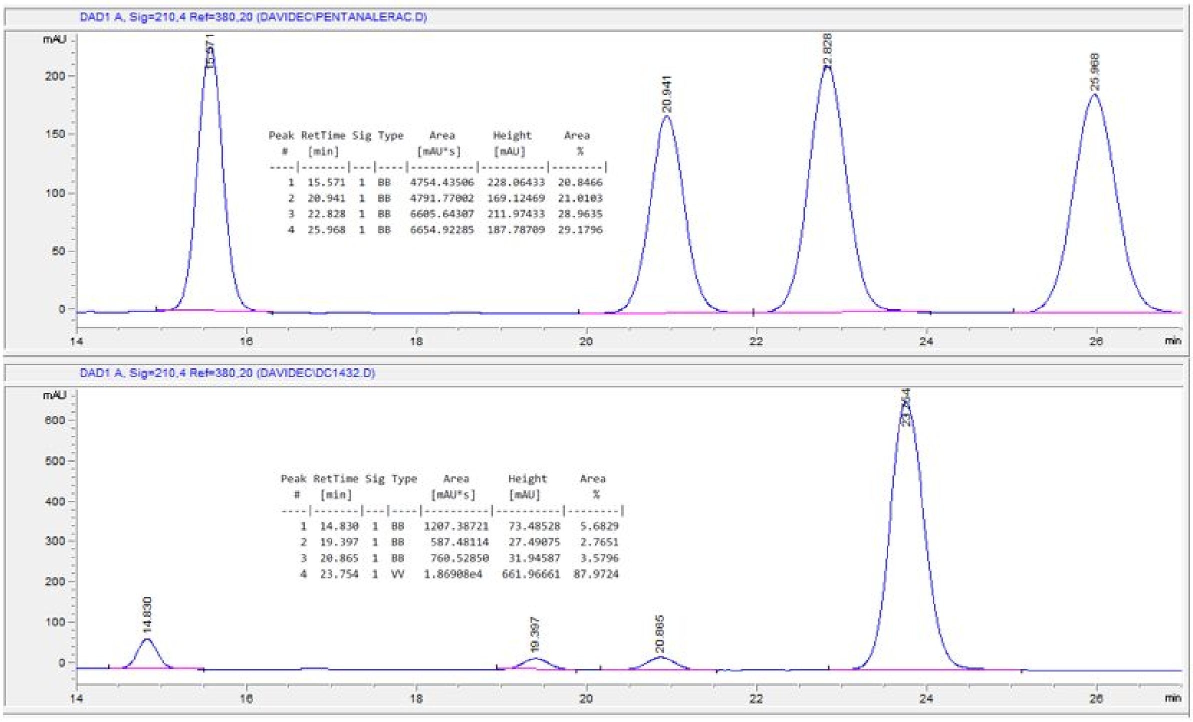

14b: Prepared according to general procedure F using **8d** as organocatalyst. IC column, Hex:IPA 90:10, 0.8 mL/min, Rt (min): 17.72 (*anti*), 25.20 (*anti*), 27.52 (*syn*), 31.13 (*syn*).

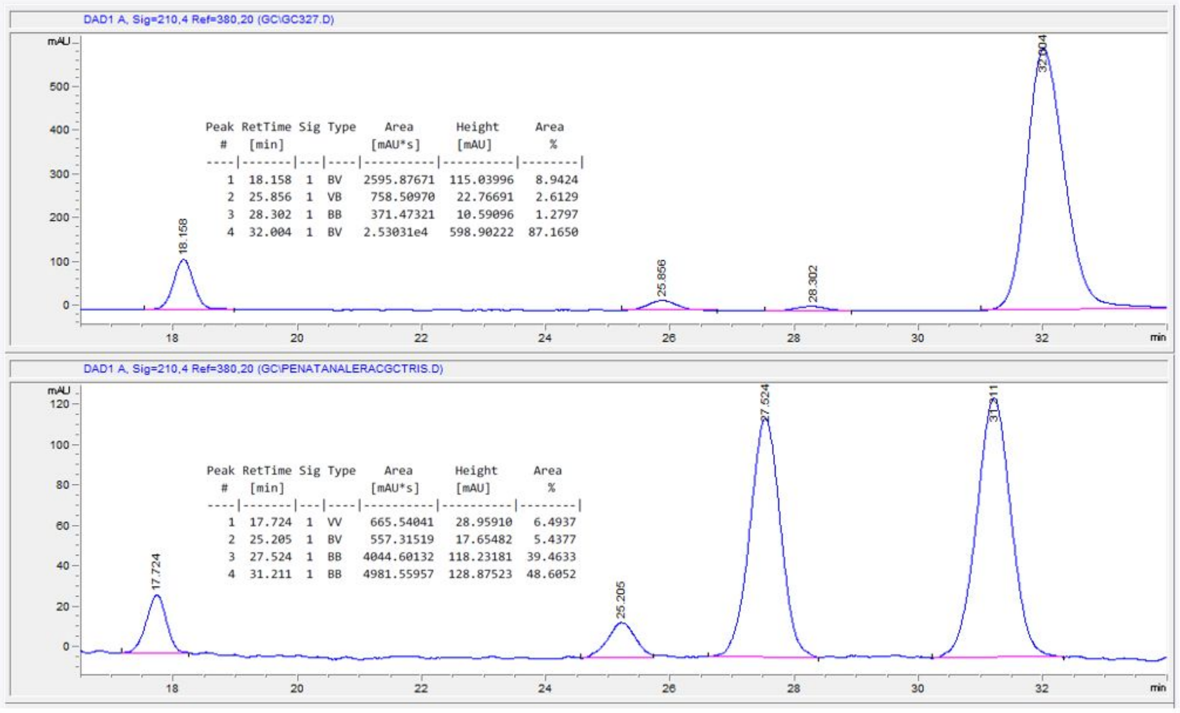

10.4 Chiral HPLC traces of imine **4a**

Prepared according to literature procedure[1]. ODH column, Hex:IPA 90:10, 0.5 mL/min. τ_{minor} = 11.57 min, τ_{major} = 14.35 min.

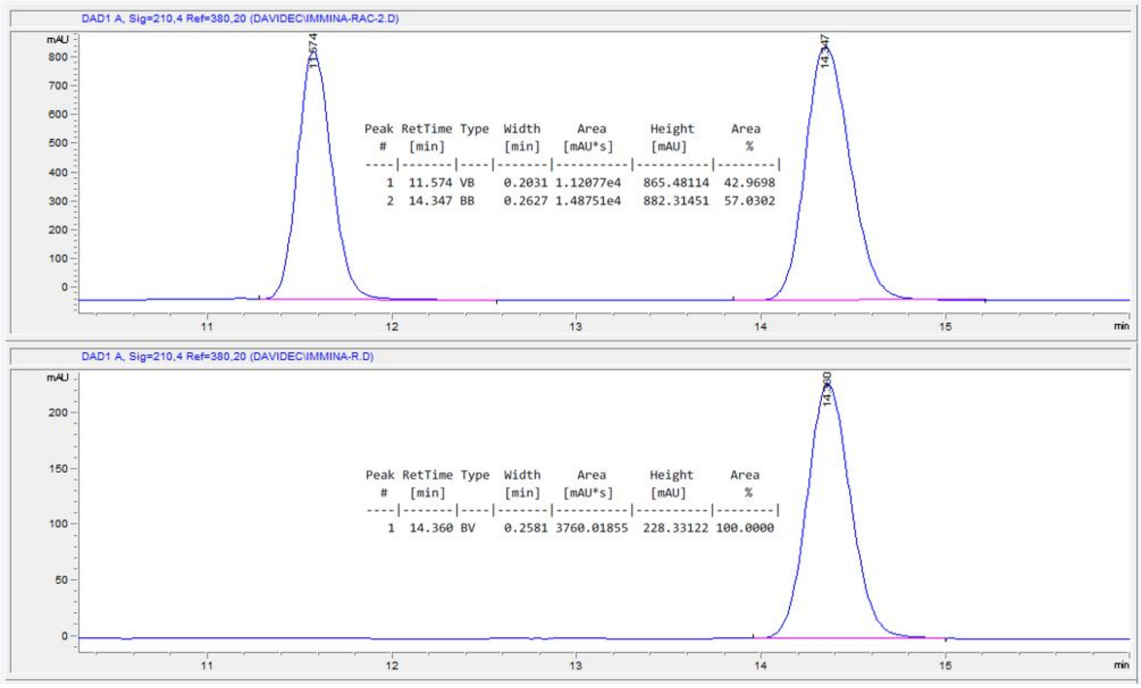

Prepared according to literature procedure[1] and injected after one week storage. ODH column, Hex:IPA 90:10, 0.5 mL/min. τ_{minor} = 11.57 min, τ_{major} = 14.35 min.

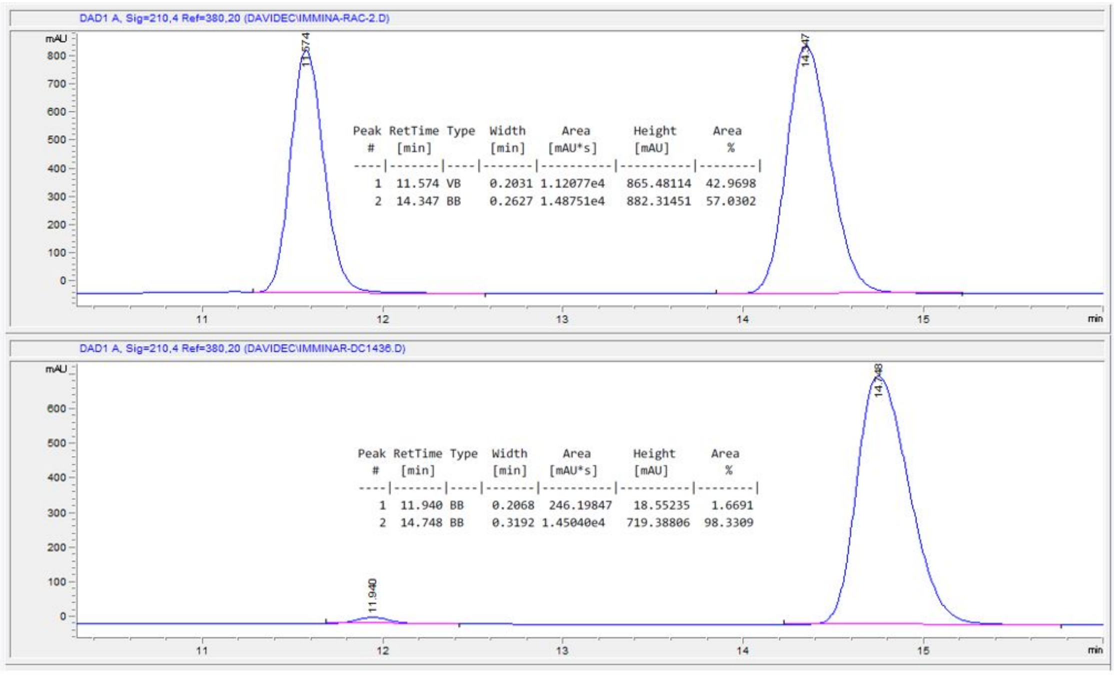

11 Bibliography

[1] Sirvent, A.; Hernández-Ibáñez, S.; Yus, M.; Foubelo, F. "Pyrrolidine and indolizidine alkaloids from chiral *N-tert*-butanesulfinyl imines derived from 4-halobutanal". *Synthesis* **2021**, *53*, 1749–1759.

[2] Reddy, A. A., Prasad, K. R. "Addition of the Lithium Anion of Diphenylmethanol Methyl/Methoxymethyl Ether to Nonracemic Sulfinimines: Two-Step Asymmetric Synthesis of Diphenylprolinol Methyl Ether and Chiral (Diphenylmethoxymethyl)amines". *J. Org. Chem.* **2018**, *18*, 10776–10785.

[3] W. C. Still, "Conjugate Addition of Trimethylsilyllithium. A Preparation of 3-Silyl Ketones", *J. Org. Chem.* **1976**, *41*, 3063–3064.

[4] Patora-Komisarska, K., Benohoud, M., Ishikawa, H., Seebach, D. and Hayashi, Y. "Organocatalyzed *Michael* Addition of Aldehydes to Nitro Alkenes – Generally Accepted Mechanism Revisited and Revised". *Helv. Chim. Acta*. **2011**, *94*, 719-745.

[5] Zhu, S., Yu, S. and Ma, D., "Highly Efficient Catalytic System for Enantioselective Michael Addition of Aldehydes to Nitroalkenes in Water". Angew. Chem Int. Ed., **2008**, *47*, 545-548.

[6] D. Seebach, U. Grošelj, D. M. Badine, W. B. Schweizer, A. K. Beck, "Isolation and X-Ray Structures of Reactive Intermediates of Organocatalysis with Diphenylprolinol Ethers and with Imidazolidinones. A Survey and Comparison with Computed Structures and with 1-Acylimidazolidinones: The 1,5-Repulsion and the Geminal-Diaryl Effect at Work", *Helv. Chim. Acta* **2008**, *92*, 1999–2034.

[7] U. Grošelj, D. Seebach, D. M. Badine, W. B. Schweizer, A. K. Beck, I. Krossing, P. Klose, Y. Hayashi, T. Uchimaru, "Structures of the Reactive Intermediates in Organocatalysis with Diarylprolinol Ethers", *Helv. Chim. Acta* **2009**, *92*, 1225–1259.

[8] Gaussian 16, Revision C.01, Frisch, M. J.; Trucks, G. W.; Schlegel, H. B.; Scuseria, G. E.; Robb, M. A.; Cheeseman, J. R.; Scalmani, G.; Barone, V.; Petersson, G. A.; Nakatsuji, H.; Li, X.; Caricato, M.; Marenich, A. V.; Bloino, J.; Janesko, B. G.; Gomperts, R.; Mennucci, B.; Hratchian, H. P.; Ortiz, J. V.; Izmaylov, A. F.; Sonnenberg, J. L.; Williams-Young, D.; Ding, F.; Lipparini, F.; Egidi, F.; Goings, J.; Peng, B.; Petrone, A.; Henderson, T.; Ranasinghe, D.; Zakrzewski, V. G.; Gao, J.; Rega, N.; Zheng, G.; Liang, W.; Hada, M.; Ehara, M.; Toyota, K.; Fukuda, R.; Hasegawa, J.; Ishida, M.; Nakajima, T.; Honda, Y.; Kitao, O.; Nakai, H.; Vreven, T.; Throssell, K.; Montgomery, J.A.; Jr., Peralta, J. E.; Ogliaro, F.; Bearpark, M. J.; Heyd, J. J.; Brothers, E. N.; Kudin, K. N.; Staroverov, V. N.; Keith, T. A.; Kobayashi, R.; Normand, J.; Raghavachari, K.; Rendell, A. P.; Burant, J. C.; Iyengar, S. S.; Tomasi, J.; Cossi, M.; Millam, J. M.; Klene, M.; Adamo, C.; Cammi, R.; Ochterski, J. W.; Martin, R. L.; Morokuma, K.; Farkas, O.; Foresman, J. B.; Fox, D. J. Gaussian, Inc., Wallingford CT (2016).

[9] Legault, C. Y.; CYLView, Université de Sherbrooke: Sherbrooke, Quebec, Canada, 2009; http://www.cylview.org.
